# Formation of cyclopentanes and cyclopropanes through alkylation of benzylic anions using ethers, thioethers and alcohols as substrates under Grubbs–Stoltz (Et_3_SiH/KO*t*Bu) conditions

**DOI:** 10.1039/d5sc10055k

**Published:** 2026-03-10

**Authors:** Alexander J. Stewart, Daniela Dimitrova, Scott T. M. Logan, Cassie Pratley, Jonathan D. Bell, Katy McGonigal, Anna Lauer, Sabine Fenner, Simon M. Nicolle, Stuart G. Leach, John A. Murphy

**Affiliations:** a Department of Pure and Applied Chemistry, University of Strathclyde 295 Cathedral Street Glasgow G1 1XL UK john.murphy@strath.ac.uk; b GSK Medicines Research Centre Gunnels Wood Road, Stevenage Herts SG1 2NY UK

## Abstract

Reaction of diarylmethanes with the Grubbs–Stoltz reagent (KO*t*Bu + Et_3_SiH) using THF as solvent led to diarylcyclopentanes through an unprecedented double-alkylation reaction, with four of the carbons of the cyclopentane coming from THF. In like manner, reaction of diarylmethanes with the same reagent in 1,4-dioxane as solvent led to double-alkylation to form diarylcyclopropanes, with two of the cyclopropane carbons coming from 1,4-dioxane. Monoalkylated substrates that were likely intermediates on the dialkylation pathway were subjected to the same conditions, leading to cyclisations to form cyclopentanes and cyclopropanes. The cyclisation chemistry also extended to formation of monoarylcyclopropanes from reaction of the corresponding benzylpotassium reagents with ethers, alcohols, sulfides, sulfoxides and sulfones.

## Abstract 2

Reaction of diarylmethanes with the Grubbs–Stoltz reagent (KO*t*Bu + Et_3_SiH) using THF as solvent led to diarylcyclopentanes through an unprecedented double-alkylation reaction, with four of the carbons of the cyclopentane coming from THF.

## Graphical abstract

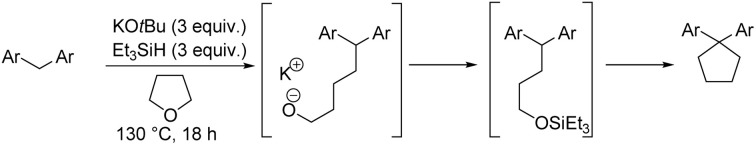

## Introduction

**Scheme 1:**
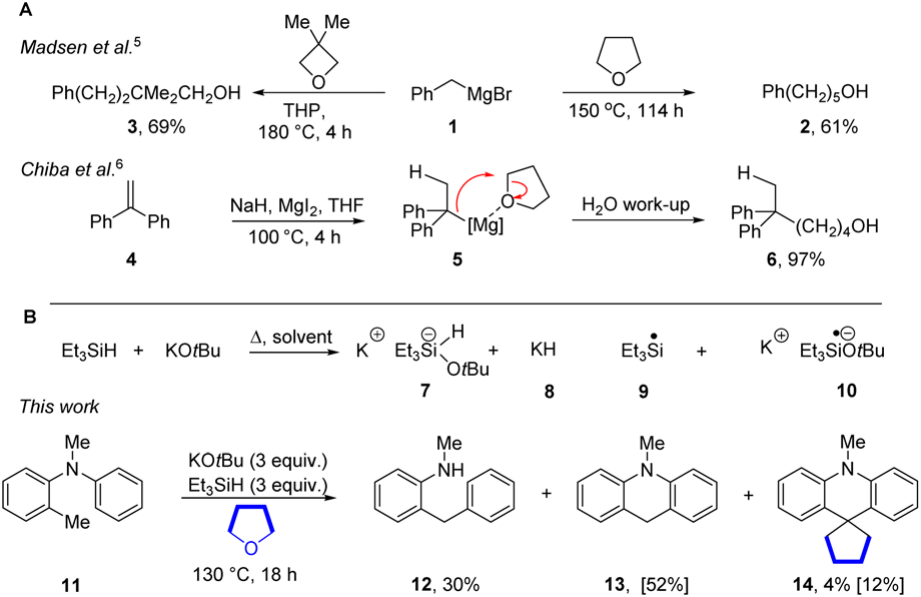
(A) Prior reactions of benzylic organomagnesium reagents; (B) our discovery of cyclopentane formation from THF (NMR yields in brackets).

Alkylations of carbanions under basic conditions routinely use very reactive electrophiles such as alkyl halides or sulfonate esters. The high reactivity of these reagents makes them convenient to use, but also underpins their ability to alkylate biomolecules, leading to their classification as major safety hazards. Ethers are much less electrophilic – indeed they are usually not recognised as electrophiles in the absence of protic or Lewis acids that activate them to C–O bond cleavage. If ethers could routinely be used as alkylating agents under defined basic conditions, this would be a very useful advance. THF has been deconstructed in many ways in the literature^1,2^ but it has never been subject to a double nucleophilic substitution by carbanions. Substitution of a single C–O bond cleavage has been reported using conventional powerful Lewis acids.^3,4^ Madsen *et al.* showed,^5^ using organomagnesium compounds as both Lewis acids and nucleophiles, that allylic and benzylic Grignard reagents such as 1 open THF and 3,3-dimethyloxetane to form alcohol products *e.g.*2 and 3 respectively (Scheme 1A). In these cases, the Lewis acidic properties of the magnesium atom are harnessed to assist ring-opening.^5^ Very recently, Chiba *et al.* have generated MgH_2_*in situ* from magnesium halides and sodium hydride and used it to add to 1,1-diarylethenes (*e.g.*4) to generate benzylic organomagnesium intermediates 5 that reacted with cyclic ethers to form alcohol products 6.^6^ We now report activation of ethers in the presence of benzylic potassium reagents; here, THF and dioxane each act as dual electrophiles in forming cyclopentanes and cyclopropanes respectively.

## Results and discussion

The Grubbs–Stoltz reagent, formed by heating a mixture of KO*t*Bu and Et_3_SiH,^7^ has been widely investigated since its introduction in 2013. This reagent is most unusual since it simultaneously generates a number of reactive intermediates, 7–10, that perform hydride transfer, hydrogen atom transfer, deprotonation, radical chemistry, silylation and electron transfer chemistry (Scheme 1B). Simultaneous exposure of substrates to such a variety of reactive entities is very unusual, and has been shown to lead to diverse and novel outcomes.^7–19^ In the course of our studies, we observed the rearrangement of the *N*-methyl-*N-o*-tolylaniline 11 to diarylmethane 12 and dihydroacridine 13; the mechanisms of the formation of 12 and 13 have been investigated and reported.^16,17^ However, in addition to these products, and relevant to this paper, cyclopentane 14 (12%) was also formed (Our reactions were routinely performed in sealed vessels).

The reaction of 11 was repeated with THF-*d*_8_ as solvent and led to 14-*d*_8_ (13%) confirming the origin of the cyclopentane. In a further experiment, this time using a 1 : 1 ratio of THF and THF-*d*_8_, the product 14 was a mixture of *d*_8_ and *d*_0_ isotopologues, with no evidence of H/D exchange. If such exchange had been seen, it would have been evidence of THF-derived radicals playing a part in the reaction. The result instead now focused our thoughts on a double aliphatic nucleophilic substitution of THF.

**Scheme 2:**
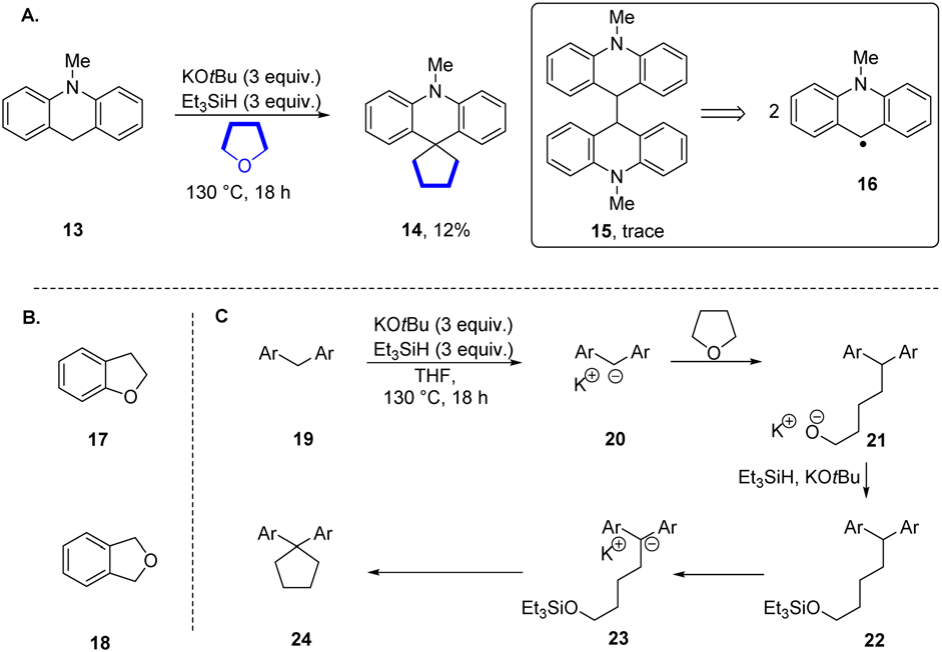
(A) *N*-Methyldihydroacridine 13 as substrate. (B) Dihydrobenzofuran 17 and phthalan 18. (C) Mechanistic proposal for formation of cyclopentane 24.

Our working hypothesis was that dihydroacridine 13 was a likely precursor of 14. In support of this, when the dihydroacridine 13 was heated with KO*t*Bu and Et_3_SiH in THF, the cyclopentane 14 (12%) was again formed (Scheme 2A). In addition, a trace of the dimer 15 was detected by GCMS, indicating a slower background radical chemistry.^13–17^ Abstraction of an H-atom from dihydroacridine 13*e.g.* by triethylsilyl radicals 9 would form a stabilised acridinyl radical 16. Such radicals could then dimerise to afford 15. Interestingly, when 2-methylTHF was used as solvent, the reaction afforded only the dimeric product 15 (64%) and when tetrahydropyran (THP) was used as solvent, no alkylation was detected and product 15 was again seen (20%). The different behaviour of 2-methylTHF was surprising.^20^ The inertness of THP to this reaction allowed us to use it as solvent in some later reactions.^21^ Before studying intermediates in this reaction, we tested dihydrobenzofuran 17 and phthalan 18 as substrates (Scheme 2B). We recognised that oxygen-containing heterocycles fused to arene rings can undergo electron transfer from 10 leading to reduction to their arene radical anions, followed by C–O bond cleavage.^7^ Both led to complex mixtures.

Our proposed mechanism for cyclopentane formation is shown in Scheme 2C. Deprotonation of diarylmethane 19 would afford diarylmethyl potassium 20 which reacts with THF to afford intermediate alkoxide 21. To proceed to the cyclopentane, this alkoxide would require protection as silyl ether 22. It is known that when alkoxides are treated with triethylsilane or other silanes, silyl ethers are formed.^22–24^ The silyl alkyl ether component of 22 can then behave as an alkylating agent, reacting intramolecularly in this case with the anion in 23 to form the cyclopentane 24.

To test this proposal, alcohol 25, silyl ether 26 and methyl ether 27 were prepared and subjected to the reaction conditions (Table 1, entries 1–3). All three compounds were converted to the cyclopentane 28 (27–77% yields). The formation of cyclopentanes in this way is novel – the cyclisations of silyl ether 24 and methyl ether 27 proceed with very unusual but credible leaving groups.

**Table 1:** Exploring cyclisation reactions to form diphenylcyclopentane 28

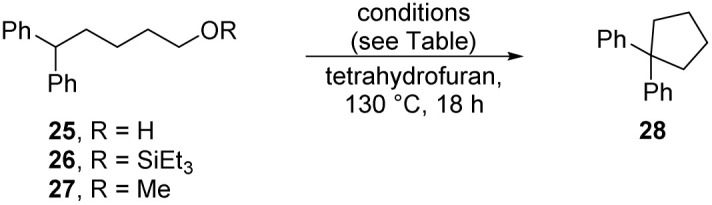							
Entry	R	Solvent	Et_3_SiH	Base	Et_3_SiO*t*Bu	28 (yield)	26 (yield)
1	H	THF	3 equiv.	KO*t*Bu – 3 equiv.	—	50%	—
2	SiEt_3_	THF	3 equiv.	KO*t*Bu – 3 equiv.	—	77%	—
3	Me	THF	3 equiv.	KO*t*Bu – 3 equiv.	—	27%	—
4	H	THF	3 equiv.	KO*t*Bu – 4 equiv.	3 equiv.	17%	—
5	SiEt_3_	THF	—	KO*t*Bu – 3 equiv.	—	—	36%^a^
6	H	THF	3 equiv.	NaO*t*Bu – 4 equiv.	—	—	3%
7	H	THF	3 equiv.	LiO*t*Bu – 4 equiv.	—	—	76%
8	H	THF	3 equiv.	NaH – 4 equiv.	—	26%	36%
9	H	THF	3 equiv.	KH – 4 equiv.	—	67%	—
10	H	2-MeTHF	3 equiv.	KO*t*Bu – 4 equiv.	—	49%	—

a From this reaction, alcohol 25 (50%) was also isolated.

A byproduct in these reactions was Et_3_SiO*t*Bu (Scheme 1B). This compound was tested for its effect on the cyclisation (entry 4). Specifically, this was to probe whether this silyl ether would provide some Lewis acid assistance to the cyclisation. However, this led to a lower yield (17%) for the cyclisation and hence does not facilitate the reaction.

Interestingly, when the silyl ether 26 was subjected to reaction with KO*t*Bu (entry 5), but in the absence of triethylsilane, no cyclisation was observed. This could be due to either or both of two reasons: (i) the silyl ether substrate 26 could be attacked by the excess KO*t*Bu to form Et_3_SiO*t*Bu and the diphenylbutoxide anion derived from the substrate. The alkoxide would then not function as an electrophile, and no cyclisation would be seen [This would be consistent with the isolation of 25 (50%) from this reaction]. The alternative possibility was that KO*t*Bu was not a strong enough base to deprotonate 26, but that when cyclopentanes had successfully formed in previous cases, that this had resulted from the generation of a stronger base, *i.e.* KH, 8, through interaction of KO*t*Bu with Et_3_SiH.^22–24^ It has been shown that LiO*t*Bu reacts with triethylsilane to liberate LiH^25^ and we have previously shown^13^ that inorganic potassium amides also react with triethylsilane to afford KH; this species would be a much stronger base than KO*t*Bu and should certainly deprotonate 26.

Accordingly, the role of base was explored with alcohol 25 using NaO*t*Bu, LiO*t*Bu, NaH and KH (entries 6–9 respectively). NaO*t*Bu reacted little, perhaps due to insolublilty; LiO*t*Bu converted the alcohol 25 to its silyl ether 26. The stronger base, NaH, formed a mixture of silyl ether 26 and cyclopentane 28 while KH made a more effective conversion to the cyclopentane product. We have recently observed that potassium salts demonstrate special reactivity relative to their sodium and lithium counterparts in a number of reaction types, and in some cases, we have identified the specific interactions responsible.^26,27^

Finally, 2-MeTHF was studied (entry 10) as an alternative solvent for the intramolecular alkylation of alcohol 25. Having seen the dramatic differences between THF and 2-methylTHF in the reaction of dihydroacridine 13 above, we were not confident of the outcome. However, in the complex reaction seen with Grubbs–Stoltz conditions, the interplay of kinetics and thermodynamics of the different possible processes, mean that it was valuable to explore this. In effect, the reaction with 25 afforded 28 (49%), similar to the yield seen with THF as solvent (55%, entry 1). So, this shows that THF and 2-MeTHF were equally suitable solvents for reactions that involve intramolecular alkylation.

**Scheme 3:**
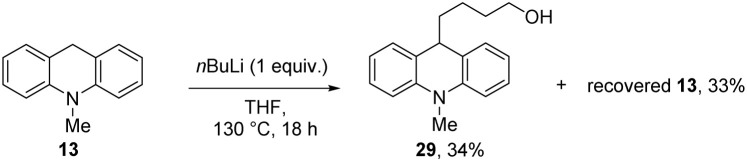
Action of strong bases on substrates with benzylic C–H.

In our case, K^+^ or an organopotassium species would be a much milder Lewis acid than magnesium ions^5,6^ and our substrates show that magnesium ions are not required for these reactions. Based on these thoughts, a further experiment with base was undertaken. *n*BuLi was examined as base, using *N*-methyldihydroacridine 13 as substrate and alcohol 29 (34%) was isolated following workup; here, the reaction was likely assisted by the Lewis acidity of the lithium species (Scheme 3). Further reaction of the alkoxide was not achieved in the absence of silane.

**Scheme 4:**
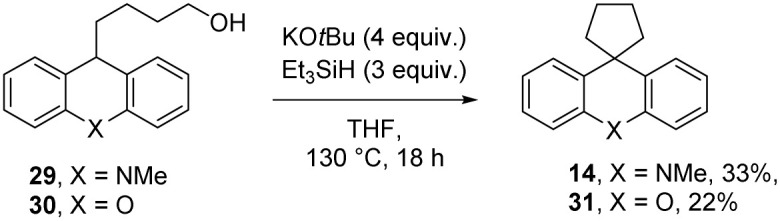
Alcohols as substrates for cyclopentane formation in the presence of silanes.

Returning to Grubbs–Stoltz conditions (KO*t*Bu + Et_3_SiH), substrates 29 and 30 were then subjected to the cyclisation reaction, leading to the cyclopentanes 14 and 31 in the yields shown in Scheme 4. We did not isolate other products from the reaction, but diaryl ethers have previously been shown to undergo cleavage of Ar–O bonds, likely resulting following electron transfer from radical anion 10.^7^ Electron transfer to the oxygen heterocycle of 30 should be easier than to the more electron-rich nitrogen heterocycle of 29, and this could underpin the higher yield of cyclopentane from substrate 29.

**Scheme 5:**
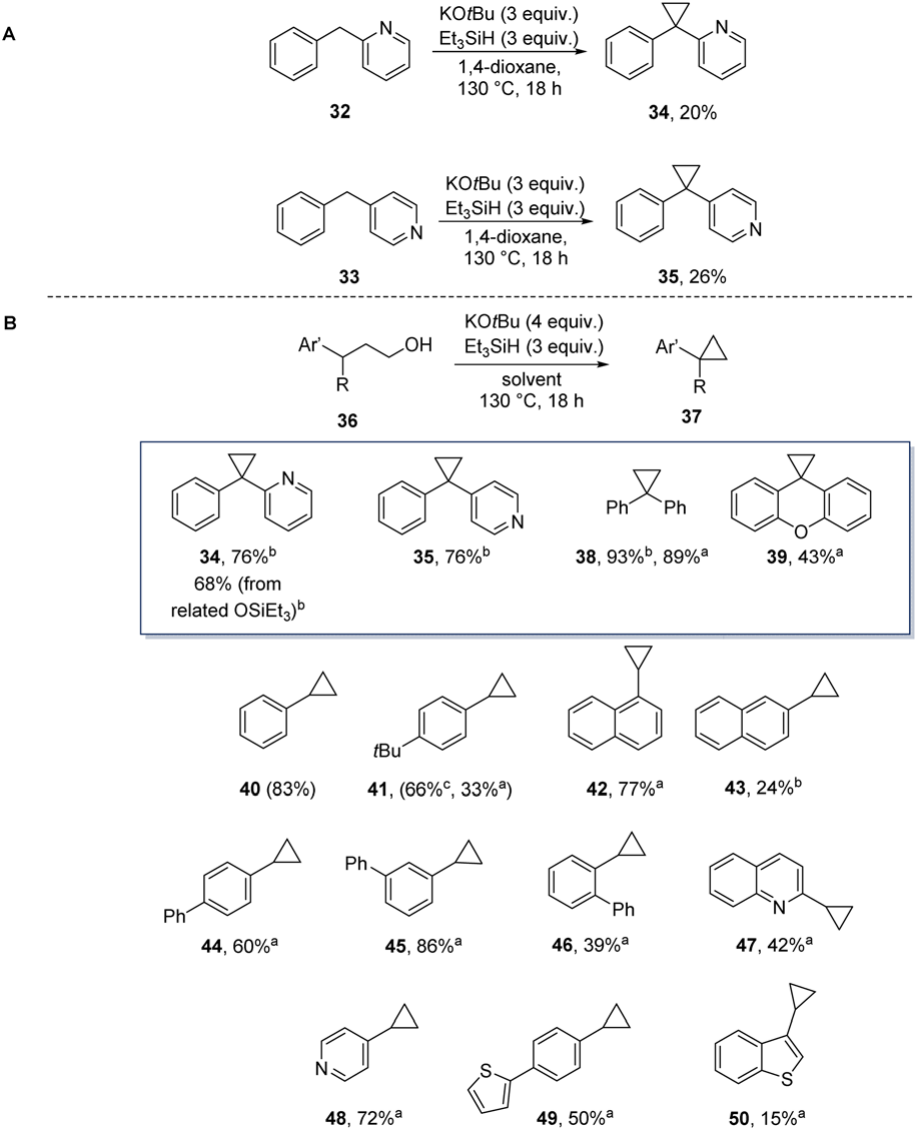
Cyclopropane formation (A) from dioxane solvent and (B) from hydroxypropyl arenes under Grubbs–Stoltz conditions. ^*a*^In THF as solvent, ^*b*^in dioxane, ^*c*^in THP.

Our first encounter with cyclopropane formation arose when 1,4-dioxane was used as solvent. This converted benzylpyridines 32 and 33 to products 34 and 35 (Scheme 5A). Again, the cyclopropane formation likely arose through sequential substitution reactions on ether C–O bonds. By analogy with the THF case, our proposal was that initial alkylation had afforded a 3,3-diarylpropyl alkyl ether. This led us to select 3,3-diarylpropanols (36, R=Ar′) as substrates for cyclopropane formation, leading to 34, 35, 38 and 39 in moderate to excellent yields (Scheme 5B). Clearly, diphenylmethane anions have the right balance of nucleophilicity and basicity to succeed in substitution reactions with the ethers. To extend the scope, simpler benzyl anions were investigated; hydroxypropylbenzenes (36, R=H) were then chosen as substrates, affording arylcyclopropanes 41–50 in a range of yields (15–89% yield) (The simplest substrate (36, Ar = Ph, R=H) cyclised cleanly to 40, giving an NMR yield (83%) but the product was too volatile to get an isolated yield.).

We had previously tested whether cyclopentanations could be achieved by simply using a strong base, KH; this was now investigated for cyclopropanations. Accordingly, substrates 51 (R = Ph, Me, Et) were treated with commercial KH, with THF as solvent, respectively affording product 38 in 49, 30 and 69% yield (Table 2, entries 1–3). Thus, KH as strong base can achieve these reactions. The yields are lower than when KH acts in conjunction with Et_3_SiH, as seen in comparing entries 4 and 5. Although we don’t have evidence for the cause of this at this stage, it may relate to aggregation state of KH. Chiba *et al.* have recently shown that the presence of additives can affect both the aggregation state and the reactivity of NaH.^29^

**Table 2:** Formation of cyclopropanes

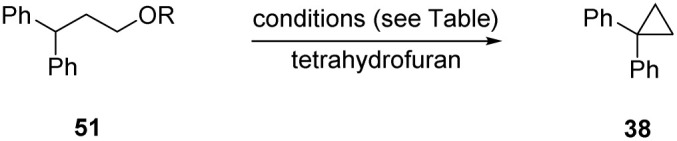							
Entry	R	Solvent	Et_3_SiH	Base	Temp °C	Duration	38 (yield)
1	Me	THF	—	KH – 3 equiv.	130 °C	18 h	49%
2	Et	THF	—	KH – 3 equiv.	130 °C	18 h	30%
3	Ph	THF	—	KH – 3 equiv.	130 °C	18 h	69%^a^
4	SiEt_3_	THF	—	KH – 3 equiv.	130 °C	18 h	34%
5	SiEt_3_	THF	3 equiv.	KH – 3 equiv.	130 °C	18 h	70%
6	H	THF	3 equiv.	KH – 4 equiv.	130 °C	18 h	82%
7	H	THF	3 equiv.	KH – 4 equiv.	100 °C	18 h	67%
8	H	THF	3 equiv.	KH – 4 equiv.	80 °C	18 h	70%
9	H	THF	3 equiv.	KH – 4 equiv.	60 °C	18 h	51%
10	H	THF	3 equiv.	KH – 4 equiv.	80 °C	3 h	42%

a See ref. 28 for formation of cyclopropanes through displacement of aryloxide leaving groups.

Entries 6–9 examine the effect of temperature on the cyclisation of 51, R=H. The reaction is clearly more effective at 130 °C than at lower temperatures. Entry 10 shows the outcome of the reaction at 80 °C but for a 3 hour duration (*cf.* entries 6 and 10).

The role of Et_3_SiH was next examined in Table 3 (Et_3_SiH appeared in Table 2, entry 5, yield 70%). Replacement of Et_3_SiH by Me_2_PhSi led to a similar outcome (76% yield) in the formation of cyclopropane 38 (R=H). However, more bulky silanes iPr_3_SiH and *t*BuMe_2_SiH led to silylation of the alcohol as silyl ether 52, but not to cyclisation, likely due to steric hindrance in the cyclisation step. When tri-*n*-butylstannane was used instead of a silane, then only starting alcohol 51 was isolated.

**Table 3:** Exploration of scope of silanes

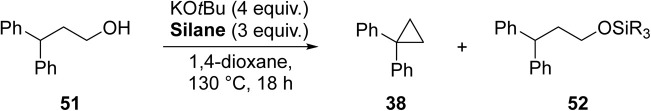			
Silane or equivalent	51 (R=H)	38	52
iPr_3_SiH	—	—	100%
Me_2_PhSiH	—	76%	—
*t*BuMe_2_SiH	—	—	89%
Bu_3_SnH	89%	—	—

**Scheme 6:**
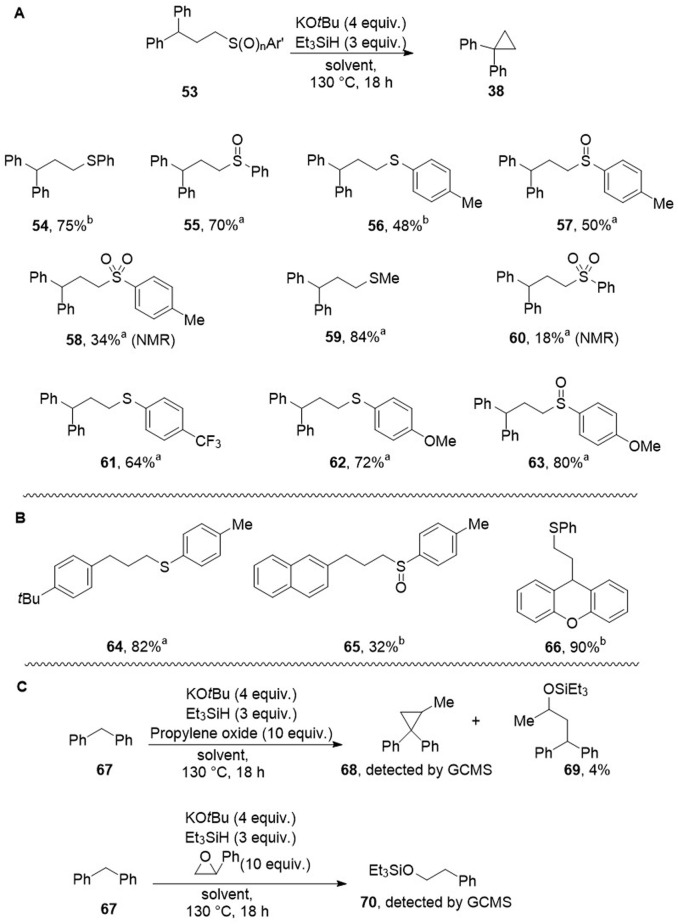
(A) Substrates 54–63 are converted to cyclopropane 38 in the yields shown; (B) substrates 64–66 are converted into the corresponding cyclopropanes. ^*a*^In THF as solvent, ^*b*^in dioxane. (C) Reactions of epoxides.

Having seen that ethers behaved favourably, thioethers were next assayed. Scheme 6 shows that cyclopropanes were again formed from sulfides, sulfoxides and sulfones. Thioethers are not viewed as electrophiles in the absence of specific activations. However, conversion of sulfides 53 to cyclopropanes 38 was also achieved. Scheme 6 shows yields (48% to 90%) of cyclopropanes derived from substrates including sulfoxides 55, 57, and 63. Sulfones 58, 60 also underwent cyclopropane formation on treatment by base in the presence of silane. The sulfones were generally less successful than the corresponding sulfides and sulfoxides, likely due to the relative ease of deprotonation α-to sulfones in competition with deprotonation of the benzylic C–H sites.

Our recent results with the Grubbs–Stoltz reagent have underlined the competition between the different types of processes that can result from intermediates that are generated, notably radical chemistry and electron transfer chemistry. Besides the formation of 15, mentioned earlier in relation to Scheme 2, the SI file exemplifies some side-reactions that can be attributed to competing processes (see pp. S45–46).

The rates of formations of cycloalkanes of different ring sizes by intramolecular displacement by nucleophiles have been studied for a number of different classes of compounds. In each case, there is a balance between enthalpic and entropic considerations. Accordingly, having witnessed formation of three-membered and five-membered ring originally arising from use of tetrahydrofuran and 1,4-dioxane as solvents, we were interested in exploring possible formation of 4-membered and 6-membered rings. In fact, these efforts led to only traces of cyclisation and the details of products are more fully discussed in the SI file (see page S57 et seq.) In these cases, the appearance of alternative products likely results from slower cyclisation kinetics compared to the 3- and 5-membered rings discussed in this paper.^30,31^ We also examined two epoxides as substrates, namely propylene oxide and styrene oxide, under our Grubbs–Stoltz conditions (Scheme 6C). Propylene oxide afforded a low yield of silyl ether 69 and trace quantities of cyclopropane 68 were detected by GCMS. With styrene oxide, silyl ether 70 was detected, arising from reductive opening of the styrene oxide. At least with these examples, it thus appears that reactions other than *S*_N_2 attack by benzylic anions occur preferentially.

## Conclusions

Initial observations showed diarylcyclopentane formation on treatment of diarylmethanes with the Grubbs–Stoltz reagent (KO*t*Bu + Et_3_SiH) when THF was used as solvent. The transformation featured two sequential substitution reactions involving cleavage of C–O bonds, with four of the carbons of the cyclopentane coming from THF. Similar reactivity was shown by 1,4-dioxane as solvent, where double-alkylation led to diarylcyclopropanes, with two of the cyclopropane carbons coming from 1,4-dioxane. In these cases, the intermediate alkoxides react with the silane to form silyl ethers, thereby allowing R_3_SiO^−^ anions to act as leaving groups in the cyclisation steps. With appropriate substrates, deprotonation of benzylic C–H bonds with potassium bases afforded anions that underwent intramolecular substitution reactions at ethers or silyl ethers to form cyclopentanes and cyclopropanes. The chemistry also extended to formation of cyclopropanes from substitution reactions at sulfides, sulfoxides and sulfones. Our examples show relatively simple cyclopropanes and cyclopentanes – in view of the importance of these ring systems, we are currently examining the expansion of the scope of this chemistry.

## Author contributions

AJS, DD, STML, CP, JDB, KMcG, ALK performed the experiments. SF, SMN, SGL and JAM supervised the research. All authors analysed and commented on the results. AJS, DD and STML drafted the manuscript. All authors contributed to the final draft and approved the paper.

## Conflicts of interest

CP and DD are now employees of AstraZeneca and may or may not own stock options.

## Supplementary Material

SC-OLF-D5SC10055K-s001

## Data Availability

Supplementary information: the experimental procedures and spectroscopic data in support of the reported results. See DOI: https://doi.org/10.1039/d5sc10055k.

## References

[cit1] Clayden J. (2010). Deconstructing THF. Nat. Chem..

[cit2] Mulvey R. E., Blair V. L., Clegg W., Kennedy A. R., Klett J., Russo L. (2010). Cleave and Capture Chemistry illustrated through Bimetallic-Induced Fragmentation of Tetrahydrofuran. Nat. Chem..

[cit3] Dalko P. I., Langlois Y. (1998). Stereoselective Synthesis of Quaternary Benzylic Carbons Using *C*_*2*_ Symmetric Imidazolines and Tetrahydrofuran as Electrophile. J. Org. Chem..

[cit4] Kunnari S. M., Oilunkanieme R., Laitinen R. S., Ahlgrén M. (2001). An Unexpected Tetrahydrofuran Ring Opening: Synthesis and Structural Characterization of Ph_3_PO(CH_2_)_4_TeBr_4_. J. Chem. Soc., Dalton Trans..

[cit5] Christensen S. H., Holm T., Madsen R. (2014). Ring-opening of Cyclic Ethers with Carbon–Carbon Bond Formation by Grignard Reagents. Tetrahedron.

[cit6] Chaisan N., Tan E. Y. K., Chiba S. (2024). Hydroalkylation of 1,1-Diarylalkenes Mediated by Magnesium Hydride in Ethereal Solvents. Helv. Chim. Acta.

[cit7] Fedorov A., Toutov A. A., Swisher N. A., Grubbs R. H. (2013). Lewis-Base Silane Activation: from reductive Cleavage of Aryl Ethers to Selective *ortho*-Silylation. Chem. Sci..

[cit8] Toutov A. A., Salata M., Fedorov A., Yang Y. F., Liang Y., Cariou R., Betz K. N., Couzijn E. P. A., Shabaker J. W., Houk K. N., Grubbs R. H. (2017). A Potassium *tert*-Butoxide and Hydrosilane System for ultra-Deep Desulfurization of Fuels. Nat. Energy.

[cit9] Liu W. B., Schuman D. P., Yang Y. F., Toutov A. A., Liang Y., Klare H. F. T., Nesnas N., Oestreich M., Blackmond D. G., Virgil S. C., Bannerjee S., Zare R. N., Grubbs R. H., Houk K. N., Stoltz B. M. (2017). Potassium *tert*-Butoxide-Catalyzed Dehydrogenative C–H Silylation of Heteroaromatics: A Combined Experimental and Computational Mechanistic Study. J. Am. Chem. Soc..

[cit10] Banerjee S., Yang Y. F., Jenkins I. D., Liang Y., Toutov A. A., Liu W. B., Schuman D. P., Grubbs R. H., Stoltz B. M., Krenske E. H., Houk K. N., Zare R. N. (2017). Ionic and Neutral Mechanisms for C–H Bond Silylation of Aromatic Heterocycles Catalyzed by Potassium *tert*-Butoxide. J. Am. Chem. Soc..

[cit11] Smith A. J., Young A., Rohrbach S., O’Connor E. F., Allison M., Wang H. S., Poole D. L., Tuttle T., Murphy J. A. (2017). Electron-Transfer and Hydride-Transfer Pathways in the Stoltz–Grubbs Reducing System (KO*t*Bu/Et_3_SiH). Angew. Chem., Int. Ed..

[cit12] Asgari P., Hua Y., Bokka A., Thiamsiri C., Prasitwatcharakorn W., Karedath A., Chen X., Sardar S., Yum K., Leem G., Pierce B. S., Nam K., Gao J., Jeon J. (2019). Catalytic Hydrogen Atom Transfer from Hydrosilanes to Vinylarenes for Hydrosilylation and Polymerization. Nat. Catal..

[cit13] Palumbo F., Rohrbach S., Tuttle T., Murphy J. A. (2019). N-Silylation of Amines Mediated by Et_3_SiH/KO^*t*^Bu. Helv. Chim. Acta.

[cit14] Smith A. J., Dimitrova D., Arokianathar J. N., Clark K. F., Poole D. L., Leach S. G., Murphy J. A. (2020). Et_3_SiH + KO^*t*^Bu Provide Multiple Reactive Intermediates that Compete in the Reactions and Rearrangements of Benzylnitriles and Indolenine. Chem. Sci..

[cit15] Smith A. J., Dimitrova D., Arokianathar J. N., Kolodziejczak K., Young A., Allison M., Poole D. L., Leach S. G., Parkinson J. A., Tuttle T., Murphy J. A. (2020). New Reductive Rearrangement of *N*-arylindoles Triggered by the Grubbs–Stoltz Reagent Et_3_SiH/KO^*t*^Bu. Chem. Sci..

[cit16] Arokianathar J. N., Kolodziejczak K., Bugden F. E., Clark K. F., Tuttle T., Murphy J. A. (2020). Benzylic C−H Functionalisation by [Et_3_SiH+KO^*t*^Bu] leads to Radical Rearrangements in *o-*tolyl Aryl Ethers, Amines and Sulfides. Adv. Synth. Catal..

[cit17] Kolodziejczak K., Stewart A. J., Tuttle T., Murphy J. A. (2021). Radical and Ionic Mechanisms in Rearrangements of o-Tolyl Aryl Ethers and Amines Initiated by the Grubbs–Stoltz Reagent, Et_3_SiH/KO^t^Bu. Molecules.

[cit18] Jenkins D., Krenske E. H. (2025). Mechanism of the Stoltz–Grubbs (KO^*t*^Bu/Et_3_SiH) Silylation: Single-Electron Transfer is the Missing Link between the Heterolytic and Radical Pathways. Angew. Chem., Int. Ed..

[cit19] Onge P. St., Nugraha H., Newman S. G. (2025). Hydroalkylation of Vinylarenes by TransitionMetal-Free *in situ* Generation of Benzylic Nucleophiles Using Tetramethyldisiloxane and Potassium *tert*-Butoxide. Angew. Chem., Int. Ed..

[cit20] Hokamp T., Dewanji A., Lübbesmeyer M., Mück-Lichtenfeld C., Würthwein E.-U., Studer A. (2017). Radical Hydrodehalogenation of Aryl Bromides and Chlorides with Sodium Hydride and 1,4-Dioxane. Angew. Chem., Int. Ed..

[cit21] Dastidar R. A., Kim M. S., Zhou P., Luo Z., Shi C., Barnett K. J., McClelland D. J., Chen E. Y.-X., Van Lehn R. C., Huber G. W. (2022). Catalytic production of tetrahydropyran (THP): a biomass-derived, economically competitive solvent with demonstrated use in plastic dissolution. *Green Chem*
**.**.

[cit22] Weickgenannt A., Oestreich M. (2009). Potassium *tert*-Butoxide-Catalyzed Dehydrogenative Si-O Coupling: Reactivity Pattern and Mechanism of an Underappreciated Alcohol Protection. Chem.–Asian J..

[cit23] Toutov A. A., Betz K. N., Haibach M. C., Romine A. M., Grubbs R. H. (2016). Sodium Hydroxide Catalyzed Dehydrocoupling of Alcohols with Hydrosilanes. Org. Lett..

[cit24] DeLucia A., Das N., Vannucci A. K. (2018). Mild Synthesis of Silyl Ethers *via* Potassium Carbonate Catalyzed Reactions between Alcohols and Hydrosilanes. Org. Biomol. Chem..

[cit25] Yoshida T., Ilies L., Nakamura E. (2018). Silylation of Aryl Halides with Monoorganosilanes Activated by Lithium Alkoxide. Org. Lett..

[cit26] Nocera G., Robb I., Clark K. F., McGuire T. M., Evans L., Chiba S., Murphy J. A. (2024). Reductive activation of arenes by potassium metal with potassium salts. Org. Chem. Front..

[cit27] Nocera G., Young A., Palumbo F., Emery K. J., Coulthard G., McGuire T., Tuttle T., Murphy J. A. (2018). Electron Transfer Reactions: KO*t*Bu (but not NaO*t*Bu) Photoreduces Benzophenone under Activation by Visible Light. J. Am. Chem. Soc..

[cit28] Crompton J. L., Frost J. R., Rowe S. M., Christensen K. E., Donohoe T. J. (2023). Synthesis of Cyclopropanes *via* Hydrogen-Borrowing Catalysis. Org. Lett..

[cit29] Too P. C., Chan G. H., Tnay Y. L., Hirao H., Chiba S. (2016). Hydride Reduction by a Sodium Hydride–Iodide Composite. Angew. Chem., Int. Ed..

[cit30] March’s Advanced Organic Chemistry, ed. M. B. Smith, J. Wiley & Sons Inc., 8th edn, 2020, pp. 284–288

[cit31] Di Martino A., Galli C., Gargano P., Mandolini L. (1985). Ring-closure Reactions. Part 23.’ Kinetics of Formation of Three- to Seven-membered-ring N-Tosylazacyclo-alkanes. The Role of Ring Strain in Small- and Common-sized ring Formation. J. Chem. Soc. Perkin Trans. 2.

