# Supplementary material for: Formation of cyclopentanes and cyclopropanes through alkylation of benzylic anions using ethers, thioethers and alcohols as substrates under Grubbs–Stoltz (Et3SiH/KOtBu) conditions
Source: Chem Sci. 2026 Mar 10;17(18):9081–7. doi: 10.1039/d5sc10055k (PMC12991026; doi:10.1039/d5sc10055k)
Supplement: SC-017-D5SC10055K-s001 [file SC-017-D5SC10055K-s001.pdf]

Supplementary Information (SI) for Chemical Science.
This journal is © The Royal Society of Chemistry 2026

Supporting Information

Formation of Cyclopentanes and Cyclopropanes through Alkylation of Benzylic Anions Using Ethers, Thioethers and Alcohols as Electrophiles under Grubbs-Stoltz (Et_3SiH/KOtBu) Conditions

A. J. Stewart,[‡] D. Dimitrova,[‡] S. T. M. Logan,[‡] C. Pratley, J. D. Bell, K. McGonigal, A. Lauer, S. Fenner, S. M. Nicolle, S. G. Leach and J. A. Murphy*

Specifications	S2
General Procedures	S3
Preparation of substrates	S6
Preparation of thioether, sulfoxide and sulfone substrates	S24
Cyclopentane and cyclopropane ring formations	S32
Reactions with KH	S49
Silane scope	S52
Reactions of thioethers, sulfoxides and sulfones	S53
Exploration of 4- and 6-membered ring formation	S58
NMR Spectra	S66
References	S145

+Specifications

All reagents and solvents that were obtained from commercial suppliers were used without further purification. Where used, anhydrous diethyl ether, tetrahydrofuran and dichloromethane were dried using a Pure-Solv 400 solvent purification system (Innovative Technology Inc., USA). Where stated, reactions were prepared in a glovebox supplied by Innovative Technology Inc., USA, which is operated with a nitrogen atmosphere. Thin layer chromatography was carried out using Merck silica plates, analysed with UV light (λ = 254 nm) and phosphomolybdic acid stain used for visualisation. Purification was achieved by column chromatography using ZEOprep 60 HYD 40-63 µm silica gel. IR spectra were obtained on a Shimadzu IRAffinity-1 instrument. NMR spectra were measured on a Bruker AV400 instrument. Spectra were recorded in chloroform-d_1, dichloromethane-d_2, benzene-d_6 and DMSO-d_6. The frequency was locked against the deuterated solvent signal and the final spectra were referenced against the residual non-deuterated solvent signal (for ^{1}H spectra) or the deuterated solvent signal (for ^{13}C spectra). The following abbreviations are used for peak multiplicities: s (singlet); d (doublet); t (triplet); q (quartet); quint (quintet); dd (doublet of doublets); ddd (doublet of doublets of doublets); tt (triplet of triplets); m (multiplet); br (broad). High resolution mass spectrometry (HRMS) analysis was recorded on various instruments at the University of Swansea in the EPSRC National Mass Spectrometry Centre, at the University of Strathclyde, or at the University of Glasgow. GC-MS data were recorded on an Agilent Technologies 7890A GC device connected to an Agilent Technologies 5975C inert XL EI MSD triple axis-mass detector. The GC was equipped with a Rxi-5Sil MS column (30 m x 0.25 mm x 0.25 µm). Helium was utilised as the carrier gas with flow rate of 1.0 mL/min flow rate. The injector temperature was 320 °C and was operated in splitless mode. LC-MS data were recorded on an Agilent 6130 dual source LC-MS with 1200 series LC and UV detection at 254 nm. Ionisation was performed with dual ESI and APCI source in positive and negative ionisation mode. The LC was equipped with an Agilent Poroshell 120 LC column (EC, C18, 2.7 µm, 4.6 mm x 75 mm) at 40 °C with a flow rate of 1.0 mL/min.UV-vis data were recorded on PerkinElmer Lambda 25 UV/VIS spectrophotometer.

General Procedures

General Procedure A: Reduction of carboxylic acid substrates with $LiAlH_4$

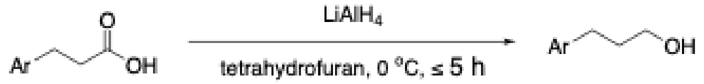

$LiAlH_4$ (X g, 4 equiv.) was added portion wise to a solution of an appropriate acid or ester (1 equiv.) in anhydrous THF at 0 °C under argon and the resulting slurry was stirred at RT until full consumption of the starting material was achieved (typically ≤ 5 h). The reaction mixture was then cooled to 0 °C and was quenched with water (X mL), 15% w/v aq. NaOH (X mL) and water (3 x X mL), where X is the mass (g) of $LiAlH_4$ used. The reaction was diluted with more THF solvent and Na_2SO_4 was added. The resulting white slurry was stirred at RT for ~15 min before it was filtered, the filtrate then concentrated to afford the respective alcohol product.

General Procedure B: Diarylmethane substitution with 4-bromobutanoic acid

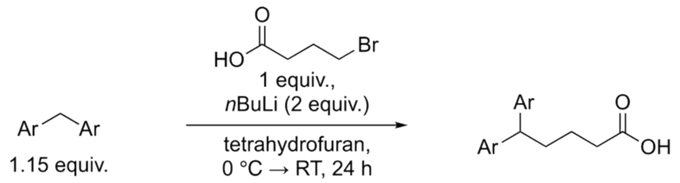

A solution of *n*BuLi (1.15 equiv.) in hexanes was added dropwise to a solution of diarylmethane (1.15 equiv.) in anhydrous THF at 0 °C under argon and the resulting solution was stirred for 15 min. In a separate flask, a solution of *n*BuLi (2.3 M, 1 equiv.) in hexanes was added dropwise to a solution of 4-bromobutanoic acid (1 equiv.) in THF at 0 °C under argon and the resulting slurry was stirred at RT for ~ 15 min. The slurry containing the carboxylate salt was transferred by cannula to the solution containing the deprotonated diarylmethane followed by THF and the resulting mixture was stirred at RT for 24 h. The reaction mixture was subsequently quenched with 6 M HCl at 0 °C until the pH was 1-2. The aqueous layer was separated and was further washed with Et_2O (2 x). The combined organic phases were concentrated, redissolved in Et_2O and water and basified with solid NaOH until the pH was 13-14. The aqueous phase was separated and was further washed with Et_2O (2 x). The aqueous phase was then acidified with 6 M HCl and was extracted with Et_2O (3 x). The ether phases obtained after the last set of extractions were dried over Na_2SO_4, filtered and concentrated *in vacuo* affording crude acid product that was used in the next step without further purification.

General Procedure C: Wittig reaction with methyl (triphenylphosphoranylidene)acetate

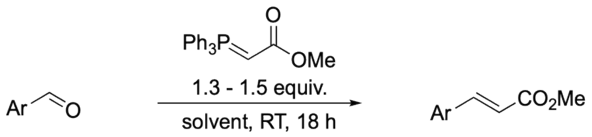

The general procedure follows an adapted procedure from the literature.[1] To an oven-dried flask equipped with a stir bar was added methyl (triphenylphosphoranylidene)acetate (1.3 -

1.5 equiv.), the flask flushed with argon, and anhydrous DCM added. The resulting slurry was stirred at RT as the aldehyde (1 equiv.) was added portion-wise. The reaction mixture was stirred for 18 h, after which it was cooled to RT, extracted with 1M HCl (2 x 20 mL) and washed with EtOAc (3 x 15 mL). The combined organics were basified with 1M NaOH and further extracted with EtOAc (3 x 15 mL). The combined organics were concentrated *in vacuo* affording crude alkene product.

General Procedure D: Alkene or alkyne reduction with H_2 over Pd/C

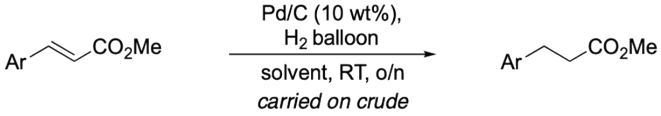

The general procedure follows an adapted procedure from the literature.[1] After general procedure C, the crude alkene or alkyne (1 equiv.) and 10 wt% Pd/C were suspended in an appropriate solvent in an oven-dried flask under an atmosphere of argon. The flask was evacuated and backfilled with H_2 from a balloon three times, and the black suspension stirred at RT overnight under an atmosphere of H_2. The reaction was filtered through Celite, washing with copious amounts of EtOAc. The filtrate was concentrated *in vacuo* affording the corresponding ester product. The crude product was carried onto the next step without further purification.

General Procedure E: Mesylation with mesyl chloride

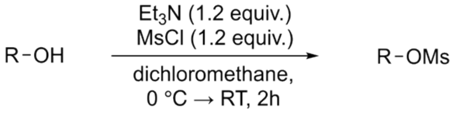

The general procedure follows an adapted procedure from the literature.[2] Et_3N (1.2 equiv.) and MsCl (1.2 equiv.) were added dropwise to a solution of alcohol (1 equiv.) in anhydrous DCM at 0 ^{o}C under an atmosphere of argon. The reaction was left to stir until complete consumption of alcohol (typically 2 hours). The organic layer was washed three times with sat. $NaHCO_3$, then dried over Na_2SO_4, filtered and concentrated to afford the desired mesylate.

General Procedure F: Substitution of mesylate by thiolate

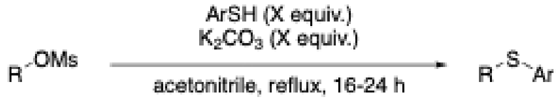

K_2CO_3 (1.2 or 2 equiv.) and alkyl mesylate (1 equiv.) were added to a solution of the appropriate thiol (1.2 or 2 equiv.) in degassed MeCN. The resulting solution was stirred at reflux for a period of 16-24 hours before being cooled to RT. The reaction mixture was diluted with water and extracted with EtOAc. The combined organics were then dried over Na_2SO_4, filtered and concentrated. The crude mixture was then purified by chromatography to afford the desired sulfide.

General Procedure G: Oxidation with *m*-CPBA to sulfoxide product, or to sulfone product

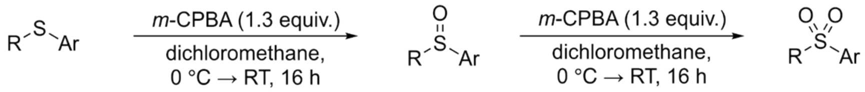

70% *m*-CPBA (1.3 equiv.) was added portion-wise to a solution of sulfide (1 equiv.) in DCM at 0 °C. The reaction was stirred at RT for 16 h, after which it was washed with saturated Na_2CO_3 solution (50 mL). The organic layer was dried over Na_2SO_4, filtered, and concentrated. The crude mixture was purified by chromatography to afford desired sulfoxide product.[3]
The procedure was repeated to afford the sulfone product, where necessary.

General Procedure H: Alkylation of alcohols

To a solution of alcohol (1 equiv.) in anhydrous THF was added NaH (1.5 equiv.) portionwise and the mixture was allowed to stir for 15 min before dropwise addition of alkyl iodide (1.2 equiv.). The reaction was stirred for 16 h, after which it was quenched with water and extracted into Et_2O (3x). The combined organic phases were dried over $MgSO_4$, filtered and concentrated affording the crude material which was purified via column chromatography where necessary.

General Procedure I: Reaction of substrate with KO*t*Bu-Et_3SiH

In a glovebox, substrate (0.5 mmol, 1 equiv.), KO*t*Bu (168 or 224 mg, 1.5 or 2 mmol, 3 or 4 equiv.), and where stated, Et_3SiH (240 µL, 1.5 mmol, 3 equiv.) were dissolved in anhydrous THF (5 mL) or 1,4-dioxane and sealed in a Synthware™ storage tube with a high vacuum valve. The tube was refluxed at 130 °C for 18 h before being cooled to room temperature, opened to air, and diluted with water (50 mL). The organic products were extracted into Et_2O (3 x 50 mL). The combined organic phases were dried over Na_2SO_4, filtered and concentrated.

General Procedure J: Reaction of substrate with KH-Et_3SiH

In a glovebox, substrate (0.5 mmol, 1 equiv.), KH (60 mg or 80 mg, 1.5 mmol or 2 mmol, 3 equiv. or 4 equiv.) and where stated, Et_3SiH (240 µL, 1.5 mmol, 3 equiv.), were dissolved in anhydrous THF (5 mL) and sealed in a Synthware™ storage tube with high vacuum valve. The tube was refluxed at 130 °C for 18 h before being cooled to room temperature, opened to air, and diluted with water (50 mL). The organic products were extracted into Et_2O (3 x 50 mL). The combined organic phases were dried over Na_2SO_4, filtered and concentrated.

Preparation of Substrates

N,2-dimethyl-*N*-phenylaniline 11

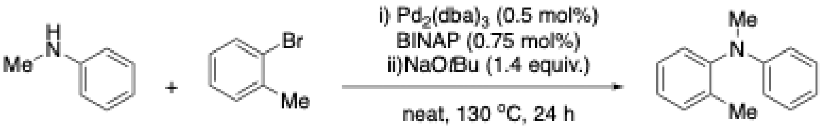

S1 **S2** **11** 37%

This substrate was prepared according to a literature procedure.[4] NaO*t*Bu (2.11 g, 22 mmol, 1.4 equiv.), $Pd_2(dba)_3$ (73 mg, 0.5 mol %), BINAP (74 mg, 0.75 mol %), *N*-methylaniline **S1** (2 mL, 19 mmol, 1.2 equiv.), and 2-bromotoluene **S2** (1.93 mL, 16 mmol, 1 equiv.) were added to an oven-dried pressure tube equipped with a stirrer bar. The liquid substrates were added last and the vial flushed with argon. The mixture was refluxed at 130 °C for 24 h. The mixture was allowed to cool to room temperature, taken up in ether (50 mL), filtered, and concentrated. The crude product was then purified by column chromatography (100% hexane→3% EtOAc in hexane) to afford *N*,2-dimethyl-*N*-phenylaniline **11** as a colourless oil (1.16 g, 37%). ν_{max} (neat)/cm^{-1} 3022, 2877, 1593, 1490, 1338, 1251, 1112, 746, 727, 691; **^{1}H NMR** (400 MHz, $CDCl_3$) δ 7.31 – 7.27 (m, 1H), 7.25 – 7.12 (m, 5H), 6.71 (tt, *J* = 7.3 Hz, 1.0 Hz, 1H), 6.52 (dd, *J* = 8.8, 1.0 Hz, 2H), 3.22 (s, 3H), 2.15 (s, 3H); **^{13}C NMR** (101 MHz, $CDCl_3$) δ 148.7, 146.3, 136.3, 130.9, 128.5, 127.8, 127.0, 125.9, 116.3, 112.3, 38.5, 17.3; ***m/z*** (EI): 197.3 (100, M+), 183.2 (89), 165.2 (62), 155.2 (66), 107.2 (63), 91.2 (97), 77.2 (93), 65.2 (96), 51.2 (82). Analytical data are in agreement with the data reported in the literature.[5]

Preparation of 10-Methyl-9,10-dihydroacridine 13

Step 1: 10-Methylacridin-10-ium iodide S4

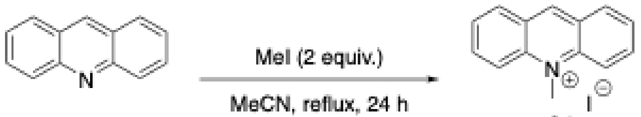

S3 **S4** 83%

Prepared according to a literature procedure.[6] To a solution of acridine **S3** (2.688 g, 15 mmol, 1 equiv.) in anhydrous MeCN (20 mL) under argon was added MeI (1.9 mL, 30 mmol, 2 equiv.) and the reaction mixture was refluxed for 24 h. The reaction mixture was cooled to RT, concentrated, and the resulting red solid triturated with Et_2O (4 x) affording 10-methylacridin-10-ium iodide **S4** (4.02 g, 83%) as a red solid. **Mp** 227 – 229 °C (lit. 230 – 231 °C[7]); **^{1}H NMR** (400 MHz, d_6-DMSO) δ 10.23 (s, 1H), 8.82 (dd, *J* = 9.2, 0.5 Hz, 2H), 8.66 (dd, *J* = 8.4, 1.3 Hz, 2H), 8.52 – 8.46 (m, 2H), 8.10 – 8.03 (m, 2H), 4.88 (s, 3H). **^{13}C NMR** (101 MHz, d_6-DMSO) δ 150.6, 141.4, 139.0, 131.7, 127.8, 126.3, 119.0, 38.7; ***m/z*** (ESI+) 194.2 $[M]^+$. Analytical data are consistent with the data previously reported in the literature.[6]

Step 2: 10-Methyl-9,10-dihydroacridine 13

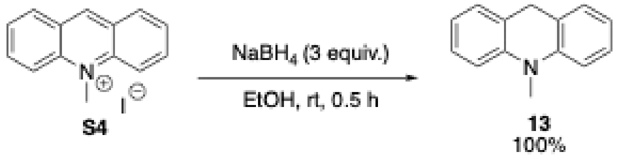

The substrate was prepared according to a literature procedure.[6] $NaBH_4$ (715 mg, 19 mmol, 3 equiv.) was added to a stirring solution of 10-methylacridin-10-ium iodide **S4** (2.02 g, 6.3 mmol, 1 equiv.) in anhydrous EtOH (60 mL) under argon and the reaction mixture stirred at RT for ~0.5 h until the red colour disappeared. The reaction volume was reduced to ~20 mL, resulting in a white precipitate, followed by addition of water (20 mL) causing further precipitation. The resulting white slurry was filtered and further washed with 1:1 H_2O/EtOH affording 10-methyl-9,10-dihydroacridine **13** (1.23 g, 100%) as a white solid. **Mp** 95 – 96 °C (lit. 95 – 96 °C[8]); $\mathbf{v_{max}}$ (neat)/cm^{-1} 2922, 1635, 1595, 1494, 1460, 1367, 1178, 752; **^{1}H NMR** (400 MHz, $CDCl_3$) δ 7.22 – 7.15 (m, 4H), 6.93 (td, *J* = 7.4, 1.1 Hz, 2H), 6.88 (d, *J* = 7.9 Hz, 2H), 3.90 (s, 2H), 3.38 (s, 3H); **^{13}C NMR** (101 MHz, $CDCl_3$) δ 143.8, 127.7, 127.0, 124.5, 120.7, 112.0, 33.4, 33.2; ***m/z*** (EI): 194 (100, [M-H]+), 176 (42), 152 (14), 126 (4), 97 (9), 63 (10). Analytical data are consistent with the data previously reported in the literature.[6]

Preparation of 5,5-diphenylpentanol 25

Step 1: 5,5-Diphenylpentanoic acid S6

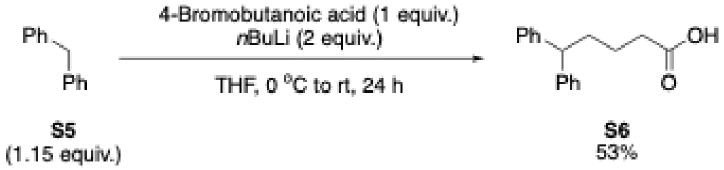

This experiment was carried out according to general procedure B using *n*-BuLi (26 mL, 2.3 M, 1.15 equiv.), diphenylmethane **S5** (9.7 g, 57.5 mmol, 1.15 equiv.) and anhydrous THF (20 mL). In a separate flask, *n*BuLi (22 mL, 2.3 M 1 equiv.), 4-bromobutanoic acid (8.35 g, 50 mmol, 1 equiv.), were added to THF (20 mL). The reaction afforded crude 5,5-diphenylpentanoic acid product **S6** (6.62 g, 53%) as a white solid that was used in the next step without further purification. **Mp** 74 – 76 °C (lit. 84 – 85 °C[9]); $\mathbf{v_{max}}$ (neat)/cm^{-1} 3024, 2930, 2868, 1701, 1599, 1283, 1153, 1088, 937; **^{1}H NMR** (400 MHz, $CDCl_3$) δ 7.31 – 7.21 (m, 8H), 7.20 – 7.14 (m, 2H), 3.91 (t, *J* = 7.7 Hz, 1H), 2.37 (t, *J* = 7.5 Hz, 2H), 2.14 – 2.00 (m, 2H), 1.67 – 1.57 (m, 2H); **^{13}C NMR** (101 MHz, $CDCl_3$) δ 177.8, 144.8, 128.6, 127.9, 126.4, 51.3, 35.1, 33.7, 23.4; ***m/z*** (APCI): 253.1 ([M-H]-). Analytical data are consistent with those previously reported in the literature.[7]

Step 2: 5,5-Diphenylpentanol 25

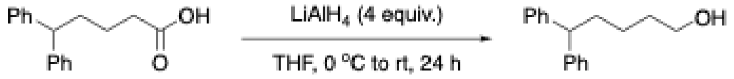

S6 25 96%

The reaction was carried out according to general procedure A with 5,5-diphenylpentanoic acid **S6** (6.62 g, 26.1 mmol, 1 equiv.), $LiAlH_4$ (3.96 g, 104 mmol, 4 equiv.) and THF (100 mL). The reaction mixture was carefully quenched with water (4 mL), 15% aq. NaOH (4 mL) and water (12 mL). The crude 5,5-diphenylpentan-1-ol **25** (6.02 g, 96%) was isolated as a pale-yellow oil and was used in the next step without further purification. $\mathbf{v_{max}}$ (neat)/cm^{-1} 3350, 3082, 3024, 2930, 1599, 1493, 1377, 1155, 1059, 1030, 986, 915; **^{1}H NMR** (400 MHz, $CDCl_3$) δ 7.31 – 7.22 (m, 8H), 7.20 – 7.14 (m, 2H), 3.90 (t, *J* = 7.8 Hz, 1H, CH), 3.61 (t, *J* = 6.7 Hz, 2H), 2.08 (q, *J* = 7.7 Hz, 2H), 1.65 – 1.57 (m, 2H), 1.42 – 1.26 (m, 2H, CH_2); **^{13}C NMR** (101 MHz, $CDCl_3$) δ 145.3, 128.7, 128.0, 126.2, 63.0, 51.6, 35.7, 33.0, 24.5; ***m/z*** (EI): 240.3 (M^+, 1%), 222.3 (4), 193.1 (1), 178.3 (2), 167.2 (100), 152.2 (18), 139.2 (1), 128.2 (3), 115.1 (5), 103.3 (1), 91.2 (8), 77.2 (6), 65.1 (1), 51.2 (4). Analytical data are consistent with the literature.[8]

((5,5-Diphenylpentyl)oxy)triethylsilane 26

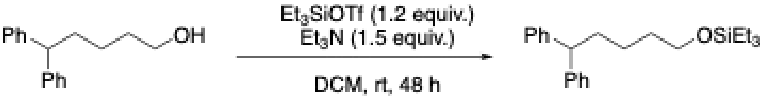

25 26 60%

The reaction was carried out according to a modified literature procedure.[9] Et_3SiOTf (1.36 mL, 6 mmol, 1.2 equiv.) was added to a stirred solution of 5,5-diphenylpentan-1-ol **25** (1.20 g, 5 mmol, 1 equiv.) and Et_3N (1.05 mL, 7.5 mmol, 1.5 equiv.) in anhydrous DCM (12.5 mL) at 0 °C under an atmosphere of argon. The resulting mixture was stirred for 48 h before it was diluted with water (20 mL) at 0 °C and extracted with DCM (50 mL + 3 x 30 mL). The combined organics were dried over $MgSO_4$, filtered and concentrated. Purification by chromatography (100% hexanes ♑4% EtOAc in hexanes ♑100% EtOAc) afforded (5,5-diphenylpentyl)oxy)triethylsilane **26** (1.064 g, 60%) as a colourless oil. $\mathbf{v_{max}}$ (neat)/cm^{-1} 3084, 3061, 3026, 2951, 2934, 2911, 2874, 1599, 1584, 1493, 1450, 1414, 1381, 1302, 1238, 1180, 1153, 1094, 1065, 1032, 1003, 976, 945, 907, 874, 800, 768, 739, 727, 696, 669, 633, 619, 602; **^{1}H NMR** (400 MHz, $CDCl_3$) δ 7.31 – 7.20 (m, 8H), 7.20 – 7.12 (m, 2H), 3.89 (t, *J* = 7.8 Hz, 1H), 3.55 (t, *J* = 6.7 Hz, 2H), 2.06 (q, *J* = 7.8 Hz, 2H), 1.62 – 1.51 (m, 2H), 1.40 – 1.18 (m, 2H), 0.93 (t, *J* = 7.9 Hz, 9H, 3 x Me), 0.56 (q, *J* = 7.9 Hz, 6H, 3 x $SiCH_2$); **^{13}C NMR** (101 MHz, $CDCl_3$) δ 145.3, 128.5, 128.0, 126.2, 62.9, 51.5, 35.7, 33.0, 24.5, 6.9, 4.6; ***m/z*** (EI) calcd. for $C_{23}H_{34}OSi^+$ (M^+): 354.2379, found: 354.2386.

5-Methoxypentane-1,1-diyl)dibenzene 27

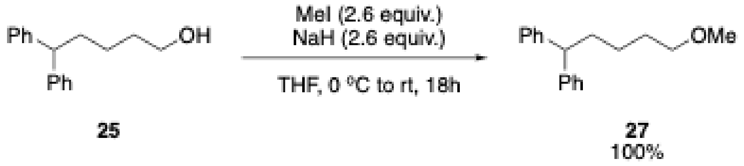

This experiment was carried out according to general procedure H. A solution of 5,5-diphenylpentan-1-ol **25** (1.202 g, 5 mmol, 1 equiv.) in anhydrous THF (10 mL) was added dropwise to a slurry of NaH (312 mg, 13 mmol, 2.6 equiv.) in anhydrous THF (10 mL) at 0 °C under an atmosphere of argon. The reaction mixture was stirred at RT for ~ 20 min, resulting in a beige slurry. MeI (813 µL, 13 mmol, 2.6 equiv.) was added at RT and the resulting mixture was stirred at RT overnight before it was quenched with water (20 mL) and extracted with Et_2O (4 x 50 mL). The combined organic phases were dried over $MgSO_4$, filtered and concentrated affording (5-methoxypentane-1,1-diyl)dibenzene **27** (1.270 g, 100%) as a yellow oil that was used without any further purification. **ν_{max}** (neat)/cm^{-1} 3082, 3059, 3024, 2930, 2860, 2826, 2806, 1599, 1493, 1450, 1387, 1352, 1302, 1263, 1244, 1192, 1171, 1117, 1030, 1003, 964, 914, 874, 845, 831, 779, 745, 696, 631, 602; **^{1}H NMR** (400 MHz, $CDCl_3$) δ 7.33 – 7.20 (m, 8H), 7.20 – 7.11 (m, 2H), 3.89 (t, *J* = 7.8 Hz, 1H), 3.33 (t, *J* = 6.7 Hz, 2H), 3.29 (s, 3H), 2.12 – 2.01 (m, 2H), 1.67 – 1.56 (m, 2H), 1.40 – 1.27 (m, 2H); **^{13}C NMR** (101 MHz, $CDCl_3$) δ 145.3, 128.6, 128.0, 126.2, 72.9, 58.7, 51.5, 35.7, 29.8, 24.8; ***m/z*** (ESI+) calcd. for $C_{18}H_{23}O^+$ $[M+H]^+$ 255.1743, found: 255.1738.

Synthesis of 4-(9*H*-Xanthen-9-yl)butan-1-ol 30

Step 1: 4-(9*H*-Xanthen-9-yl)butan-1-ol S8

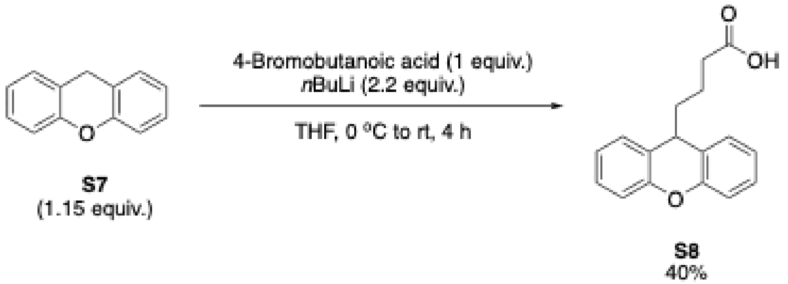

This experiment was carried out according to general procedure B using ^{n}BuLi in hexanes (9.6 mL, 1.6 M, 15.4 mmol, 2.2 equiv.), 9*H*-xanthene **S7** (1.46 g, 8 mmol, 1.15 equiv.), and anhydrous THF (35 mL) under argon at 0 °C. In a separate flask, 4-bromobutanoic acid (1.17 g, 6.99 mmol, 1 equiv.) in anhydrous THF (15 mL). The reaction afforded the crude 4-(9*H*-xanthen-9-yl)butanoic acid product **S8** (0.756 g, 40%) as a viscous yellow oil that was used in the next step. **ν_{max}** (neat)/cm^{-1} 3067, 3038, 2932, 2870, 1707, 1655, 1645, 1609, 1574, 1479, 1458, 1412, 1346, 1331, 1308, 1252, 1240, 1215, 1182, 1148, 1101, 1047, 1032, 934, 872, 843, 802, 754, 702, 671, 656, 629, 613; **^{1}H NMR** (400 MHz, $CDCl_3$) δ 7.25 – 7.16 (m, 4H), 7.12 – 7.03 (m, 4H), 4.02 (t, *J* = 6.0 Hz, 1H), 2.21 (t, *J* = 7.5 Hz, 2H), 1.85 – 1.64 (m, 2H), 1.65 – 1.43 (m, 2H); **^{13}C NMR** (101 MHz, $CDCl_3$) δ 178.5, 152.3, 128.7, 127.8, 125.1, 123.4, 116.6, 40.0, 38.9, 33.8, 20.9; ***m/z*** (ESI+) calcd. for $C_{17}H_{15}O_3^-$ $[M-H]^-$ 267.1027, found: 267.1030.

Step 2: 4-(9H-Xanthen-9-yl)butan-1-ol 30

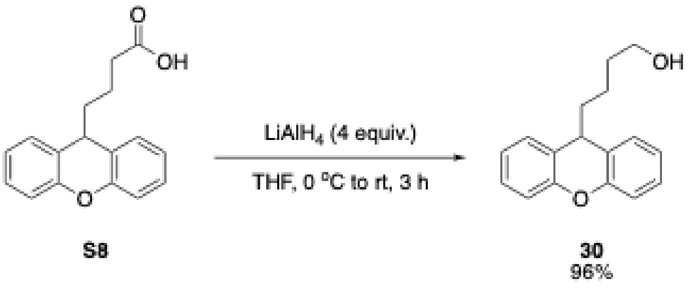

This reaction was carried out according to general procedure A using 4-(9*H*-xanthen-9-yl)butanoic acid **S8** (0.746 g, 2.8 mmol, 1 equiv.,), $LiAlH_4$ (0.426g, 11.2 mmol, 4 equiv.) and anhydrous THF (20 mL). The reaction mixture was carefully quenched with water (0.5 mL), 2 M NaOH (0.85 mL) and water (0.85 mL). The crude 4-(9*H*-xanthen-9-yl)butan-1-ol **30** (0.680 g, 96%) was isolated as a viscous pale-yellow oil. $\mathbf{v_{max}}$ (neat)/cm^{-1} 3339, 3194, 3071, 3040, 2934, 2862, 1647, 1603, 1574, 1477, 1449, 1346, 1325, 1308, 1248, 1213, 1186, 1152, 1130, 1119, 1098, 1069, 1060, 1034, 935, 891, 860, 820, 750, 704, 671, 656, 642, 619, 604; ^{1}**H NMR** (400 MHz, $CDCl_3$) δ 7.25 – 7.16 (m, 4H), 7.11 – 7.03 (m, 4H), 4.00 (t, *J* = 6.0 Hz, 1H), 3.53 (t, *J* = 6.6 Hz, 2H), 1.80 – 1.70 (m, 2H), 1.50 – 1.39 (m, 2H), 1.29 – 1.19 (m, 2H); ^{13}**C NMR** (101 MHz, $CDCl_3$) δ 152.4, 128.7, 127.7, 125.6, 123.3, 116.5, 62.9, 40.7, 39.2, 32.8, 21.8; ***m/z*** (ESI+) calcd. for $C_{17}H_{17}O_2^+$ $[M-H]^+$ 253.1223, found: 253.1216.

Synthesis of 4-(10-Methyl-9,10-dihydroacridin-9-yl)butan-1-ol 29

4-(10-Methyl-9,10-dihydroacridin-9-yl)butanoic acid S9

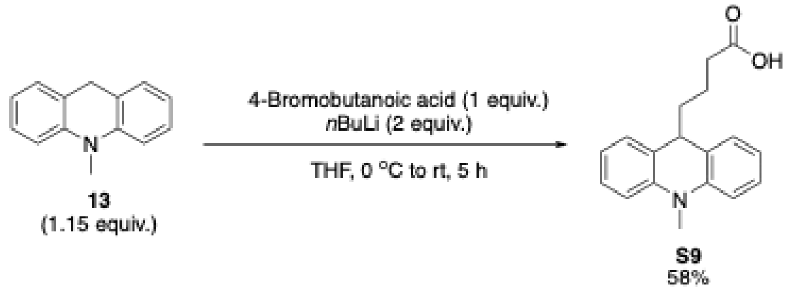

This reaction was carried out according to general procedure B using 10-methyl-9,10-dihydroacridine **13** (2.23 g, 11.4 mmol, 1.15 equiv.), ^{n}BuLi (2 M, 5 mL + 6 mL, 21.8 mmol, 2.2 equiv.), 4-bromobutanoic acid (1.65 g, 9.9 mmol, 1 equiv.) and anhydrous THF (65 mL). The crude 4-(10-methyl-9,10-dihydroacridin-9-yl)butanoic acid product **S9** (1.64 g, 58%) was isolated as a brown oil that was used directly in the next step. $\mathbf{v_{max}}$ (neat)/cm^{-1} 3049, 3022, 2932, 2909, 2874, 2820, 1701, 1607, 1591, 1551, 1503, 1472, 1454, 1420, 1377, 1341, 1317, 1269, 1234, 1192, 1165, 1128, 1099, 1065, 1042, 989, 932, 908, 872, 837, 748, 737, 702, 646, 627, 613; ^{1}**H NMR** (400 MHz, $CDCl_3$) δ 7.25 – 7.19 (m, 2H), 7.15 (dd, *J* = 7.4, 1.5 Hz, 2H), 6.98 – 6.88 (m, 4H), 3.89 – 3.80 (m, 1H), 3.39 (s, 3H), 2.26 – 2.20 (m, 2H), 1.61 – 1.53 (m, 4H); ^{13}**C NMR** (101 MHz, $CDCl_3$) δ 179.0, 142.5, 128.3, 127.6, 127.1, 120.7, 112.3, 44.2, 37.0, 33.9, 33.1, 22.1; ***m/z*** (ESI+) calcd. for $C_{18}H_{18}O_2N^-$ $[M-H]^-$ 280.1343, found: 280.1346.

Step 2: 4-(10-Methyl-9,10-dihydroacridin-9-yl)butan-1-ol 29

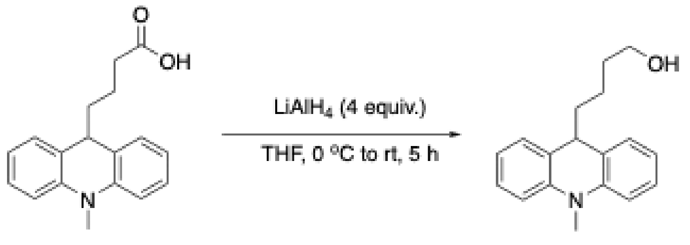

S9 29 85%

Carried out according to general procedure A using 4-(10-methyl-9,10-dihydroacridin-9-yl)butanoic acid **S9** (1.64 g, 5.8 mmol, 1 equiv.), $LiAlH_4$ (0.88 g, 23.2 mmol, 4 equiv.) and anhydrous THF (40 mL). The reaction mixture was carefully quenched with water (1 mL), 2 M NaOH (2 mL) and water (2 mL). The crude 4-(10-methyl-9,10-dihydroacridin-9-yl)butan-1-ol **29** (1.380 g, 85%) was isolated as a viscous brown oil and was used in the next step without any further purification. $\mathbf{v_{max}}$ (neat)/cm^{-1} 3347, 3055, 3034, 2930, 2884, 2870, 2859, 2820, 1591, 1472, 1454, 1379, 1342, 1315, 1267, 1211, 1165, 1142, 1128, 1103, 1065, 1042, 999, 941, 928, 883, 849, 745, 700, 675, 646, 615; **^{1}H NMR** (400 MHz, $CDCl_3$) δ 7.25 – 7.18 (m, 2H), 7.14 (dd, *J* = 7.3, 1.5 Hz, 2H), 6.99 – 6.89 (m, 4H), 3.83 (t, *J* = 7.2 Hz, 1H), 3.56 (t, *J* = 6.6 Hz, 2H), 3.39 (s, 3H), 1.61 – 1.52 (m, 2H), 1.52 – 1.42 (m, 2H), 1.36 – 1.23 (m, 2H); **^{13}C NMR** (101 MHz, $CDCl_3$) δ 142.5, 128.3, 128.0, 127.0, 120.6, 112.2, 63.0, 44.4, 37.6, 33.1, 32.8, 23.1; ***m/z*** (ESI+) calcd. for $C_{18}H_{22}NO^+$ $[M+H]^+$ 268.1696, found: 268.1687.

Preparation of substrates for intramolecular cyclopropanations

3,3-Diphenylpropan-1-ol 36

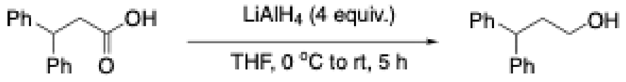

S10 36 100%

This experiment was carried out according to general procedure A using 3,3-diphenylpropanoic acid **S10** (1 g, 4.4 mmol, 1 equiv.), $LiAlH_4$ (685 mg, 17.7 mmol, 4 equiv.) and THF (25 mL). The reaction mixture was carefully quenched with water (0.7 mL), 15% aq. NaOH (0.7 mL) and water (3 mL). The crude 3,3-diphenylpropan-1-ol **36** (944 mg, 100%) was isolated as a colourless oil and was sufficiently pure. $\mathbf{v_{max}}$ (neat)/cm^{-1} 3375, 3080, 3061, 3024, 2931, 2860, 1599, 1492, 1265; **^{1}H NMR** (400 MHz, $CDCl_3$) δ 7.32 – 7.24 (m, 8H), 7.22 – 7.15 (m, 2H), 4.14 (t, *J* = 7.8 Hz, 1H), 3.62 (t, *J* = 5.8 Hz, 2H), 2.36 – 2.29 (m, 2H); **^{13}C NMR** (101 MHz, $CDCl_3$) δ 144.6, 128.6, 127.9, 126.4, 61.2, 47.4, 38.3; ***m/z* (EI):** 212.1 (M+, 8%), 194.1 (45), 179.1 (10), 167.1 (100), 152.1 (29), 139.1 (3), 128.1 (3), 116.1 (15), 103.1 (12), 91.1 (8), 77.1 (20), 63.1 (7), 51.1 (15). Analytical data are consistent with those previously reported in the literature.[10]

3-Phenyl-3-(pyridin-2-yl)propan-1-ol S12

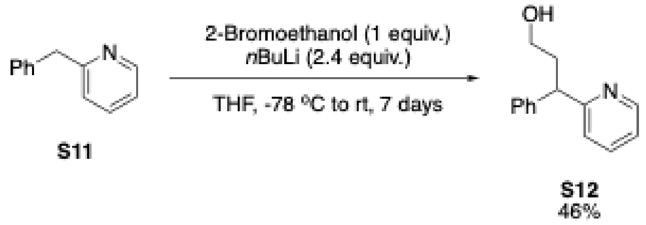

^{n}BuLi in hexanes (2.3 M, 5.2 mL, 12 mmol, 1.2 equiv.) was added dropwise to a stirred solution of 2-benzylpyridine **S11** (1.61 mL, 10 mmol, 1 equiv.) in anhydrous THF (30 mL) at -78 °C under an atmosphere of argon, resulting in a deep red solution. ^{n}BuLi in hexanes (2.3 M, 5.2 mL, 12 mmol, 1.2 equiv.) was added dropwise to a stirred solution of 2-bromoethanol (0.71 mL, 10 mmol, 1 equiv.) in anhydrous THF (10 mL) at -78 °C under an atmosphere of argon and the reaction was stirred at -78 °C for ~15 min before it was added to the solution of deprotonated 2-benzylpyridine at -78 °C. The reaction was warmed up to RT and was stirred for 7 days before it was quenched with water (10 mL) at 0 °C. The reaction mixture was concentrated, diluted with water (30 mL), and extracted with DCM (3 x 30 mL) and EtOAc (3 x 30 mL). The combined organic phases were dried over $MgSO_4$, filtered and concentrated. Purification by chromatography (100% DCM → 2% MeOH in DCM) afforded 3-phenyl-3-(pyridin-2-yl)propan-1-ol **S12** (972 mg, 46%) as a brown oil. $\mathbf{v_{max}}$ (neat)/cm^{-1} 3300, 3082, 3059, 3024, 3005, 2928, 2872, 1589, 1568, 1493, 1472, 1452, 1431, 1352, 1342, 1335, 1290, 1227, 1202, 1171, 1150, 1099, 1047, 1030, 993, 966, 914, 899, 880, 853, 833, 787, 746, 698, 652, 619; **^{1}H NMR** (400 MHz, $CDCl_3$) δ 8.55 (ddd, *J* = 4.9, 1.7, 0.8 Hz, 1H), 7.56 (td, *J* = 7.7, 1.9 Hz, 1H), 7.34 – 7.26 (m, 4H), 7.24 – 7.18 (m, 1H), 7.15 – 7.06 (m, 2H), 4.38 (dd, *J* = 8.4, 5.8 Hz, 1H), 3.73 – 3.56 (m, 2H), 2.53 – 2.41 (m, 1H, diastereotopic CH), 2.40 – 2.28 (m, 1H, diastereotopic CH); **^{13}C NMR** (101 MHz, $CDCl_3$) δ 163.5, 148.7, 143.5, 136.9, 128.7, 128.4, 126.7, 123.6, 121.6, 60.8, 50.9, 37.7; ***m/z*** (ESI+) calcd. for $C_{14}H_{16}NO^+$ $[M+H]^+$ 214.1226, found: 214.1220. Analytical data are consistent with those previously reported in the literature.[11]

3-Phenyl-3-(pyridin-4-yl)propan-1-ol S14

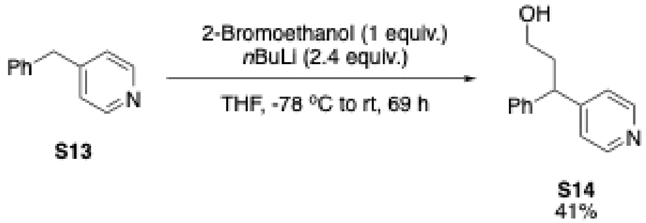

^{n}BuLi in hexanes (2.3 M, 15.6 mL, 36 mmol, 1.2 equiv.) was added dropwise to a stirred solution of 4-benzylpyridine **S13** (4.79 mL, 30 mmol, 1 equiv.) in anhydrous THF (90 mL) at 0 °C under an atmosphere of argon, resulting in a deep red solution. ^{n}BuLi in hexanes (2.3 M, 15.6 mL, 36 mmol, 1.2 equiv.) was added dropwise to a stirred solution of 2-bromoethanol (2.13 mL, 30 mmol,1 equiv.) in anhydrous THF (30 mL) at 0 °C under an atmosphere of argon and the reaction was stirred at 0 °C for ~15 min before it was added to the deep red solution of deprotonated 4-benzylpyridine at 0 °C. The reaction was warmed up to RT and was stirred at RT for 69 h before it was quenched with water (10 mL), concentrated, diluted with water (40

mL) and extracted with EtOAc (80 mL + 3 x 50 mL). The combined organic phases were dried over $MgSO_4$, filtered and concentrated. Purification by column chromatography (100% DCM → 2% MeOH in DCM) afforded 3-phenyl-3-(pyridin-4-yl)propan-1-ol **S14** (2.589 g, 41%) as a brown oil. **v_{max}** (neat)/cm^{-1} 3213, 3059, 3026, 2932, 2870, 1595, 1557, 1493, 1452, 1416, 1375, 1341, 1306, 1275, 1219, 1171, 1157, 1069, 1051, 1030, 1003, 968, 922, 881, 856, 814, 768, 745, 698, 664, 631, 617; **^{1}H NMR** (400 MHz, $CDCl_3$) δ 8.49 (dd, *J* = 4.5, 1.6 Hz, 2H), 7.36 – 7.28 (m, 2H), 7.25 – 7.20 (m, 3H), 7.17 (dd, *J* = 4.7, 1.5 Hz, 2H), 4.16 (t, *J* = 7.9 Hz, 1H), 3.61 (t, *J* = 6.3 Hz, 2H), 2.31 (dt, *J* = 7.9, 6.3 Hz, 2H); **^{13}C NMR** (101 MHz, $CDCl_3$) δ 153.7, 150.0, 142.7, 128.9, 128.1, 127.1, 123.4, 60.5, 46.7, 37.5; ***m/z* (EI):** 213.1 (M^+, 3%), 195.1 (88), 180.1 (27), 167.1 (100), 152.1 (8), 139.1 (21), 128.1 (5), 115.1 (28), 104.1 (8), 91.1 (9), 77.1 (11), 63.1 (9), 51.1 (21). Analytical data are consistent with those previously reported in the literature.[12]

2-(1-Phenyl-3-((triethylsilyl)oxy)propyl)pyridine S15

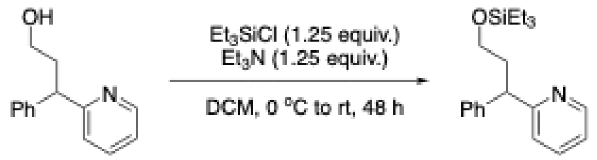

S12

S15
94%

Et_3SiCl (0.7 mL, 4.6 mmol, 1.25 equiv.) was added to a stirred solution of 3-phenyl-3-(pyridin-2-yl)propan-1-ol **S12** (0.8 g, 3.7 mmol, 1 equiv.) and Et_3N (0.76 mL, 5.5 mmol 1.5 equiv.) in anhydrous DCM (9 mL) at 0 °C under an atmosphere of argon. The resulting mixture was stirred for 48 h before it was diluted with water (20 mL) at 0 °C and extracted with DCM (50 mL + 2 x 30 mL). The combined organic phases were dried over $MgSO_4$, filtered and concentrated. Purification by column chromatography (hexane → 10% EtOAc in hexanes) afforded 2-(1-phenyl-3-((triethylsilyl)oxy)propyl)pyridine **S15** (1.13 g, 94%) as an orange oil. **v_{max}** (neat)/cm^{-1} 3082, 3061, 3026, 3005, 2953, 2934, 2911, 2874, 1588, 1568, 1493, 1470, 1458, 1431, 1414, 1381, 1236, 1148, 1094, 1053, 1005, 993, 974, 941, 899, 862, 799, 772, 743, 727, 669, 619; **^{1}H NMR** (400 MHz, $CDCl_3$) δ 8.56 (ddd, *J* = 4.9, 1.8, 0.9 Hz, 1H), 7.55 (td, *J* = 7.7, 1.9 Hz, 1H), 7.38 – 7.31 (m, 2H), 7.31 – 7.24 (m, 2H), 7.25 – 7.13 (m, 2H), 7.07 (ddd, *J* = 7.5, 4.9, 1.1 Hz, 1H), 4.28 (t, *J* = 7.7 Hz, 1H), 3.66 – 3.46 (m, 2H), 2.60 – 2.41 (m, 1H, diastereotopic CH), 2.41 – 2.20 (m, 1H, diastereotopic CH), 0.91 (t, *J* = 7.9 Hz, 9H), 0.53 (q, *J* = 7.9 Hz, 6H); **^{13}C NMR** (101 MHz, $CDCl_3$) δ 163.7, 149.4, 143.9, 136.4, 128.6, 128.3, 126.5, 123.2, 121.4, 60.9, 49.7, 38.0, 6.9, 4.6; ***m/z*** (ESI+) calcd. for $C_{20}H_{30}NOSi^+$ $[M+H]^+$ 328.2091, found: 328.2080.

Preparation of 2-(9*H*-Xanthen-9-yl)ethan-1-ol S18

Step 1: 2-(9*H*-xanthen-9-yl)acetic acid S17

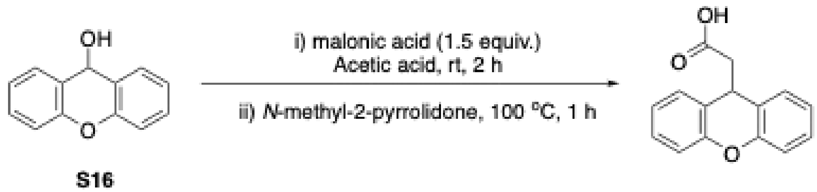

S16 S17 77%

9*H*-Xanthen-9-ol **S16** (1 g, 5 mmol, 1 equiv.), malonic acid (0.78 g, 7.5 mmol, 1.5 equiv.) and acetic acid (20 mL) was stirred at RT for 2 h. The reaction mixture was diluted with EtOAc (20 mL), water (10 mL) and brine (10 mL) and the layers were separated. The aqueous layer was extracted with EtOAc (3 x 20 mL). The organics were combined, filtered through a hydrophobic frit and concentrated *in vacuo* to give a blue oil. The oil was dissolved in *N*-methylpyrrolidone (NMP) (10 mL) and heated to 100 °C for 1 h. The reaction was diluted with toluene and saturated LiCl solution (20 mL) and aqueous layer was extracted with toluene (3 x 20 mL). The organics were combined, filtered, and concentrated *in vacuo* to give an oil. The oil was diluted with 15% aqueous NaOH solution (20 mL) and extracted with toluene (2 x 30 mL). The aqueous layer was then acidified to pH 1 with 6 M aqueous HCl solution and extracted with toluene (2 x 30 mL) and EtOAc (2 x 30 mL). The organic layers were combined, filtered and concentrated *in vacuo* to give a white gum. Purification by chromatography (100% hexanes →30% EtOAc in hexanes) afforded 2-(9*H*-xanthen-9-yl)acetic acid as a white solid **S17** (925 mg, 77%). **Mp** 153 – 155 °C; $\mathbf{v_{max}}$ (neat)/cm^{-1} 3058, 2932, 2676, 1719, 1584, 1476, 1261; **^{1}H NMR** (400 MHz, $CDCl_3$) δ 7.32 (dd, *J* = 1.3, 7.5 Hz, 2H), 7.29 - 7.24 (m, 2H), 7.16 - 7.06 (m, 4H), 4.55 (t, *J* = 7.0 Hz, 1H), 2.75 (d, *J* = 7.0 Hz, 2H); **^{13}C NMR** (101 MHz, $CDCl_3$) δ 176.7, 152.2, 128.3, 128.2, 124.2, 123.5, 116.7, 45.2, 35.5; ***m/z*** (ESI+) calcd. for $C_{15}H_{13}O_3$ $[M+H]^+$ 241.0859, found 241.0859.

Step 2: 2-(9*H*-xanthen-9-yl)ethan-1-ol S18

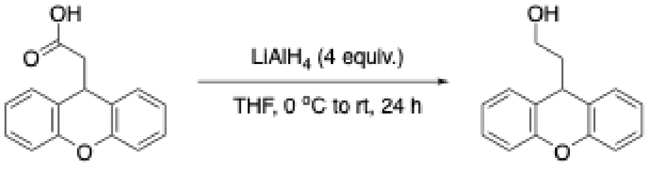

S17 S18 45%

This experiment followed General Procedure A using 2-(9*H*-xanthen-9-yl)acetic acid **S17** (0.9 g, 3.74 mmol, 1 equiv.), $LiAlH_4$ (0.43 g, 11.2 mmol, 3 equiv.) and anhydrous THF (25 mL) for 16 h. Purification by chromatography (100% hexanes→30% EtOAc in hexanes) afforded 2-(9*H*-xanthen-9-yl)ethan-1-ol **S18** as a pale yellow oil (355 mg, 45%). **^{1}H NMR** (400 MHz, $CDCl_3$) δ 7.31 - 7.23 (m, 4H), 7.17 - 7.08 (m, 4H), 4.21 (t, *J* = 6.8 Hz, 1H), 3.61 (t, *J* = 6.5 Hz, 2H), 1.98 (q, *J* = 6.5 Hz, 2H), 1.50 (br s, 1H); **^{13}C NMR** (101 MHz, $CDCl_3$) δ 151.8, 128.2, 127.2, 124.8, 122.8, 116.0, 59.0, 42.3, 35.3. Data were in accordance with previously reported literature.[13]

Preparation of 3-(4-(*tert*-Butyl)phenyl)propan-1-ol S21

Step 1: Methyl 3-(4-(*tert*-butyl)phenyl)propanoate S20

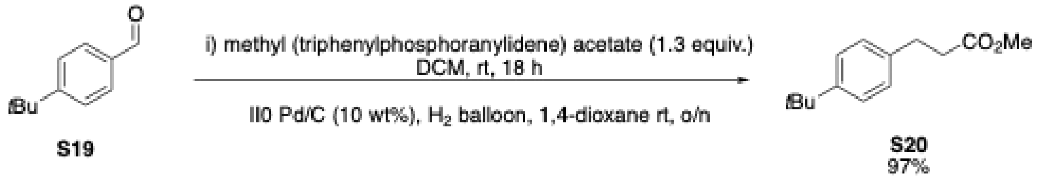

The first step was carried out according to general procedure C using 4-(*tert*-butyl)benzaldehyde **S19** (3.5 mL, 20 mmol, 1 equiv.), methyl (triphenylphosphoranylidene)acetate (8.6 g, 26 mmol, 1.3 equiv.) in anhydrous DCM (50 mL). Purification by chromatography (100% hexanes ϒ30% EtOAc in hexanes) affording the product as a yellow oil (4.3 g, 99%). The second step followed general procedure D using 3-(4-(*tert*-butyl)phenyl)acrylate (4.1 g, 19 mmol, 1 equiv.), Pd/C (202 mg, 1.9 mmol, 10 mol%), H_2 (balloon), in dioxane (60 mL). The crude 3-(4-(*tert*-butyl)phenyl)propanoate **S20** was isolated as a yellow oil (4.05 g, 97 %) and was used directly in the next step. $\mathbf{v_{max}}$ (neat)/cm^{-1} 3012, 2958, 2871, 1741, 1521, 1439, 1366, 1271, 1199, 1113, 1024, 990, 834; **^{1}H NMR** ($CDCl_3$, 400 MHz) δ 7.34 – 7.32 (m, 2H), 7.16 – 7.14 (m, 2H), 3.69 (s, 3H), 2.94 (t, *J* = 8.2 Hz, 2H), 2.66 – 2.62 (m, 2H), 1.32 (s, 9H); **^{13}C NMR** (101 MHz, $CDCl_3$) δ 172.9, 148.5, 136.9, 127.4, 124.9, 51.0, 35.1, 33.8, 30.8, 29.9; ***m/z*** (ESI+) calcd. for $C_{14}H_{20}O_2$ $[M+H]^+$ 221.1536, found 221.1538. NMR data were in accordance with the previous literature.[14]

Step 2: 3-(4-(*tert*-Butyl)phenyl)propan-1-ol S21

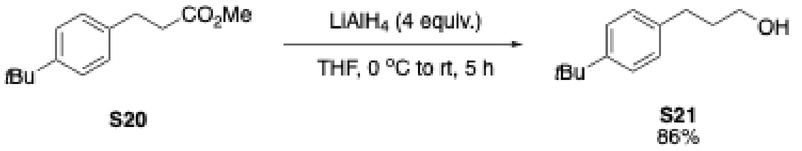

This experiment followed general procedure A using 3-(4-(*tert*-butyl)phenyl)propanoate **S20** (2.2 g, 10 mmol, 1 equiv.), $LiAlH_4$ (1.5 g, 40 mmol, 4 equiv.) and anhydrous THF (50 mL). The reaction was quenched with water (1.5 mL), 15% aq. NaOH (1.5 mL) and water (4.5 mL) affording 3-(4-(*tert*-butyl)phenyl)propan-1-ol **S21** as a yellow oil (1.65 g, 86%). $\mathbf{v_{max}}$ (neat)/cm^{-1} 3348, 3114, 3066, 2979, 2883, 1910, 1524, 1473, 1417, 1366, 1278, 1211, 1115, 1060, 1024, 921, 833, 809, 571; **^{1}H NMR** ($CDCl_3$, 400 MHz) δ 7.34 – 7.31 (m, 2H), 7.17 – 7.14 (m, 2H), 3.69 (t, *J* = 5.9 Hz, 2H), 2.69 (t, *J* = 7.95 Hz, 2H), 1.94 – 1.87 (m, 2H), 1.33 (s, 9H); **^{13}C NMR** (101 MHz, $CDCl_3$) δ 148.1, 138.2, 127.5, 124.7, 61.9, 33.8, 33.7, 31.0, 30.9; ***m/z*** (ESI+) 193.2 $[M+H^+]$. Data were in accordance with the previous literature.[15]

3-(Naphthalen-1-yl)propan-1-ol S23

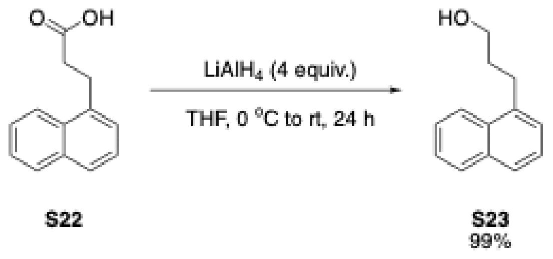

This experiment followed general procedure A using 3-(naphthalen-1-yl)propanoic acid **S22** (1 g, 5 mmol, 1 equiv.) $LiAlH_4$ (570 mg, 15 mmol, 3 equiv.) and anhydrous THF (50 mL) and afforded 3-(naphthalen-1-yl)propan-1-ol **S23** as a pale yellow oil (920 mg, >99%). $\mathbf{v_{max}}$ (neat)/cm^{-1} 3323, 3057, 2937, 1595, 1508, 1394, 1165, 1055, 1006, 775, 732, 692; **^{1}H NMR** (400 MHz, $CDCl_3$) δ 8.08 (d, *J* = 8.3 Hz, 1H), 7.87 (d, *J* = 7.8 Hz, 1H), 7.74 (d, *J* = 8.0 Hz, 1H), 7.57 - 7.46 (m, 2H), 7.45 - 7.33 (m, 2H), 3.77 (t, *J* = 6.4 Hz, 2H), 3.20 (t, *J* = 7.8 Hz, 2H), 2.09 - 1.99 (m, 2H), 1.58 (br s, 1H); **^{13}C NMR** (101 MHz, $CDCl_3$) δ 138.2, 134.2, 132.1, 129.1, 127.0, 126.3, 126.1, 125.8, 125.8, 124.1, 62.8, 33.8, 29.5; ***m/z*** (EI): 186.1 (M^+, 50), 167.1 (16), 153.1 (47), 141.1 (100), 115.1 (46), 102.0 (3), 89.0 (4), 77.0 (4). Data were in accordance with previously reported literature.[16]

Preparation of 3-(Naphthalen-2-yl)propan-1-ol S26

Step 1: Methyl 3-(naphthalen-2-yl)propanoate S25

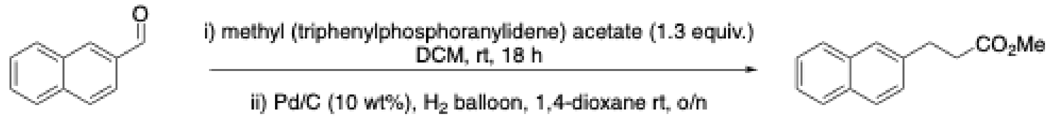

S24 S25
86% over 2 steps

The first step followed general procedure C using 2-naphthaldehyde **S24** (2.34 g, 15 mmol, 1 equiv.), methyl (triphenylphosphoranylidene)acetate (6.5 g, 19.5 mmol, 1.3 equiv.) in anhydrous DCM (40 mL). Purification by column chromatography (10% EtOAc in hexanes→30% EtOAc in hexanes) afforded methyl 3-(naphthalen-2-yl)acrylate as a white solid (2.98 g, 94%). The second step followed general procedure D using methyl 3-(naphthalen-2-yl)acrylate (2.9 g, 13.7 mmol, 1 equiv.), Pd/C (150 mg, 1.4 mmol, 10 mol%), H_2 (balloon), in dioxane (60 mL) and afforded methyl 3-(naphthalen-2-yl)propanoate **S25**, isolated as a white solid (2.71 g, 92%) (86% over 2 steps). **Mp**: 57-60 °C; $\mathbf{v_{max}}$ (neat)/cm^{-1} 3074, 3034, 2971, 1739, 1612, 1517, 1437, 1370, 1310, 1286, 1203, 1147, 1060, 1020, 988, 901, 869, 825, 746, 694, 623, 531; **^{1}H NMR** ($CDCl_3$, 400 MHz) δ 7.83 – 7.78 (m, 3H), 7.65 (s, 1H), 7.49 – 7.42 (m, 2H), 7.35 (dd, 1H, *J* = 1.72, 8.4 Hz), 3.69 (s, 3H), 3.13 (t, *J* = 7.7 Hz, 2H), 2.74, (t, *J* = 7.5 Hz, 2H); **^{13}C NMR** (101 MHz, $CDCl_3$) δ 172.8, 137.5, 133.1, 131.6, 127.6, 127.1, 127.0, 126.4, 125.9, 125.5, 124.9, 51.1, 35.1, 30.6. Analytical data were in accordance with previous literature.[17]

Step 2: 3-(Naphthalen-2-yl)propan-1-ol S26

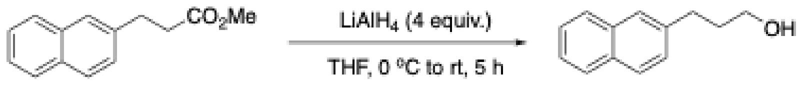

S25 S26
82%

This experiment followed general procedure A using methyl 3-(naphthalen-2-yl)propanoate **S25** (2.57 g, 12 mmol, 1 equiv.), $LiAlH_4$ (1.82 g, 48 mmol, 4 equiv.) and anhydrous THF (50 mL). The reaction was quenched with water (1.9 mL), 15% aq. NaOH (1.9 mL) and water (5.7

mL). Purification by chromatography (100% hexanes→50% EtOAc in hexanes) afforded 3-(naphthalen-2-yl)propan-1-ol **S26** as a white solid (1.79 g, 82 %). **Mp**: 34 – 36 °C (lit. 35 – 36 °C[18]); $\mathbf{v_{max}}$ (neat)/cm^{-1} 3316, 3074, 3034, 2959, 2883, 1608, 1517, 1481, 1445, 1385, 1370, 1290, 1215, 1179, 1127, 1044, 1008, 976, 901, 825, 742, 646, 563; **^{1}H NMR** ($CDCl_3$, 400 MHz) δ 7.82 – 7.77 (m, 3H), 7.64 (s, 1H), 7.48 – 7.40 (m, 2H), 7.35 (dd, *J* = 1.70, 8.4 Hz, 1H), 3.71 (t, *J* = 6.5 Hz, 2H), 2.88 (t, *J* = 7.9 Hz, 2H), 2.03 – 1.96 (m, 2H), 1.40 (s, 1H, OH); **^{13}C NMR** (101 MHz, $CDCl_3$) δ 138.8, 133.1, 131.5, 127.4, 127.1, 126.9, 126.7, 125.9, 125.4, 124.6, 61.7, 33.5, 31.7; ***m/z*** (EI) 186.1 (M$^+$, 44), 167.1 (20), 142.1 (100), 115.1 (38), 98 (3), 77 (2). Analytical data were in agreement with data previously reported in the literature.[15]

Preparation of 3-([1,1'-Biphenyl]-4-yl)propan-1-ol S29

Step 1: 3-([1,1'-Biphenyl]-4-yl)prop-2-yn-1-ol S28

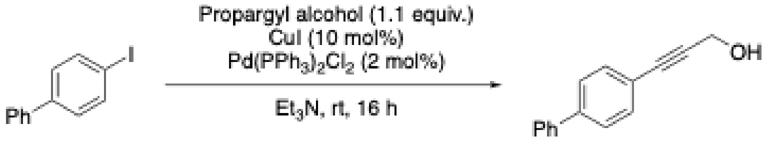

S27 **S28** 73%

Et_3N (4 mL, 0.45 M) was degassed in a microwave vial for 10 min after which 4-iodo-1,1'-biphenyl **S27** (500 mg, 1.79 mmol, 1 equiv.), $Pd(PPh_3)_2Cl_2$ (25 mg, 0.036 mmol, 2 mol%) and CuI (13.6 mg, 0.072 mmol, 4 mol%) were added and the reaction mixture stirred for 5 min. Then, propargyl alcohol (115 µL, 1.97 mmol, 1.1 equiv.) was added and the reaction stirred at RT overnight (16 h). The reaction was diluted with EtOAc (20 mL) and 1 M aqueous HCl solution (20 mL) and the layers were separated. The aqueous layer was extracted with EtOAc (2 x 20 mL) and the organics were combined, filtered through a hydrophobic frit and concentrated *in vacuo* to give the crude product. Purification by chromatography (100% hexanes→40% Et_2O in hexanes) afforded 3-([1,1'-biphenyl]-4-yl)prop-2-yn-1-ol **S28** as an orange gum (271 mg, 73%). $\mathbf{v_{max}}$ (neat)/cm^{-1} 3253, 3074, 3046, 2939, 2883, 2252, 2969, 1938, 1691, 1493, 1449, 1409, 1370, 1278, 1234, 1123, 1044, 1016, 960, 841, 766, 730, 698, 678, 559; **^{1}H NMR** (400 MHz, $CDCl_3$) δ 7.63 - 7.50 (m, 6H), 7.49 - 7.43 (m, 2H), 7.41 - 7.34 (m, 1H), 4.54 (s, 2H), 1.87 (br s, 1H); **^{13}C NMR** (101 MHz, $CDCl_3$) δ 141.3, 140.3, 132.1, 128.9, 127.7, 127.03, 127.02, 121.4, 87.9, 85.6, 51.7; ***m/z*** (EI) 208.1 (M$^+$, 100), 178.1 (70), 152.1 (32), 131.1 (29), 103.1 (9), 77.0 (12). Data were in accordance with previously reported literature.[18]

Step 2: 3-([1,1'-Biphenyl]-4-yl)propan-1-ol S29

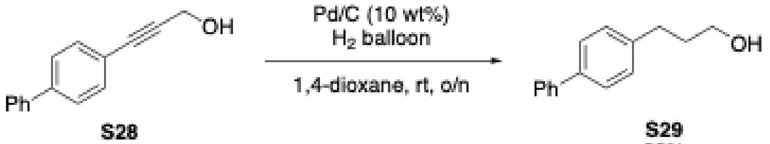

S28 **S29** 92%

This experiment followed general procedure D using 3-([1,1'-biphenyl]-4-yl)prop-2-yn-1-ol (250 mg, 1.2 mmol, 1 equiv.) **S28**, Pd/C [10 wt.%] (10 mol%, 127 mg, 0.12 mmol) and 1,4-dioxane (10 mL) for 16 h. Purification by chromatography (100% hexanes→80% Et_2O in

hexanes) affording 3-([1,1'-biphenyl]-4-yl)propan-1-ol **S29** as a white solid (235 mg, 92%). **Mp** 59 – 61 °C (lit. 60 – 62 °C[23]); **ν_{max}** (neat)/cm^{-1} 3336, 3082, 3050, 2951, 2875, 1965, 1894, 1612, 1572, 1528, 1501, 1409, 1389, 1167, 1131, 1076, 1052, 1016, 913, 873, 817, 766, 742, 690, 555, 511; **^{1}H NMR** (400 MHz, $CDCl_3$) δ 7.61 (dd, *J* = 1.3, 8.3 Hz, 2H), 7.56 (d, *J* = 8.5 Hz, 2H), 7.46 (t, *J* = 7.8 Hz, 2H), 7.36 (tt, *J* = 1.6, 7.4 Hz, 1H), 7.31 (d, *J* = 8.0 Hz, 2H), 3.75 (t, *J* = 6.5 Hz, 2H), 2.83 - 2.75 (m, 2H), 2.02 - 1.93 (m, 2H), 1.45 (br s, 1H); **^{13}C NMR** (101 MHz, $CDCl_3$) δ 140.6, 140.4, 138.4, 128.4, 128.2, 126.9, 126.7, 126.6, 61.8, 33.7, 31.2; ***m/z*** (EI) 212.1 (M^+, 59), 194.1 (34), 167.1 (100), 151.1 (33), 132.9 (5), 115.1 (8). Data were in accordance with previously reported literature.[19]

Preparation of 3-([1,1'-Biphenyl]-3-yl)propan-1-ol S32

Step 1: Methyl 3-([1,1'-biphenyl]-3-yl)propanoate S31

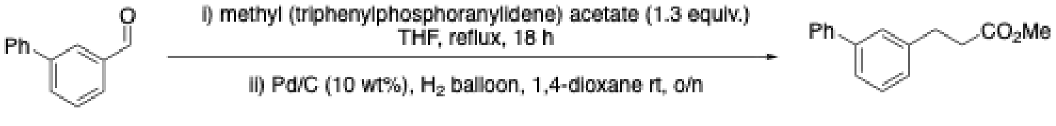

S30

S31
74%

The first step was carried out according to general procedure C using methyl (triphenylphosphoranylidene)acetate (5.5 g, 16.5 mmol, 1.5 equiv.), [1,1'-biphenyl]-3-carbaldehyde **S30** (2 g, 10.9 mmol, 1 equiv.) and anhydrous THF (50 mL) refluxed at 65 °C for 18h, affording methyl 3-([1,1'-biphenyl]-3-yl)acrylate as a white crystalline powder (2.06 g, 79%). The second step was carried out according to general procedure D using methyl 3-([1,1'-biphenyl]-3-yl)acrylate (2.06 g, 9.5 mmol, 1 equiv.), Pd/C (206 mg, 10 wt%), and 1,4-dioxane (50 mL) affording methyl 3-([1,1'-biphenyl]-3-yl)propanoate **S31** (1.7 g, 74%) as a yellow oil. **ν_{max}** (neat)/cm^{-1} 2949, 1734, 1598, 1479, 1435, 1363, 1303, 1195, 1153, 1091, 1023, 985, 887, 800, 756, 698, 615; **^{1}H NMR** (400 MHz, $CDCl_3$) δ 7.61 – 7.55 (m, 2H), 7.47 – 7.40 (m, 4H), 7.40 – 7.31 (m, 2H), 7.22 – 7.17 (m, 1H), 3.69 (s, 3H), 3.03 (t, *J* = 7.8 Hz, 2H), 2.74 – 2.62 (m, 2H); **^{13}C NMR** (101 MHz, $CDCl_3$) δ 173.5, 141.7, 141.3, 141.2, 129.1, 128.9, 127.4, 127.4, 127.3, 125.3, 51.8, 35.9, 31.2,; ***m/z*** (EI) 240.1 (M^+, 55), 209.1 (4), 180.1 (100), 165.1 (41), 152.0 (27), 139.0 (4), 128.1 (4), 89.0 (4), 77.0 (5). NMR data were in agreement with literature reports.[20]

Step 2: 3-([1,1'-Biphenyl]-3-yl)propan-1-ol S32

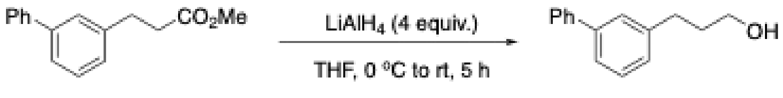

S31

S32
96%

This experiment followed general procedure A, using methyl 3-([1,1'-biphenyl]-3-yl)propanoate **S31** (1 g, 4.16 mmol, 1 equiv.), $LiAlH_4$ (632 mg, 16.6 mmol 4 equiv.) and THF (40 mL) affording 3-([1,1'-biphenyl]-3-yl)propan-1-ol **S32** (849 mg, 96 %) as a white powder. **ν_{max}** (neat)/cm^{-1} 3353, 3282, 2926, 2858, 1598, 1477, 1419, 1361, 1232, 1155, 1055, 1037, 918, 896, 800, 754, 723, 698, 615; **^{1}H NMR** (400 MHz, $CDCl_3$) δ 7.63 – 7.56 (m, 2H), 7.48 – 7.40 (m, 4H), 7.40 – 7.31 (m, 2H), 7.22 – 7.17 (m, 1H), 3.73 – 3.67 (m, 2H), 2.83 – 2.74 (m,

2H), 2.01 – 1.90 (m, 2H); **^{13}C NMR** (101 MHz, $CDCl_3$) δ 142.5, 141.5, 141.4, 129.0, 128.9, 127.5, 127.5, 127.4, 127.3, 124.9, 62.4, 34.4, 32.3; ***m/z*** (EI): 212.1 (M^+, 56), 194.1 (12), 179.1 (25), 168.1 (100), 152.1 (47), 141.1 (4), 128.1 (8), 115.0 (16), 102.0 (4), 91.0 (4), 77.0 (9).

Preparation of 3-([1,1'-biphenyl]-2-yl)propan-1-ol S35

Step 1: Methyl 3-([1,1'-biphenyl]-2-yl)propanoate S34

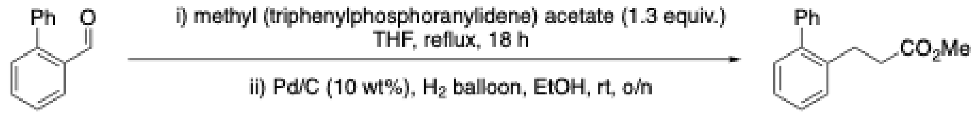

S33

S34
80% over 2 steps

The first step followed general procedure C using methyl(triphenylphosphoranylidene)acetate (5.5 g, 16.5 mmol, 1.5 equiv.), [1,1'-biphenyl]-2-carbaldehyde **S33** (2 g, 10.9 mmol, 1 equiv.) and anhydrous THF (50 mL) affording methyl 3-([1,1'-biphenyl]-2-yl)acrylate as a yellow oil (2.47 g, 95 %). The second step was carried out according to general procedure D using methyl 3-([1,1'-biphenyl]-2-yl)acrylate (2.47 g, 10.4 mmol, 1 equiv.), Pd/C (247 mg, 10 wt%), and EtOH (50 mL) affording methyl 3-([1,1'-biphenyl]-2-yl)propanoate **S34** (2.09 g, 84 %) as a yellow oil (80% over 2 steps). **v_{max}** (neat)/cm^{-1} 2949, 1735, 1479, 1435, 1363, 1294, 1251, 1155, 1105, 1008, 985, 835, 748, 702, 617; **^{1}H NMR** (400 MHz, $CDCl_3$) δ 7.44 – 7.38 (m, 2H), 7.38 – 7.34 (m, 1H), 7.32 – 7.31 (m, 1H), 7.30 – 7.27 (m, 3H), 7.26 – 7.23 (m, 1H), 7.23 – 7.19 (m, 1H), 3.60 (s, 3H), 2.97 – 2.90 (m, 2H), 2.46 – 2.39 (m, 2H); **^{13}C NMR** (101 MHz, $CDCl_3$) δ 173.4, 142.2, 141.6, 138.0, 130.4, 129.2, 129.2, 128.4, 127.7, 127.2, 126.4, 51.7, 35.3, 28.5; ***m/z*** (EI): 240.1 (M^+, 84), 209.1 (11), 180.1 (100), 165.1 (96), 152.1 (47), 139.0 (0), 128.0 (5), 115.0 (11), 89.0 (7), 77.0 (7).

Step 2: 3-([1,1'-biphenyl]-2-yl)propan-1-ol S35

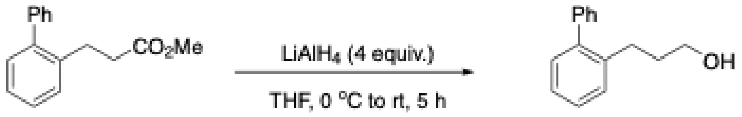

S34

S35
100%

This experiment followed general procedure A using methyl 3-([1,1'-biphenyl]-2-yl)propanoate **S34** (1 g, 4.16 mmol, 1 equiv.), $LiAlH_4$ (632 mg, 16.6 mmol 4 equiv.) and THF (30 mL) affording 3-([1,1'-biphenyl]-2-yl)propan-1-ol **S35** as a colourless oil (881 mg, 100 %). **v_{max}** (neat)/cm^{-1} 3325, 2933, 2866, 1597, 1477, 1435, 1053, 1039, 1008, 914, 748, 700, 617; **^{1}H NMR** (400 MHz, $CDCl_3$) δ 7.45 – 7.38 (m, 2H), 7.38 – 7.29 (m, 5H), 7.26 – 7.20 (m, 2H), 3.50 (t, *J* = 6.4 Hz, 2H), 2.75 – 2.65 (m, 2H), 1.76 – 1.66 (m, 2H), 1.35 – 1.01 (m, 1H); **^{13}C NMR** (101 MHz, $CDCl_3$) δ 142.1, 141.9, 139.4, 130.3, 129.4, 129.3, 128.3, 127.7, 127.0, 126.0, 62.4, 34.3, 29.3; ***m/z*** (EI): 212.1 (M^+, 29), 194.1 (23), 179.1 (86), 165.1 (100), 152.1 (41), 139.1 (7), 128.1 (7), 115.1 (12), 89.1 (4), 76.0 (4). ***m/z*** (ESI+) calcd. for $C_{15}H_{16}O^{23}Na$: 235.1093 found 235.1093.

3-(Quinolin-2-yl)propan-1-ol S37

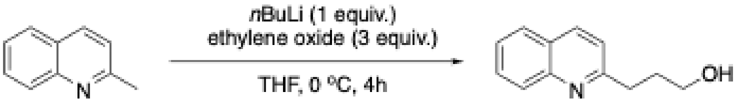

S36 S37 18%

This general procedure follows an adapted procedure from the literature.[21] Under an atmosphere of argon, quinaldine **S36** (1 g, 7 mmol, 1 equiv.) was dissolved in anhydrous THF (15 mL) and the solution cooled to 0 °C, followed by the dropwise addition of *n*BuLi (2.8 mL, 2.3 M, 1 equiv.). After 30 min, ethylene oxide (8.4 mL, 21 mmol, 3 equiv.), was added, and the reaction stirred at RT under argon overnight. The reaction was quenched with water, extracted with ethyl acetate (3 x), the organic phases combined and dried over Na_2SO_4. The crude mixture was purified by chromatography (20% EtOAc→100% EtOAc→30% MeOH in EtOAc) affording 3-(quinolin-2-yl)propan-1-ol **S37** (232 mg, 18%) as a dark yellow oil. **ν_{max}** (neat)/cm^{-1} 3265, 2930, 2864, 1618, 1599, 1504, 1425, 1314, 1053; **^{1}H NMR** (400 MHz, $CDCl_3$) δ 8.06 (d, *J* = 8.3 Hz, 1H), 8.00 (d, *J* = 8.6 Hz, 1H), 7.78 – 7.74 (m, 1H), 7.69 – 7.63 (m, 1H), 7.50 – 7.45 (m, 1H), 7.30 (d, *J* = 8.5 Hz, 1H), 3.77 (t, *J* = 5.9 Hz, 2H), 3.15 (t, *J* = 6.8 Hz, 2H), 3.15 (quint., *J* = 6.3 Hz, 2H); **^{13}C NMR** (101 MHz, $CDCl_3$) δ161.2, 147.3, 136.8, 129.7, 128.4, 127.6, 126.8, 126.1, 121.8, 62.4, 36.4, 31.4; ***m/z*** (ESI+) calcd. for $C_{12}H_{14}NO$ $[M+H]^+$ 188.1070, found 188.1067.

3-(Pyridin-4-yl)propan-1-ol S39

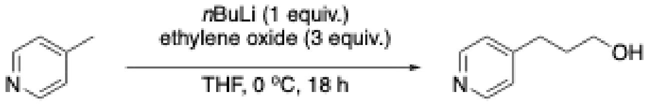

S38 S39 56%

This general procedure follows an adapted procedure from the literature.[21] Under an atmosphere of argon, 4-picoline **S38** (1.06 mL, 10.7 mmol, 1 equiv.) was dissolved in anhydrous THF (15 mL) and the solution cooled to -96 °C, followed by the dropwise addition of *n*BuLi (4.3 mL, 2.5M, 10.7 mmol, 1 equiv.). After 30 mins, ethylene oxide (12.8 mL, 32.1 mmol, 3 equiv.) was added, and the reaction stirred at RT under argon overnight. The reaction was quenched with water, extracted with ethyl acetate (3 x), the organic phases combined and dried over Na_2SO_4. The crude mixture was purified by chromatography (100% EtOAc→30% MeOH in EtOAc) affording 3-(pyridin-4-yl)propan-1-ol **S39** (82 mg, 56%) as an orange oil. **ν_{max}** (neat)/cm^{-1} 3258, 2937, 1605, 1417, 1220, 1057, 1002, 916; **^{1}H NMR** (400 MHz, $CDCl_3$) δ 8.45 (dd, *J* = 4.4, 1.5 Hz, 2H), 7.12 (dd, *J* = 4.5, 1.4 Hz, 2H), 3.67 (t, *J* = 6.2 Hz, 2H), 2.71 (t, *J* = 7.5 Hz, 2H), 1.93 – 1.85 (m, 2H); **^{13}C NMR** (101 MHz, $CDCl_3$) δ 151.3, 149.6, 124.1, 61.7, 33.2, 31.6; ***m/z*** (EI): 136 (M^+, 6), 118 (100), 106 (25), 92 (33), 77 (10), 65 (12), 51 (12). Analytical data were in agreement with data previously reported in the literature.[22]

3-(4-(Thiophen-2-yl)phenyl)propan-1-ol S41

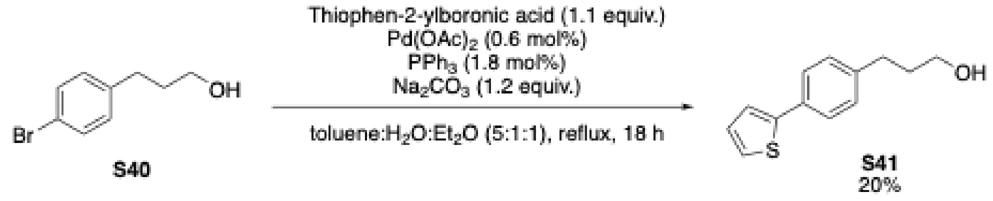

3-(4-Bromophenyl)propan-1-ol **S40** (1 g, 4.7 mmol, 1 equiv.), $Pd(OAc)_2$ (63 mg, 0.3 mmol, 0.6 mol%), thiophen-2-ylboronic acid (662 mg, 5.2 mmol, 1.1 equiv.), PPh_3 (146 mg, 0.6 mmol, 1.8 mol%) and Na_2CO_3 (591 mg, 5.6 mmol, 1.2 equiv.), were suspended in toluene (20 mL), water (4 mL) and EtOH (4 mL) and the flask was flushed with an argon. The reaction mixture was refluxed for 18 h, after which was cooled to RT, diluted with 15% aq. NaOH (20 mL), and extracted with CH_2Cl_2 (3 x 50 mL). The organics were combined, dried over Na_2CO_3, and concentrated *in vacuo*. The crude product was purified by chromatography, (100% hexanes→35% EtOAc in hexanes) affording 3-(4-(thiophen-2-yl)phenyl)propan-1-ol **S41** (200 mg, 20%) as a white solid. **Mp** 70-73 °C; $\mathbf{v_{max}}$ (neat)/cm^{-1} 3319, 3085, 2953, 2876, 1436, 1369; ^{1}**H NMR** (400 MHz, $CDCl_3$) δ 7.55 (d, *J* = 8.3 Hz, 1H), 7.30 - 7.20 (m, 5H), 7.08 (dd, *J* = 3.6, 5.1 Hz, 1H), 3.71 (t, *J* = 6.4 Hz, 2H), 2.79 - 2.69 (m, 2H), 1.99 - 1.88 (m, 2H); ^{13}**C NMR** (101 MHz, $CDCl_3$) δ (ppm) = 144.4, 141.2, 132.2, 128.9, 127.9, 126.0, 124.4, 122.7, 62.2, 34.1, 31.7; ***m/z*** (ESI+) calcd. for $C_{13}H_{15}OS$ $[M+H]^+$ 219.0838, found 219.0838.

Preparation of 3-(benzo[*b*]thiophen-3-yl)propan-1-ol S44

Step 1: Methyl 3-(benzo[*b*]thiophen-3-yl)propanoate S43

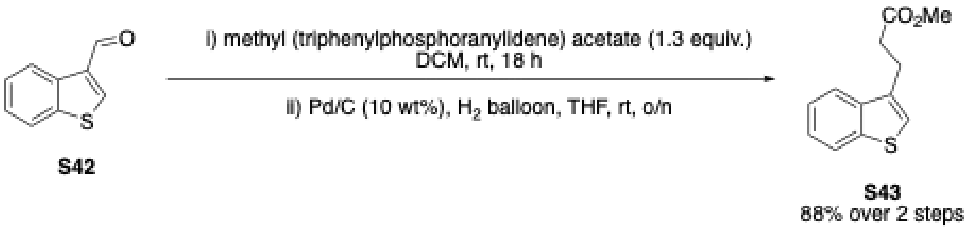

The first step was carried out according to general procedure C using benzothiophene-3-carbaldehyde **S42** (1 g, 6.2 mmol, 1 equiv.), methyl(triphenylphosphoranylidene)acetate (3.1 g, 9.1 mmol, 1.5 equiv.), and anhydrous dichloromethane (25 mL) affording methyl (*E*)-3-(benzo[*b*]thiophen-3-yl)acrylate (1.26 g, 88%). The second step followed general procedure D using methyl (*E*)-3-(benzo[*b*]thiophen-3-yl)acrylate (1 g, 4.6 mmol, 1 equiv.), 10 wt% Pd/C (100 mg, 0.46 mmol) and anhydrous THF (25 mL) affording methyl 3-(benzothiophen-3-yl)propanoate **S43** (1.01g, 100%) as a colourless oil. The crude product was carried onto the next step without further purification (88% over 2 steps). $\mathbf{v_{max}}$ (neat)/cm^{-1} 3448, 2949, 1734, 1436, 1196; ^{1}**H NMR** (400 MHz, $CDCl_3$) δ 7.88 – 7.85 (m, 1H), 7.78 – 7.74 (m, 1H), 7.42 – 7.33 (m, 2H), 7.14 (s, 1H), 3.70 (s, 3H), 3.22 – 3.17 (m, 2H), 2.80 – 2.75 (m, 2H); ^{13}**C NMR** (101 MHz, $CDCl_3$) δ 173.4, 140.6, 138.8, 135.0, 124.5, 124.1, 123.1, 121.8, 121.6, 51.9, 33.7, 23.9. ***m/z* (EI):** 218 (M+, 89), 187 (100), 158 (33), 132 (3), 115 (82), 89 (14). Analytical data were in agreement with data previously reported in the literature.[23]

Step 2: 3-(Benzo[*b*]thiophen-3-yl)propan-1-ol S44

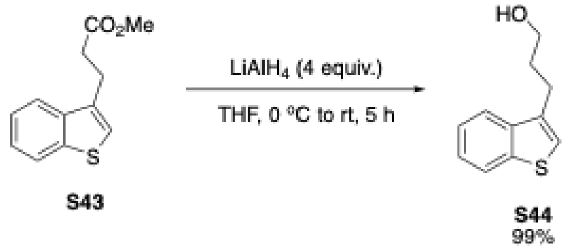

The reaction was carried out according to general procedure A using methyl 3-(benzo[*b*]thiophen-3-yl)propanoate **S43** (1 g, 4.5 mmol, 1 equiv.), $LiAlH_4$ (690 mg, 18.2 mmol, 4 equiv.) and THF (15 mL). The reaction mixture was carefully quenched with water (0.7 mL), 15% aq. NaOH (0.7 mL) and water (2.1 mL). The crude 3-(benzo[*b*]thiophen-3-yl)propan-1-ol product **S44** (868 mg, 99%) was isolated as a yellow oil. $\mathbf{v_{max}}$ (neat)/cm^{-1} 3333, 2939, 2870, 1427, 1255, 1020, 919; **^{1}H NMR** (400 MHz, $CDCl_3$) δ 7.88 – 7.84 (m, 1H), 7.79 – 7.75 (m, 1H), 7.40 – 7.32 (m, 2H), 7.12 (s, 1H), 3.76 (t, *J* = 6.2 Hz, 2H), 2.96 (td, *J* = 7.7, 1.1 Hz, 2H), 2.07 – 1.99 (m, 2H); **^{13}C NMR** (101 MHz, $CDCl_3$) δ140.7, 139.1, 136.3, 124.3, 124.0, 123.0, 121.8, 121.4, 62.5, 32.1, 24.9; ***m/z* (EI):** 192 (M+, 79), 173 (15), 147 (100), 115 (31), 89 (11). Analytical data are in agreement with data previously reported in the literature.[1]

3-(4-Ethylphenyl)propan-1-ol S46

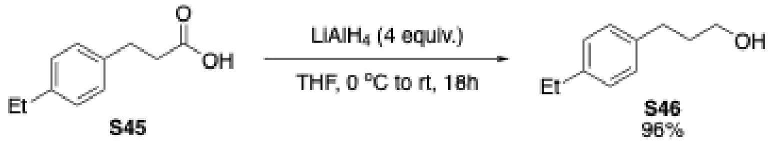

This experiment was carried out according to general procedure A using 3-(4-ethylphenyl)propanoic acid **S45** (600 mg, 3.4 mmol, 1 equiv.), $LiAlH_4$ (511 mg, 13.5 mmol, 4 equiv.) and THF (20 mL). The reaction mixture was carefully quenched with water (0.5 mL), 15% aq. NaOH (0.5 mL) and water (1.5 mL). The crude 3-(4-ethylphenyl)propan-1-ol **S46** product (868 mg, 99%) was isolated as a yellow oil and was sufficiently pure. $\mathbf{v_{max}}$ (neat)/cm^{-1} 3319, 2963, 2930, 2870, 1514, 1452, 1057, 1033, 913; **^{1}H NMR** (400 MHz, $CDCl_3$) δ 7.13 (s, 4H), 3.68 (t, *J* = 6.4 Hz, 2H), 2.72 – 2.66 (m, 2H), 2.65 – 2.59 (m, 2H), 1.95 – 1.85 (m, 2H), 1.24 (t, *J* = 7.5 Hz, 3H); **^{13}C NMR** (101 MHz, $CDCl_3$) δ 141.9, 139.1, 128.5, 128.0, 62.5, 34.4, 31.8, 28.6, 15.8; ***m/z*** (ESI+) calcd. for $C_{11}H_{17}O$ $[M+H]^+$ 165.1274, found 165.1271. Analytical data were in agreement with data previously reported in the literature.[24]

(3-Methoxypropane-1,1-diyl)dibenzene S47

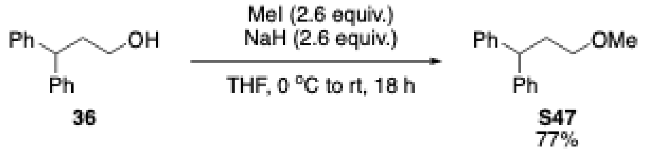

This experiment was carried out according to a modified literature procedure.[30] A solution of 3,3-diphenylpropan-1-ol **36** (500 mg, 2.4 mmol) in anhydrous THF (5 mL) was added dropwise

to a suspension of NaH (146 mg, 6.1 mmol, 2.6 equiv.) in anhydrous THF (10 mL) at 0 °C under an atmosphere of argon. The reaction mixture was stirred at 0 °C for 1 h before the addition of MeI (0.4 mL, 6.1 mmol, 2.6 equiv.) and the reaction mixture left to warm to RT and stir for 18 h. After reaction, the mixture was quenched with water (20 mL) and extracted with diethyl ether (4 x 20 mL). The combined organics were dried over Na_2SO_4 and concentrated *in vacuo*, affording (3-methoxypropane-1,1-diyl)dibenzene **S47** (409 mg, 77%) as a colourless oil, sufficiently pure to not require further purification. $\mathbf{v_{max}}$ (neat)/cm^{-1}; 3026, 2920, 2872, 1600, 1492, 1387, 1186,1119; ^{1}**H NMR** ($CDCl_3$, 400 MHz) δ 7.32 – 7.23 (m, 8H), 7.21 – 7.16 (m, 2H), 4.12 (t, *J* = 7.8 Hz, 1H), 3.33 – 3.28 (m, 5H, overlapping t, 2H and s, 3H), 2.32 (q, *J* = 6.8 Hz, 2H); ^{13}**C NMR** (101 MHz, $CDCl_3$) δ 144.8, 128.6, 128.0, 126.3, 70.8, 58.7, 47.4, 35.4; ***m/z*** (EI) 226 (M+, 5), 194 (100), 179 (37), 167 (97), 152 (46), 139 (6), 116 (37). Analytical data were in agreement with data previously reported in the literature.[25]

(3-Ethoxypropane-1,1-diyl)dibenzene S48

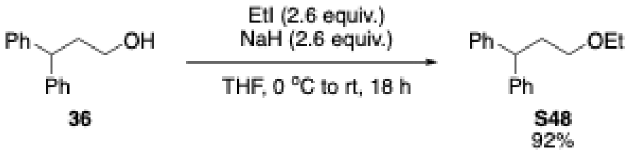

This experiment was carried out according to a modified literature procedure.[30] A solution of 3,3-diphenylpropan-1-ol **36** (500 mg, 2.4 mmol) in anhydrous THF (5 mL) was added dropwise to a suspension of NaH (146 mg, 6.1 mmol, 2.6 equiv.) in anhydrous THF (10 mL) at 0 °C under an atmosphere of argon. The reaction mixture was stirred at 0 °C for 1 h before the addition of EtI (0.5 mL, 6.1 mmol, 2.6 equiv.) and the reaction mixture left to warm to RT and stir for 18 h. After reaction, the mixture was quenched with water (20 mL) and extracted with diethyl ether (4 x 20 mL). The combined organics were dried over Na_2SO_4 and concentrated *in vacuo*, affording (3-ethoxypropane-1,1-diyl)dibenzene **S48** (522 mg, 92%) as an orange oil, sufficiently pure to not require further purification. $\mathbf{v_{max}}$ (neat)/cm^{-1} 3082, 3044, 2988, 2879, 1505, 1461, 1118, 755; ^{1}**H NMR** ($CDCl_3$, 400 MHz) δ 7.31 – 7.23 (m, 8H), 7.20 – 7.14 (m, 2H), 4.11 (t, *J* = 7.8 Hz, 1H), 3.41 (q, *J* = 7.0 Hz, 2H), 3.33 (t, *J* = 6.8 Hz, 2H), 2.36 – 2.30 (m, 2H), 1.18 (t, *J* = 7.0 Hz, 3H); ^{13}**C NMR** (101 MHz, $CDCl_3$) δ 144.8, 128.6, 128.1, 126.3, 68.7, 66.3, 47.5, 35.5, 15.4; ***m/z*** (ESI+) calcd. for $C_{17}H_{21}O$ $[M+H]^+$ 241.1587, found 241.1586.

(3-Phenoxypropane-1,1-diyl)dibenzene S49

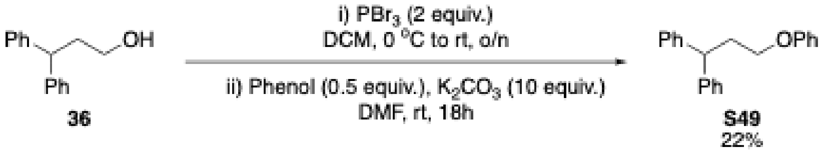

3,3-Diphenylpropan-1-ol **36** (3 g, 14.1 mmol) was dissolved in anhydrous DCM (40 mL) and cooled to 0 °C under an atmosphere of argon. PBr_3 (2.7 mL, 28.2 mmol, 2 equiv.) was added dropwise and the mixture purged with argon to vent the build-up of HBr. The reaction mixtutre was left to warm to RT and stirred overnight. After reaction, the mixture was quenched with H_2O (10 mL) and sat. $NaHCO_3$ (10 mL). The mixture was washed with brine (10 mL) and H_2O (10 mL), the organics dried over Na_2SO_4, and concentrated *in vacuo*. The crude (3-bromopropane-1,1-diyl)dibenzene (3.8 g, 97%) was sufficiently pure and carried onto the next

step. Phenol (1 g, 10.6 mmol) and K_2CO_3 (14.6 g, 106 mmol, 10 equiv.) were suspended in DMF (25 mL) followed by the addition of (3-bromopropane-1,1-diyl)dibenzene (3.8 g, 13.8 mmol, 1.3 equiv.) and the mixture stirred at RT overnight. After reaction, the mixture was filtered and the DMF removed *in vacuo*. The remaining solid was dissolved in H_2O (100 mL) and extracted with Et_2O (3 x 50 mL). The combined organics were dried over Na_2SO_4 and concentrated *in vacuo*. Purification by chromatography (100% hexanes→10% DCM in hexanes) afforded (3-phenoxypropane-1,1-diyl)dibenzene **S49** (671 mg, 22%) as a crystalline white solid. **Mp** 76-78 °C; $\mathbf{v_{max}}$ (neat)/cm^{-1} 3044, 2963, 2944, 1605, 1592, 1505, 1470, 1249, 1046; **^{1}H NMR** ($CDCl_3$, 400 MHz) δ 7.32 – 7.23 (m, 10H), 7.22 – 7.17 (m, 2H), 6.95 – 6.90 (m, 1H), 6.87 – 6.82 (m, 2H), 4.26 (t, *J* = 7.8 Hz, 1H), 3.90 (t, *J* = 6.5 Hz, 2H), 2.57 – 2.51 (m, 2H); **^{13}C NMR** (101 MHz, $CDCl_3$) δ 159.0, 144.4, 129.5, 128.7, 128.1, 126.5, 120.7, 114.7, 65.9, 47.4, 35.1; ***m/z*** (ESI+) calcd. for $C_{17}H_{21}O$ $[M+H]^+$ 241.1587, found 241.1586.

Synthesis of thioether and related substrates

3,3-Diphenylpropyl methanesulfonate S50

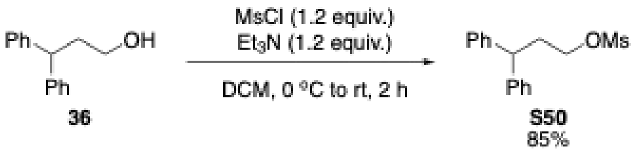

This experiment followed general procedure E using 3,3-diphenylpropan-1-ol **36** (2.1 g, 10 mmol, 1 equiv.), methanesulfonyl chloride (1 mL, 12 mmol, 1.2 equiv.), triethylamine (1.8 mL, 12 mmol, 1.2 equiv.) and anhydrous DCM (40 mL) affording 3,3-diphenylpropyl methanesulfonate **S56** was isolated as a yellow solid (2.45 g, 85%). The crude material was sufficiently pure. **Mp**: 78 – 81 °C (lit. 78 – 82 °C)[32]; $\mathbf{v_{max}}$ (neat)/cm^{-1} 3061, 3039, 2971, 2922, 1611, 1466, 1355, 1340, 1174, 1075, 974, 961, 924, 844, 826, 783, 706, 641, 567, 567, 530; **^{1}H NMR** ($CDCl_3$, 400 MHz) δ 7.32 – 7.28 (m, 4H), 7.25 – 7.18 (m, 6H), 4.16 (t, *J* = 6.4Hz, 2H), 4.13 (t, *J* = 8.1 Hz, 1H), 2.89 (s, 3H, CH_3), 2.50 (dt, *J* = 8.0, 6.4 Hz, 2H); **^{13}C NMR** (101 MHz, $CDCl_3$) δ 143.2, 128.7, 127.7, 126.7, 68.3, 46.9, 37.1, 34.7; ***m/z*** (ESI+) calculated for $C_{16}H_{18}O_3NaS$ $[M+Na]^+$ 313.0866, found 313.0870. NMR data were in agreement with data previously reported in the literature.[26]

(3,3-Diphenylpropyl)(phenyl)sulfane 54

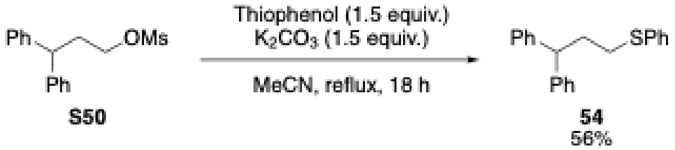

This experiment followed general procedure F using 3,3-diphenylpropyl methanesulfonate **S50** (1.16 g, 4 mmol, 1 equiv.), thiophenol (0.75 mL, 6 mmol, 1.5 equiv.), K_2CO_3 (829 mg, 6 mmol, 1.5 equiv.), in degassed acetonitrile (40 mL). Purification by chromatography (100% hexanes→20% DCM in hexanes) afforded (3,3-diphenylpropyl)(phenyl)sulfane **54** as a white solid (683 mg, 2.24 mmol, 56%). **Mp**: 69 – 71 °C; $\mathbf{v_{max}}$ (neat)/cm^{-1} 3102, 3074, 3050, 2979, 2927, 2891, 2867, 1592, 1501, 1485, 1461, 1445, 1298, 1278, 1159, 1095, 1090, 1032, 980,

913, 881, 762, 631, 595, 527; **^{1}H NMR** ($CDCl_3$, 400 MHz) δ 7.33 – 7.23 (m, 13H), 7.22 – 7.16 (m, 2H), 4.16 (t, *J* = 7.7 Hz, 1H), 2.90 – 2.86 (m, 2H), 2.41 (dt, *J* = 7.6, 7.5 Hz, 2H); **^{13}C NMR** (101 MHz, $CDCl_3$) δ 143.5, 135.8, 128.7, 128.4, 128.1, 127.4, 125.9, 125.4, 49.5, 34.5, 31.3; ***m/z*** (ESI+) calculated for $C_{21}H_{21}S$ $[M+H]^+$ 305.1358, found 305.1363. ^{1}H NMR data were in agreement with the literature.[27]

(3-(Phenylsulfinyl)propane-1,1-diyl)dibenzene 55

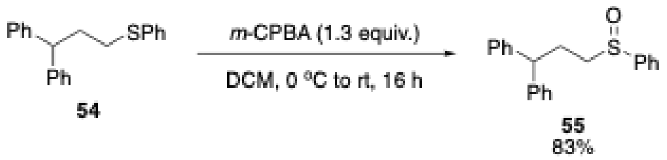

This experiment followed general procedure G using (3,3-diphenylpropyl)(phenyl)sulfane **54** (609 mg, 2 mmol, 1 equiv.), *m*-CPBA (448 mg, 2.6 mmol, 1.3 equiv.) and DCM (40 mL). Purification by chromatography (20% EtOAc in hexanes→40% EtOAc in hexanes) afforded (3-(phenylsulfinyl)propane-1,1-diyl)dibenzene **55** as a white solid (532 mg, 83%). **Mp** 82 – 84 °C; **v_{max}** (neat)/cm^{-1} 3078, 3046, 2979, 2931, 2907, 1969, 1898, 1826, 1779, 1612, 1596, 1505, 1461, 1405, 1314, 1270, 1195, 1091, 1048, 1012, 976, 913, 857, 758, 698, 639, 591, 559; **^{1}H NMR** ($CDCl_3$, 400 MHz) δ 7.56 – 7.48 (m, 5H), 7.28 – 7.24 (m, 4H), 7.19 – 7.13 (m, 6H), 3.98 (t, *J* = 7.9 Hz, 1H), 2.82 – 2.67 (m, 2H), 2.54 – 2.45 (m, 1H), 2.38 – 2.29 (m, 1H); **^{13}C NMR** (101 MHz, $CDCl_3$) δ 142.9, 142.7, 130.4, 128.7, 128.2, 128.2, 127.3, 127.2, 126.1, 126.0, 123.5, 54.6, 49.5, 26.9. ***m/z*** (ESI+) calculated for $C_{21}H_{21}O_2S$ $[M+H]^+$ 337.1256, found 337.1260.

(3,3-Diphenylpropyl)(*p*-tolyl)sulfane 56

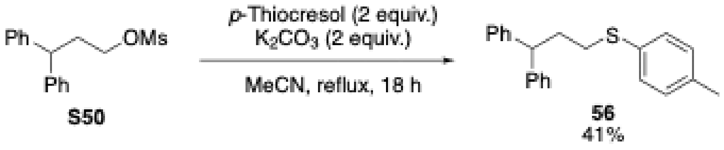

This experiment followed general procedure F using 3,3-diphenylpropyl methanesulfonate **S50** (1.16 g, 4 mmol, 1 equiv.), *p*-thiocresol (0.99 g, 8 mmol, 2 equiv.), K_2CO_3 (1.11 g, 8 mmol, 2 equiv.), in degassed acetonitrile (40 mL). Purification by chromatography (100% hexanes→20% DCM in hexanes) afforded (3,3-diphenylpropyl)(*p*-tolyl)sulfane **56** as a white solid (525 mg, 1.64 mmol, 41%). **Mp** 60 – 62 °C; **v_{max}** (neat)/cm^{-1} 3082, 3042, 2975, 2951, 2927, 1608, 1501, 1457, 1278, 1219, 1095, 1028, 797, 754, 710, 623, 587, 563; **^{1}H NMR** ($CDCl_3$, 400 MHz) δ 7.28 – 7.26 (m, 2H), 7.25 – 7.24 (m, 2H), 7.22 – 7.15 (m, 8H), 7.07 – 7.05 (m, 2H), 4.11 (t, *J* = 7.7 Hz, 1H), 2.81 – 2.78 (m, 2H), 2.36 – 2.33 (m, 2H), 2.30 (s, 3H); **^{13}C NMR** (101 MHz, $CDCl_3$) δ 143.6, 129.5, 129.1, 128.0, 127.3, 125.8, 49.4, 34.4, 32.0, 20.4; ***m/z*** (ESI) calculated for $C_{22}H_{23}S$ $[M+H]^+$ 319.1515, found 319.1524.

(3-(*p*-Tolylsulfinyl)propane-1,1-diyl)dibenzene 57

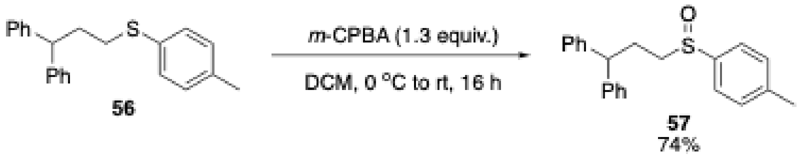

This experiment followed general procedure G using (3,3-diphenylpropyl)(*p*-tolyl)sulfane **56** (995 mg, 3 mmol, 1 equiv.), *m*-CPBA (673 mg, 3.9 mmol, 1.3 equiv.) and DCM (40 mL). Purification by column chromatography (20% EtOAc in hexanes→50% EtOAc in hexanes) afforded (3-(*p*-tolylsulfinyl)propane-1,1-diyl)dibenzene **57** as a white solid (738 mg, 2.21 mmol, 74%). **Mp** 104 – 107 °C; $\mathbf{v_{max}}$ (neat)/cm^{-1} 3042, 2939, 2883, 2442, 2335, 1612, 1505, 1453, 1091, 1060, 1048, 1024, 984, 913, 809, 758, 706, 639, 587, 551, 507; ^{1}**H NMR** ($CDCl_3$, 400 MHz) δ 7.44 – 7.42 (m, 2H), 7.27 – 7.22 (m, 6H), 7.19 – 7.13 (m, 6H), 3.98 (t, *J* = 8.04 Hz, 1H), 2.80 – 2.66 (m, 2H), 2.53 – 2.44 (m, 1H), 2.36 (s, 3H), 2.35 – 2.29 (m, 1H); ^{13}**C NMR** (101 MHz, $CDCl_3$) δ 143.0, 142.8, 140.8, 139.9, 129.4, 128.2, 128.1, 127.2, 126.1, 123.5, 54.6, 49.6, 26.9, 20.5; ***m/z*** (ESI+) calculated for $C_{22}H_{23}OS$ $[M+H]^+$ 335.1464, found 335.1467.

(3-Tosylpropane-1,1-diyl)dibenzene 58

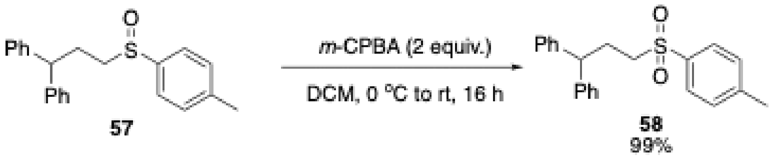

This experiment followed general procedure G using (3-(phenylsulfinyl)propane-1,1-diyl)dibenzene **57** (320 mg, 1 mmol, 1 equiv.), *m*-CPBA (345 mg, 2 mmol, 2 equiv.) and DCM (30 mL), affording (3-(phenylsulfonyl)propane-1,1-diyl)dibenzene **58** as a white solid (336 mg, 99%). **Mp** 122 – 124 °C; $\mathbf{v_{max}}$ (neat)/cm^{-1} 3074, 3038, 2979, 2943, 1612, 1501, 1453, 1421, 1334, 1239, 1234, 1147, 1091, 1024, 988, 925, 865, 821, 774, 750, 702, 670, 591, 571, 551; ^{1}**H NMR** ($CDCl_3$, 400 MHz) δ 7.75 – 7.73 (m, 2H), 7.34 – 7.32 (m, 2H), 7.27 – 7.24 (m, 4H), 7.20 – 7.13 (m, 6H), 3.93 (t, *J* = 7.9 Hz, 1H), 3.02 – 2.98 (m, 2H), 2.47 – 2.41 (m, 2H), 2.44 (s, 3H); ^{13}**C NMR** (101 MHz, $CDCl_3$) δ 144.1, 142.4, 135.6, 129.4, 128.2, 127.5, 127.1, 126.2, 54.3, 49.2, 27.8, 21.1; ***m/z*** (ESI+) calculated for $C_{22}H_{23}O_2S$ $[M+H]^+$ 351.1413, found 351.1417.

(3,3-diphenylpropyl)(methyl)sulfane 59

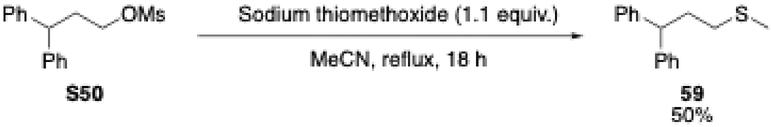

This experiment followed general procedure F using 3,3-diphenylpropyl methanesulfonate **S50** (1.45 g, 5 mmol, 1 equiv.) and sodium thiomethoxide (386 mg, 5.5 mmol, 1.1 equiv.), in degassed acetonitrile (60 mL). The crude was purified via column chromatography (100% hexanes →10% DCM in hexanes) affording (3,3-diphenylpropyl)(methyl)sulfane **59** as a colourless oil (603 mg, 2.50 mmol, 50%).

^{1}H NMR ($CDCl_3$, 400 MHz) δ: 7.31 – 7.24 (m, 8H), 7.20 – 7.16 (m, 2H), 4.10 (t, 1H, *J* = 7.6 Hz), 2.45 – 2.41 (m, 2H), 2.37 – 2.31 (m, 2H), 2.07 (s, 3H). **^{13}C NMR** (101 MHz, $CDCl_3$) δ: 144.3, 128.5, 127.9, 126.3, 49.9, 34.8, 32.5, 15.4. **ATR-IR** υ_{max} (neat)/ cm^{-1} 3125, 3103, 3075, 3044, 2929, 1611, 1589, 1498, 1461, 1277, 1086, 1033, 968, 752, 699, 640,624, 577, 552, 537, 477. **HRMS** (ESI) calculated for ($C_{16}H_{18}NaS$) $[M+Na]^+$ 265.1021, found; 265.1033

(3-(Phenylsulfonyl)propane-1,1-diyl)dibenzene 60

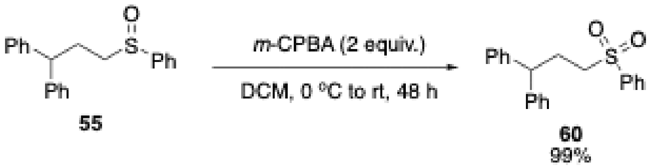

This experiment followed general procedure G using (3-(phenylsulfinyl)propane-1,1-diyl)dibenzene **55** (320 mg, 1 mmol, 1 equiv.), *m*-CPBA (345 mg, 2 mmol, 2 equiv.) and DCM (30 mL), affording (3-(phenylsulfonyl)propane-1,1-diyl)dibenzene **60** as a white solid (336 mg, 99%). **Mp** 98 – 100 °C; **v_{max}** (neat)/cm^{-1} 3102, 3078, 3038, 2990, 2975, 2951, 2935, 1779, 1715, 1612, 1588, 1505, 1457, 1326, 1314, 1294, 1227, 1155, 1091, 1080, 1028, 984, 925, 865, 786, 706, 690, 607, 539; **^{1}H NMR** ($CDCl_3$, 400 MHz) δ 7.88 – 7.86 (m, 2H), 7.67 – 7.63 (m, 1H), 7.58 – 7.52 (m, 2H), 7.28 – 7.24 (m, 4H), 7.20 – 7.12 (m, 6H), 3.94 (t, *J* = 8.1 Hz, 1H), 3.05 – 3.01 (m, 2H), 2.49 – 2.43 (m, 2H); **^{13}C NMR** (101 MHz, $CDCl_3$) δ 142.3, 138.6, 133.2, 128.8, 128.2, 127.5, 127.1, 126.2, 54.2, 49.1, 27.7; ***m/z*** (ESI+) calculated for $C_{21}H_{21}O_2S^+$ $[M-H]^+$ 337.1257, found 337.1260.

(3,3-diphenylpropyl)(4-(trifluoromethyl)phenyl)sulfane 61

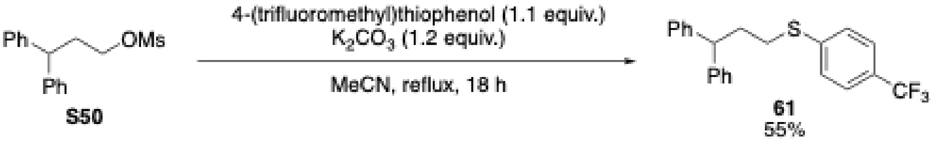

This experiment followed general procedure G using 3,3-diphenylpropyl methanesulfonate **S50** (1.45 g, 5 mmol, 1 equiv.), 4-(trifluoromethyl)thiophenol (0.74 mL, 5.5 mmol, 1.1 equiv.), K_2CO_3 (829 mg, 6 mmol, 1.2 equiv.), in degassed acetonitrile (60 mL). The crude was purified via column chromatography (100% hexanes→20% DCM in hexanes) affording (3,3-diphenylpropyl)(4-(trifluoromethyl)phenyl)sulfane **61** as a white solid (1.03 g, 2.77 mmol, 55%).

^{1}H NMR ($CDCl_3$, 400 MHz) δ:7.52 (d, 2H, *J* = 8.2 Hz), 7.37 – 7.33 (m, 4H), 7.32 – 7.24 (m, 8H), 4.18 (t, 1H, *J* = 7.8 Hz), 2.95 (t, 2H, *J* = 7.6 Hz), 2.48 (q, 2H, *J* = 7.6 Hz). **^{13}C NMR** (101 MHz, $CDCl_3$) δ: 143.7, 142.0, 128.6, 127.8, 127.3 128.5, 125.6 (q, *J* = 4.0 Hz), 50.0, 34.7, 30.6. **^{19}F NMR** δ: (376 MHz, $CDCl_3$) -62.42. **ATR-IR** υ_{max} (neat)/ cm^{-1} 3103, 3075, 6044, 2954, 2935, 2900, 2876, 1602, 1583, 1505, 1464, 1452, 1411, 1336, 1286, 1167, 1124, 1102, 1071, 1014, 961, 924, 880, 824, 786, 762, 708, 640, 596, 531, 493. Mp = 47 - 50 °C. **HRMS** (ESI) calculated for ($C_{22}H_{19}F_3NaS$) $[M+Na]^+$ 395.1051, found: 395.1035

(3,3-diphenylpropyl)(4-methoxyphenyl)sulfane 62

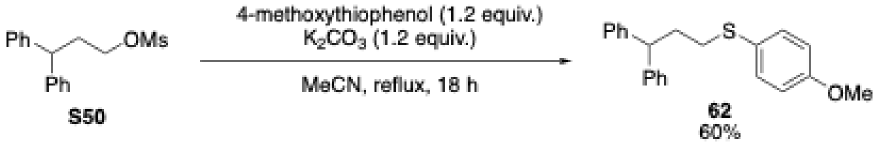

This experiment followed general procedure F using 3,3-diphenylpropyl methanesulfonate **S50** (1.45 g, 5 mmol, 1 equiv.), 4-methoxythiophenol (0.74 mL g, 6 mmol, 1.2 equiv.), K_2CO_3 (1.04 g, 7.5 mmol, 1.5 equiv.), in degassed acetonitrile (40 mL). Purification by chromatography (100% hexanes → 40% DCM in hexanes) afforded a mixture of the product and the corresponding disulfide. Column chromatography was repeated (100% hexanes→10% EtOAc/hexanes) affording (3,3-diphenylpropyl)(4-methoxyphenyl)sulfane **62** as a white solid (1.01 g, 3.02 mmol, 60%).

^{1}H NMR ($CDCl_3$, 400 MHz) δ: 7.32 – 7.26 (m, 6H), 7.22 – 7.16 (m, 6H), 6.85 – 6.81 (m, 2H), 4.12 (t, 1H, *J* = 7.8 Hz), 3.80 (s, 3H), 2.78 – 2.74 (m, 2H), 2.35 – 2.29 (m, 2H). **^{13}C NMR** (101 MHz, $CDCl_3$) δ: 158.9, 144.2, 133.2, 128.5, 127.9, 126.3, 114.6, 55.4, 49.9, 33.1, 34.0. **ATR-IR** υ_{max} (neat)/ cm^{-1} 3107, 3075, 3047, 2969, 2947, 2882, 2847, 1605, 1580, 1505, 1458, 1445, 1292, 1249, 1171, 1111, 1086, 1030, 921, 837, 777, 755, 727, 708, 696, 646, 634, 593, 559, 524. Mp = 48 - 51 °C. **HRMS** (ESI) calculated for ($C_{22}H_{22}OS$) $[M]^+$ 334.1386, found: 334.1387

(3-((4-methoxyphenyl)sulfinyl)propane-1,1-diyl)dibenzene 63

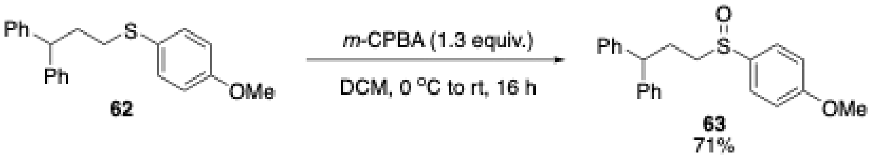

This experiment followed general procedure G using (3,3-diphenylpropyl)(4-methoxyphenyl)sulfane **62** (669 mg, 2 mmol, 1 equiv.), *m*-CPBA (448 mg, 2.6 mmol, 1.3 equiv.) and DCM (40 mL). Purification by chromatography (20% EtOAc in hexanes→70% EtOAc in hexanes) afforded (3-((4-methoxyphenyl)sulfinyl)propane-1,1-diyl)dibenzene **63** as a white solid (498 mg, 1.42 mmol, 71%). **Mp** 107 - 110 °C; **v_{max}** (neat)/cm^{-1}; 3110, 3075, 3038, 2979, 2950, 2919, 2860, 1608, 1583, 1505, 1470, 1449, 1314, 1252, 1186, 1096, 1043, 980, 918, 861, 827, 799, 762, 699, 634, 590, 555, 534, 512. **^{1}H NMR** ($CDCl_3$, 400 MHz) δ; 7.50 – 7.47 (m, 2H), 7.28 – 7.24 (m, 4H), 7.20 – 7.15 (m, 6H), 7.01 – 6.99 (m, 2H), 3.97 (t, 1H, *J* = 8.0 Hz), 3.85 (s, 3H), 2.72 (t, 2H, *J* = 7.0 Hz), 2.48 – 2.40 (m, 1H), 2.39 – 2.29 (m, 1H).**^{13}C NMR** (101 MHz, $CDCl_3$) 161.9, 143.5, 143.3, 134.5, 128.6, 127.8, 127.7, 126.6, 125.9, 114.7, 55.5, 55.3, 50.1, 27.8. δ ***m/z*** (ESI+) calculated for ($C_{22}H_{22}O_2NaS$) $[M+Na]^+$ 373.1232, found: 373.1225.

Synthesis of 3-(4-(*tert*-butyl)phenyl)propyl)(*p*-tolyl)sulfane 64

Step 1: 3-(4-(*tert*-butyl)phenyl)propyl methanesulfonate S51

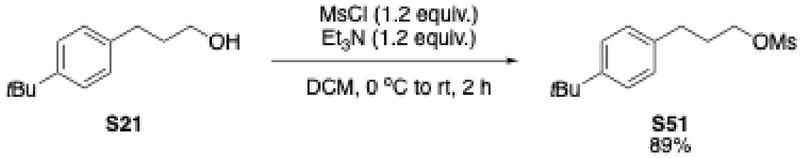

This experiment followed general procedure E using 3-(4-(*tert*-butyl)phenyl)propan-1-ol **S21** (962 mg, 5 mmol, 1 equiv.), methanesulfonyl chloride (0.54 mL, 6 mmol, 1.2 equiv.), triethylamine (0.9 mL, 6 mmol, 1.2 equiv.) and anhydrous DCM (30 mL), affording 3-(4-(*tert*-butyl)phenyl)propyl methanesulfonate **S51** as a beige solid (1.1 g, 4.1 mmol, 82%). **Mp** 52 – 55 °C; $\mathbf{v_{max}}$ (neat)/cm^{-1} 3055, 2975, 2925, 2882, 1521, 1481, 1461, 1361, 1340, 1186, 1084, 1007, 974, 927, 841, 804, 770, 721, 576, 536; **^{1}H NMR** ($CDCl_3$, 400 MHz) δ 7.33 – 7.30 (m, 2H), 7.13 – 7.10 (m, 2H), 4.23 (t, *J* = 6.3 Hz, 2H), 2.99 (s, 3H), 2.72 (t, *J* = 7.8 Hz, 2H), 2.10 – 2.03 (m, 2H), 1.31 (s, 9H); **^{13}C NMR** (101 MHz, $CDCl_3$) δ 148.7, 136.6, 127.6, 124.9, 68.7, 36.8, 33.9, 30.8, 30.4, 30.1; ***m/z*** (ESI+) calculated for $C_{16}H_{18}O_2NaS$ ($[M+Na]^-$): 313.0869, found 313.0870.

Step 2: 3-(4-(*tert*-butyl)phenyl)propyl)(*p*-tolyl)sulfane 64

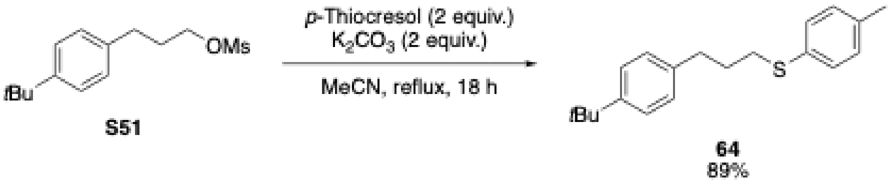

This experiment followed general procedure F using 3-(4-(*tert*-butyl)phenyl)propyl methanesulfonate **S51** (810 mg, 3 mmol, 1 equiv.), *p*-thiocresol (750 mg, 6 mmol, 2 equiv.), K_2CO_3 (820 mg, 6 mmol, 2 equiv.) and acetonitrile (40 mL). Purification by chromatography (100% hexanes→20% DCM in hexanes) afforded (3-(4-(*tert*-butyl)phenyl)propyl)(*p*-tolyl)sulfane **64** as a colourless oil (801 mg, 2.68 mmol, 89%). $\mathbf{v_{max}}$ (neat)/cm^{-1} 3021, 2958, 2867, 1495, 1386, 1271, 1111, 1094, 1020, 830, 741; **^{1}H NMR** ($CDCl_3$, 400 MHz) δ 7.34 – 7.31 (m, 2H), 7.27 – 7.25 (m, 2H), 7.14 – 7.10 (m, 4H), 2.92 (t, *J* = 7.4 Hz, 2H), 2.74 (t, *J* = 7.9 Hz, 2H), 2.34 (s, 3H), 1.96 (quint, *J* = 7.5 Hz, 2H), 1.34 (s, 9H); **^{13}C NMR** (101 MHz, $CDCl_3$) δ 148.2, 137.8, 135.5, 132.2, 129.5, 129.1, 127.6, 124.7, 33.8, 33.6, 33.2, 30.9, 30.2, 20.5; ***m/z*** (ESI+) calcd. for $C_{20}H_{27}{}^{32}S$ $[M+H]^+$ 299.1828, found 299.1829.

Preparation of 2-(3-(*p*-tolylsulfinyl)propyl)naphthalene 65

Step 1: 3-(Naphthalen-2-yl)propyl methanesulfonate S52

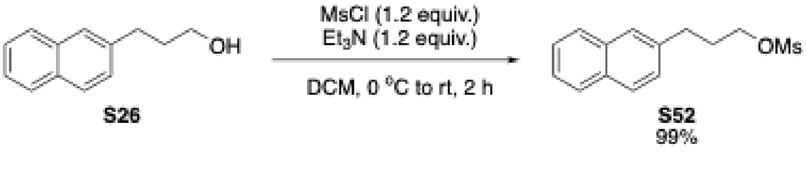

This experiment followed general procedure E using 3-(naphthalen-2-yl)propan-1-ol **S26** (1.58 g, 8.5 mmol, 1 equiv.), methanesulfonyl chloride (0.79 mL, 10.2 mmol, 1.2 equiv.), triethylamine (1.44 mL, 10.2 mmol, 1.2 equiv.) and anhydrous DCM (40 mL), affording 3-(naphthalen-2-yl)propyl methanesulfonate **S52** as a beige solid (2.22 g, 99%). **Mp** 60 – 63 °C; **ν_{max}** (neat)/cm^{-1} 3082, 3054, 2971, 2939, 2875, 1962, 1779, 1608, 1521, 1481, 1425, 1350, 1246, 1167, 1076, 1024, 972, 933, 897, 937, 786, 714, 650, 571, 531; **^{1}H NMR** ($CDCl_3$, 400 MHz) δ 7.82 – 7.77 (m, 3H), 7.63 (s, 1H), 7.49 – 7.24 (m, 2H), 7.33 (dd, *J* = 1.7, 8.4 Hz, 1H), 4.26 (t, *J* = 6.3 Hz, 2H), 2.98 (s, 3H), 2.92 (t, *J* = 7.8 Hz, 2H), 2.21 – 2.14 (m, 2H); **^{13}C NMR** (101 MHz, $CDCl_3$) δ 137.2, 133.0, 131.6, 127.7, 121.1, 126.9, 126.4, 126.2, 125.6, 124.9, 68.6, 36.8, 31.2, 30.0; ***m/z*** (EI) 264.1 (M^+, 26), 167.1 (100), 153.1 (61), 141.1 (83), 128.1 (23), 115.0 (44), 102.0 (3), 79.0 (23). Data were in agreement with corresponding data in the literature.[28]

Step 2: (3-(Naphthalen-2-yl)propyl)(*p*-tolyl)sulfane S53

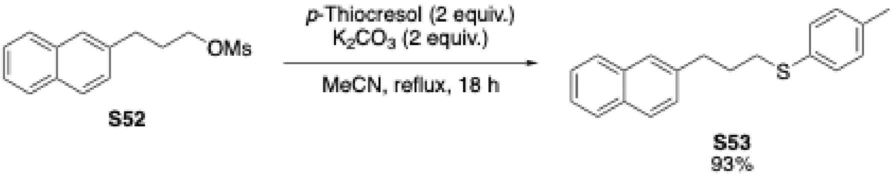

This experiment followed general procedure F using 3-(naphthalen-2-yl)propyl methanesulfonate **S52** (1.05 g, 4 mmol, 1 equiv.), *p*-thiocresol (0.99 g, 8 mmol, 2 equiv.), K_2CO_3 (1.11 g, 8 mmol, 2 equiv.) and acetonitrile (40 mL). Purification by chromatography (10% →20% DCM in petroleum ether) afforded (3-(naphthalen-2-yl)propyl)(*p*-tolyl)sulfane **S53** as a colourless oil (1.09 g, 93%). **ν_{max}** (neat)/cm^{-1} 3070, 3034, 2963, 2927, 2863, 1748, 1604, 1501, 1463, 1441, 1413, 1374, 1294, 1211, 1119, 1099, 1016, 972, 909, 869, 802, 746, 507; **^{1}H NMR** ($CDCl_3$, 400 MHz) δ 7.81 – 7.75 (m, 3H), 7.60 (s, 1H), 7.47 – 7.39 (m, 2H), 7.31 (dd, *J* = 1.6, 8.4 Hz,1H), 7.26 – 7.24 (m, 2H), 7.09 – 7.07 (m, 2H), 2.91 (t, *J* = 7.6 Hz, 4H), 2.32 (s, 3H), 2.03 (quint, *J* = 7.4 Hz, 2H); **^{13}C NMR** (101 MHz, $CDCl_3$) δ 138.3, 135.5, 133.0, 132.1, 131.5, 129.6, 129.1, 127.4, 127.1, 126.9, 126.7, 126.1, 125.4, 124.7, 34.2, 33.2, 30.0, 22.1; ***m/z*** (ESI+) calcd. for $C_{20}H_{21}{}^{32}S$ $[M+H]^+$ 293.1359, found 293.1359.

Step 3: 2-(3-(*p*-Tolylsulfinyl)propyl)naphthalene 65

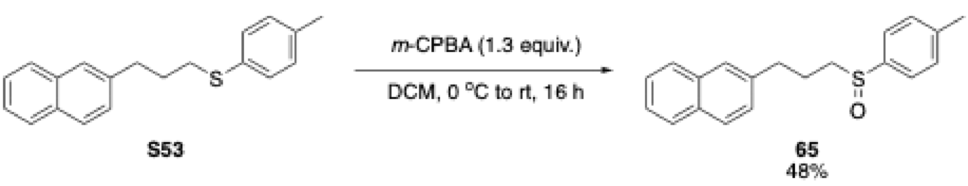

This experiment followed general procedure G using (3-(naphthalen-2-yl)propyl)(*p*-tolyl)sulfane **S53** (497 mg, 1.7 mmol, 1 equiv.), *m*-CPBA (380 mg, 2.2 mmol, 1.3 equiv.) and DCM (20 mL). Purification by chromatography (10% EtOAc in hexanes→50% EtOAc in hexanes) afforded 2-(3-(*p*-tolylsulfinyl)propyl)naphthalene **65** as a white solid (252 mg, 48%). **Mp** 101 – 103 °C; **ν_{max}** (neat)/cm^{-1} 3119, 3070, 3033, 2931, 1725, 1638, 1611, 1512, 1497, 1457, 1405, 1278, 1192, 1127, 1084, 1054, 1017, 866, 829, 810, 746, 706, 620, 512; **^{1}H NMR**

(CDCl$_3$, 400 MHz) δ 7.81 – 7.74 (m, 3H), 7.56 (s, 1H), 7.48 – 7.40 (m, 4H), 7.29 – 7.26 (m, 3H), 2.90 (t, *J* = 7.3 Hz, 2H), 2.80 (t, *J* = 7.5 Hz, 2H), 2.39 (s, 3H), 2.23 – 2.12 (m, 1H), 2.10 – 1.99 (m, 1H); **^{13}C NMR** (101 MHz, CDCl$_3$) δ 140.9, 140.0, 137.4, 133.0, 131.6, 129.4, 127.6, 127.1, 126.9, 126.4, 126.1, 125.5, 124.9, 123.5, 55.8, 34.1, 22.9, 20.9; ***m/z*** (ESI+) calcd. for $C_{20}H_{21}OS$ [M+H]$^+$ 309.1308, found 309.1307

9-(2-(Phenylthio)ethyl)-4a,9a-dihydro-9*H*-xanthene 66

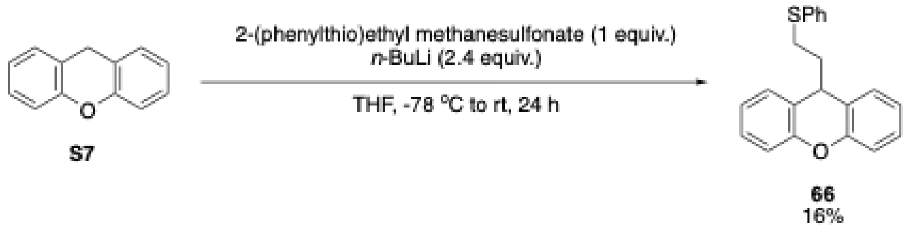

A solution on *n*BuLi (2.5 M in hexanes) (5.2 mL, 9.6 mmol, 2.4 equiv.) was added dropwise to a solution of xanthene **S7** (720 mg, 4 mmol, 1 equiv.) in anhydrous THF at -78 °C under an atmosphere of argon and the mixture allowed to stir. A solution of 2-(phenylthio)ethyl methanesulfonate (930 mg, 4 mmol, 1 equiv.) in anhydrous THF was transferred dropwise to the solution containing the deprotonated diarylmethane and the mixture stirred for 24h. The reaction was quenched with water (10 mL) and diluted further with water (50 mL), then extracted into EtOAc (3 x 50 mL). The combined organics were dried over $MgSO_4$, filtered, and concentrated *in vacuo* . The crude was then purified via column chromatography to afford the desired product. Purification by chromatography (100% hexanes→20% DCM in hexanes) afforded 9-(2-(phenylthio)ethyl)-4a,9a-dihydro-9*H*-xanthene **66** as a yellow oil (210 mg, 16%). **v_{max}** (neat)/cm^{-1} 3073, 3008, 2964, 1633, 1460, 1430, 1352, 1198, 1080, 895, ,766, 557; **^{1}H NMR** (CDCl$_3$, 400 MHz) δ 7.24 – 7.18 (m, 6H), 7.15 – 7.05 (m, 7H), 4.20 (t, *J* = 5.9 Hz, 1H), 2.76 – 2.72 (m, 2H), 2.06 – 2.01 (m, 2H); **^{13}C NMR** (101 MHz, CDCl$_3$) δ 151.7, 128.3, 128.1, 128.0, 127.3, 125.2, 123.8, 122.8, 116.0, 38.7, 37.2, 28.5; ***m/z*** (ESI+) calcd. for $C_{21}H_{19}OS$ [M+H]$^+$ 319.1151, found 319.1146.

Cyclopentane and Cyclopropane ring formations

Reaction of *N*,2-dimethyl-*N*-phenylaniline 11 in THF

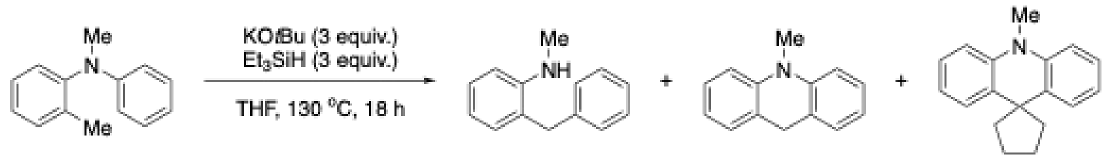

11 **12** 10% (30% NMR yield) **13** 14% (52% NMR yield) **14** 4% (12% NMR yield)

This experiment was carried out according to general procedure I with *N*,2-dimethyl-*N*-phenylaniline **11** (99 mg, 0.5 mmol, 1 equiv.), KO*t*Bu (168 mg, 1.5 mmol, 3 equiv.), and Et_3SiH (240 µL, 1.5 mmol, 3 equiv.) in anhydrous THF (5 mL). Analysis by NMR showed the presence of **12** (30%),**13** (52%) and **14** (12%). Purification using column chromatography (50% hexanes in toluene → 100% toluene) afforded 10-methyl-9,10-dihydroacridine **13** as a yellow oil (15.4 mg, 14%), 2-benzyl-*N*-methylaniline **12** as a yellow oil (10.8 mg, 10%), and 10-methyl-10*H*-spiro[acridine-9,1'-cyclopentane] **14** (5.0 mg, 4%) as a yellow oil. Analytical data for **13** corresponded with the data reported for its synthesis. **12** $\mathbf{v_{max}}$ (neat)/cm^{-1} 3431, 2893, 1604, 1512, 1307, 1161, 729; **^{1}H NMR** (400 MHz, $CDCl_3$) δ 7.32 – 7.26 (m, 2H), 7.24 – 7.18 (m, 2H), 7.16 (d, *J* = 7.3 Hz, 2H), 7.05 – 7.00 (d, *J* = 7.1 Hz, 1H), 6.77 (t, *J* = 7.4 Hz, 1H), 6.65 (d, *J* = 8.1 Hz, 1H), 3.87 (s, 2H), 3.53 (bs, 1H), 2.77 (s, 3H); **^{13}C NMR** (101 MHz, $CDCl_3$) δ 147.3, 139.4, 130.6, 128.8, 128.6, 128.0, 126.5, 124.7, 117.1, 110.1, 38.0, 30.9; ***m/z*** (ESI+): calcd. for $C_{14}H_{16}N$ $[M+H]^+$ 198.1277, found 198.1277. NMR data are in agreement with those reported in the literature.[29] **14** $\mathbf{v_{max}}$ (neat)/cm^{-1} 3065, 3032, 2997, 2870, 2853, 2812, 1503, 1450, 1342, 1314, 1292, 1229, 1196, 1169, 1098, 1053, 1015, 964; **^{1}H NMR** (400 MHz, $CDCl_3$) δ 7.37 (dd, *J* = 7.7, 1.4 Hz, 2H), 7.30 – 7.19 (m, 2H), 7.06 – 6.94 (m, 4H), 3.46 (s, 3H), 2.17 – 2.05 (m, 4H), 1.89 – 1.78 (m, 4H); **^{13}C NMR** (101 MHz, $CDCl_3$) δ 143.0, 132.2, 126.5, 123.9, 120.5, 112.2, 48.9, 36.5, 33.6, 24.2; ***m/z*** (ESI+) calcd. for $C_{18}H_{19}NNa$ $[M+Na]^+$ 272.1407, found: 272.1410.

Reaction of *N*,2-dimethyl-*N*-phenylaniline 11 in *d*$_8$-THF

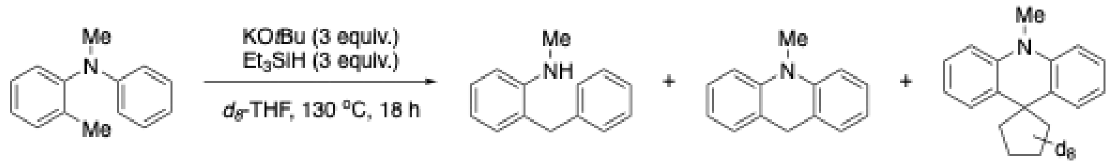

11 **12** 21% **13** [26%] **d$_8$-14** [13%]

The reaction was carried out according to general procedure I with *N*,2-dimethyl-*N*-phenylaniline **11** (99 mg, 0.5 mmol, 1 equiv.), KO*t*Bu (168 mg, 1.5 mmol, 3 equiv.), and Et_3SiH (240 µL, 1.5 mmol, 3 equiv.) in anhydrous *d*$_8$-THF (5 mL). Purification using column chromatography (50% hexanes in toluene → 100% toluene) afforded 2-benzyl-*N*-methylaniline **12** as a yellow oil (22.4 mg, 21%) and a mixture (45.5 mg) of 10-methyl-9,10-dihydroacridine **13** (27.8 mg, 26%) and 10-methyl-10*H*-spiro[acridine-9,1'-cyclopentane]-

2',2',3',3',4',4',5',5'-d_8 d_8-**14** (17.7 mg, 13%) with analytical data for **13** and **12** consistent with the data outlined above for these compounds. Preparative TLC (100% toluene) of the mixture of **13** and d_8-**14** afforded a clean sample of d_8-**14** **v_{max}** (neat)/cm^{-1}; 3030, 1589, 1463, 1452, 1344, 1269,1132, 1059; **^{1}H NMR** (400 MHz, $CDCl_3$) δ 7.33 (dd, *J* = 7.6, 1.2 Hz, 2H), 7.25 – 7.19 (m, 2H), 7.00 – 6.92 (m, 4H), 3.44 (s, 3H); **^{2}H NMR** (76 MHz, $CHCl_3$, with $CDCl_3$ as internal standard): δ 2.06 (s, 4H), 1.76 (s, 4H); **^{13}C NMR** (101 MHz, $CDCl_3$) δ 143.1, 132.2, 126.5, 123.9, 120.5, 112.2, 33.6; ***m/z*** (APCI+) calcd. for $C_{18}H_{12}{}^2H_8N$ $[M+H]^+$ 258.2092, found 258.2089.

When the experiment was repeated, but with 1:1 mixture of THF and THF-d_8, the product **14** was a mixture of d_0 and d_8 isotopologues. No H/D exchange was seen.

Reaction of 10-methyl-9,10-dihydroacridine 13

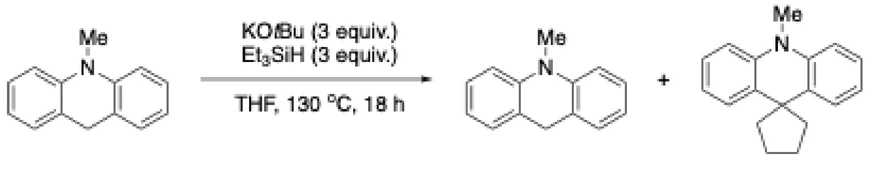

13 **13** [36%] **14** [12%]

The reaction was carried out according to general procedure I with 10-methyl-9,10-dihydroacridine (99 mg, 0.5 mmol, 1 equiv.), Et_3SiH (3.0 equiv., 1.5 mmol, 240 μL), KO^{t}Bu (3.0 equiv., 1.5 mmol, 168 mg) and anhydrous THF (5 mL). The crude mixture was purified by column chromatography (hexane[illegible]20% toluene/80% hexane) affording a mixture (51 mg) of 10-methyl-9,10-dihydroacridine **13** (35.4 mg, 36%) and 10-methyl-10*H*-spiro[acridine-9,1'-cyclopentane] **14** (15.6 mg, 12%) (2.88:1 ratio by ^{1}H NMR). 10,10'-Dimethyl-9,9',10,10'-tetrahydro-9,9'-biacridine **15** (RT 19.93 min, *m/z* 388.4) was detected by GC-MS. Analytical data for compounds **13** and **14** is in agreement with the data reported above. Full characterisation of compound **15** is available in the data reported below, after it was isolated.

Reaction of 10-methyl-9,10-dihydroacridine 13 in 2-methyltetrahydrofuran

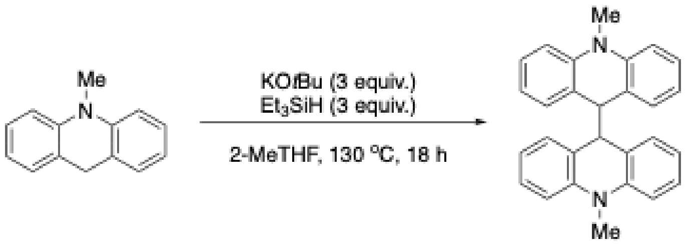

13 **15** 64%

The reaction followed general procedure I using 10-methyl-9,10-dihydroacridine **13** (99 mg, 0.5 mmol, 1 equiv.), Et_3SiH (240 μL, 1.5 mmol, 3 equiv.), KO*t*Bu (168 mg, 1.5 mmol, 3 equiv.) and anhydrous 2-Me-THF (5 mL). The crude reaction mixture was recrystallised from DCM/cold MeCN and the resulting yellow solid was washed with hexane. 10,10'-Dimethyl-9,9',10,10'-tetrahydro-9,9'-biacridine **15** (62 mg, 64%) was isolated as a yellow solid. **Mp** 259

- 261 °C (decomp.) (lit. mp. 261-263 °C)[30]; **v_{max}** (neat)/cm^{-1} 3065, 3017, 2899, 2822, 1591, 1464, 1458, 1423, 1412, 1341, 1310, 1279, 1261, 1213, 1186, 1157, 1130, 1101, 1086, 1063, 1042, 930, 883, 854, 814, 741, 698, 675, 635; **^{1}H NMR** (400 MHz, $CDCl_3$) δ 7.20 – 7.07 (m, 4H), 6.76 – 6.62 (m, 8H), 6.48 (dd, *J* = 7.4, 1.5 Hz, 4H), 3.92 (s, 2H), 3.01 (s, 6H); **^{13}C NMR** (101 MHz, $CDCl_3$) δ 143.1, 129.2, 127.1, 124.7, 119.7, 111.5, 52.1, 33.0. ***m/z*** (ESI+) 389.2 ([M+H]$^+$). Analytical data were consistent with those previously reported in the literature.[31]

Reaction of 10-methyl-9,10-dihydroacridine 13 in tetrahydropyran

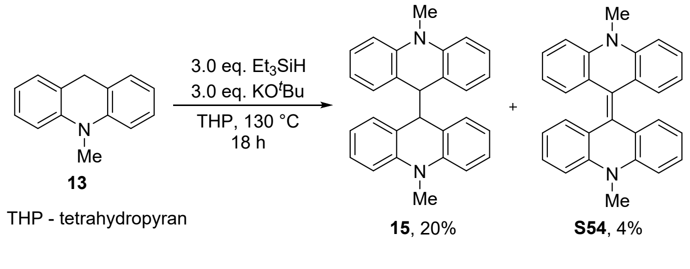

Carried out according to **General Procedure I** using 10-methyl-9,10-dihydroacridine **13** (1.0 eq., 0.5 mmol, 98.6 mg), Et_3SiH (3.0 eq., 1.5 mmol, 240 µL), KO*t*Bu (3.0 eq., 1.5 mmol, 168 mg) and dry THP (5 mL). The crude reaction mixture was recrystallised from CH_2Cl_2/cold MeCN and the resulting yellow solid was washed with cold MeCN and hexane. A mixture of 10,10'-dimethyl-9,9',10,10'-tetrahydro-9,9'-biacridine **15** (19 mg, 20%) and 10,10'-dimethyl-10*H*,10'*H*-9,9'-biacridinylidene **S54** (3 mg, 3%) was isolated as a yellow solid. Some yellow solid residue was observed on the walls of the phase separator used for the filtration of the crude mixture in Et_2O after work-up. The yellow solid residue was dissolved in CH_2Cl_2 and concentrated, affording clean 10,10'-dimethyl-10*H*,10'*H*-9,9'-biacridinylidene **S54** (3.8 mg, 4%) as a yellow solid. **^{1}H NMR** (400 MHz, $CDCl_3$) δ 7.21 – 7.13 (m, 4H, 4 x ArH), 7.05 – 6.98 (m, 8H, 8 x ArH), 6.80 – 6.67 (m, 4H, 4 x ArH), 3.53 (s, 6H, 2 x NMe). **^{13}C NMR** (101 MHz, $CDCl_3$) δ 145.3, 136.0, 128.4, 127.2, 124.8, 120.2, 112.6, 33.5. **ATR-IR** v_{max} (neat)/cm^{-1} 3073, 3024, 2995, 2926, 2882, 1607, 1582, 1551, 1499, 1452, 1422, 1389, 1377, 1339, 1288, 1263, 1231, 1194, 1173, 1103, 1032, 934, 885, 866, 760, 745, 704, 696, 669, 638, 617, 600. **HRMS** (ESI) calcd for $C_{28}H_{22}N_2^+$ (M$^+$): 386.1783, found: 386.1764.

Reaction of 5,5-diphenylpentan-1-ol 25 (Table 1, entry 1)

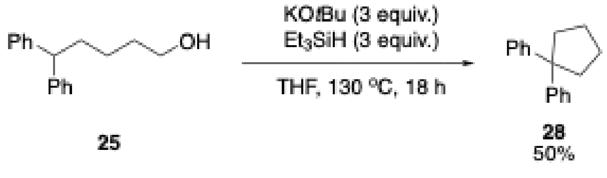

The reaction followed general procedure I using 5,5-diphenylpentan-1-ol **25** (120 mg, 0.5 mmol, 1 equiv.), Et_3SiH (240 µL, 1.5 mmol, 3 equiv.), KO*t*Bu (168 mg, 1.5 mmol, 4 equiv) and anhydrous THF (5 mL). Purification by chromatography (100% hexanes) afforded cyclopentane-1,1-diyldibenzene **28** (56 mg, 50%) as a white solid. **Mp** 60-63 °C (lit. mp. 72-72.5 °C)[32]; **v_{max}** (neat)/cm^{-1} 3082, 3017, 2998, 2963, 2947, 2913, 2866, 1593, 1489, 1474, 1450, 1379, 1335, 1321, 1304, 1244, 1233, 1200, 1157, 1126, 1061, 1030, 1001, 980, 961, 949, 907, 862, 833, 772, 745, 691, 646, 611; **^{1}H NMR** (400 MHz, $CDCl_3$) δ 7.34 – 7.20 (m, 8H), 7.18 – 7.09 (m, 2H), 2.45 – 2.20 (m, 4H), 1.85 – 1.61 (m, 4H); **^{13}C NMR** (101 MHz, $CDCl_3$)

δ 149.1, 128.2, 127.2, 125.6, 56.0, 38.7, 23.1; ***m/z*** (EI+) calcd. for $C_{17}H_{18}^+$ [M^+] 222.1409, found: 222.1400.

Reaction of ((5,5-diphenylpentyl)oxy)triethylsilane 26 (Table 1, entry 2)

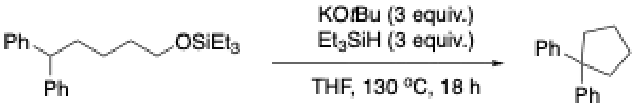

26 **28** 77%

The reaction followed general procedure I using ((5,5-diphenylpentyl)oxy)triethylsilane **26** (177 mg, 0.5 mmol, 1 equiv.), Et_3SiH (240 μL, 1.5 mmol, 3 equiv.), KO*t*Bu (168 mg, 1.5 mmol, 3 equiv.) and anhydrous THF (5 mL). Purification by chromatography (100% hexanes) afforded cyclopentane-1,1-diyldibenzene **28** (80 mg, 77%) as a white solid with analytical data consistent with the data outlined above.

Reaction of (5-methoxypentane-1,1-diyl)dibenzene 27 (Table 1, entry 3)

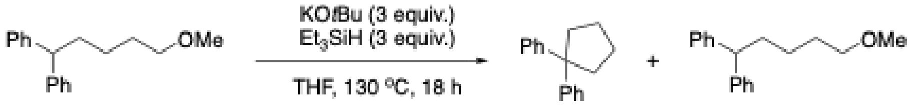

27 **28** 27% **27** 56%

The reaction followed general procedure I using (5-methoxypentane-1,1-diyl)dibenzene **27** (1.0 equiv., 0.5 mmol, 127 mg), Et_3SiH (240 μL, 1.5 mmol, 3 equiv.), KO*t*Bu (168 mg, 1.5 mmol, 3 equiv.) and anhydrous THF (5 mL). Purification by column chromatography (100% hexanes→30% EtOAc in hexanes) afforded (5-methoxypentane-1,1-diyl)dibenzene starting material **27** (71 mg, 56%) as a yellow oil and cyclopentane-1,1-diyldibenzene **28** (30 mg, 27%) as a white solid with analytical data consistent with the data outlined above.

Reaction of 5,5-diphenylpentan-1-ol 25 (Table 1, entry 4)

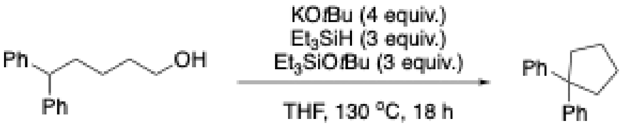

25 **28** 17%

The reaction followed general procedure I using 5,5-diphenylpentan-1-ol **25** (120 mg, 0.5 mmol, 1 equiv.), Et_3SiH (240 μL, 1.5 mmol, 3 equiv.), KO*t*Bu (224 mg, 2 mmol, 4 equiv.) and anhydrous THF (5 mL). $Et_3SiOtBu$ (283 mg, 1.5 mmol, 3 equiv.) was used as an additive. Purification by chromatography (100% hexanes→2% EtOAc in hexanes) afforded cyclopentane-1,1-diyldibenzene **28** (19 mg, 17%) as a white solid, with analytical data consistent with the data above.

Reaction of ((5,5-diphenylpentyl)oxy)triethylsilane 26 (Table 1, entry 5)

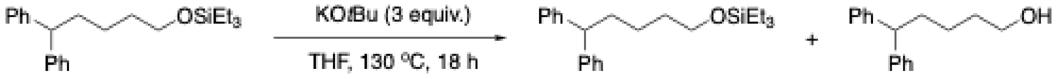

26 — 28 36% — 25 50%

The reaction followed general procedure I using ((5,5-diphenylpentyl)oxy)triethylsilane **26** (177 mg, 0.5 mmol, 1 equiv.), KO*t*Bu (168 mg, 1.5 mmol, 3 equiv.) and anhydrous THF (5 mL). Purification by chromatography (100% hexane→3% EtOAc in hexanes) afforded ((5,5-diphenylpentyl)oxy)triethylsilane starting material **26** (63 mg, 36%) as a yellow oil and 5,5-diphenylpentan-1-ol **25** (60 mg, 50%) as a yellow oil with analytical data consistent with the data outlined above for these compounds.

Reaction of 5,5-diphenylpentan-1-ol 25 with NaO*t*Bu (Table 1, entry 6)

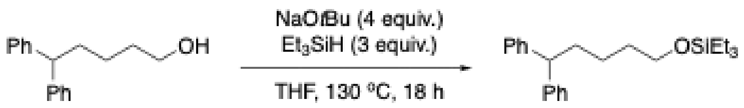

25 — 26 3%

The reaction followed general procedure I using 5,5-diphenylpentan-1-ol **25** (122 mg, 0.5 mmol, 1 equiv.), NaO*t*Bu (192 mg, 2 mmol, 4 equiv.) and Et_3SiH (240 µL, 1.50 mmol, 3 equiv.) in anhydrous THF (5 mL). Purification by column chromatography (100% hexanes→3% EtOAc in hexanes) afforded ((5,5-diphenylpentyl)oxy)triethylsilane **26** (5.2 mg, 3%) as a yellow oil with analytical data consistent with the data outlined above.

Reaction of 5,5-diphenylpentan-1-ol 25 with LiO*t*Bu (Table 1, entry 7)

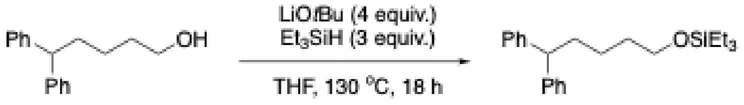

25 — 26 76%

The reaction followed a modified general procedure I using 5,5-diphenylpentan-1-ol **25** (122 mg, 0.5 mmol, 1 equiv.), LiO*t*Bu (160 mg, 2 mmol, 4 equiv.) and Et_3SiH (240 μL, 1.50 mmol, 3 equiv.) in anhydrous THF (5 mL). Purification by column chromatography (100% hexanes→3% EtOAc in hexanes) afforded ((5,5-diphenylpentyl)oxy)triethylsilane **26** (137 mg, 76%) as a yellow oil with analytical data consistent with the data outlined above.

Reaction of 5,5-diphenylpentan-1-ol 25 with NaH + Et_3SiH (Table 1, entry 8)

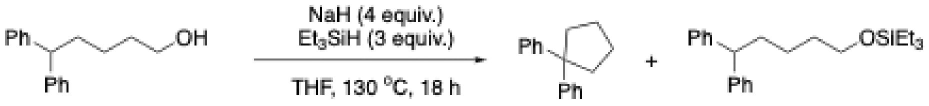

25 28 26
26% 36%

The reaction followed a modified general procedure H using 5,5-diphenylpentan-1-ol **25** (122 mg, 0.5 mmol, 1 equiv.), NaH (48 mg, 2 mmol, 4 equiv.) and Et_3SiH (240 μL, 1.5 mmol, 3 equiv.) in anhydrous THF (5 mL). Purification by column chromatography (100% hexanes→3% EtOAc in hexanes) afforded cyclopentane-1,1-diyldibenzene **26** (29 mg, 26%) as a white solid and ((5,5-diphenylpentyl)oxy)triethylsilane **26** (64.4 mg, 36%) as a yellow oil with analytical data for both consistent with the data outlined above.

Reaction of 5,5-diphenylpentan-1-ol 25 with KH + Et_3SiH (Table 1, entry 9)

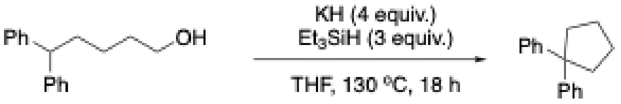

25 28
67%

The reaction followed general procedure I using 5,5-diphenylpentan-1-ol **25** (122 mg, 0.5 mmol, 1 equiv.), KH (80 mg, 2 mmol, 4 equiv.) and Et_3SiH (240 μL, 1.5 mmol, 3 equiv.) in anhydrous THF (5 mL). Purification by chromatography (100% hexanes) afforded cyclopentane-1,1-diyldibenzene **28** (77 mg, 67%) as white solid, analytical data consistent with the data outlined above.

Reaction of 5,5-diphenylpentan-1-ol 25 with 2-methyltetrahydrofuran (Table 1, entry 10)

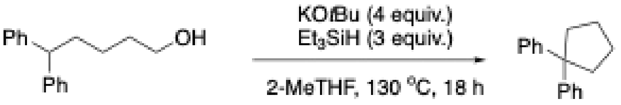

25 28
49%

The reaction followed using 5,5-diphenylpentan-1-ol **25** (122 mg, 0.5 mmol, 1 equiv.), KO*t*Bu (224 mg, 2 mmol, 4 equiv.) and Et_3SiH (240 μL, 1.5 mmol, 3 equiv.) in anhydrous 2-methyl-THF (5 mL). Purification by chromatography (100% hexanes) afforded cyclopentane-1,1-diyldibenzene **28** (54 mg, 49%) as white solid, analytical data consistent with the data outlined above.

Reaction of 10-methyl-9,10-dihydroacridine 13 with *n*BuLi

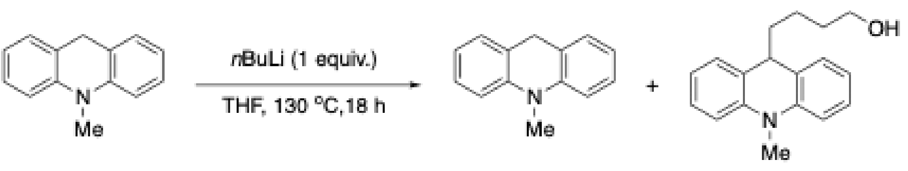

13 13 29
33% 34%

Under an atmosphere of argon, 10-methyl-9,10-dihydroacridine **13** (98 mg, 0.5 mmol, 1 equiv.) was dissolved in anhydrous THF (5 mL) in an oven-dried Schlenk tube, followed by the addition of *n*BuLi (0.2 mL, 2.5 M, 1 equiv.), affording a deep red solution. The mixture was refluxed at 130 °C for 18 h and had a distinct blue colour. After reaction, the mixture was quenched with H_2O (15 mL) and extracted with DCM (2 x 20 mL). The combined organics were dried over Na_2SO_4 and concentrated *in vacuo*. The crude mixture was purified by chromatography (100% hexanes→50% EtOAc in hexanes) affording 10-methyl-9,10-dihydroacridine **13** starting material (32.9 mg, 33%) and 4-(10-methyl-9,10-dihydroacridin-9-yl)butan-1-ol **29** (45.7 mg, 34%). 10-Methyl-9,10-dihydroacridine **13** analytical data were consistent with the data outlined above for this compound **29**. $\mathbf{v_{max}}$ (neat)/cm^{-1} 2932, 2860, 1593, 1473, 1342, 1265, 1130, 1041; ^{1}**H NMR** (400 MHz, $CDCl_3$) δ 7.25 – 7.20 (m, 2H), 7.15 (dd, *J* = 7.4, 1.6 Hz, 2H), 6.98 – 6.91 (m, 4H), 3.83 (t, *J* = 7.3 Hz, 1H), 3.56 (t, *J* = 6.5 Hz, 2H), 3.39 (s, 3H), 1.61 – 1.52 (m, 2H), 1.52 – 1.43 (m, 2H), 1.36 – 1.25 (m, 2H), 1.21 (s, 1H); ^{13}**C NMR** (101 MHz, $CDCl_3$) δ 142.5, 128.3, 128.0, 126.9, 120.6, 112.2, 63.0, 44.4, 37.6, 33.0, 32.8, 23.1; ***m/z*** (APCI+) calcd. for $C_{18}H_{22}NO$ $[M+H]^+$ 268.1696, found 268.1689.

Reaction of *N*,2-dimethyl-*N*-phenylaniline 11 with KH

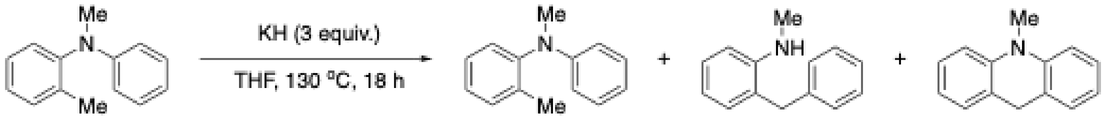

11 **11** 14% **12** 7% **13** 18%

The reaction followed general procedure J with *N*,2-dimethyl-*N*-phenylaniline **11** (99 mg, 0.5 mmol, 1 equiv.) and KH (60 mg, 1.5 mmol, 3 equiv.) in anhydrous THF (5 mL). Purification by chromatography (100% hexanes→50% toluene in hexanes) afforded *N*,2-dimethyl-*N*-phenylaniline starting material **11** (14.3 mg, 14%), 10-methyl-9,10-dihydroacridine **13** (18.4 mg, 18%), and 2-benzyl-*N*-methylaniline **12** (7.6 mg, 7%), analytical data for all compounds were consistent with the data outlined above.

Reaction of 4-(9*H*-xanthen-9-yl)butan-1-ol 30

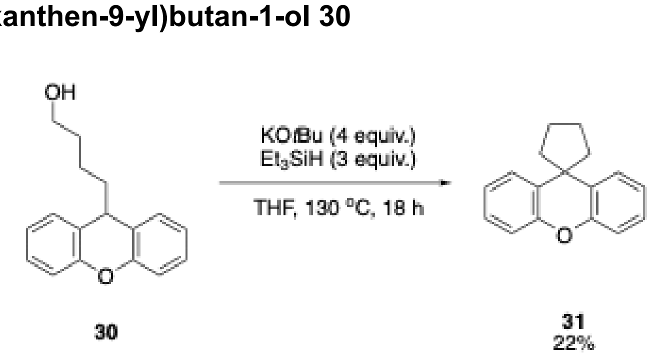

30 **31** 22%

The reaction followed general procedure I using 4-(9*H*-xanthen-9-yl)butan-1-ol **30** (127 mg, 0.5 mmol, 1 equiv.), KO*t*Bu (224 mg, 2 mmol, 4 equiv.) and Et_3SiH (240 μL, 1.5 mmol, 3 equiv.) in anhydrous THF (5 mL). Purification by chromatography (100% hexanes→30% EtOAc in hexanes) afforded spiro[cyclopentane-1,9'-xanthene] **31** (23 mg, 22%) as a yellow oil. $\mathbf{v_{max}}$

(neat)/cm^{-1} 3065, 3032, 2951, 2870, 1597, 1572, 1477, 1441, 1389, 1323, 1300, 1279, 1254, 1215, 1155, 1096, 1076, 1040, 953, 883, 858, 806, 746, 617; **^{1}H NMR** (400 MHz, $CDCl_3$) δ 7.35 (dd, *J* = 7.8, 1.5 Hz, 2H), 7.23 – 7.14 (m, 2H), 7.11 – 7.02 (m, 4H), 2.24 – 2.15 (m, 4H), 2.05 – 1.91 (m, 4H); **^{13}C NMR** (101 MHz, $CDCl_3$) δ 151.0, 131.8, 127.2, 126.3, 123.2, 116.3, 45.4, 45.1, 27.0; ***m/z*** (EI+) calcd. for $C_{17}H_{16}O^+$ [M^+] 236.1201, found: 236.1203.

Reaction of 4-(10-methyl-9,10-dihydroacridin-9-yl)butan-1-ol 29

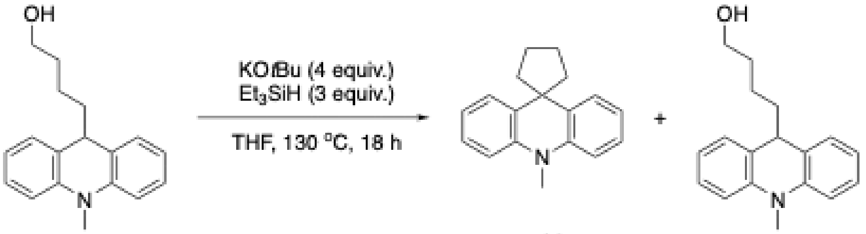

29 **14** 33% **29** 40%

This reaction was carried out according to general procedure I using 4-(10-methyl-9,10-dihydroacridin-9-yl)butan-1-ol **29** (134 mg, 0.5 mmol, 1 equiv.), KO*t*Bu (224 mg, 2 mmol, 4 equiv.), and Et_3SiH (240 µL, 1.5 mmol, 3 equiv.) in anhydrous THF (5 mL). Purification by column chromatography (hexane→3% EtOAc in hexanes) afforded the 4-(10-methyl-9,10-dihydroacridin-9-yl)butan-1-ol starting material **29** (53 mg, 40%) as a brown oil with analytical data consistent with the data outlined above. Impure 10-methyl-10*H*-spiro[acridine-9,1'-cyclopentane] **14** was also isolated as a red solid, which was recrystallised from cold hexane affording a clean crop of product **14** (26 mg). The mother liquor resulting obtained after recrystallisation was further purified by column chromatography (100% hexanes→5% DCM in hexanes) affording a second crop of pure product **14** (15 mg) as a white solid. Both crops of the 10-methyl-10*H*-spiro[acridine-9,1'-cyclopentane] product **14** (41 mg, 33%) were combined. Analytical data for **14** were consistent with the data outlined above, and a melting point was obtained. **Mp** 89-91 °C (no lit. mp).

Reaction of 2-benzylpyridine 32 in 1,4-dioxane

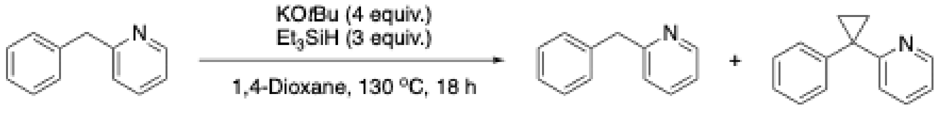

32 **32** 80% **34** 20%

This reaction was carried out according to general procedure I using 2-benzylpyridine **32** (80 µL, 0.5 mmol, 1 equiv.), Et_3SiH (240 µL, 1.5 mmol, 3 equiv.), KO*t*Bu (168 mg 1.5 mmol, 3 equiv.) and anhydrous 1,4-dioxane (5 mL). Purification by column chromatography (hexane→3% EtOAc in hexanes) afforded 2-(1-phenylcyclopropyl)pyridine **34** (20 mg, 20%) as a yellow oil and 2-benzylpyridine starting material **30** (68 mg, 80%) as a yellow oil. 2-(1-Phenylcyclopropyl)pyridine **34** $\mathbf{v_{max}}$ (neat)/cm^{-1} 3080, 3057, 3024, 3003, 1584, 1568, 1555, 1495, 1470, 1443, 1427, 1408, 1389, 1337, 1300, 1281, 1236, 1198, 1175, 1150, 1142, 1101, 1076, 1059, 1049, 1024, 989, 962, 935, 908, 895, 872, 845, 829, 808, 775, 760, 745, 700, 658, 644, 623; **^{1}H NMR** (400 MHz, $CDCl_3$) δ 8.50 (ddd, *J* = 4.8, 1.8, 0.9 Hz, 1H), 7.46 – 7.38

(m, 3H), 7.38 – 7.32 (m, 2H), 7.32 – 7.26 (m, 1H), 7.00 (ddd, *J* = 7.4, 4.8, 1.1 Hz, 1H), 6.83 (dt, *J* = 8.0, 1.0 Hz, 1H), 1.67 (dd, *J* = 6.5, 3.8 Hz, 2H), 1.32 (dd, *J* = 6.5, 3.8 Hz, 2H); **^{13}C NMR** (101 MHz, $CDCl_3$) δ 164.5, 149.1, 143.7, 135.8, 130.9, 128.7, 127.0, 122.3, 120.3, 31.6, 17.6; ***m/z*** (ESI+) calcd. for $C_{14}H_{14}N^+$ $[M+H]^+$ 196.1121, found: 196.1116.

2-Benzylpyridine **32** $\mathbf{v_{max}}$ (neat)/cm^{-1} 3082, 3059, 3026, 3005, 2918, 1587, 1568, 1495, 1474, 1452, 1433, 1304, 1281, 1244, 1221, 1148, 1092, 1072, 1049, 1030, 993, 962, 937, 887, 824, 808, 752, 696, 669, 631, 610; **^{1}H NMR** (400 MHz, $CDCl_3$) δ 8.64 – 8.51 (m, 1H), 7.57 (td, *J* = 7.7, 1.8 Hz, 1H), 7.36 – 7.25 (m, 4H), 7.25 – 7.18 (m, 1H), 7.15 – 7.03 (m, 2H), 4.17 (s, 2H); **^{13}C NMR** (101 MHz, $CDCl_3$) δ 161.1, 149.5, 139.6, 136.6, 129.2, 128.7, 126.5, 123.2, 121.3, 44.9; ***m/z* (EI):** 168.1 [$(M-1)^+$, 100%], 154.1 (1), 139.1 (5), 115.1 (5), 102.1 (1), 91.1 (9), 83.6 (5), 78.1 (7), 65.1 (12), 51.1 (15). Analytical data are consistent with those previously reported in the literature.[33]

Reaction of 4-benzylpyridine 33 in 1,4-dioxane

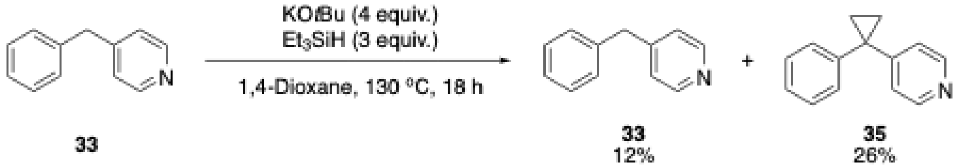

This reaction was carried out according to general procedure I using 4-benzylpyridine **33** (80 µL, 0.5 mmol, 1 equiv.), Et_3SiH (240 µL, 1.5 mmol, 3 equiv.), KO*t*Bu (168 mg, 1.5 mmol, 3 equiv.) and anhydrous 1,4-dioxane (5 mL). Purification by chromatography (100% hexane→15% EtOAc in hexane) afforded 4-(1-phenylcyclopropyl)pyridine **35** (25 mg, 26%) as a yellow oil and 4-benzylpyridine **33** (10 mg, 12%) as a yellow oil.

35 $\mathbf{v_{max}}$ (neat)/cm^{-1} 3080, 3057, 3022, 3007, 1593, 1545, 1495, 1458, 1445, 1410, 1389, 1342, 1314, 1287, 1252, 1223, 1177, 1155, 1132, 1099, 1069, 1024, 991, 961, 935, 910, 866, 810, 760, 745, 700, 615; **^{1}H NMR** (400 MHz, $CDCl_3$) δ 8.39 (d, *J* = 5.5 Hz, 2H), 7.41 – 7.19 (m, 5H), 7.00 – 6.87 (m, 2H), 1.45 – 1.36 (m, 2H), 1.36 – 1.29 (m, 2H) **^{13}C NMR** (101 MHz, $CDCl_3$) δ 155.5, 149.7, 143.2, 129.9, 128.7, 127.1, 121.9, 29.2, 17.8; ***m/z*** (EI) 195.1 (M^+, 78%), 194.1 (100), 180.1 (32), 167.1 (40), 152.1 (16), 139.1 (25), 128.1 (5), 115.1 (34), 102.1 (5), 91.1 (15), 77.0 (10), 63.0 (16), 51.1 (25). Analytical data are consistent with those previously reported in the literature.[34]

33 $\mathbf{v_{max}}$ (neat)/cm^{-1} 3065, 3026, 2920, 2849, 1665, 1595, 1558, 1495, 1449, 1414, 1323, 1279, 1217, 1180, 1153, 1069, 1028, 993, 841, 810, 785, 741, 700, 638, 613; **^{1}H NMR** (400 MHz, $CDCl_3$) δ 8.54 (br s, 2H), 7.35 – 7.28 (m, 2H), 7.26 – 7.21 (m, 1H), 7.20 – 7.15 (m, 2H), 7.12 (br s, 2H), 3.97 (s, 2H); **^{13}C NMR** (101 MHz, $CDCl_3$) δ 150.2, 149.9, 139.0, 129.2, 128.9, 126.8, 124.5, 41.4; ***m/z* (EI)**: 169.1 (M^+, 100%), 154.1 (3), 141.1 (12), 115.1 (22), 102.1 (3), 91.0 (39), 83.5 (5), 77.0 (7), 65.0 (23), 51.1 (36). Analytical data are consistent with those previously reported in the literature.[35]

Reaction of 3,3-diphenylpropan-1-ol 36 in THF

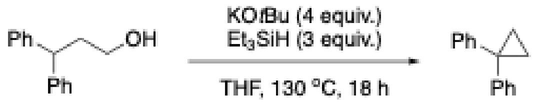

36 38 89%

This reaction was carried out according to general procedure I with 3,3-diphenylpropan-1-ol **36** (106 mg, 0.5 mmol, 1 equiv.), Et_3SiH (240 µL, 1.5 mmol, 3 equiv.), KO*t*Bu (224 mg, 2 mmol, 4 equiv.) and anhydrous THF (5 mL). Purification by chromatography (100% hexanes) afforded cyclopropane-1,1-diyldibenzene **38** (86 mg, 89%) as a colourless oil. $\mathbf{v_{max}}$ (neat)/cm^{-1} 3080, 3057, 3024, 3005, 1599, 1578, 1495, 1458, 1445, 1425, 1325, 1190, 1177, 1155, 1126, 1109, 1076, 1053, 1024, 1001, 982, 964, 934, 908, 874, 826, 754, 694, 610; **^{1}H NMR** (400 MHz, $CDCl_3$) δ 7.35 – 7.21 (m, 8H), 7.22 – 7.13 (m, 2H), 1.31 (s, 4H); **^{13}C NMR** (101 MHz, $CDCl_3$) δ 145.9, 128.6, 128.4, 126.1, 30.0, 16.6; ***m/z* (EI)**: 194.1 (M^+, 100%), 178.1 (63), 165.1 (63), 152.1 (16), 139.1 (12), 128.1 (4), 115.1 (97), 103.1 (12), 91.1 (25), 77.1 (15), 63.1 (16), 51.1 (21). Analytical data are consistent with those previously reported in the literature.[36]

Reaction of 3,3-diphenylpropan-1-ol 36 in 1,4-dioxane

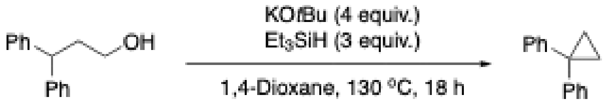

36 38 93%

This reaction was carried out according to general procedure I with 3,3-diphenylpropan-1-ol **36** (106 mg, 0.5 mmol, 1 equiv.), Et_3SiH (240 µL, 1.5 mmol, 3 equiv.), KO*t*Bu (224 mg, 2 mmol, 4 equiv.) and anhydrous 1,4-dioxane (5 mL). Purification by column chromatography (hexane) afforded cyclopropane-1,1-diyldibenzene **38** (90 mg, 93%) as a colourless oil with analytical data in agreement with those outlined above.

Reaction of 3-phenyl-3-(pyridin-2-yl)propan-1-ol S12

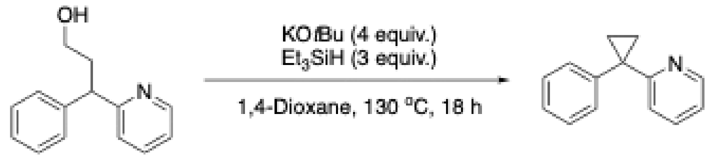

S12 34 76%

The reaction followed general procedure I using 3-phenyl-3-(pyridin-2-yl)propan-1-ol **S12** (107 mg, 0.5 mmol, 1 equiv.), Et_3SiH (240 µL, 1.5 mmol, 3 equiv.), KO*t*Bu (224 mg, 4 equiv., 2 mmol,) and anhydrous 1,4-dioxane (5 mL). Purification by chromatography (100% hexane→3% EtOAc in hexane) afforded 2-(1-phenylcyclopropyl)pyridine **34** (74 mg, 76%) as a yellow oil with analytical data in agreement with those outlined above.

Reaction of 3-phenyl-3-(pyridin-4-yl)propan-1-ol S14

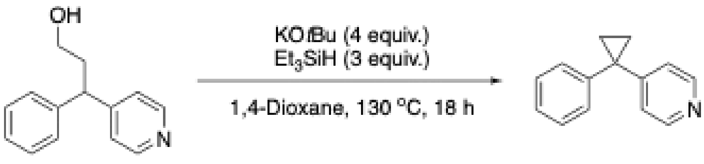

S14 **35** 76%

The reaction followed general procedure I using 3-phenyl-3-(pyridin-2-yl)propan-1-ol **S14** (107 mg, 0.5 mmol, 1 equiv.), Et_3SiH (240 μL, 1.5 mmol, 3 equiv.), KO*t*Bu (224 mg, 4 equiv., 2 mmol,) and anhydrous 1,4-dioxane (5 mL). Purification by chromatography (100% hexane→3% EtOAc in hexane) afforded 4-(1-phenylcyclopropyl)pyridine **35** (74 mg, 76%) as a brown oil with analytical data in agreement with those outlined above.

Reaction of 2-(1-phenyl-3-((triethylsilyl)oxy)propyl)pyridine S15

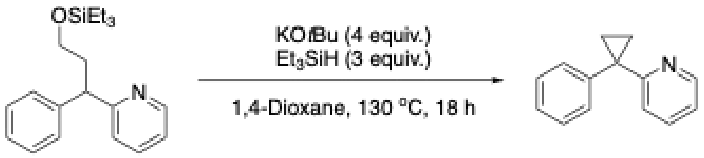

S15 **34** 68%

The reaction followed general procedure I using 2-(1-phenyl-3-((triethylsilyl)oxy)propyl) pyridine **S15** (164 mg, 0.5 mmol, 1 equiv.), Et_3SiH (240 μL, 1.5 mmol, 3 equiv.), KO*t*Bu (168 mg, 1.5 mmol, 3 equiv.) and anhydrous 1,4-dioxane (5 mL). Purification by chromatography (100% hexane→3% EtOAc in hexane) afforded 2-(1-phenylcyclopropyl)pyridine **34** (66 mg, 68%) as a yellow oil with analytical data consistent with the data outlined above.

Reaction of 2-(9*H*-xanthen-9-yl)ethan-1-ol S18

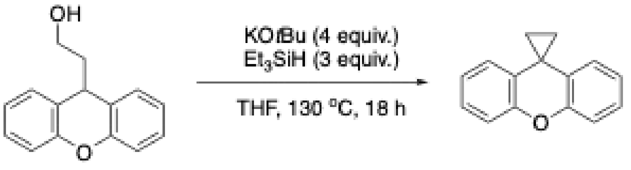

S18 **39** 43%

The reaction followed general procedure I using 2-(9*H*-xanthen-9-yl)ethan-1-ol **S18** (106 mg, 0.5 mmol, 1 equiv.), triethylsilane (236 μL, 1.5 mmol, 3 equiv.), KO*t*Bu (224 mg, 2 mmol, 4 equiv.) and 1,4-dioxane (5 mL). Purification by chromatography (100% hexanes) affording spiro[cyclopropane-1,9'-xanthene] **39** as a colourless oil (58 mg, 43%). $\mathbf{v_{max}}$ (neat)/cm^{-1} 3067, 2935, 2876, 1581, 1458, 1270; ^{1}**H NMR** (400 MHz, $CDCl_3$) δ 7.17 - 7.08 (m, 2H), 7.01 - 6.96 (m, 4H), 6.73 (dd, *J* = 1.5, 8.0 Hz, 2H), 1.43 (s, 4H); ^{13}**C NMR** (101 MHz, $CDCl_3$) δ (ppm) = 152.0, 126.8, 125.8, 123.1, 122.0, 115.8, 24.4, 17.6; ***m/z*** (ESI+) calcd. for $C_{15}H_{13}O$ $[M+H]^+$ 209.0961, found 209.0956.

Reaction of 3-(4-(*tert*-butyl)phenyl)propan-1-ol S21

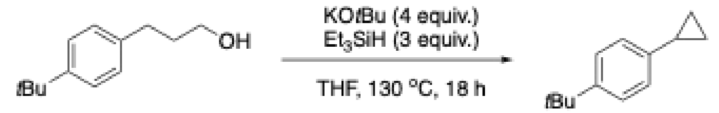

S21 **41** 33%

The reaction was carried out according to general procedure I using 3-(4-(*tert*-butyl)phenyl)propan-1-ol **S21** (96 mg, 0.5 mmol, 1 equiv.), KO*t*Bu (224 mg, 2 mmol, 4 equiv.), Et_3SiH (0.24 mL, 1.5 mmol, 3 equiv.) and THF (5mL). The crude material was purified via column chromatography (100% hexane) affording 1-(*tert*-butyl)-4-cyclopropylbenzene **41** as a colourless oil (31.8 mg, 33%). $\mathbf{v_{max}}$ (neat)/cm^{-1} 3106, 2987, 2927, 2891, 1532, 1477, 1370, 1246, 1207, 1056, 1028, 909, 825, 746, 730, 567; **^{1}H NMR** ($CDCl_3$, 400 MHz) δ 7.29 – 7.27 (m, 2H), 7.02 – 7.00 (m, 2H), 1.90 – 1.83 (m, 1H), 1.30 (s, 9H), 0.95 – 0.89 (m, 2H), 0.69 – 0.65 (m, 2H); **^{13}C NMR** (101 MHz, $CDCl_3$) δ 148.2, 140.8, 125.3, 125.1, 34.3, 31.3, 14.8, 8.9. NMR data were in agreement with previous literature.[37]

Reaction of 3-(4-(*tert*-butyl)phenyl)propan-1-ol in THP

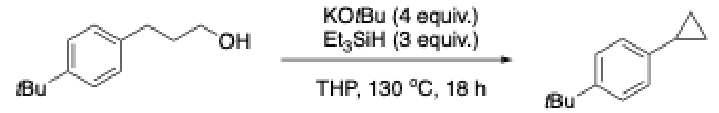

S21 **41** 66%

The reaction was carried out according to general procedure I using 3-(4-(*tert*-butyl)phenyl)propan-1-ol (96 mg, 0.5 mmol, 1 equiv.), KO*t*Bu (224 mg, 2 mmol, 4 equiv.), Et_3SiH (0.24 mL, 1.5 mmol, 3 equiv.) and THP (5 mL). The crude material was purified via column chromatography (100% hexane) affording 1-(*tert*-butyl)-4-cyclopropylbenzene **41** as a colourless oil (50 mg, 66%), with analytical data in agreement with that reported above.

Reaction of 3-(naphthalen-1-yl)propan-1-ol

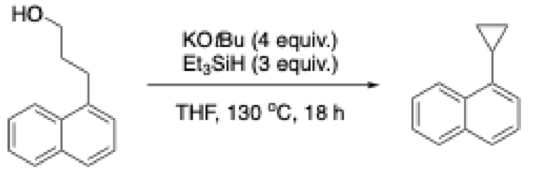

S23 **42** 77%

This experiment followed general procedure I using 3-(naphthalen-1-yl)propan-1-ol **S23** (93 mg, 0.5 mmol, 1 equiv.), Et_3SiH (240 µL, 1.5 mmol, 3 equiv.), and KO*t*Bu (224 mg, 2 mmol, 4 equiv.), in THF (5 mL). Purification by chromatography (100% hexanes) afforded 1-cyclopropylnaphthalene **42** as a colourless oil (65 mg, 77%). **^{1}H NMR** (400 MHz, $CDCl_3$) δ 8.47 (dd, J = 1.0, 8.5 Hz, 1H), 7.90 (dd, J = 1.0, 8.3 Hz, 1H), 7.75 (dd, J = 0.5, 8.3 Hz, 1H), 7.62 - 7.51 (m, 2H), 7.43 (t, J = 7.3 Hz, 1H), 7.32 (td, J = 1.1, 7.2 Hz, 1H), 2.40 (tt, J = 5.5, 8.4 Hz, 1H), 1.14 - 1.08 (m, 2H), 0.86 - 0.80 (m, 2H); **^{13}C NMR** (101 MHz, $CDCl_3$) δ 139.1, 133.5, 133.5, 128.4, 126.5, 125.7, 125.55, 125.46, 124.4, 123.7, 13.2, 6.4. Data were in accordance with previously published literature.[37]

Reaction of 3-(naphthalen-2-yl)propan-1-ol S26

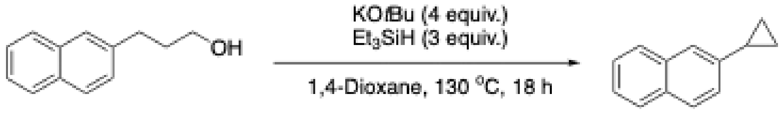

This experiment followed general procedure I using 3-(naphthalen-2-yl)propan-1-ol **S26** (93 mg, 0.5 mmol, 1 equiv.), KO*t*Bu (224 mg, 2 mmol, 4 equiv.), Et_3SiH (0.24 mL, 1.5 mmol, 3 equiv.) and dioxane (5mL). The crude was purified via column chromatography (100% hexane) affording 2-cyclopropylnaphthalene **43** as a white gum (19.5 mg, 24%). $\mathbf{v_{max}}$ (neat)/cm^{-1} 3110, 3074, 3014, 1644, 1604, 1521, 1397, 1278, 1227, 1175, 1020, 956, 853, 817, 742, 647; **^{1}H NMR** ($CDCl_3$, 400 MHz) δ 7.79 – 7.73 (m, 3H), 7.54 (s, 1H), 7.46 – 7.37 (m, 2H), 7.20 (dd, *J* = 1.8, 8.5 Hz, 1H), 2.10 – 2.04 (m, 1H), 1.06 – 1.01 (m, 2H), 0.84 – 0.80 (m, 2H); **^{13}C NMR** (101 MHz, $CDCl_3$) δ 140.9, 133.0, 131.4, 127.3, 127.0, 126.7, 125.4, 124.3, 124.1, 123.2, 15.1, 8.6. Data were in accordance with previously published literature.[37]

Reaction of 3-([1,1'-biphenyl]-4-yl)propan-1-ol S29

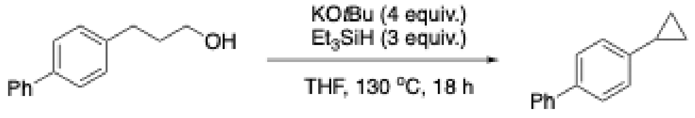

This experiment followed general procedure I, using 3-([1,1'-biphenyl]-4-yl)propan-1-ol **S29** (106 mg, 0.5 mmol, 1 equiv.), Et_3SiH (240 μL, 1.5 mmol, 3 equiv.), KO*t*Bu (224 mg, 2 mmol, 4 equiv.) and THF (5 mL). Purification by chromatography (100% hexane) afforded 4-cyclopropyl-1,1'-biphenyl **44** as a white solid (45 mg, 60%). **^{1}H NMR** (400 MHz, $CDCl_3$) δ 7.61 - 7.56 (m, 2H), 7.51 (d, *J* = 8.3 Hz, 2H), 7.46 - 7.41 (m, 2H), 7.33 (tt, *J* = 1.7, 7.3 Hz, 1H), 7.16 (d, *J* = 8.0 Hz, 2H), 1.96 (tt, *J* = 5.1, 8.4 Hz, 1H), 1.04 - 0.98 (m, 2H), 0.78 - 0.73 (m, 2H); **^{13}C NMR** (101 MHz, $CDCl_3$ δ 143.5, 141.4, 138.7, 129.0, 127.3, 127.3, 127.2, 126.4, 15.4, 9.6. Data were in accordance with previously reported literature.[37]

Reaction of 3-([1,1'-biphenyl]-3-yl)propan-1-ol S32

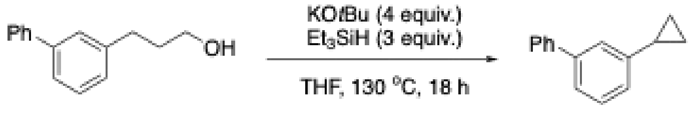

This experiment followed general procedure I using 3-([1,1'-biphenyl]-3-yl)propan-1-ol **S32** (106 mg, 0.5 mmol, 1 equiv.), KO*t*Bu (224mg, 2 mmol, 4 equiv.) and Et_3SiH (240 µL, 1.5 mmol, 3 equiv.), 3-cyclopropyl-1,1'-biphenyl **45** (84 mg, 86 %) was isolated as a colourless oil. $\mathbf{v_{max}}$ (neat)/cm^{-1} 3030, 1598, 1479, 1419, 1170, 1043, 1018, 906, 812, 794, 754, 698, 669; **^{1}H NMR** (400 MHz, $CDCl_3$) δ 7.62 – 7.54 (m, 2H), 7.48 – 7.40 (m, 2H), 7.40 – 7.27 (m, 4H), 7.06 (dt, *J* = 7.4, 1.5 Hz, 1H), 2.03 – 1.92 (m, 1H), 1.05 – 0.97 (m, 2H), 0.81 – 0.72 (m, 2H); **^{13}C NMR** (101 MHz, $CDCl_3$) δ 144.6, 141.6, 141.5, 128.8, 127.4, 127.3, 124.9, 124.7, 124.5, 15.6, 9.4. 1 carbon not observed, hidden under aromatic peaks; ***m/z*** (EI) 194.1 (M^+, 100), 178.1 (60), *165.1 (52), 152.0 (29), 139.0 (5), 128.1 (7), 117.1 (21), 103.1 (4), 89.0 (7), 77.0 (8). NMR data* were in agreement with literature reports.[38]

Reaction of 3-([1,1'-biphenyl]-2-yl)propan-1-ol S35

In this case, besides the expected product **46**, a byproduct **S54** was also isolated. We attribute this to abstraction of a benzylic H-atom in **S35** or its silyl ether. The radical then undergoes intramolecular attack on the Ph group. Deprotonation of the resulting radical and loss of an electron (SET) gives the fluorene ring system (see mechanistic proposal under the spectrum below)

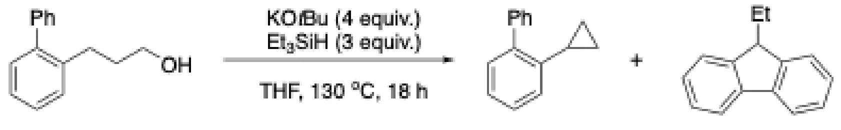

S35 **46** 39% **S55** 9%

This experiment followed general procedure I using 3-([1,1'-biphenyl]-2-yl)propan-1-ol **S35** (106 mg, 0.5 mmol, 1 equiv.), KO*t*Bu (224 mg, 2 mmol, 4 equiv.) and Et_3SiH (240 µL, 1.5 mmol, 3 equiv.) in anhydrous THF (5 mL). Purification afforded 2-cyclopropyl-1,1'-biphenyl **46** (39 %) and 9-ethyl-9H-fluorene **S55** (9 %), as a co-eluting mixture as a yellow oil. The yields were determined by internal standard NMR using TCE (40.8 mg, 0.24 mmol) as an internal standard. TCE CH signal (5.96 ppm) was integrated to 2H, 2-cyclopropyl-1,1'-biphenyl **46** CH peak at 1.94 ppm was used to calculate the yield. For 9-ethyl-9H-fluorene **S55**, the CH peak at 4.01 ppm was used to calculate the yield

2-cyclopropyl-1,1'-biphenyl **46**, yield $= \frac{\left({}^{0.8/1}/_{2/2}\right) \times 0.24mmol}{0.5mmol} = 39\%$.

9-ethyl-9H-fluorene **S55**, yield $= \frac{\left({}^{0.18/1}/_{2/2}\right) \times 0.24mmol}{0.5mmol} = 9\%$.

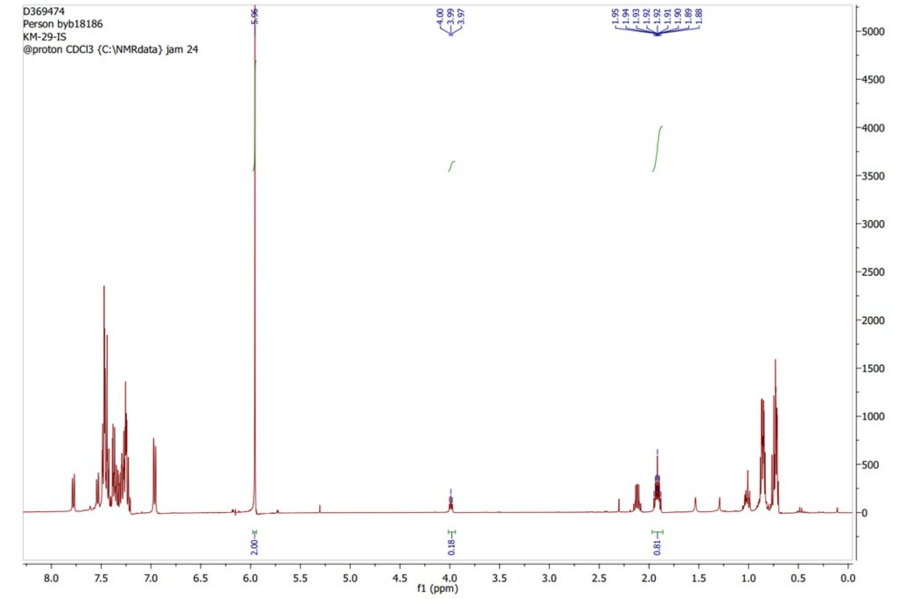

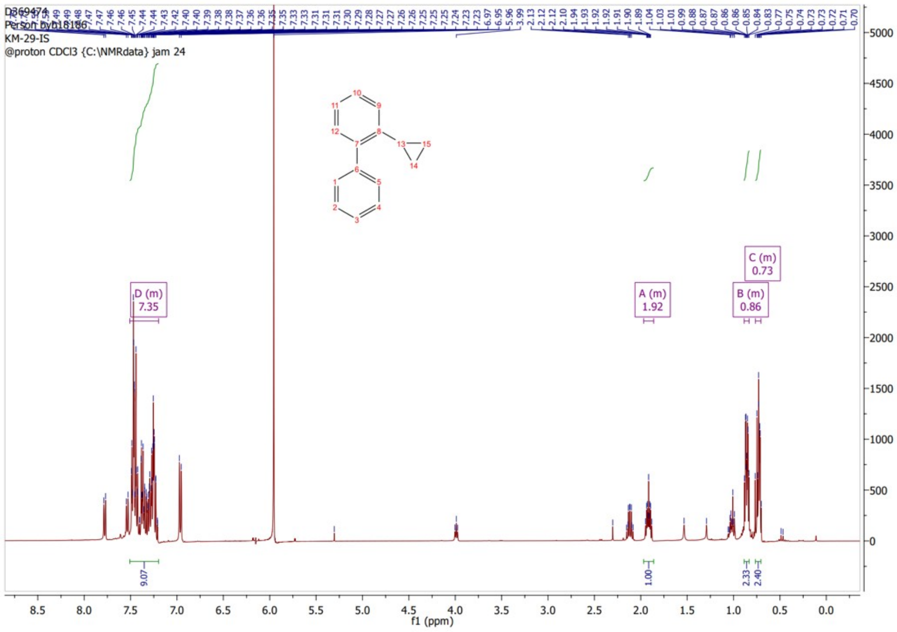

Possible mechanism for formation of **S55**

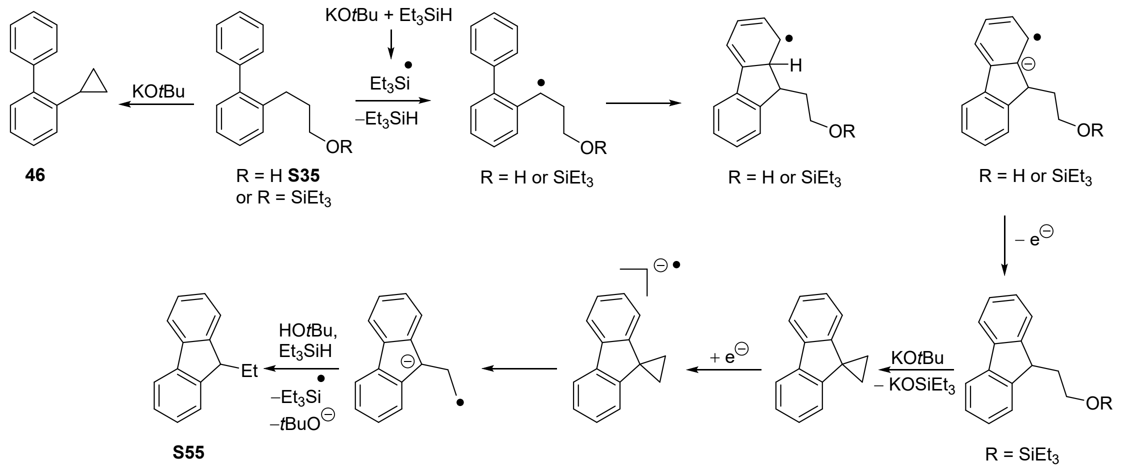

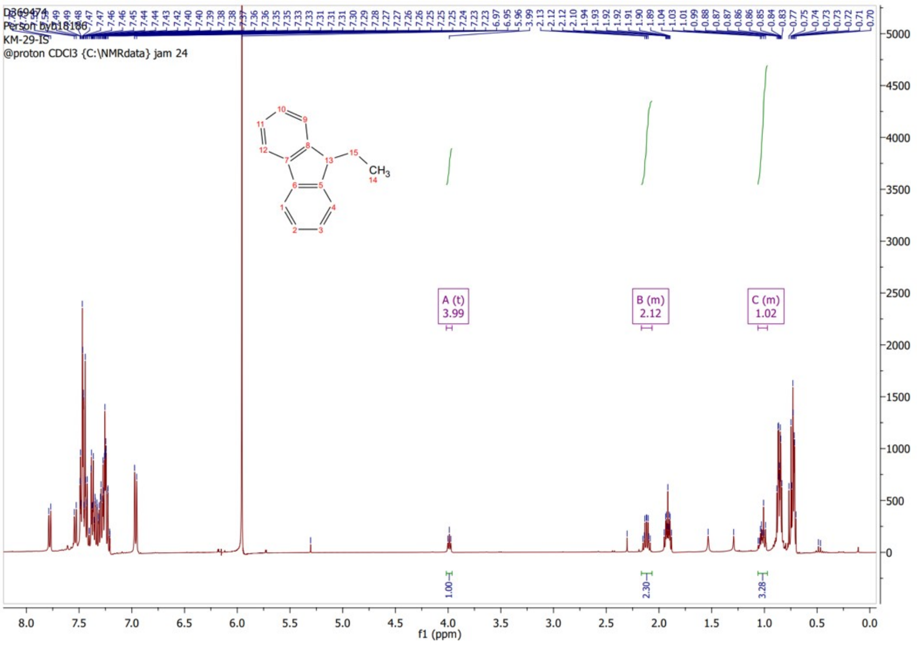

Reaction 3-(quinolin-2-yl)propan-1-ol S37

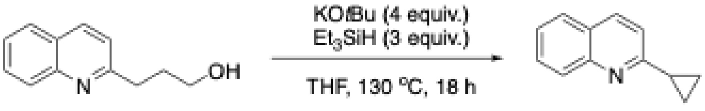

S37 **47** 42%

The reaction was carried out according to general procedure I with 3-(quinolin-2-yl)propan-1-ol **S37** (94 mg, 0.5 mmol, 1 equiv.), KO*t*Bu (224 mg, 2 mmol, 4 equiv.), and Et_3SiH (240 μL, 1.5 mmol, 3 equiv.) in anhydrous THF (5 mL). The crude mixture was purified by chromatography (100% hexanes→5% EtOAc in hexanes) affording 2-cyclopropylquinoline **47** (36.8 mg, 42%) as a colourless oil. $\mathbf{v_{max}}$ (neat)/cm^{-1} 3005, 1616, 1599, 1560, 1503, 1425, 1204, 1082, 1022, 951; **^{1}H NMR** (400 MHz, $CDCl_3$) δ 8.00 (d, *J* = 8.5 Hz, 1H), 7.97 (d, *J* = 8.7 Hz, 1H), 7.74 (dd, *J* = 8.0, 1.2 Hz, 1H), 7.67 – 7.61 (m, 1H), 7.46 – 7.40 (m, 1H), 7.17 (d, *J* = 8.5 Hz, 1H), 2.29 – 2.21 (m, 1H), 1.19 – 1.14 (m, 2H), 1.13 – 1.06 (m, 2H); **^{13}C NMR** (101 MHz, $CDCl_3$) δ 163.6, 148.1, 136.0, 129.4, 128.8, 127.6, 126.9, 125.3, 119.5, 18.2, 10.4; ***m/z*** (EI): 168 (M-1, 100), 154 (3), 143 (11), 128 (9), 115 (7), 101 (7), 84 (7), 75 (7), 63 (6), 51 (6).

Reaction of 3-(pyridin-4-yl)propan-1-ol S39

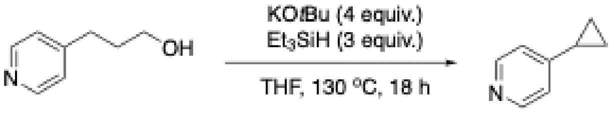

S39 **48** 72%

The reaction was carried out according to general procedure I with 3-(pyridin-4-yl)propan-1-ol **S39** (68.6 mg, 0.5 mmol, 1 equiv.), KO*t*Bu (224 mg, 2 mmol, 4 equiv.), and Et_3SiH (240 μL, 1.5 mmol, 3 equiv.) in anhydrous THF (5 mL). The yield of 4-cyclopropylpyridine **48** was

determined by the addition of 1,1,2,2-tetrachloroethane (TCE) (41.9 mg, 0.25 mmol) as an internal standard. The CH signal of 1,1,2,2-tetrachloroethane (5.94 ppm) was integrated to 2H, and the relative intensity of the 2x Ar-H group of 4-cyclopropylpyridine **46** (6.93 ppm) was used to calculate the yield. For 4-cyclopropylpyridine **48**, yield = [[2.86/2] x 0.25 mmol] / 0.5 mmol = 72%. The crude mixture was then purified by chromatography (100% hexanes→50% EtOAc in hexanes) affording 4-cyclopropylpyridine **48** (5 mg, 8%) as a yellow oil. $\mathbf{v_{max}}$ (neat)/cm^{-1} 2955, 2922, 2853, 1495, 1463, 1364, 1248, 1186, 1080; **^{1}H NMR** (400 MHz, $CDCl_3$) δ 8.43 (br s, 2H), 6.96 (d, *J* = 4.8 Hz, 2H), 1.89 – 1.81 (m, 1H), 1.13 – 1.04 (m, 2H), 0.82 – 0.76 (m, 2H); **^{13}C NMR** (101 MHz, $CDCl_3$) δ 154.0, 149.4, 120.9, 15.1, 10.7; ***m/z*** (ESI+) calcd. for $C_{14}H_{26}ONSi$ $[M+H]^+$ 252.1778, found 252.1778.

Reaction of 3-(4-(thiophen-2-yl)phenyl)propan-1-ol S41

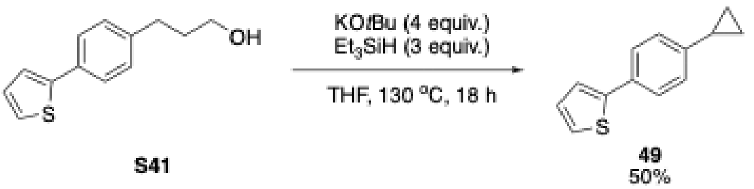

The reaction was carried out according to general procedure I with 3-(4-(thiophen-2-yl)phenyl)propan-1-ol **S41** (109 mg, 0.5 mmol, 1 equiv.), KO*t*Bu (224 mg, 2 mmol, 4 equiv.), and Et_3SiH (240 µL, 1.5 mmol, 3 equiv.) in anhydrous THF (5 mL). The crude mixture was purified by chromatography (100% hexanes) affording 2-(4-cyclopropylphenyl)thiophene **49** (50 mg, 50%) as a white solid. **Mp** 62-64°C; $\mathbf{v_{max}}$ (neat)/cm^{-1} 3075, 1505, 1460, 1263, 1043, 902, 812; **^{1}H NMR** (400 MHz, $CDCl_3$) δ 7.51 (d, *J* = 8.3 Hz, 2H), 7.28 – 7.22 (m, 2H), 7.12 – 7.05 (m, 3H), 1.96 – 1.87 (m, 1H), 1.03 – 0.96 (m, 2H), 0.77 – 0.69 (m, 2H); **^{13}C NMR** (101 MHz, $CDCl_3$) δ 144.7, 143.7, 131.8, 128.0, 126.2, 126.0, 124.4, 122.7 15.4, 9.5; ***m/z*** (EI): 200 (M+, 100), 184 (21), 165 (41), 139 (7), 115 (22).

Reaction of 3-(benzo[*b*]thiophen-3-yl)propan-1-ol S44

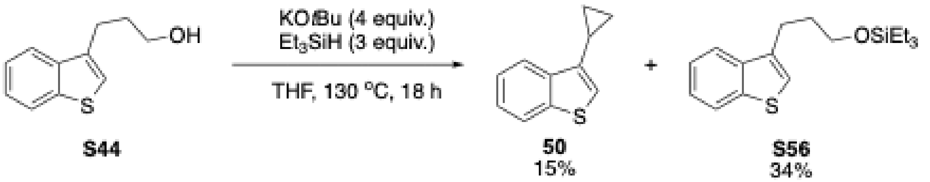

The reaction was carried out according to general procedure I with 3-(benzo[*b*]thiophen-3-yl)propan-1-ol **S44** (96 mg, 0.5 mmol, 1 equiv.), KO*t*Bu (224 mg, 2 mmol, 4 equiv.), and Et_3SiH (240 µL, 1.5 mmol, 3 equiv.) in anhydrous THF (5 mL). The crude mixture was purified by chromatography (100% hexanes→30% EtOAc in hexanes) affording 3-cyclopropylbenzo[*b*]thiophene **50** (12.6 mg, 15%) as a colourless oil and (3-(benzo[*b*]thiophen-3-yl)propoxy)triethylsilane **S56** (52 mg, 34%) as a yellow oil. **50** $\mathbf{v_{max}}$ (neat)/cm^{-1} 3003, 2953, 2924, 1460, 1428, 1305, 1254, 1020; **^{1}H NMR** (400 MHz, $CDCl_3$) δ 7.99 – 7.94 (m, 1H), 7.84 (d, *J* = 7.9 Hz, 1H), 7.44 – 7.32 (m, 2H), 6.96 (s, 1H), 2.11 – 2.01 (m, 1H), 1.02 – 0.94 (m, 2H), 0.75 – 0.69 (m, 2H); **^{13}C NMR** (101 MHz, $CDCl_3$) δ 140.6, 140.1, 138.9, 124.5, 124.0, 122.9, 122.2, 119.9, 9.3, 6.3; ***m/z*** (EI): 174 (M+, 100), 159 (4), 147 (92), 129 (26), 115 (31), 102 (17). (3-(Benzo[*b*]thiophen-3-yl)propoxy)triethylsilane **S55** $\mathbf{v_{max}}$

(neat)/cm^{-1} 3292, 2932, 2872, 1425, 1256, 1055, 1020; ^{1}**H NMR** (400 MHz, $CDCl_3$) δ 7.89 – 7.86 (m, 1H), 7.81 – 7.77 (m, 1H), 7.42 – 7.32 (m, 2H), 7.11 (s 1H), 3.74 (t, *J* = 6.1 Hz, 2H), 2.97 – 2.91 (m, 2H), 2.05 – 1.96 (m, 2H), 1.00 (t, *J* = 7.9 Hz, 9H), 0.64 (q, *J* = 7.7 Hz, 6H); ^{13}**C NMR** (101 MHz, $CDCl_3$) δ 140.6, 139.2, 136.8, 124.2, 123.9, 123.0, 121.9, 121.1, 62.3, 32.4, 25.0, 7.0, 4.6; ***m/z*** (ESI+) calcd. for $C_{17}H_{27}OSSi$ $(M+H)^+$ 357.15464, found 357.1543.

Reactions with KH

Reaction of (3-methoxypropane-1,1-diyl)dibenzene S47 (Table 2, entry 1)

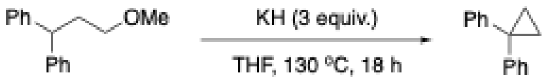

S47 **38** 49%

The reaction followed general procedure J using (3-methoxypropane-1,1-diyl)dibenzene **S47** (113 mg, 0.5 mmol, 1 equiv.) and KH (60 mg, 1.5 mmol, 3 equiv.) in anhydrous THF (5 mL). The crude mixture was purified by chromatography (100% hexanes→1% DCM in hexanes) affording cyclopropane-1,1-diyldibenzene **38** (47.6 mg, 49%) as a colourless oil. Analytical data were in agreement with the corresponding data reported previously.

Reaction of (3-ethoxypropane-1,1-diyl)dibenzene S48 (Table 2, entry 2)

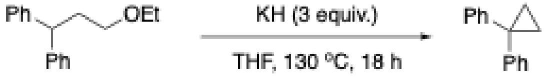

S48 **38** 30%

The reaction followed general procedure J using (3-ethoxypropane-1,1-diyl)dibenzene **S48** (120 mg, 0.5 mmol, 1 equiv.) and KH (60 mg, 1.5 mmol, 3 equiv.) in anhydrous THF (5 mL). The crude mixture was purified by chromatography (100% hexanes→1% DCM in hexanes) affording cyclopropane-1,1-diyldibenzene **38** (29.9 mg, 30%) as a colourless oil. Analytical data were in agreement with the corresponding data reported previously.

Reaction of (3-phenoxypropane-1,1-diyl)dibenzene S49 (Table 2, entry 3)

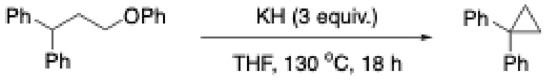

S49 **38** 69%

The reaction followed general procedure J using (3-phenoxypropane-1,1-diyl)dibenzene **S49** (144 mg, 0.5 mmol, 1 equiv.) and KH (60 mg, 1.5 mmol, 3 equiv.) in anhydrous THF (5 mL). The crude mixture was purified by chromatography (100% hexanes) affording cyclopropane-1,1-diyldibenzene **38** (68.7 mg, 69%) as a colourless oil. Analytical data were in agreement with the corresponding data reported previously.

Reaction of (3,3-diphenylpropoxy)triethylsilane S57 without Et_3SiH (Table 2, entry 4)

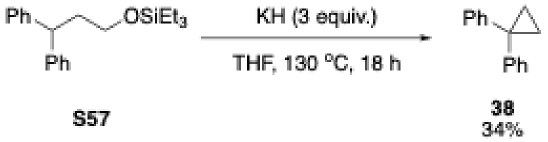

The reaction followed general procedure J using (3,3-diphenylpropoxy)triethylsilane **S57** (163 mg, 0.5 mmol, 1 equiv.) and KH (60 mg, 1.5 mmol, 3 equiv.) in anhydrous THF (5 mL). The crude mixture was purified by chromatography (100% hexanes) affording cyclopropane-1,1-diyldibenzene **38** (33 mg, 34%) as a colourless oil. Analytical data were in agreement with the corresponding data reported previously.

Reaction of (3,3-diphenylpropoxy)triethylsilane S57 with Et_3SiH (Table 2, entry 5)

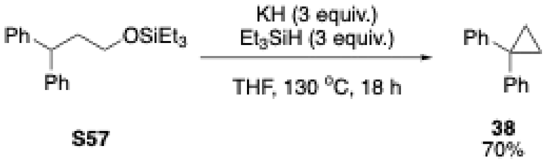

The reaction followed general procedure J using (3,3-diphenylpropoxy)triethylsilane **S57** (163 mg, 0.5 mmol, 1 equiv.), KH (80 mg, 2 mmol, 4 equiv.), and Et_3SiH (240 μL, 1.5 mmol, 3 equiv.) in anhydrous THF (5 mL). Purification by chromatography (100% hexanes) afforded cyclopropane-1,1-diyldibenzene **38** (67.5 mg, 70%) as a colourless oil. Analytical data were in agreement with the corresponding data reported previously.

Reaction of 3,3-diphenylpropan-1-ol 36 at 130°C (Table 2, entry 6)

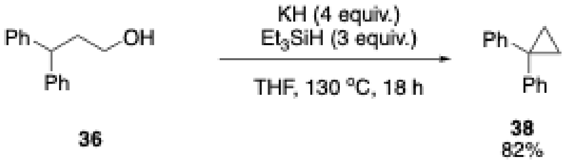

The reaction followed general procedure J using 3,3-diphenylpropan-1-ol **36** (106 mg, 0.5 mmol, 1 equiv.), KH (80 mg, 2 mmol, 4 equiv.), and Et_3SiH (240 μL, 1.5 mmol, 3 equiv.) in anhydrous THF (5 mL). Purification by chromatography (100% hexanes) afforded cyclopropane-1,1-diyldibenzene **38** (79.6 mg, 82%) as a colourless oil. Analytical data were in agreement with the corresponding data reported previously.

Reaction of 3,3-diphenylpropan-1-ol at 100°C (Table 2, entry 7)

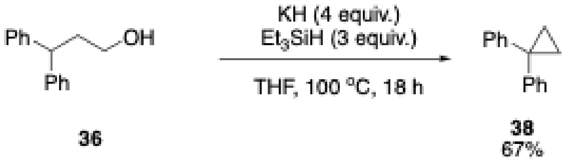

The reaction followed an adapted general procedure J using 3,3-diphenylpropan-1-ol **36** (106 mg, 0.5 mmol, 1 equiv.), KH (80 mg, 2 mmol, 4 equiv.), and Et_3SiH (240 µL, 1.5 mmol, 3 equiv.) in anhydrous THF (5 mL). The reaction was refluxed at 100 °C for 18 h. Purification by chromatography (100% hexanes) afforded cyclopropane-1,1-diyldibenzene **38** (74 mg, 67%) as a colourless oil. Analytical data were in agreement with the corresponding data reported previously.

Reaction of 3,3-diphenylpropan-1-ol 36 at 80°C (Table 2, entry 8)

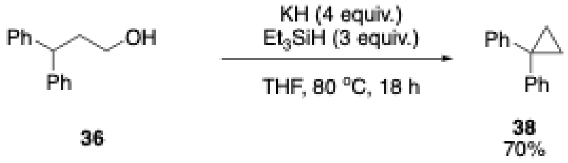

The reaction followed an adapted general procedure J using 3,3-diphenylpropan-1-ol **36** (106 mg, 0.5 mmol, 1 equiv.), KH (80 mg, 2 mmol, 4 equiv.), and Et_3SiH (240 µL, 1.5 mmol, 3 equiv.) in anhydrous THF (5 mL). The reaction was refluxed at 80 °C for 18 h. Purification by chromatography (100% hexanes) afforded cyclopropane-1,1-diyldibenzene **38** (71.9 mg, 70%) as a colourless oil. Analytical data were in agreement with the corresponding data reported previously.

Reaction of 3,3-diphenylpropan-1-ol 49 at 60°C (Table 2, entry 9)

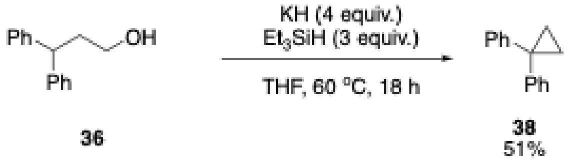

The reaction followed an adapted general procedure J using 3,3-diphenylpropan-1-ol **36** (106 mg, 0.5 mmol, 1 equiv.), KH (80 mg, 2 mmol, 4 equiv.), and Et_3SiH (240 µL, 1.5 mmol, 3 equiv.) in anhydrous THF (5 mL). The reaction was refluxed at 60 °C for 18 h. Purification by chromatography (100% hexanes) afforded cyclopropane-1,1-diyldibenzene **38** (50 mg, 51%) as a colourless oil. Analytical data were in agreement with the corresponding data reported previously.

Reaction of 3,3-diphenylpropan-1-ol 49 at 80°C for 3h (Table 2, entry 10)

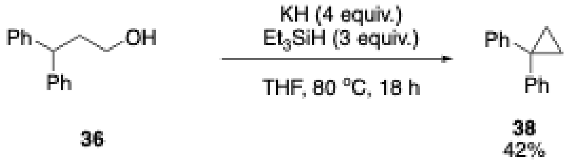

The reaction followed an adapted general procedure J using 3,3-diphenylpropan-1-ol **36** (106 mg, 0.5 mmol, 1 equiv.), KH (80 mg, 2 mmol, 4 equiv.), and Et_3SiH (240 µL, 1.5 mmol, 3 equiv.) in anhydrous THF (5 mL). The reaction was refluxed at 80 °C for 3 h. Purification by chromatography (100% hexanes) afforded cyclopropane-1,1-diyldibenzene **38** (40 mg, 42%)

as a colourless oil. Analytical data were in agreement with the corresponding data reported previously.

Silane Scope

Reaction of 3,3-diphenylpropan-1-ol 36 with *i*Pr$_3$SiH (Table 3, entry 1)

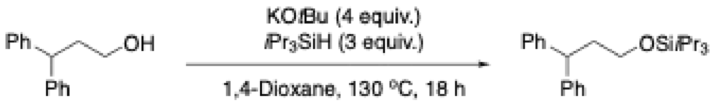

This reaction was carried out according to an adapted general procedure I with 3,3-diphenylpropan-1-ol **36** (106 mg, 0.5 mmol, 1 equiv.), iPr$_3$SiH (308 μL, 1.5 mmol, 3 equiv.), KO*t*Bu (224 mg, 2 mmol, 4 equiv.) and anhydrous 1,4-dioxane (5 mL) afforded (3,3-diphenylpropoxy)triisopropylsilane **S58** (195 mg, 100%) as a yellow oil with no further purification required. **v_{max}** (neat)/cm^{-1} 2929, 2889, 2864, 1492, 1462, 1450, 1382, 1246, 1101, 1051, 1012, 995, 943, 881, 783, 758, 742, 696, 678, 657, 638; **^{1}H NMR** (400 MHz, CDCl$_3$) δ 7.30 – 7.11 (m, 10H), 4.19 (t, *J* = 7.8 Hz, 1H), 3.60 (t, *J* = 6.3 Hz, 2H), 2.27 (dt, *J* = 6.4, 7.8 Hz, 2H), 1.11 – 0.92 (m, 21H, 3 x SiCH; 6 x CH$_3$); **^{13}C NMR** (101 MHz, CDCl$_3$) δ 145.0, 128.5, 128.2, 126.2, 61.3, 47.1, 38.9, 18.2, 12.1**.** ***m/z*** (EI): 325.2 (M+, 100%), 239.1 (4), 221.1 (70), 193.1 (14), 179.1 (33), 167.1 (45), 152.1 (14), 143.1 (7), 135.0 (4), 123.0 (7), 115.1 (22), 103.1 (42), 91.0 (18), 75.1 (8).

Reaction of 3,3-diphenylpropan-1-ol 36 with Me$_2$PhSiH (Table 3, entry 2)

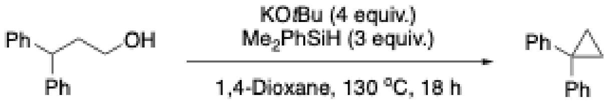

This reaction was carried out according to an adapted general procedure I with 3,3-diphenylpropan-1-ol **36** (106 mg, 0.5 mmol, 1 equiv.), Me$_2$PhSiH (229 μL, 1.5 mmol, 3 equiv.), KO*t*Bu (224 mg, 2 mmol, 4 equiv.) and anhydrous 1,4-dioxane (5 mL). Purification by chromatography (100% hexanes) afforded cyclopropane-1,1-diyldibenzene **38** (73.7 mg, 76%) as a colourless oil. Analytical data were consistent with those reported above.

Reaction of 3,3-diphenylpropan-1-ol with *t*BuMe$_2$SiH (Table 3, entry 3)

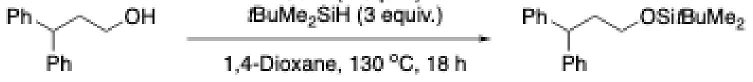

This reaction was carried out according to an adapted general procedure I with 3,3-diphenylpropan-1-ol **36** (106 mg, 0.5 mmol, 1 equiv.), *t*BuMe$_2$SiH (248 μL, 1.5 mmol, 3 equiv.),

KO*t*Bu (224 mg, 2 mmol, 4 equiv.) and anhydrous 1,4-dioxane (5 mL) afforded *tert*-butyl(3,3-diphenylpropoxy)dimethylsilane **S59** (146 mg, 89%) without requiring further purification. $\mathbf{v_{max}}$ (neat)/cm^{-1} 2951, 2927, 2854, 1492, 1450, 1386, 1251, 1099, 1051, 1029, 1004, 945, 831, 812, 773, 754, 696, 663; **^{1}H NMR** (400 MHz, $CDCl_3$) δ 7.33 – 7.12 (m, 10H), 4.15 (t, *J* = 7.8 Hz, 1H), 3.55 (t, *J* = 6.4 Hz, 2H), 2.27 (dt, *J* = 6.4, 7.8 Hz, 2H), 0.90 (s, 9H), -0.01 (s, 6H, 2 x $SiCH_3$); **^{13}C NMR** (101 MHz, $CDCl_3$) δ 144.9, 128.5, 128.1, 126.2, 61.1, 47.2, 38.6, 26.1, 18.4, -5.2; ***m/z*** (EI+) 269.1 (M+, 45%), 193.1 (6), 178.1 (4), 165.1 (100), 152.1 (11), 135.1 (30), 115.1 (8), 102.0 (3), 89.1 (18), 75.0 (15), 59 (7).

Reaction of 3,3-diphenylpropan-1-ol 36 with Bu_3SnH (Table 3, entry 4)

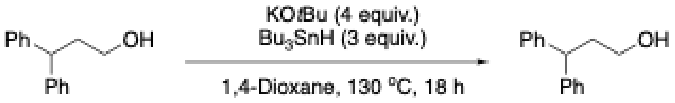

36 36 89%

This reaction was carried out according to an adapted general procedure I with 3,3-diphenylpropan-1-ol **36** (106 mg, 0.5 mmol, 1 equiv.), Bu_3SnH (248 µL, 1.5 mmol, 3 equiv.), KO*t*Bu (224 mg, 2 mmol, 4 equiv.) and anhydrous 1,4-dioxane (5 mL) afforded unreacted starting material (94.9 mg, 89%)

Reactions of thioethers, sulfoxides and sulfones

Reaction of (3,3-diphenylpropyl)(phenyl)sulfane 54

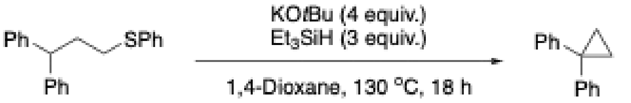

54 38 75%

The reaction was carried out according to general procedure I with (3,3-diphenylpropyl)(phenyl)sulfane **54** (152 mg, 0.5 mmol, 1 equiv.), KO*t*Bu (224 mg, 2 mmol, 4 equiv.), Et_3SiH (240 µL, 1.5 mmol, 3 equiv.) and anhydrous 1,4-dioxane (5 mL). The crude was purified by column chromatography (100% hexanes) affording cyclopropane-1,1-diyldibenzene **38** as a colourless oil (72.4 mg, 75%). Analytical data were in agreement with the corresponding data reported previously.

Reaction of (3-(phenylsulfinyl)propane-1,1-diyl)dibenzene 53

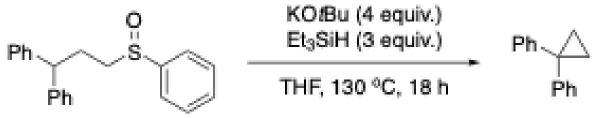

55 38 70%

The reaction was carried out according to general procedure I using (3-(phenylsulfinyl)propane-1,1-diyl)dibenzene **55** (160 mg, 0.5 mmol, 1 equiv.), KO*t*Bu (224 mg, 2 mmol, 4 equiv.), Et_3SiH (240 µL, 1.5 mmol, 3 equiv.) and anhydrous THF (5 mL). The crude was further reacted with *m*-CPBA (235 mg, 1.4 mmol) for 16 hours then purified via column chromatography (100% hexane) affording cyclopropane-1,1-diyldibenzene **38** as a colourless oil (68 mg, 70%). Analytical data were in agreement with the corresponding data reported previously.

Reaction of (3,3-diphenylpropyl)(*p*-tolyl)sulfane 56

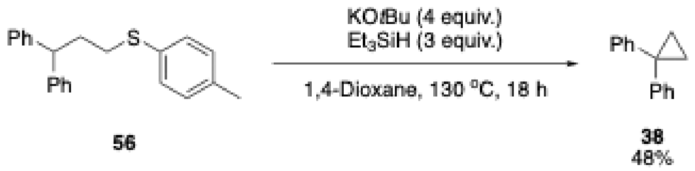

The reaction was carried out according to general procedure I using (3,3-diphenylpropyl)(*p*-tolyl)sulfane **56** (159 mg, 0.5 mmol, 1 equiv.), KO*t*Bu (224 mg, 2 mmol, 4 equiv.), Et_3SiH (240 µL, 1.5 mmol, 3 equiv.) and anhydrous 1,4-dioxane (5 mL). The crude was purified via column chromatography (100% hexane) affording cyclopropane-1,1-diyldibenzene **38** as a colourless oil (47 mg, 48%). Analytical data were in agreement with the corresponding data reported previously.

Reaction of (3-(*p*-tolylsulfinyl)propane-1,1-diyl)dibenzene 57

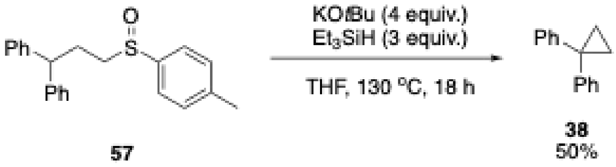

The reaction was carried out according to general procedure I using (3-(*p*-tolylsulfinyl)propane-1,1-diyl)dibenzene **57** (175 mg, 0.5 mmol, 1 equiv.), KO*t*Bu (224 mg, 2 mmol, 4 equiv.), Et_3SiH (240 µL, 1.5 mmol, 3 equiv.) and anhydrous THF (5 mL). The crude was further reacted with *m*-CPBA (235 mg, 1.4 mmol) for 16 hours then purified via column chromatography (100% hexanes) affording cyclopropane-1,1-diyldibenzene **38** as a colourless oil (48 mg, 50%). Analytical data were in agreement with the corresponding data reported previously.

Reaction of (3-tosylpropane-1,1-diyl)dibenzene 58

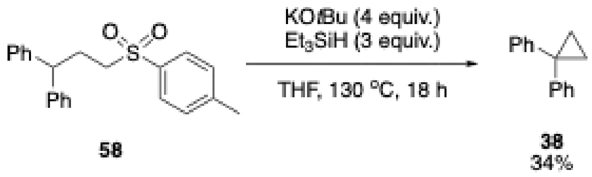

The reaction was carried out according to general procedure I using (3-tosylpropane-1,1-diyl)dibenzene **58** (175 mg, 0.5 mmol, 1 equiv.), KO*t*Bu (224 mg, 2 mmol, 4 equiv.), Et_3SiH

(240 μL, 1.5 mmol, 3 equiv.) and anhydrous THF (5 mL). The crude was purified via column chromatography (100% hexane) affording cyclopropane-1,1-diyldibenzene **38** in a mixture with inseparable impurities. The yield was determined using TCE (63.4 mg, 0.378 mmol) as an internal standard. TCE CH signal (5.96 ppm) was integrated to 2H. The cyclopropane $2xCH_2$ peak (1.32 ppm) was used to calculate the yield.

Cyclopropane-1,1-diyldibenzene **36** yield = $\frac{\left(\frac{2.041 \div 4}{2 \div 2}\right) \times 0.329mmol}{0.5mmol} \times 100 = 34\%$

Reaction of (3,3-diphenylpropyl)(methyl)sulfane 59

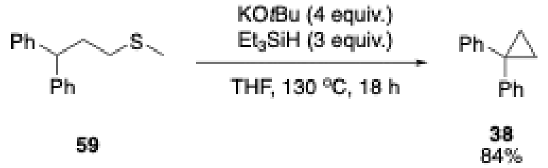

The reaction was carried out according to general procedure I using (3,3-diphenylpropyl)(methyl)sulfane **59** (121 mg, 0.5 mmol, 1 equiv.), KO*t*Bu (224 mg, 2 mmol, 4 equiv.), Et_3SiH (240 μL, 1.5 mmol, 3 equiv.) and anhydrous THF (5 mL). The crude was purified via column chromatography (100% hexane) affording cyclopropane-1,1-diyldibenzene **38** as a colourless oil (81 mg, 84%). Analytical data were in agreement with the corresponding data reported previously.

Reaction of (3-(phenylsulfonyl)propane-1,1-diyl)dibenzene 60

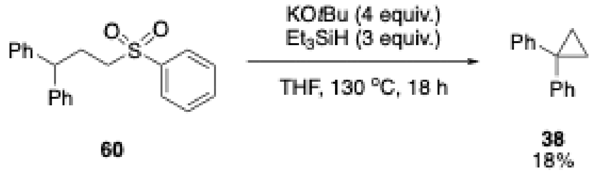

The reaction was carried out according to general procedure I using (3-(phenylsulfonyl)propane-1,1-diyl)dibenzene **60** (168 mg, 0.5 mmol, 1 equiv.), KO*t*Bu (224 mg, 2 mmol, 4 equiv.), Et_3SiH (240 μL, 1.5 mmol, 3 equiv.) and THF (5mL). The crude was purified via column chromatography (100% hexane) affording cyclopropane-1,1-diyldibenzene **38** as a colourless oil as a mixture with impurities. The yields for the compounds were determined using TCE (1,1,2,2-tetrachloroethane) (63.4 mg, 0.378 mmol) as an internal standard. TCE CH signal (5.96 ppm) was integrated to 2H. The cyclopropane CH_4 peak (1.31 ppm) was used to calculate the yields.

Cyclopropane-1,1-diyldibenzene **36** yield = $\frac{\left(\frac{0.977 \div 4}{2 \div 2}\right) \times 0.378mmol}{0.5mmol} \times 100 = 18\%$

Reaction of (3,3-diphenylpropyl)(4-(trifluoromethyl)phenyl)sulfane 61

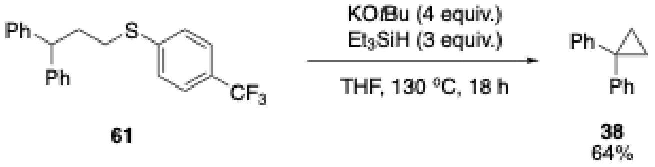

The reaction was carried out according to general procedure I using (3,3-diphenylpropyl)(4-(trifluoromethyl)phenyl)sulfane **61** (186 mg, 0.5 mmol, 1 equiv.), KO*t*Bu (224 mg, 2 mmol, 4 equiv.), Et_3SiH (240 µL, 1.5 mmol, 3 equiv.) and anhydrous THF (5 mL). The crude was purified via column chromatography (100% hexane) affording cyclopropane-1,1-diyldibenzene **38** as a colourless oil (62 mg, 64%). Analytical data were in agreement with the corresponding data reported previously.

Reaction of (3,3-diphenylpropyl)(4-methoxyphenyl)sulfane 62

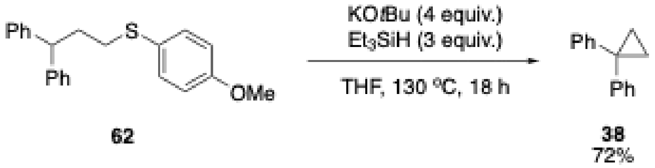

The reaction was carried out according to general procedure I using (3,3-diphenylpropyl)(4-methoxyphenyl)sulfane **62** (167 mg, 0.5 mmol, 1 equiv.), KO*t*Bu (224 mg, 2 mmol, 4 equiv.), Et_3SiH (240 µL, 1.5 mmol, 3 equiv.) and anhydrous THF (5 mL). The crude was purified via column chromatography (100% hexane) affording cyclopropane-1,1-diyldibenzene **38** as a colourless oil (69 mg, 72%). Analytical data were in agreement with the corresponding data reported previously.

Reaction of (3-((4-methoxyphenyl)sulfinyl)propane-1,1-diyl)dibenzene 63

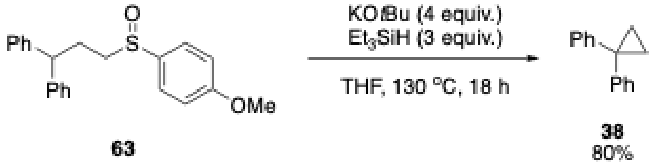

The reaction was carried out according to general procedure I using (3-((4-methoxyphenyl)sulfinyl)propane-1,1-diyl)dibenzene **63** (175 mg, 0.5 mmol, 1 equiv.), KO*t*Bu (224 mg, 2 mmol, 4 equiv.), Et_3SiH (240 µL, 1.5 mmol, 3 equiv.) and anhydrous THF (5 mL). The crude was further reacted with *m*-CPBA (235 mg, 1.4 mmol) for 16 h then purified via column chromatography (100% hexanes) affording cyclopropane-1,1-diyldibenzene **38** as a colourless oil (78 mg, 80%). Analytical data were in agreement with the corresponding data reported previously.

Reaction of (3-(4-(*tert*-butyl)phenyl)propyl)(*p*-tolyl)sulfane 64

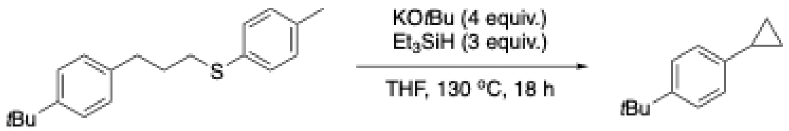

64

41
82%

The reaction was carried out according to general procedure I using (3-(4-(*tert*-butyl)phenyl)propyl)(*p*-tolyl)sulfane **64** (149 mg, 0.5 mmol, 1 equiv.), KO*t*Bu (224 mg, 2 mmol, 4 equiv.), Et_3SiH (240 µL, 1.5 mmol, 3 equiv.) and anhydrous THF (5 mL). The crude was purified via column chromatography (100% hexane) affording 1-(*tert*-butyl)-4-cyclopropylbenzene **41** as a colourless oil (71.1 mg, 82%). Analytical data were in agreement with the corresponding data reported previously.

Reaction of 2-(3-(*p*-tolylsulfinyl)propyl)naphthalene 65

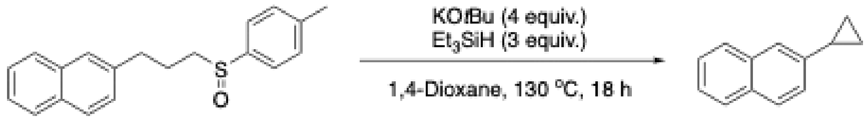

65

43
32%

The reaction was carried out according to general procedure I using 2-(3-(*p*-tolylsulfinyl)propyl)naphthalene **65** (159 mg, 0.5 mmol, 1 equiv.), KO*t*Bu (224 mg, 2 mmol, 4 equiv.), Et_3SiH (240 µL, 1.5 mmol, 3 equiv.) and dioxane (5 mL). The crude was purified by column chromatography (100% hexane) affording 2-cyclopropylnaphthalene **43** as a white gum (26.1 mg, 32%). Analytical data were in agreement with the corresponding data reported previously.

Reaction of 9-(2-(phenylthio)ethyl)-9*H*-xanthene 66

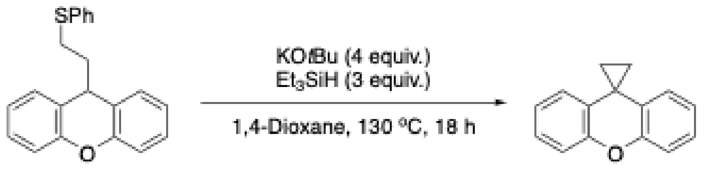

66

39
90%

The reaction was carried out according to general procedure I using 9-(2-(phenylthio)ethyl)-9*H*-xanthene **66** (159 mg, 0.5 mmol, 1 equiv.), KO*t*Bu (224 mg, 2 mmol, 4 equiv.), Et_3SiH (240 µL, 1.5 mmol, 3 equiv.) and anhydrous dioxane (5 mL). The crude material was purified via column chromatography (100% hexane) affording spiro[cyclopropane-1,9'-xanthene] **39** as a colourless oil (95 mg, 90%). Analytical data were in agreement with the corresponding data reported previously.

Exploration of 4-membered and 6-membered ring formation

Our examples show that cyclopentane and cyclopropane rings can be formed *via* the Grubbs-Stoltz reagent-mediated intramolecular deoxygenative alkylation.

Now we examined whether analogous 4- and 6-membered cyclobutane and cyclohexane products could be formed. As will be shown below, only trace amounts of cyclobutane **S64** were detected by GC-MS after the treatment of alcohol **S60** with the Grubbs-Stoltz reagent. The major product isolated from the reaction mixture was phenol **S61** in 21% yield, indicating that the reductive C-O cleavage is the predominant pathway accessible to substrate **S60**. Identical C-O bond cleavage was also observed upon exposure of substrate **S67** under the Grubbs-Stoltz reagent, where product **S69** was obtained in 19% yield. More surprisingly, fluorene product **S68** was also isolated in 9% yield, indicating that two consecutive reductive C-O cleavage events occur, ultimately producing **S68**. The simpler 6,6-diphenylhexanol starting material **S74** failed to afford significant amounts of product **S76** on treatment with Et_3SiH and KO^tBu. Silyl ether **S75** was also treated with the Grubbs-Stoltz reagent (not shown in scheme) but only trace amounts of product **S76** were detected in the crude mixture by GC-MS. Therefore, it was concluded that the formation of 4- and 6-membered rings is not favoured under the conditions of the Grubbs-Stoltz reagent.

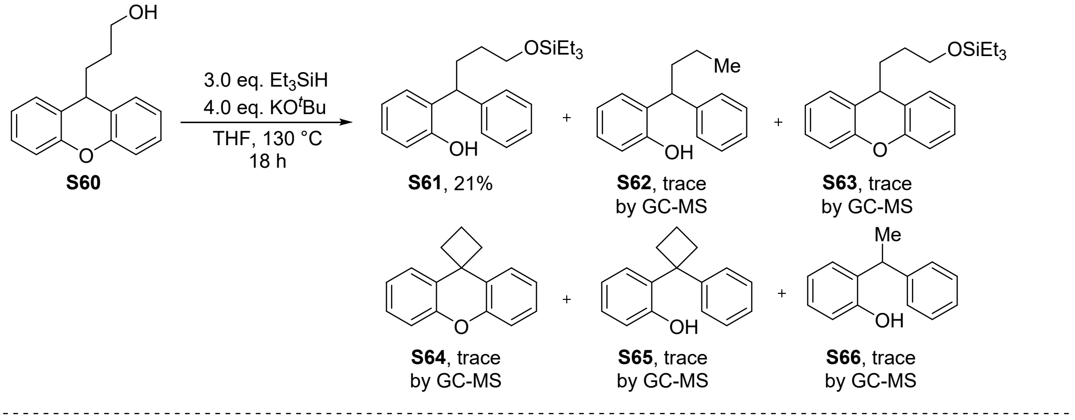

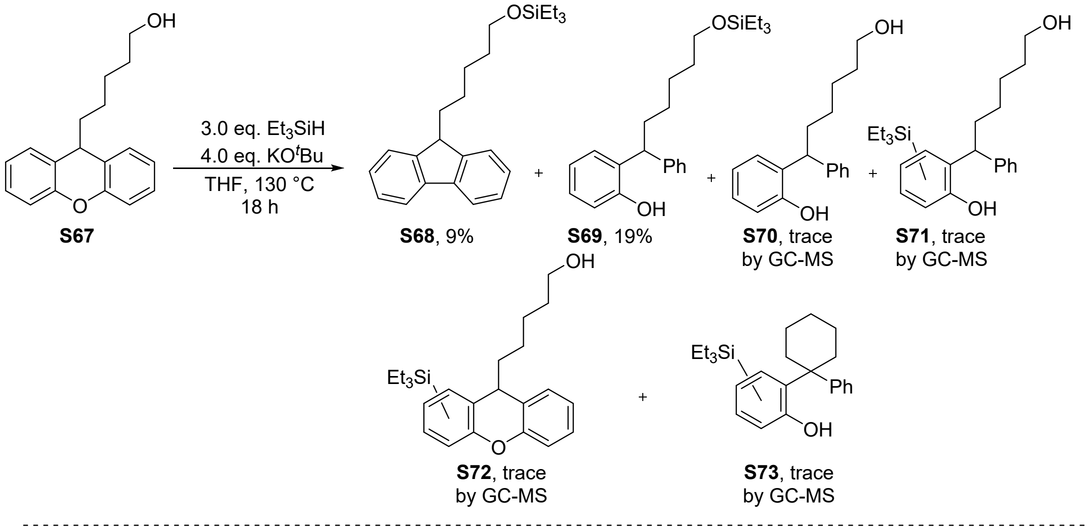

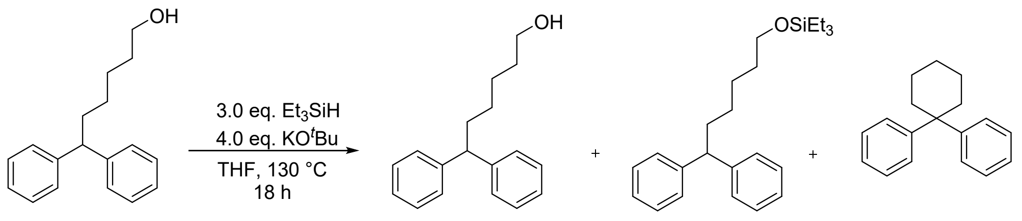

S74 **S74**, 31% **S75**, 60% **S76**, trace by GC-MS

Scope of the intramolecular alkylation for the construction of 4- and 6-membered cycloalkane rings

Preparation of 3-(9*H*-xanthen-9-yl)propan-1-ol (**S60**)

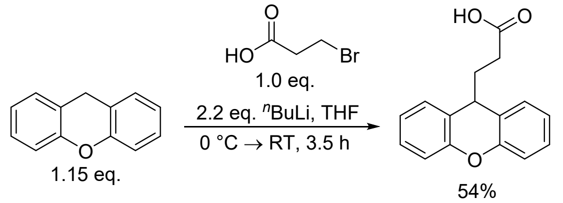

3-(9*H*-Xanthen-9-yl)propanoic acid was prepared according to a modified patent procedure[39] ^{n}BuLi in hexanes (1.6 M, 2.2 eq., 22.0 mmol, 13.8 mL) was added to a stirred solution of 9*H*-xanthene (1.15 eq., 11.5 mmol, 2.096 g) in dry THF (60 mL) under argon at 0 ℃. The reaction mixture was stirred at 0 ℃ for ~10 min before a solution of 4-bromopropionic acid (1.0 eq., 10.0 mmol, 1.530 g) in dry THF (10 mL) was added. The reaction mixture was stirred at RT for 3.5 h before it was quenched with sat. NH_4Cl at 0 ℃ and pH was adjusted to 3-4 with 6 M HCl. The volatile organic components of the mixture were evaporated under reduced pressure and the resulting aqueous residue was extracted with Et_2O and CH_2Cl_2 (2 x). The combined organic phases were concentrated, redissolved in Et_2O and basified with 1M NaOH until pH = 11-12. The aqueous phase was separated, washed with Et_2O, acidified with 6 M HCl until pH = 1-2 and extracted with CH_2Cl_2. The organic phase was concentrated, affording the crude 3-(9*H*-xanthen-9-yl)propanoic acid product (1.365 g, 54%) as a viscous yellow oil that was used in the next step without any further purification. **^{1}H NMR** (400 MHz, $CDCl_3$) δ 7.25 – 7.19 (m, 4H, 4 x ArH), 7.14 – 7.04 (m, 4H, 4 x ArH), 4.11 (t, *J* = 5.8 Hz, 1H, CH), 2.25 – 2.14 (m, 2H, CH_2), 2.15 – 2.00 (m, 2H, CH_2). **^{13}C NMR** (101 MHz, $CDCl_3$) δ 178.9, 152.4, 128.7, 128.1, 124.2, 123.5, 116.7, 37.9, 35.1, 29.9. **ATR-IR** ν_{max} (neat)/cm^{-1} 3038, 2926, 2859, 1701, 1601, 1574, 1477, 1454, 1414, 1348, 1323, 1300, 1250, 1209, 1188, 1155, 1096, 1030, 957, 935, 889, 824, 779, 750, 714, 671, 644, 611. ***m/z* (ESI):** 253.2 ([M-H]$^-$). **HRMS** (ESI) calcd for $C_{16}H_{14}O_3Na^+$ ([M+Na]$^+$): 277.0835, found: 277.0826.

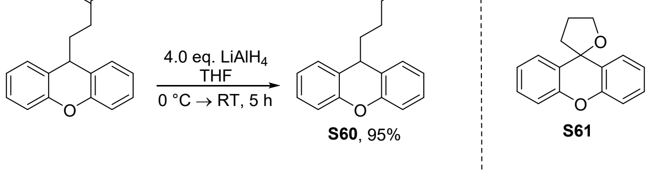

S60, 95% **S61**

Carried out according to **General Procedure A** using 3-(9*H*-xanthen-9-yl)propanoic acid (1.0 eq., 5.0 mmol, 1.260 g), $LiAlH_4$ (4.0 eq., 20.0 mmol, 0.759 g) and THF (35 mL). The reaction mixture was carefully quenched with water (0.8 mL), 2 M NaOH (1.5 mL) and water (1.5 mL). The crude 3-(9*H*-xanthen-9-yl)propan-1-ol **S60** (1.146 g, 95%) was isolated as a viscous yellow oil and was used in the next step without any further purification. **^{1}H NMR** (400 MHz, $CDCl_3$) δ 7.25 – 7.17 (m, 4H, 4 x ArH), 7.14 – 7.02 (m, 4H, 4 x ArH), 4.06 (t, *J* = 5.8 Hz, 1H,

CH), 3.50 (t, *J* = 6.6 Hz, 2H, OCH_2), 1.89 – 1.75 (m, 2H, CH_2), 1.51 – 1.36 (m, 2H, CH_2). **^{13}C NMR** (101 MHz, $CDCl_3$) δ 152.4, 128.7, 127.8, 125.3, 123.3, 116.5, 63.0, 38.8, 36.9, 28.7. **ATR-IR** $ν_{max}$ (neat)/cm^{-1} 3267, 3069, 3040, 2932, 2901, 2868, 2849, 1599, 1574, 1477, 1456, 1369, 1346, 1319, 1300, 1252, 1217, 1192, 1152, 1121, 1098, 1061, 1032, 986, 974, 937, 928, 899, 885, 862, 824, 812, 777, 748, 716, 675, 631, 613. ***m/z* (EI):** 240.2 (M^+, 36%), 221.2 (7), 205.2 (7), 194.2 (15), 181.2 (100), 165.2 (21), 152.2 (90), 139.2 (7), 127.2 (25), 115.2 (8), 102.2 (7), 89.1 (7), 77.2 (17), 63.1 (18), 51.2 (15). **HRMS** (ESI) calcd for $C_{16}H_{15}O_2^+$ ([M-H]$^+$): 239.1067, found: 239.1060.

When a test reaction was carried out on 0.5 mmol of 3-(9*H*-xanthen-9-yl)propanoic acid under the conditions described above, 3-(9*H*-xanthen-9-yl)propan-1-ol **S60** (67 mg, 56%) was isolated after purification by column chromatography (hexane → 50% EtOAc/50% hexane) along with 4,5-dihydro-3*H*-spiro[furan-2,9'-xanthene] **S61**(18 mg, 15%), which was isolated as a white solid. **Mp** = 62-64 ℃ (lit. mp. 83-84 ℃),[40] **^{1}H NMR** (400 MHz, $CDCl_3$) δ 7.59 – 7.47 (m, 2H, 2 x ArH), 7.33 – 7.23 (m, 2H, 2 x ArH), 7.20 – 7.10 (m, 4H, 4 x ArH), 4.44 (t, *J* = 6.5 Hz, 2H, OCH_2), 2.27 – 2.15 (m, 2H, CH_2), 2.15 – 2.05 (m, 2H, CH_2). **^{13}C NMR** (101 MHz, $CDCl_3$) δ 150.6, 129.4, 128.3, 125.5, 123.5, 116.3, 78.6, 71.4, 45.3, 26.0. **ATR-IR** $ν_{max}$ (neat)/cm^{-1} 3073, 3034, 2978, 2928, 2857, 1597, 1572, 1483, 1470, 1449, 1314, 1298, 1277, 1240, 1200, 1177, 1159, 1103, 1080, 1047, 1028, 978, 943, 910, 874, 845, 810, 764, 745, 714, 669, 635, 623. ***m/z* (EI):** 238.2 (M^+, 36%), 207.2 (57), 196.1 (100), 181.2 (21), 168.2 (67), 152.2 (23), 139.2 (53), 126.2 (5), 115.2 (10), 105.2 (4), 89.2 (8), 76.2 (11), 63.1 (15), 50.1 (10). **HRMS** (ESI) calcd for $C_{16}H_{15}O_2^+$ ([M+H]$^+$): 239.1067, found: 239.1061.

Preparation of 5-(9*H*-xanthen-9-yl)pentan-1-ol (**S68**)

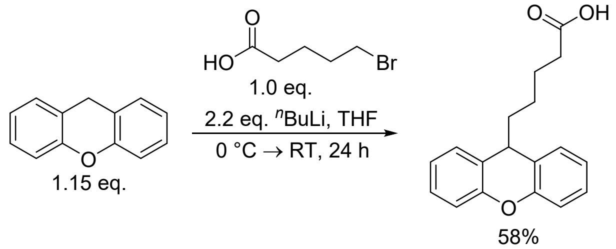

Carried out according to **General Procedure B** using 9*H*-xanthene (1.15 eq., 11.5 mmol, 2.096 g), ^{n}BuLi in hexanes (2 M, 2.2 eq., 22.0 mmol, 5 mL + 6 mL), 5-bromopentanoic acid (1.0 eq., 10.0 mmol, 1.810 g) and dry THF (65 mL). The crude 5-(9*H*-xanthen-9-yl)pentanoic acid product (1.646 g, 58%) was isolated as a yellow oil that was used in the next step without any further purification. **^{1}H NMR** (400 MHz, $CDCl_3$) δ 7.24 – 7.15 (m, 4H, 4 x ArH), 7.12 – 7.03 (m, 4H, 4 x ArH), 4.00 (t, *J* = 6.0 Hz, 1H, CH), 2.24 (t, *J* = 7.6 Hz, 2H, CH_2), 1.82 – 1.64 (m, 2H, CH_2), 1.58 – 1.48 (m, 2H, CH_2), 1.28 – 1.15 (m, 2H, CH_2). **^{13}C NMR** (101 MHz, $CDCl_3$) δ 179.1, 152.4, 128.7, 127.7, 125.5, 123.3, 116.5, 40.4, 39.0, 33.8, 25.1, 24.7. **ATR-IR** $ν_{max}$ (neat)/cm^{-1} 3075, 3028, 2934, 2864, 1703, 1655, 1616, 1605, 1576, 1477, 1456, 1412, 1344, 1331, 1304, 1250, 1240, 1213, 1180, 1146, 1119, 1099, 1080, 1057, 1032, 1024, 966, 934, 881, 843, 827, 806, 754, 704, 669, 656, 638, 627, 615. **HRMS** (ESI) calcd for $C_{18}H_{17}O_3^-$ ([M-H]$^-$): 281.1172, found: 281.1185.

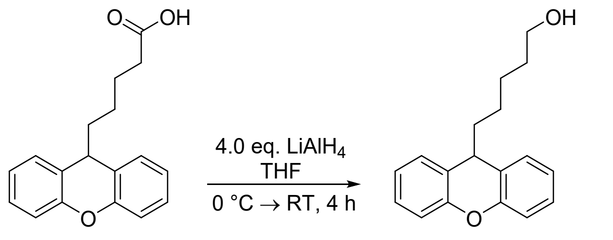

S68, 100%

Carried out according to **General Procedure A** using 5-(9*H*-xanthen-9-yl)pentanoic acid (1.0 eq., 5.8 mmol, 1.646 g), $LiAlH_4$ (4.0 eq., 23.3 mmol, 0.885 g) and THF (40 mL). The reaction mixture was carefully quenched with water (1.0 mL), 2 M NaOH (2.0 mL) and water (2.0 mL). The crude 5-(9*H*-xanthen-9-yl)pentan-1-ol **S68** (1.560 g, 100%) was isolated as a viscous yellow oil and was used in the next step without any further purification. **^{1}H NMR** (400 MHz, $CDCl_3$) δ 7.24 – 7.17 (m, 4H, 4 x ArH), 7.10 – 7.02 (m, 4H, 4 x ArH), 3.99 (t, *J* = 6.0 Hz, 1H, CH), 3.53 (t, *J* = 6.6 Hz, 2H, OCH_2), 1.78 – 1.69 (m, 2H, CH_2), 1.52 – 1.40 (m, 2H, CH_2), 1.30 – 1.15 (m, 4H, 2 x CH_2). **^{13}C NMR** (101 MHz, $CDCl_3$) δ 152.4, 128.7, 127.6, 125.7, 123.2, 116.5, 63.0, 40.8, 39.1, 32.7, 25.8, 25.3. **ATR-IR** ν_{max} (neat)/cm^{-1} 3335, 3069, 3040, 2930, 2857, 1599, 1576, 1477, 1456, 1414, 1381, 1337, 1298, 1250, 1213, 1184, 1152, 1119, 1098, 1072, 1051, 1032, 995, 968, 937, 891, 856, 841, 814, 750, 669, 633, 617. **HRMS** (ESI) calcd for $C_{18}H_{19}O_2^-$ ([M-H]$^-$): 267.1391, found: 267.1390.

Preparation of 6,6-diphenylhexan-1-ol (**S75**)

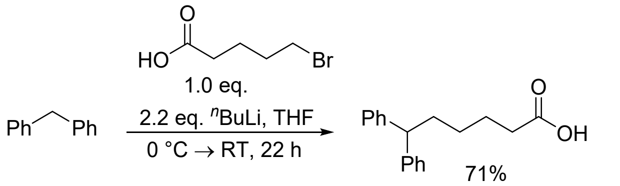

Carried out according to **General Procedure B** using diphenylmethane (1.15 eq., 11.5 mmol, 1.935 g), ^{n}BuLi in hexanes (2 M, 2.2 eq., 22.0 mmol, 5 mL + 6 mL), 5-bromopentanoic acid (1.0 eq., 10.0 mmol, 1.810 g) and dry THF (50 mL). The crude 6,6-diphenylhexanoic acid product (1.916 g, 71%) was isolated as a grey solid that was used in the next step without any further purification. **Mp** = 75-78 ℃ (no lit. mp.) **^{1}H NMR** (400 MHz, $CDCl_3$) δ 7.34 – 7.20 (m, 8H, 8 x ArH), 7.20 – 7.12 (m, 2H, 2 x ArH), 3.89 (t, *J* = 7.8 Hz, 1H, CH), 2.31 (t, *J* = 7.5 Hz, 2H, CH_2), 2.07 (q, *J* = 7.8 Hz, 2H, CH_2), 1.74 – 1.59 (m, 2H, CH_2), 1.39 – 1.26 (m, 2H, CH_2). **^{13}C NMR** (101 MHz, $CDCl_3$) δ 179.0, 145.1, 128.6, 128.0, 126.3, 51.3, 35.4, 33.8, 27.6, 24.8. **ATR-IR** ν_{max} (neat)/cm^{-1} 3082, 3053, 3022, 2999, 2938, 2913, 2868, 2855, 2631, 1705, 1599, 1578, 1493, 1464, 1450, 1445, 1425, 1406, 1360, 1339, 1314, 1292, 1279, 1269, 1236, 1202, 1184, 1138, 1096, 1082, 1063, 1053, 1030, 943, 914, 903, 820, 775, 750, 733, 696, 679, 633, 617. ***m/z*** (ESI+APCI): 267.1 ([M-H]$^-$). **HRMS** (ESI) calcd for $C_{18}H_{19}O_2^-$ ([M-H]$^-$): 267.1391, found: 267.1393.

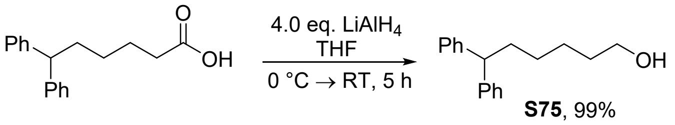

S75, 99%

Carried out according to **General Procedure A** using 6,6-diphenylhexanoic acid (1.0 eq., 7.14 mmol, 1.916 g), $LiAlH_4$ (4.0 eq., 28.6 mmol, 1.084 g) and THF (51 mL). The reaction mixture was carefully quenched with water (1.2 mL), 2M NaOH (2.5 mL) and water (2.5 mL). The crude 6,6-diphenylhexan-1-ol (1.805 g, 99%) **S75** was isolated as a colourless oil and was used in the next step without any further purification. **^{1}H NMR** (400 MHz, $CDCl_3$) δ 7.31 – 7.20 (m, 8H,

8 x ArH), 7.20 – 7.13 (m, 2H, 2 x ArH), 3.89 (t, *J* = 7.8 Hz, 1H, CH), 3.60 (t, *J* = 6.5 Hz, 2H, OCH_2), 2.06 (q, *J* = 7.8 Hz, 2H, CH*CH_2*), 1.63 – 1.46 (m, 2H, CH_2), 1.44 – 1.35 (m, 2H, CH_2), 1.35 – 1.24 (m, 2H, CH_2). **^{13}C NMR** (101 MHz, $CDCl_3$) δ 145.3, 128.5, 128.0, 126.2, 63.1, 51.5, 35.8, 32.8, 28.0, 25.9. **ATR-IR** ν_{max} (neat)/cm^{-1} 3350, 3269, 3082, 3059, 3024, 2930, 2857, 1599, 1582, 1493, 1450, 1308, 1184, 1155, 1067, 1051, 1030, 1003, 962, 910, 887, 843, 766, 746, 696, 631, 619. ***m/z* (EI):** 254.3 (M^+, 58%), 236.3 (27), 193.2 (29), 180.2 (30), 165.3 (100), 152.2 (66), 139.2 (18), 128.2 (25), 115.2 (51), 103.2 (27), 91.2 (53), 77.2 (39), 65.2 (21), 51.2 (27). **HRMS** (EI) calcd for $C_{18}H_{22}O^+$ (M^+): 254.1671, found: 254.1678.

Preparation of ((6,6-diphenylhexyl)oxy)triethylsilane (**S76**)

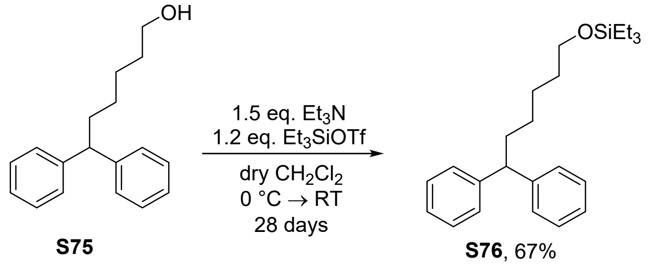

((6,6-Diphenylhexyl)oxy)triethylsilane **S76** was prepared according to a modified literature procedure.[41]` Et_3SiOTf (1.2 eq., 1.2 mmol, 271 μL) was added to a stirred solution of 6,6-diphenylhexan-1-ol **S75** (1.0 eq., 1.0 mmol., 254 mg) and Et_3N (1.5 eq., 1.5 mmol, 209 μL) in dry CH_2Cl_2 (2.5 mL) under argon at 0 ℃. The resulting mixture was stirred for 28 days before it was diluted with water (10 mL) and extracted with CH_2Cl_2 (3 x 20 mL). The combined organic phases were dried over $MgSO_4$, filtered and concentrated. Purification by column chromatography (hexane → 2% EtOAc/98% hexane → EtOAc) afforded ((6,6-diphenylhexyl)oxy)triethylsilane **S76** (247 mg, 67%) as a colourless oil. **^{1}H NMR** (400 MHz, $CDCl_3$) δ 7.33 – 7.21 (m, 8H, 8 x ArH), 7.20 – 7.12 (m, 2H, 2 x ArH), 3.88 (t, *J* = 7.8 Hz, 1H, CH), 3.56 (t, *J* = 6.5 Hz, 2H, OCH_2), 2.05 (q, 7.8 Hz, 2H, CH_2), 1.53 – 1.43 (m, 2H, CH_2), 1.42 – 1.32 (m, 2H, CH_2), 1.32 – 1.23 (m, 2H, CH_2), 0.95 (t, *J* = 7.9 Hz, 9H, 3 x Me), 0.58 (q, *J* = 7.9 Hz, 6H, 3 x $SiCH_2$). **^{13}C NMR** (101 MHz, $CDCl_3$) δ 145.4, 128.5, 128.0, 126.2, 63.0, 51.5, 35.9, 32.9, 28.0, 26.0, 6.9, 4.6. **ATR-IR** ν_{max} (neat)/cm^{-1} 3312, 3080, 3059, 3024, 3005, 2930, 2859, 1589, 1568, 1493, 1472, 1450, 1431, 1375, 1333, 1298, 1240, 1186, 1150, 1051, 1032, 1018, 993, 945, 910, 889, 841, 789, 746, 698, 650, 619. ***m/z* (EI):** 368.4 (M^+, 38%), 339.4 (79), 236.3 (59), 193.2 (38), 178.2 (12), 167.3 (100), 157.2 (58), 152.2 (73), 143.2 (73), 131.2 (23), 115.2 (77), 103.2 (51), 91.2 (82), 75.2 (71), 59.2 (34). **HRMS** (ESI) calcd for $C_{24}H_{37}OSi^+$ ($[M+H]^+$): 369.2608, found: 369.2597.

Treatment of 3-(9*H*-xanthen-9-yl)propan-1-ol (S60) with Et_3SiH and KO*^{t}*Bu in THF

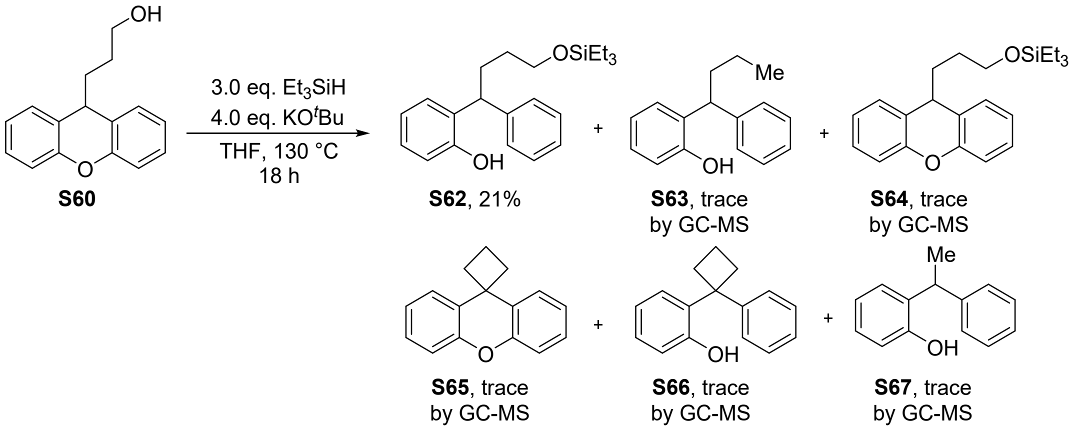

Carried out according to **General Procedure I** using 3-(9*H*-xanthen-9-yl)propan-1-ol **S60** (1.0 eq., 0.5 mmol, 120.2 mg), Et_3SiH (3.0 eq., 1.5 mmol, 240 µL), KO^tBu (4.0 eq., 2.0 mmol, 224 mg) and dry THF (5 mL). Purification by column chromatography (hexane → 10% EtOAc/90 % hexane) afforded 2-(1-phenyl-4-((triethylsilyl)oxy)butyl)phenol **S62** (38 mg, 21%) as a yellow oil. **^{1}H NMR** (400 MHz, $CDCl_3$) δ 7.36 – 7.23 (m, 4H, 4 x ArH), 7.23 – 7.16 (m, 1H, ArH), 7.13 (dd, *J* = 7.7, 1.5 Hz, 1H, ArH), 7.08 (td, *J* = 7.7, 1.7 Hz, 1H, ArH), 6.87 (td, *J* = 7.5, 1.1 Hz, 1H, ArH), 6.78 (dd, *J* = 8.0, 1.2 Hz, 1H, ArH), 5.54 (br s, 1H, OH), 4.32 (t, *J* = 7.7 Hz, 1H, CH), 3.79 – 3.62 (m, 2H, OCH_2), 2.19 – 2.06 (m, 2H, CH_2), 1.60 – 1.46 (m, 2H, CH_2), 0.96 (t, *J* = 7.9 Hz, 9H, 3 x Me), 0.62 (q, *J* = 7.9 Hz, 6H, 3 x $SiCH_2$). **^{13}C NMR** (101 MHz, $CDCl_3$) δ 153.8, 144.3, 131.3, 128.6, 128.3, 127.7, 127.4, 126.4, 120.9, 116.4, 63.4, 43.5, 31.9, 30.3, 6.9, 4.5. **ATR-IR** ν_{max} (neat)/cm^{-1} 3329, 3061, 3026, 2951, 2911, 2874, 1593, 1493, 1452, 1412, 1379, 1333, 1263, 1234, 1171, 1155, 1098, 1076, 1045, 1003, 976, 962, 910, 891, 843, 810, 746, 698, 673, 629. ***m/z* (EI):** 356.4 (M^+, 53%), 309.3 (5), 285.3 (58), 249.3 (71), 239.2 (74), 231.2 (68), 224.2 (63), 219.2 (32), 211.2 (22), 203.2 (28), 196.2 (71), 191.2 (48), 183.2 (100), 165.2 (95), 152.2 (46), 146.2 (21), 131.1 (100), 120.2 (64), 115.2 (100), 103.2 (59), 87.2 (97), 75.2 (86), 59.1 (68). **HRMS** (ESI) calcd for $C_{22}H_{32}O_2SiNa^+$ ($[M+Na]^+$): 379.2064, found: 379.2059.

(3-(9*H*-Xanthen-9-yl)propoxy)triethylsilane **S64** (RT 18.63 min, *m/z* 353.4), 2-(1-phenylbutyl) phenol **S63** (RT 14.07 min, *m/z* 226.2), 2-(1-phenylethyl)phenol **S67** (RT 13.25 min, *m/z* 198.2), spiro[cyclobutane-1,9'-xanthene] **S65** (RT 13.84 min, *m/z* 222.3) and 2-(1-phenylcyclobutyl)phenol **S65** (RT 13.73 min, *m/z* 224.3) were detected by GC-MS.

Treatment of 5-(9*H*-xanthen-9-yl)pentan-1-ol (S68) with Et_3SiH and KO^tBu in THF

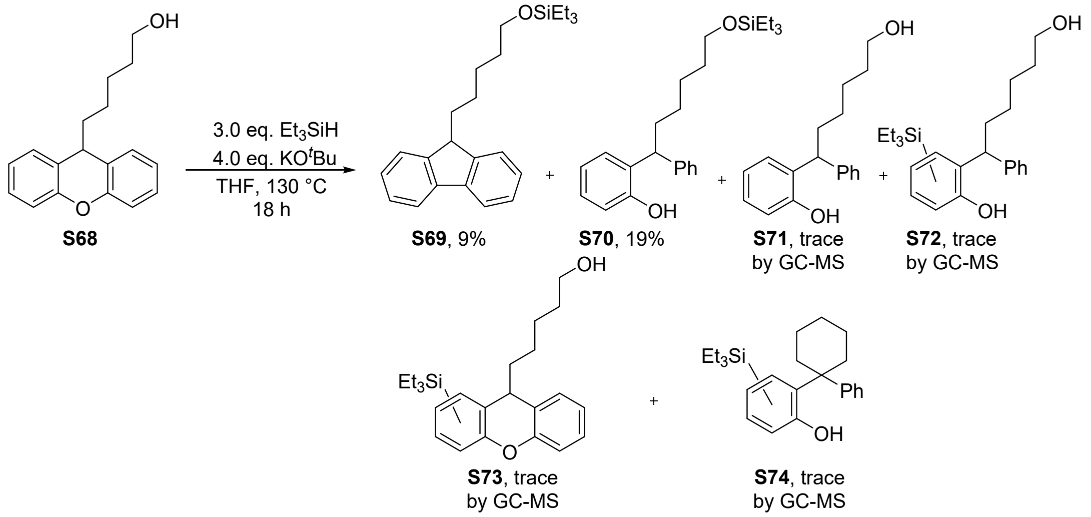

Carried out according to **General Procedure I** using 5-(9*H*-xanthen-9-yl)pentan-1-ol **S68** (1.0 eq., 0.5 mmol, 134 mg), Et_3SiH (3.0 eq., 1.5 mmol, 240 µL), KO^tBu (4.0 eq., 2.0 mmol, 224 mg) and dry THF (5 mL). Purification by column chromatography (hexane → 40% CH_2Cl_2/60% hexane) afforded ((5-(9*H*-fluoren-9-yl)pentyl)oxy)triethylsilane **S69** (16 mg, 9%) as a yellow oil and 2-(1-phenyl-6-((triethylsilyl)oxy)hexyl)phenol **S70** (37 mg, 19%) as a yellow oil.

((5-(9*H*-Fluoren-9-yl)pentyl)oxy)triethylsilane **S69**: **^{1}H NMR** (400 MHz, $CDCl_3$) δ 7.75 (d, *J* = 7.3 Hz, 2H, 2 x ArH), 7.51 (d, *J* = 7.6 Hz, 2H, 2 x ArH), 7.36 (t, *J* = 7.1 Hz, 2H, 2 x ArH), 7.30 (td, *J* = 7.4, 1.2 Hz, 2H, 2 x ArH), 3.98 (t, *J* = 5.9 Hz, 1H, CH), 3.53 (t, *J* = 6.6 Hz, 2H, OCH_2), 2.06 – 1.96 (m, 2H, CH_2), 1.51 – 1.39 (m, 2H, CH_2), 1.37 – 1.25 (m, 2H, CH_2), 1.23 – 1.16 (m, 2H, CH_2), 1.00 – 0.88 (m, 9H, 3 x Me), 0.61 – 0.52 (m, 6H, 3 x CH_2). **^{13}C NMR** (101 MHz, $CDCl_3$) δ 147.7, 141.3, 127.0, 126.9, 124.5, 119.9, 62.9, 47.6, 33.2, 32.8, 26.3, 25.5, 6.9, 4.6. **ATR-IR** ν_{max} (neat)/cm^{-1} 3063, 3038, 3017, 2949, 2932, 2911, 2874, 1609, 1570, 1477, 1449, 1412, 1387, 1344, 1302, 1238, 1215, 1152, 1096, 1005, 976, 976, 935, 916, 800, 785, 737, 669, 662, 621. ***m/z* (EI):** 366.4 (M^+, 3%), 337.3 (14), 234.3 (27), 191.2 (26), 178.2 (40), 165.2 (100), 143.3 (3), 115.2 (10), 75.2 (21), 59.1 (8). **HRMS** (ESI) calcd for $C_{22}H_{34}NOSi^+$ ($[M+H]^+$): 367.2452, found: 367.2449.

2-(1-Phenyl-6-((triethylsilyl)oxy)hexyl)phenol **S70**: **^{1}H NMR** (400 MHz, $CDCl_3$) δ 7.33 – 7.23 (m, 5H, 5 x ArH), 7.22 – 7.14 (m, 1H, ArH), 7.09 (td, *J* = 7.7, 1.7 Hz, 1H, ArH), 6.93 (td, *J* = 7.5, 1.2 Hz, 1H, ArH), 6.73 (dd, *J* = 8.0, 1.2 Hz, 1H, ArH), 4.74 (s, 1H, OH), 4.19 (t, *J* = 7.7 Hz, 1H, CH), 3.57 (t, *J* = 6.6 Hz, 2H, CH_2), 2.20 – 1.90 (m, 2H, CH_2), 1.57 – 1.45 (m, 2H, CH_2), 1.45 – 1.20 (m, 4H, 2 x CH_2), 0.95 (t, *J* = 7.9 Hz, 9H, 3 x Me), 0.58 (q, *J* = 8.1 Hz, 6H, 3 x $SiCH_2$). **^{13}C NMR** (101 MHz, $CDCl_3$) δ 153.6, 144.6, 131.4, 128.7, 128.2, 127.4, 126.4, 121.0, 116.1, 63.0, 44.3, 34.9, 32.9, 27.9, 26.0, 6.9, 4.6 (one aromatic signal missing due to overlap). **ATR-IR** ν_{max} (neat)/cm^{-1} 3296, 3061, 3026, 2949, 2934, 2913, 2874, 1595, 1495, 1452, 1414, 1379, 1333, 1263, 1236, 1171, 1155, 1096, 1057, 1003, 976, 916, 845, 806, 779, 746, 729, 698, 669, 631, 619, 610. ***m/z* (EI):** 384.5 (M^+, 60%), 355.4 (59), 337.4 (12), 277.3 (89), 259.3 (83), 239.2 (82), 209.2 (87), 183.3 (100), 165.2 (96), 143.2 (59), 115.2 (97), 91.2 (96), 75.2 (94), 59.2 (85). **HRMS** (ESI) calcd for $C_{24}H_{37}O_2Si^+$ ($[M+H]^+$): 385.2557, found: 385.2556.

2-(6-Hydroxy-1-phenylhexyl)phenol **S71** (RT 16.51 min, *m/z* 270.3), silylated 2-(1-phenyl-6-((triethylsilyl)oxy)hexyl)phenol **S72** (RT 17.90 min, *m/z* 384.4), silylated 5-(9*H*-xanthen-9-yl)pentan-1-ol **S73** (RT 22.14 min, *m/z* 381.4) and silylated 2-(1-phenylcyclohexyl)phenol **S74** (RT 18.54 min, *m/z* 366.4) were detected by GC-MS.

Treatment of 6,6-diphenylhexan-1-ol (S75) with Et_3SiH and KO^{t}Bu in THF

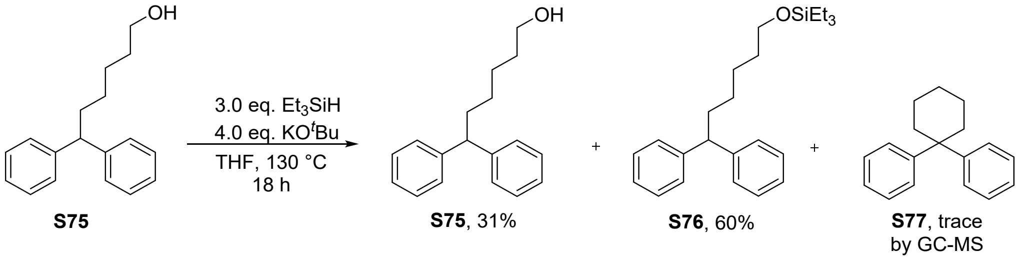

Carried out according to **General Procedure I** using 6,6-diphenylhexan-1-ol **S75** (1.0 eq., 0.5 mmol, 127.2 mg), Et_3SiH (3.0 eq., 1.5 mmol, 240 µL), KO^{t}Bu (4.0 eq., 2.0 mmol, 224 mg) and dry THF (5 mL). Purification by column chromatography (hexane → 3% EtOAc/ 97% hexane → EtOAc) afforded the 6,6-diphenylhexan-1-ol starting material **S75** (39 mg, 31%) as a colourless oil and ((6,6-diphenylhexyl)oxy)triethylsilane **S76** (110 mg, 60%) as a colourless oil. The analytical data for both compounds were consistent with the data outlined above.

Treatment of ((6,6-diphenylhexyl)oxy)triethylsilane (S75) with Et_3SiH and KO^{t}Bu in THF

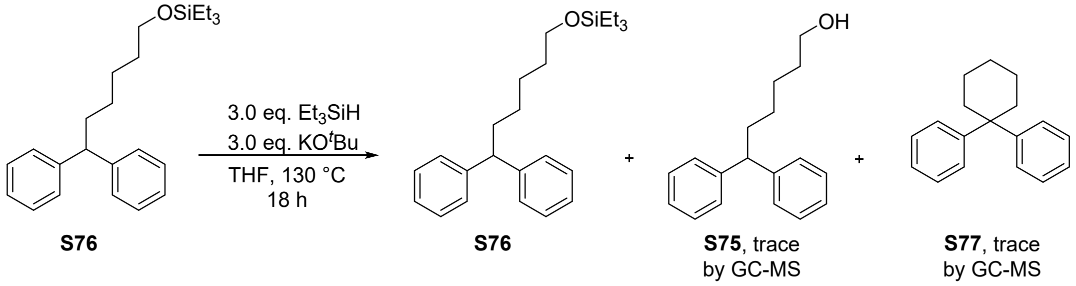

Carried out according to **General Procedure I** using ((6,6-diphenylhexyl)oxy)triethylsilane **S76** (1.0 eq., 0.5 mmol, 184 mg), Et_3SiH (3.0 eq., 1.5 mmol, 240 µL), KO^{t}Bu (3.0 eq., 1.5 mmol, 168 mg) and dry THF (5 mL). The reaction mixture was stirred at 130 ℃ for 36 h. The ((6,6-diphenylhexyl)oxy)triethylsilane starting material **S76** (RT 17.03 min, *m/z* 368.3) was the major component of the crude reaction mixture by ^{1}H NMR and GC-MS. Two minor products were also detected by GC-MS - 6,6-diphenylhexan-1-ol **S75** (RT 15.34 min, *m/z* 254.2) and cyclohexane-1,1-diyldibenzene **S77** (RT 14.48 min, *m/z* 236.1).

Appendix

N,2-Dimethyl-*N*-phenylaniline 11

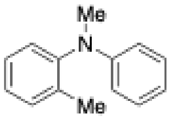

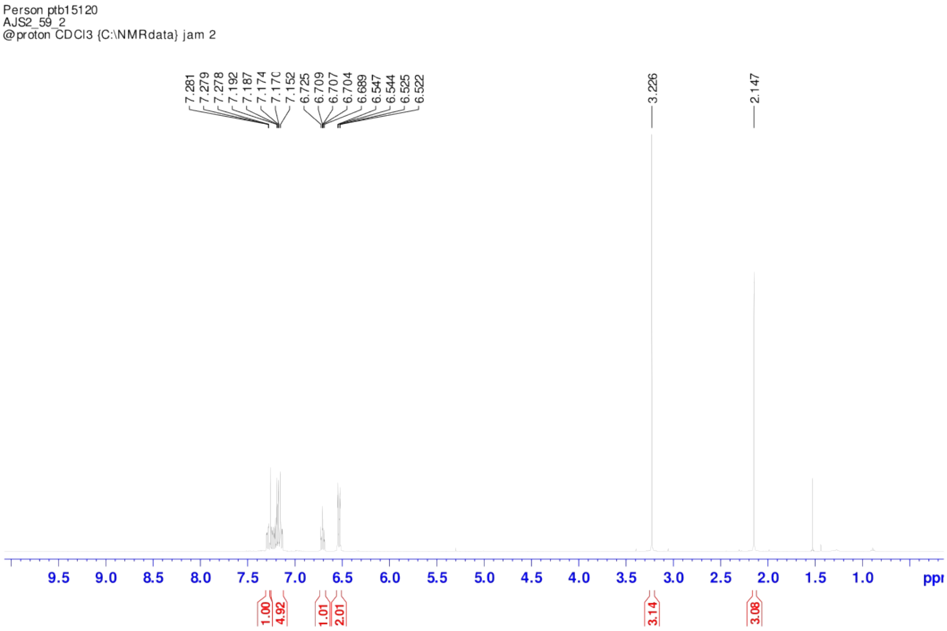

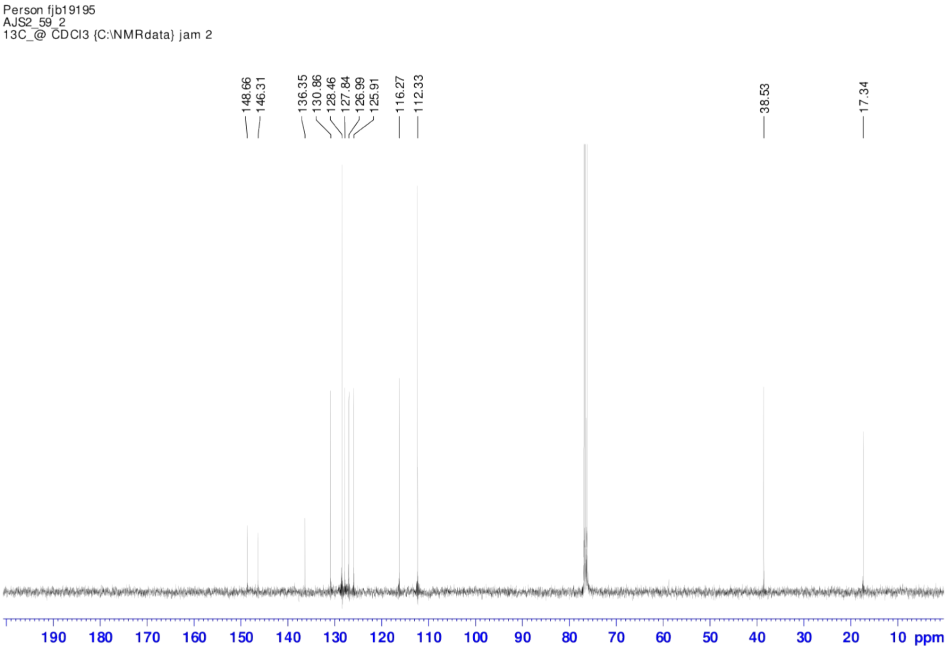

10-Methylacridin-10-ium iodide S4

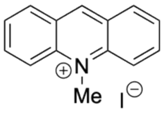

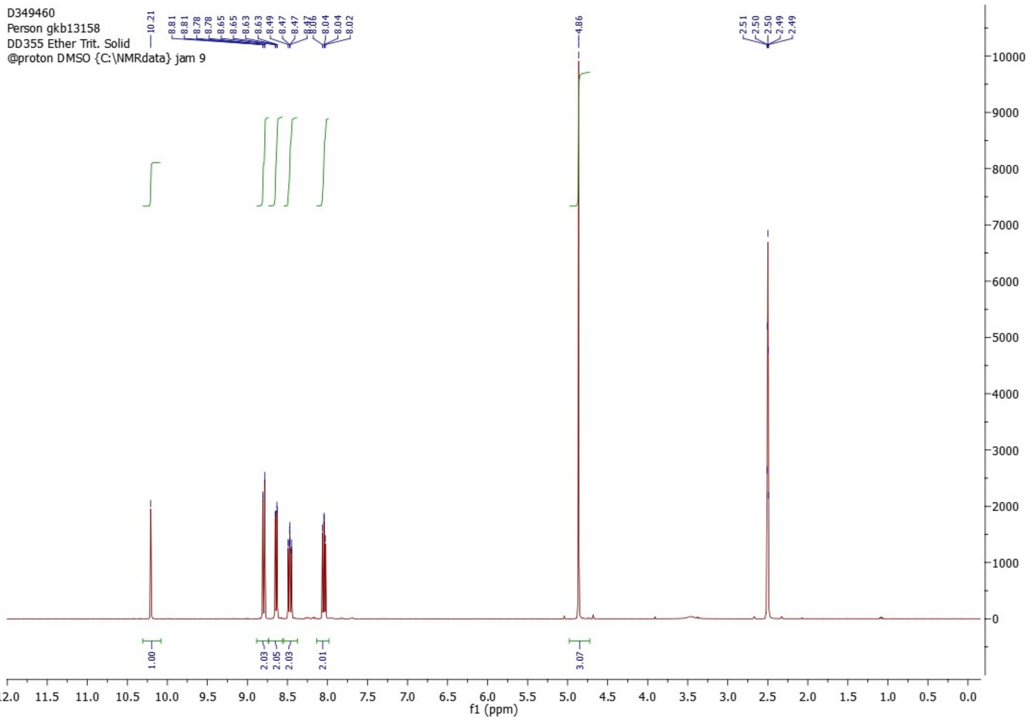

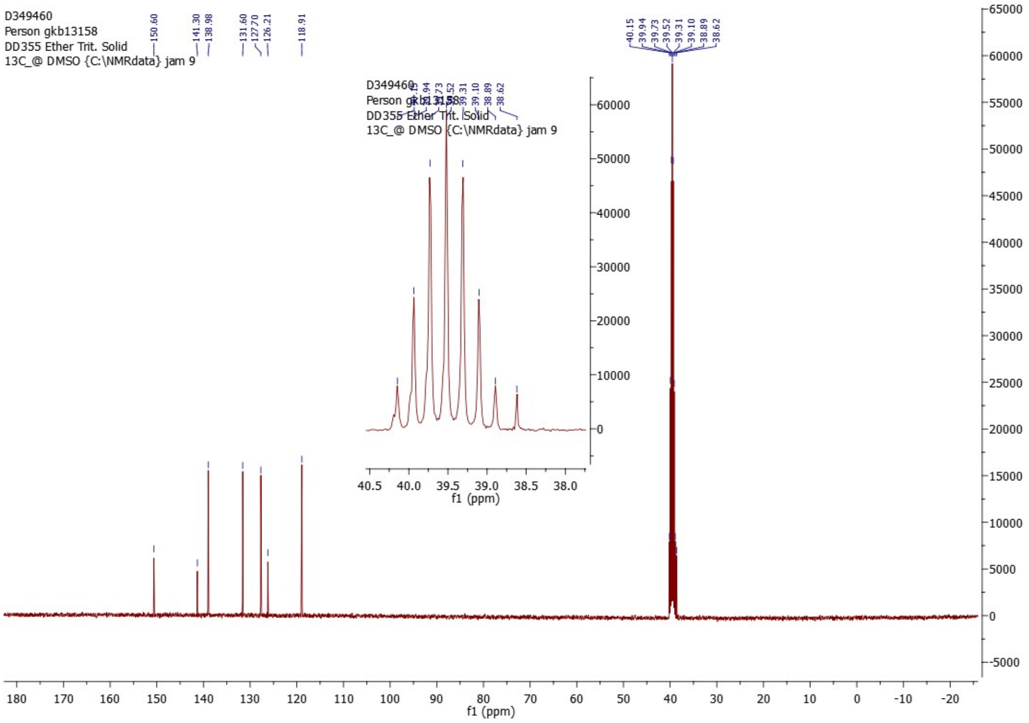

10-Methyl-9,10-dihydroacridine 13

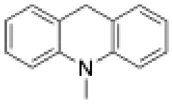

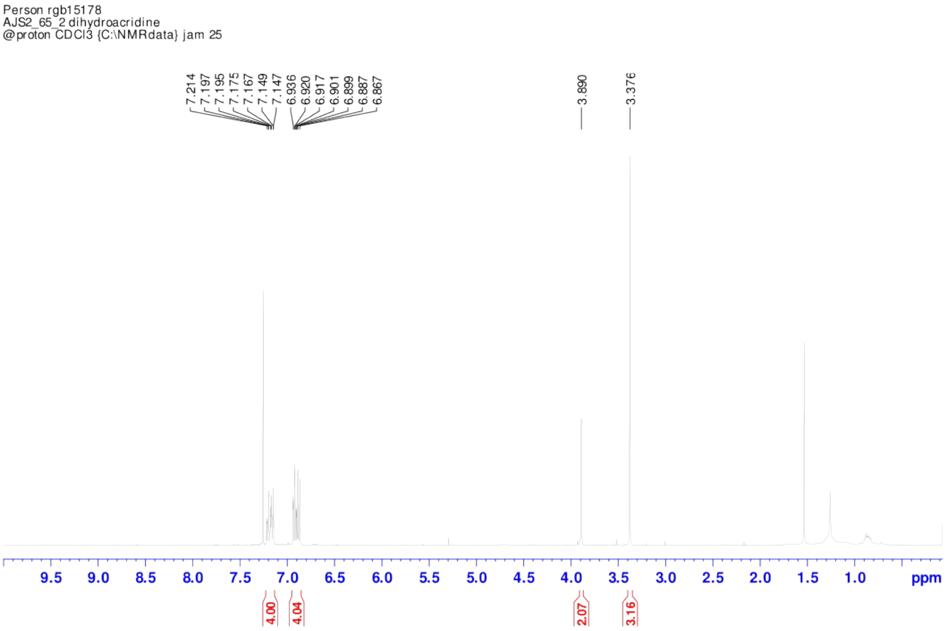

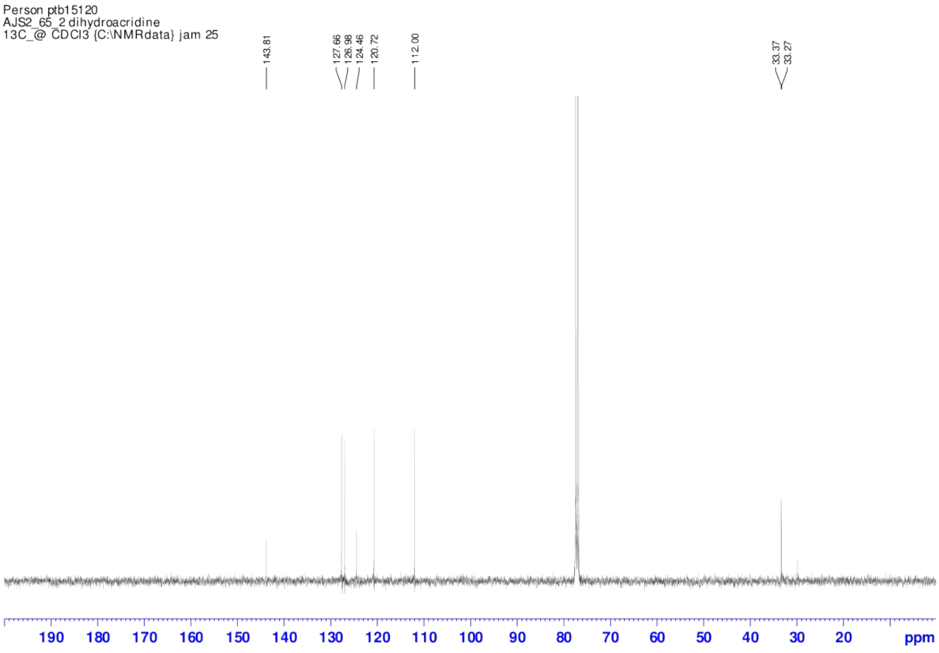

5,5-diphenylpentanoic acid S6

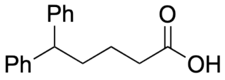

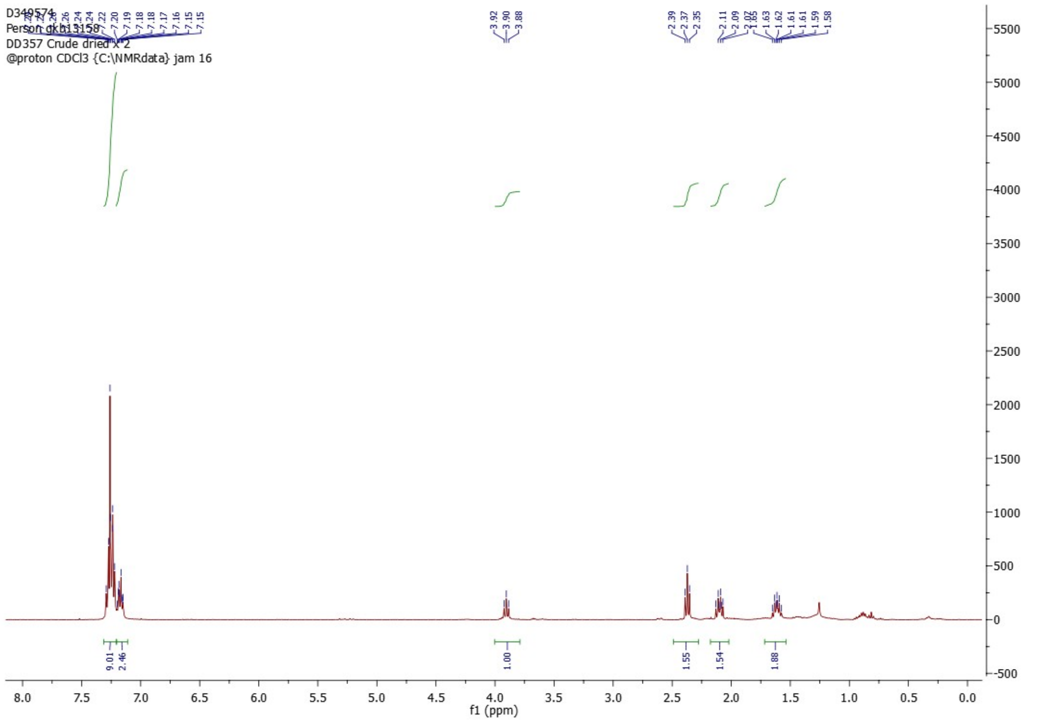

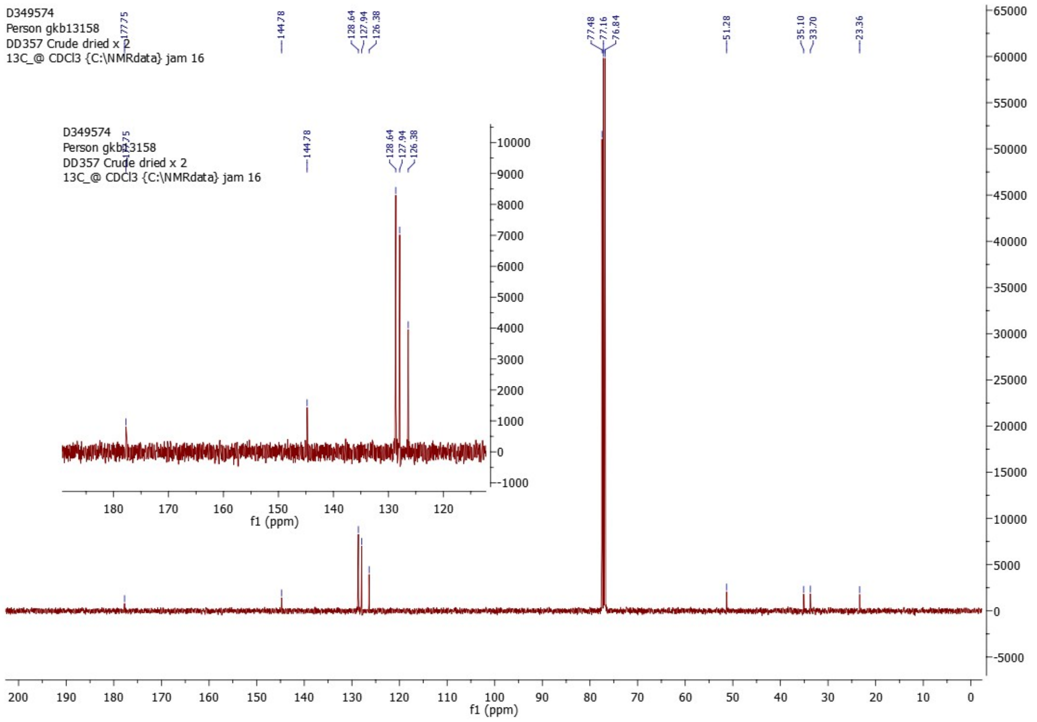

5,5-Diphenylpentan-1-ol 25

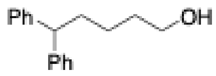

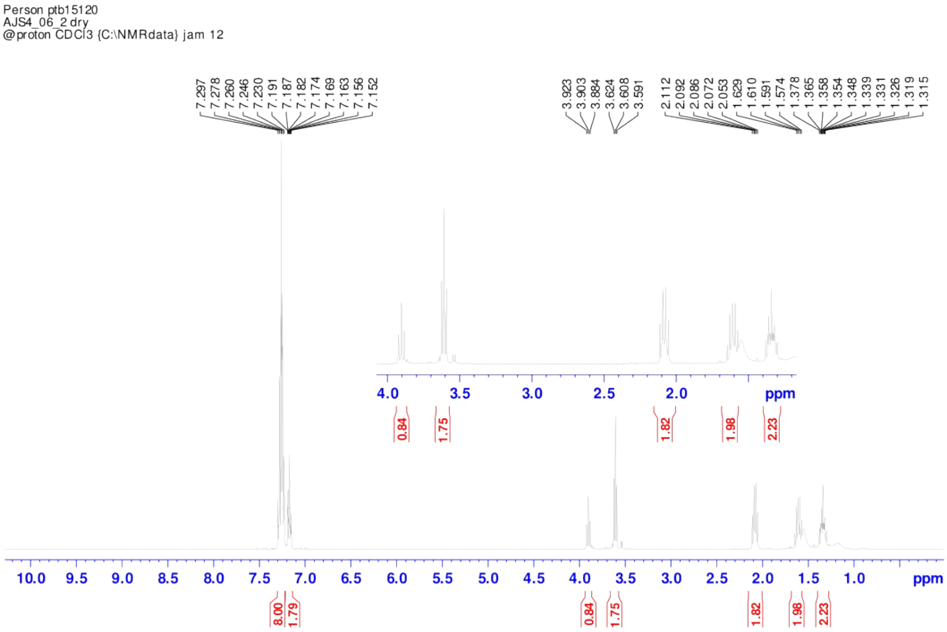

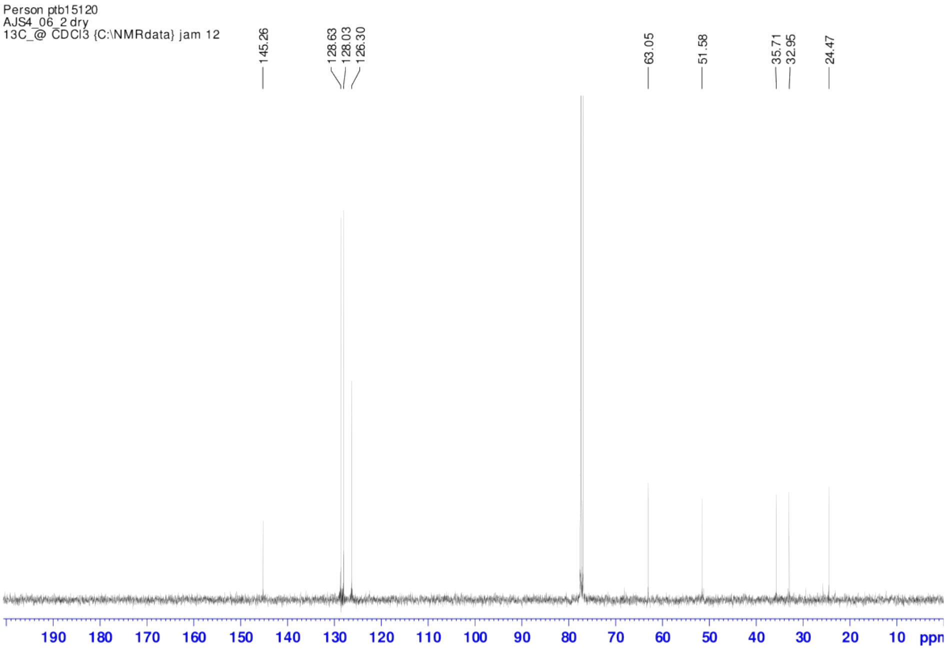

((5,5-diphenylpentyl)oxy)triethylsilane 26

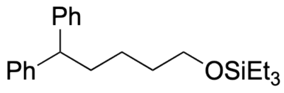

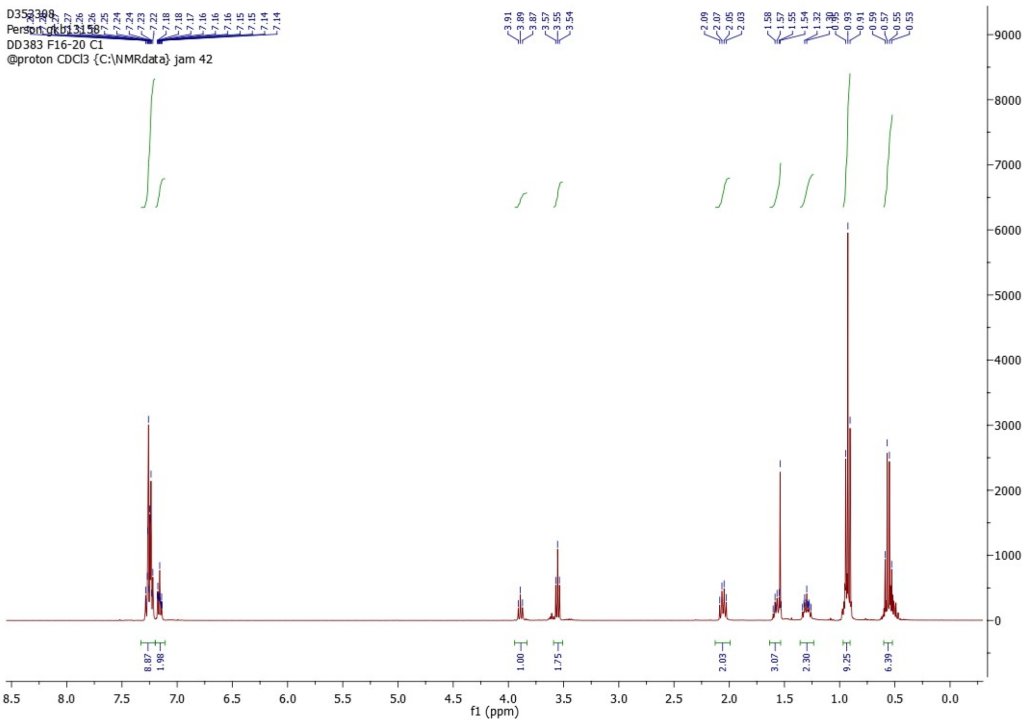

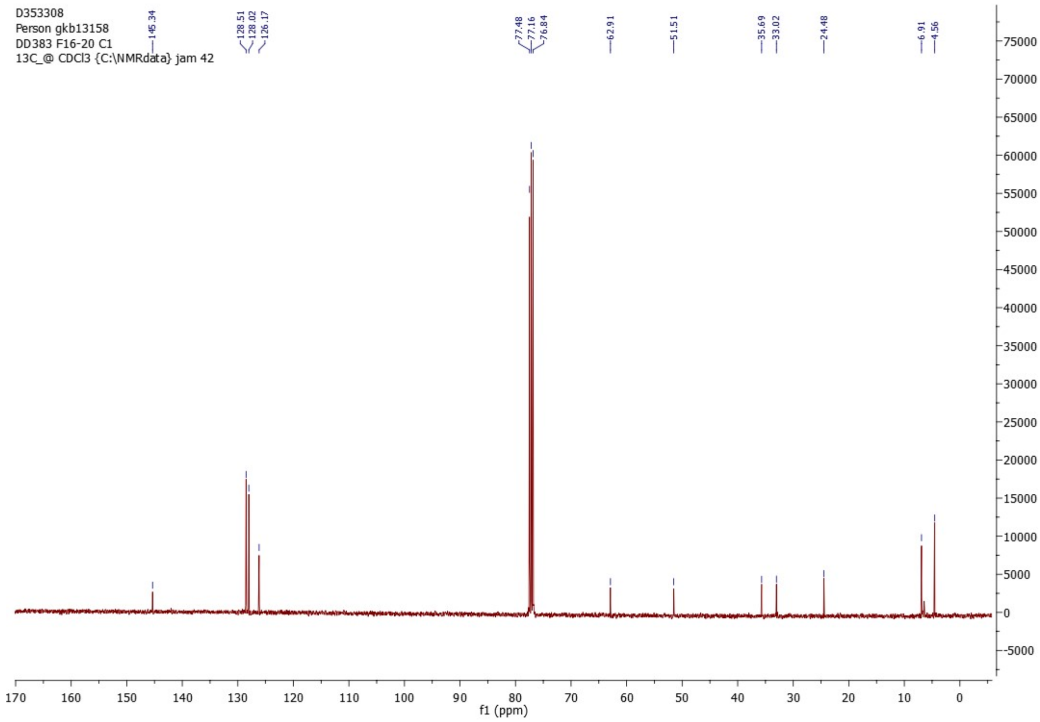

(5-Methoxypentane-1,1-diyl)dibenzene 27

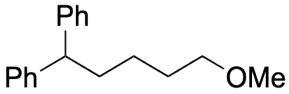

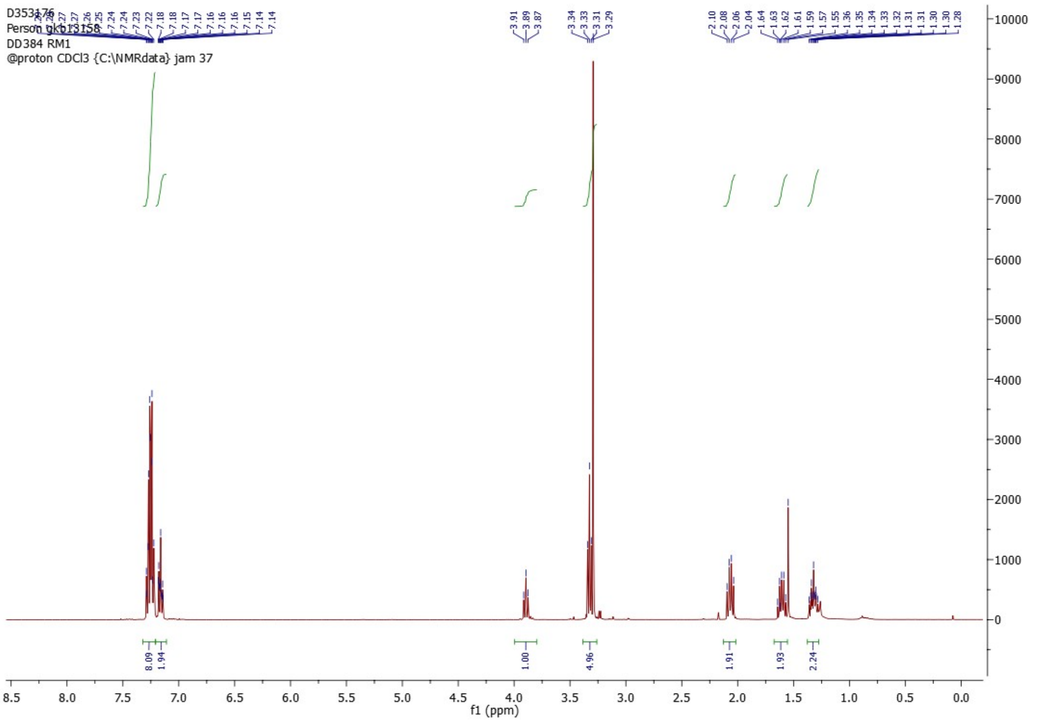

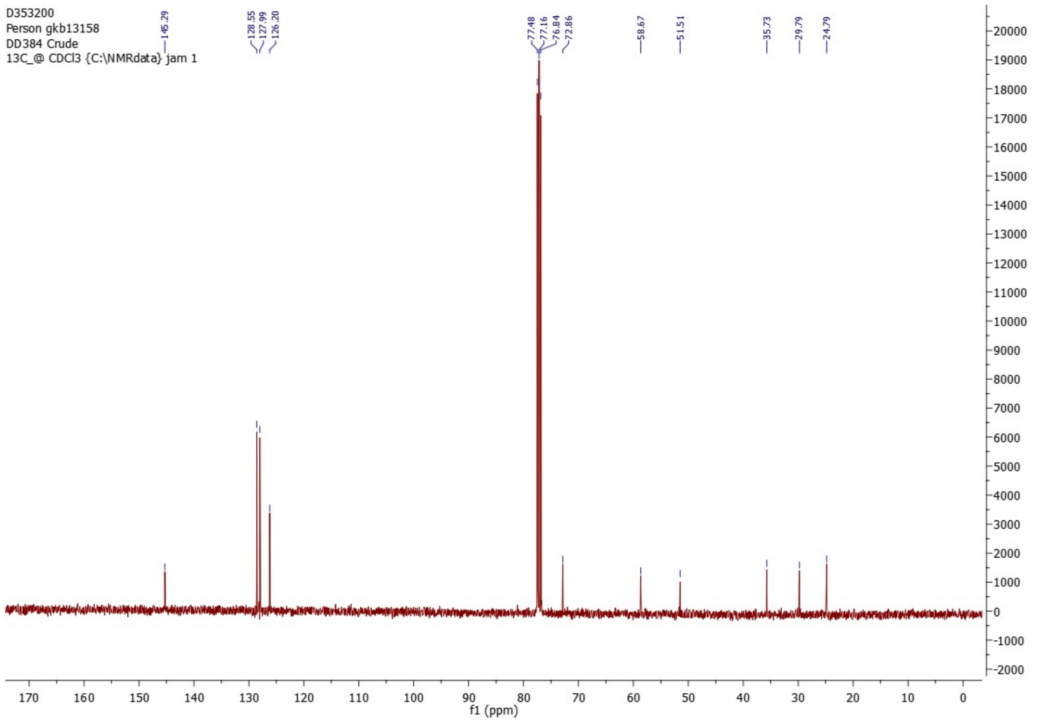

4-(9*H*-Xanthen-9-yl)butanoic acid S8

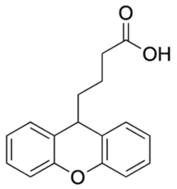

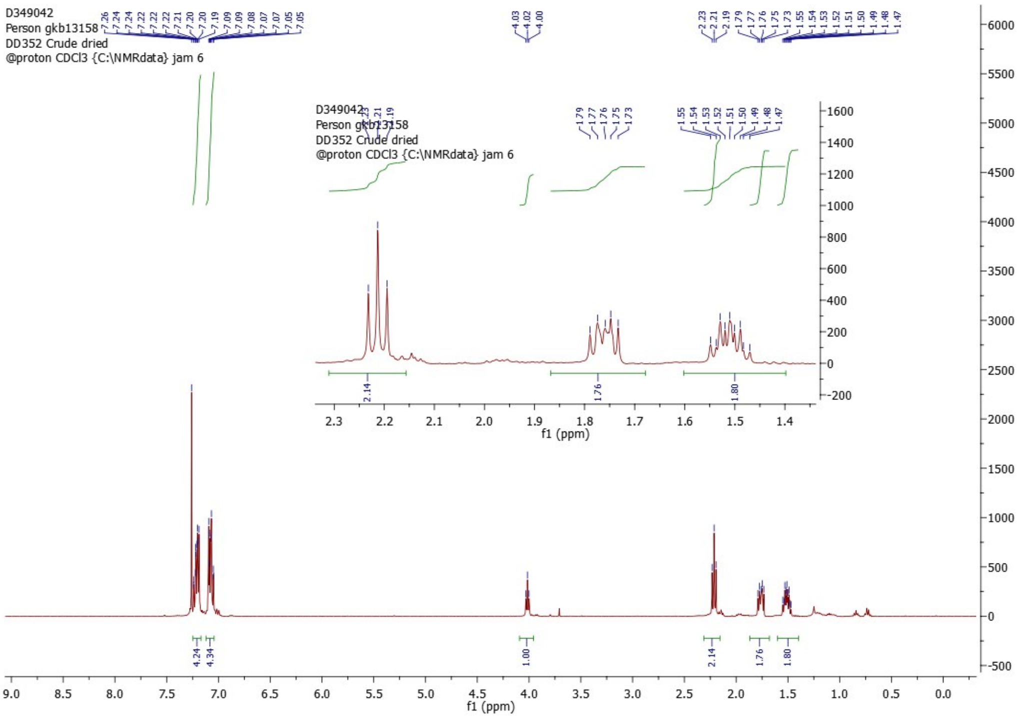

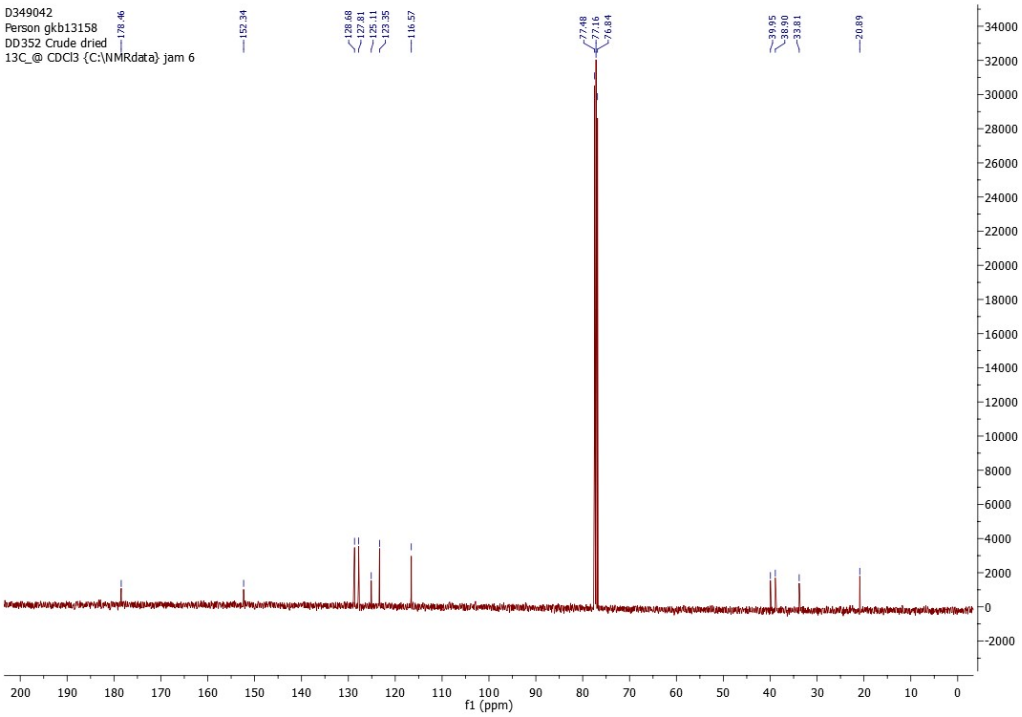

4-(9*H*-Xanthen-9-yl)butan-1-ol 30

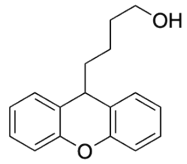

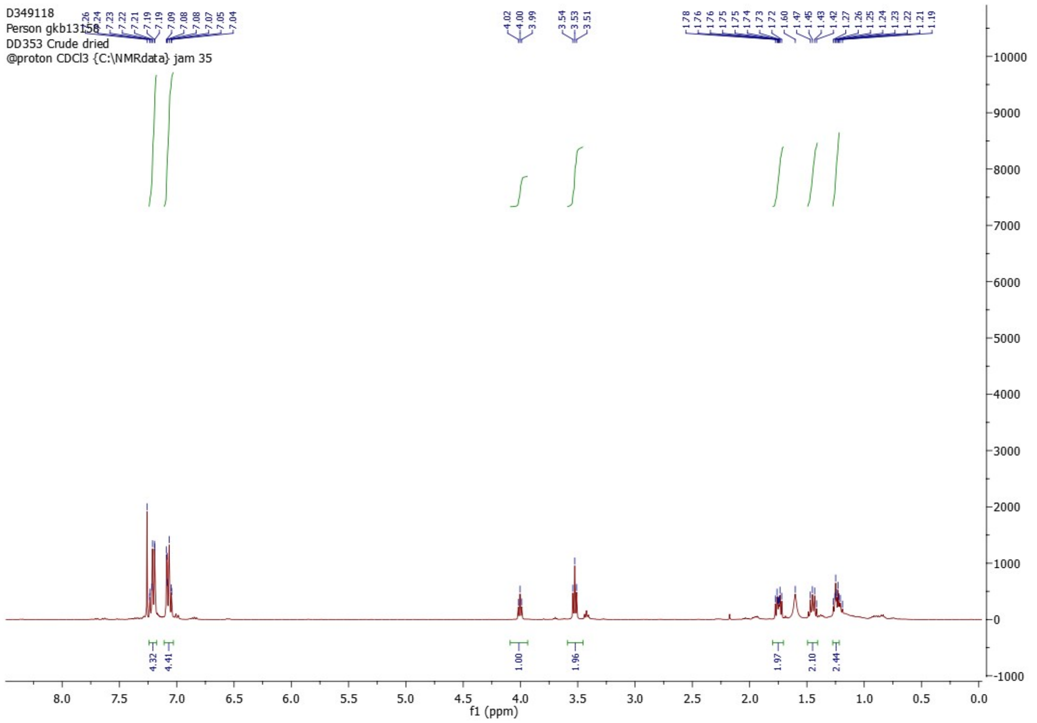

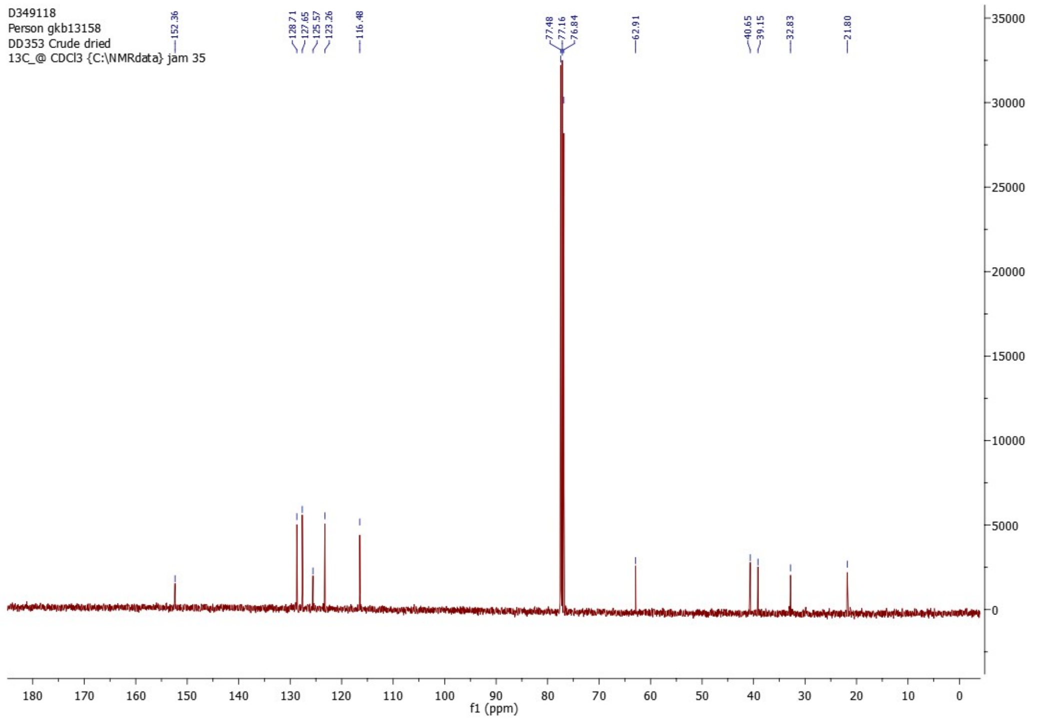

4-(10-Methyl-9,10-dihydroacridin-9-yl)butanoic acid S9

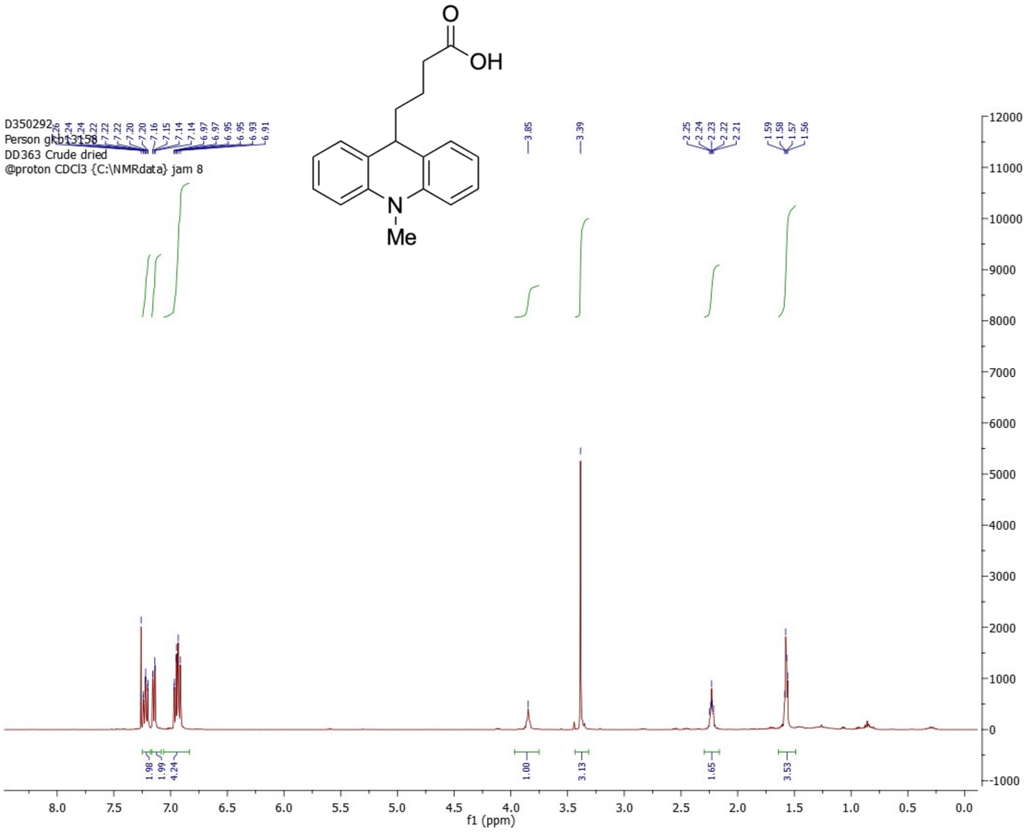

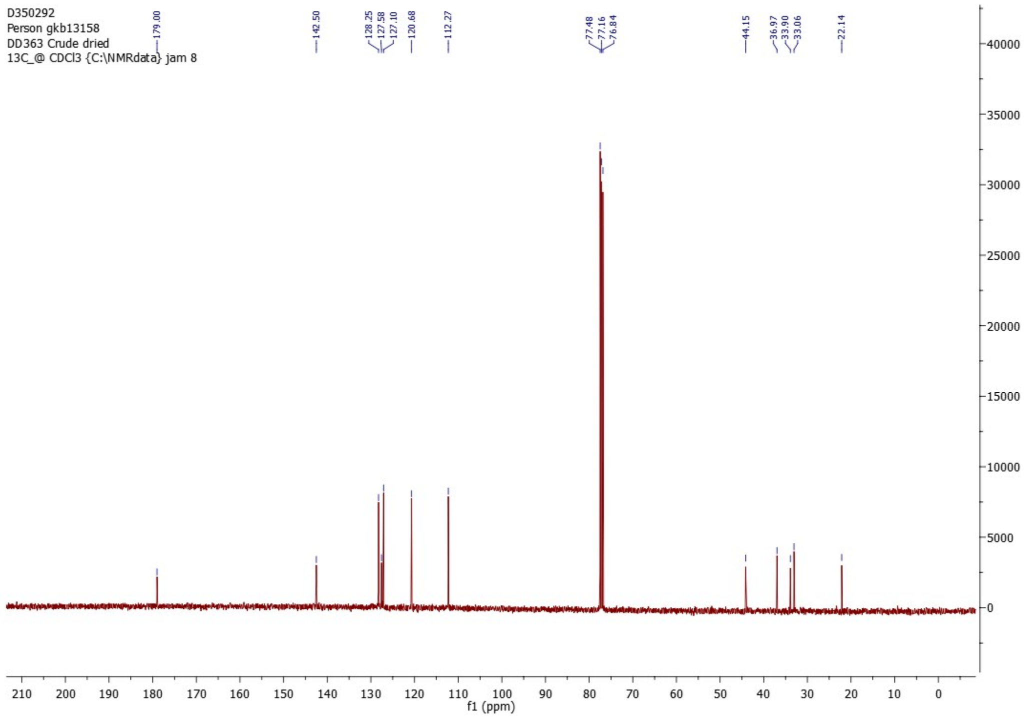

4-(10-Methyl-9,10-dihydroacridin-9-yl)butan-1-ol 29

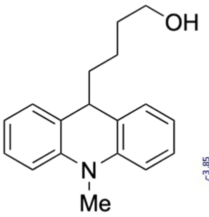

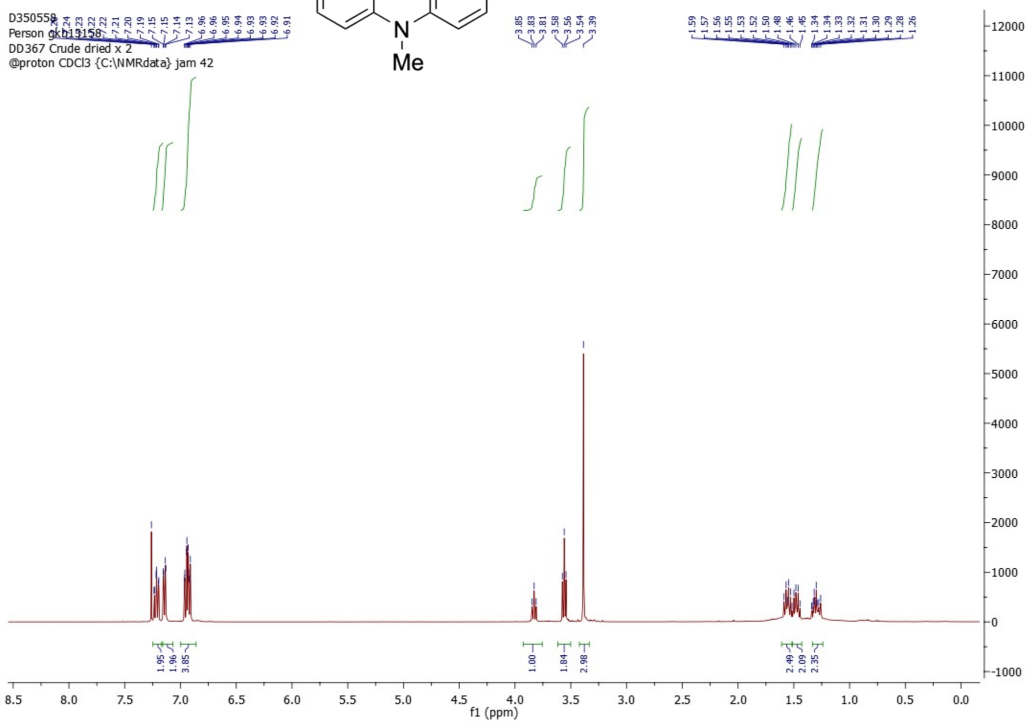

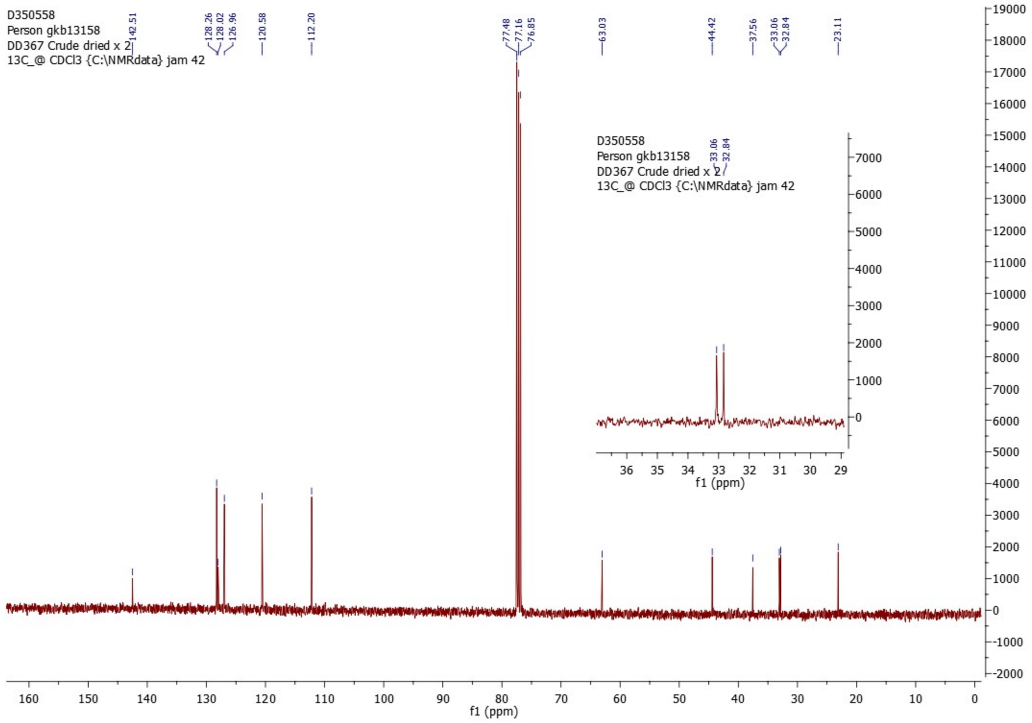

3,3-Diphenylpropan-1-ol 36

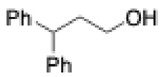

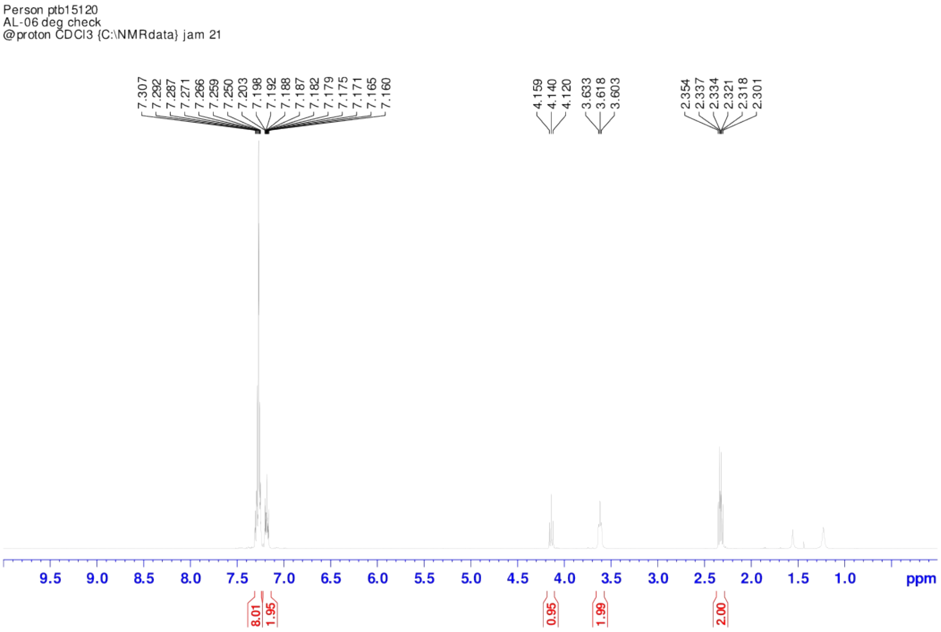

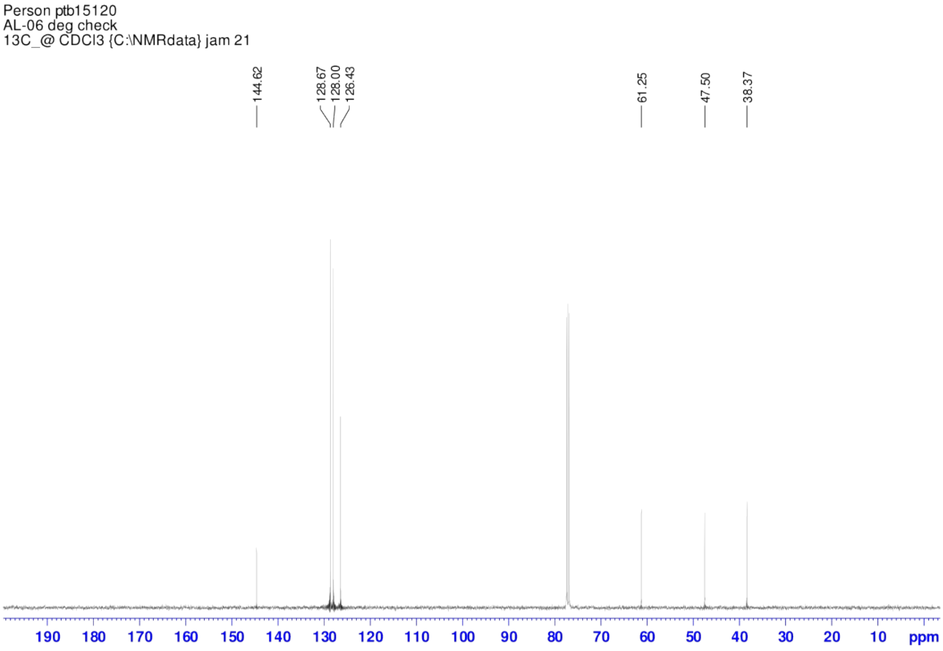

3-Phenyl-3-(pyridin-2-yl)propan-1-ol S12

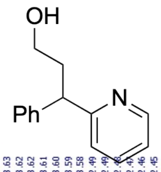

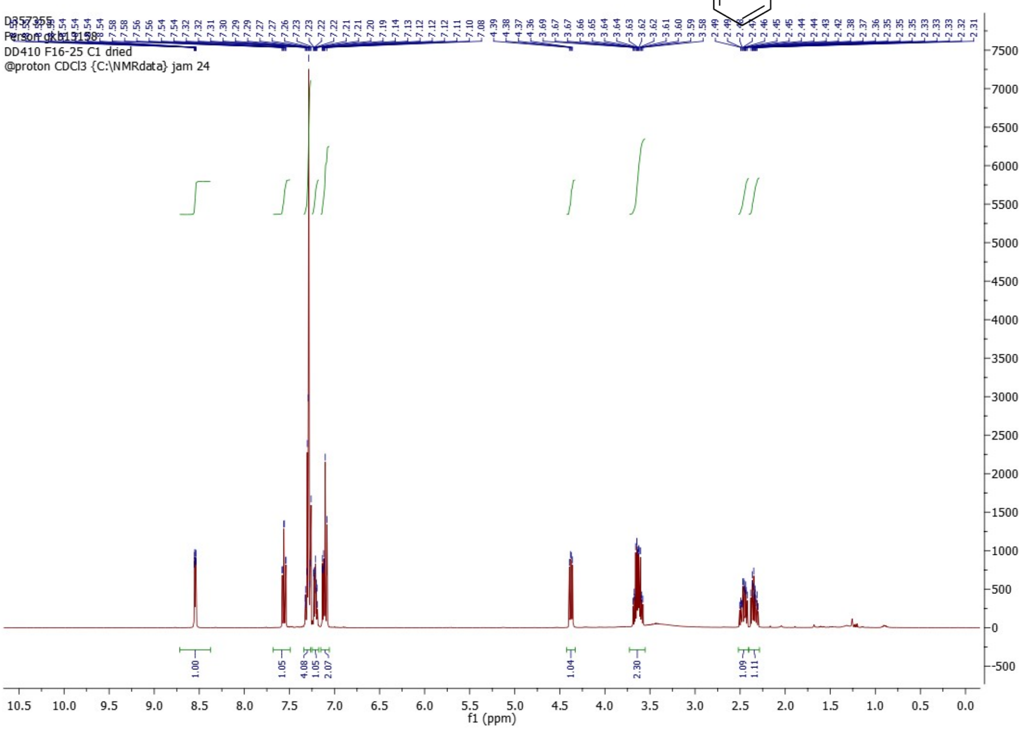

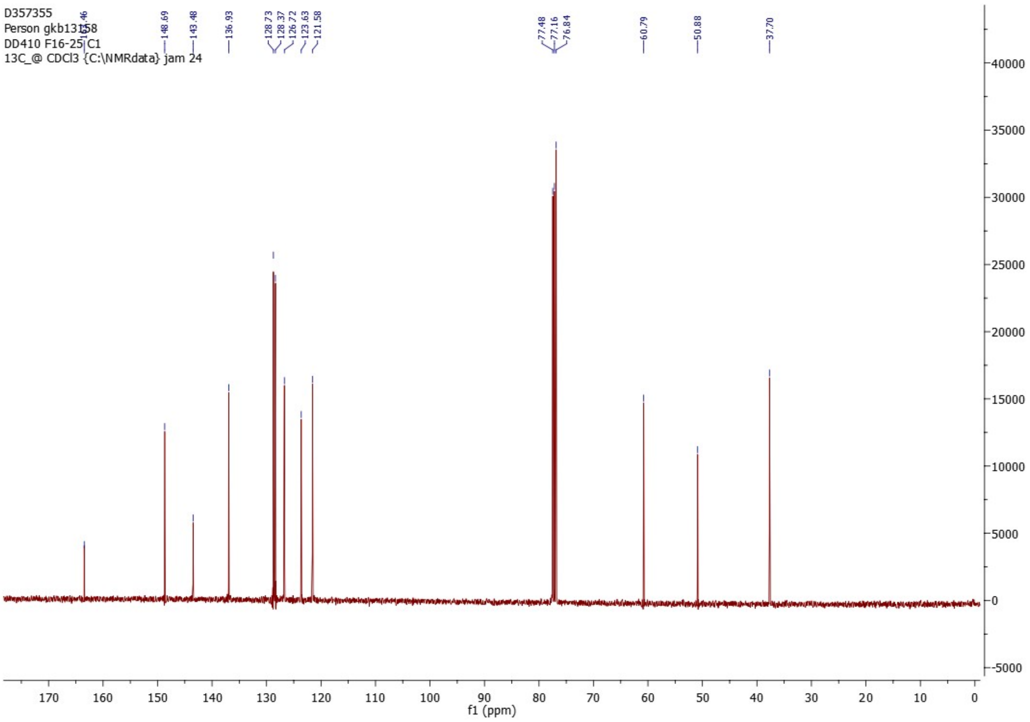

3-Phenyl-3-(pyridin-4-yl)propan-1-ol S14

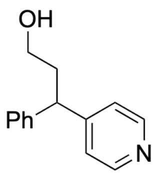

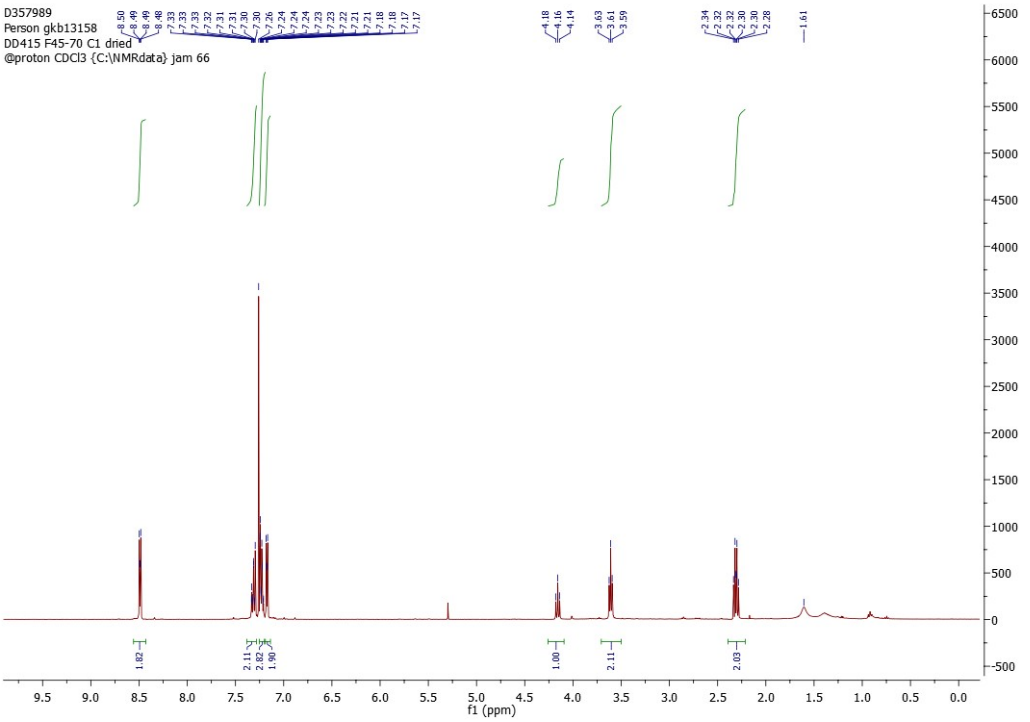

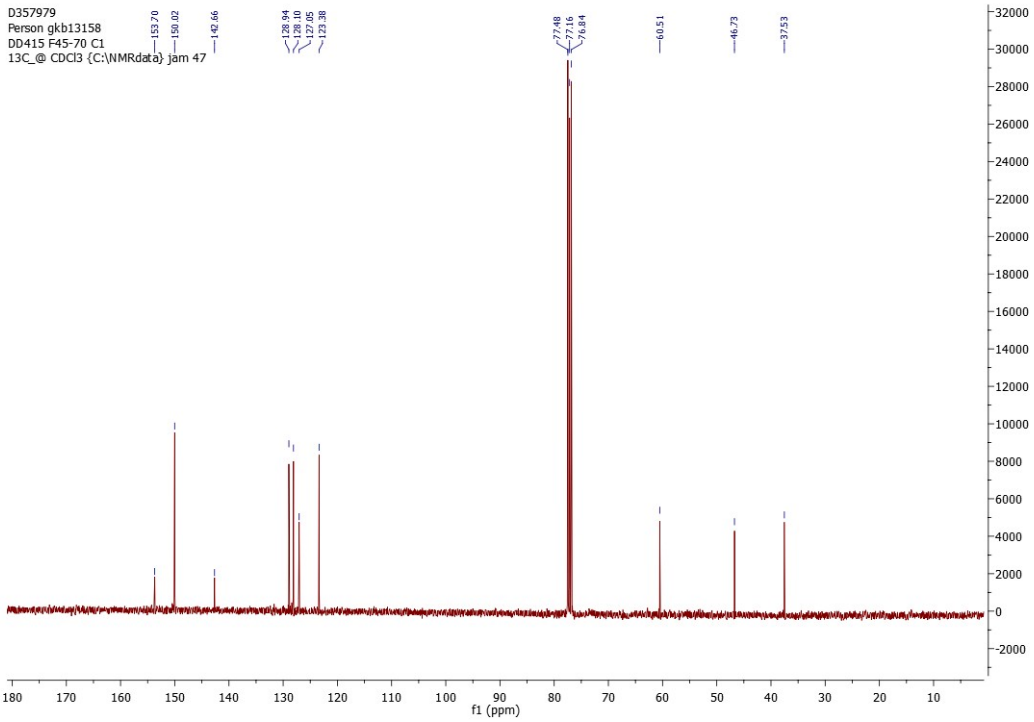

2-(1-Phenyl-3-((triethylsilyl)oxy)propyl)pyridine S15

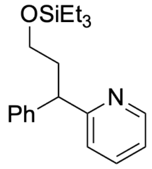

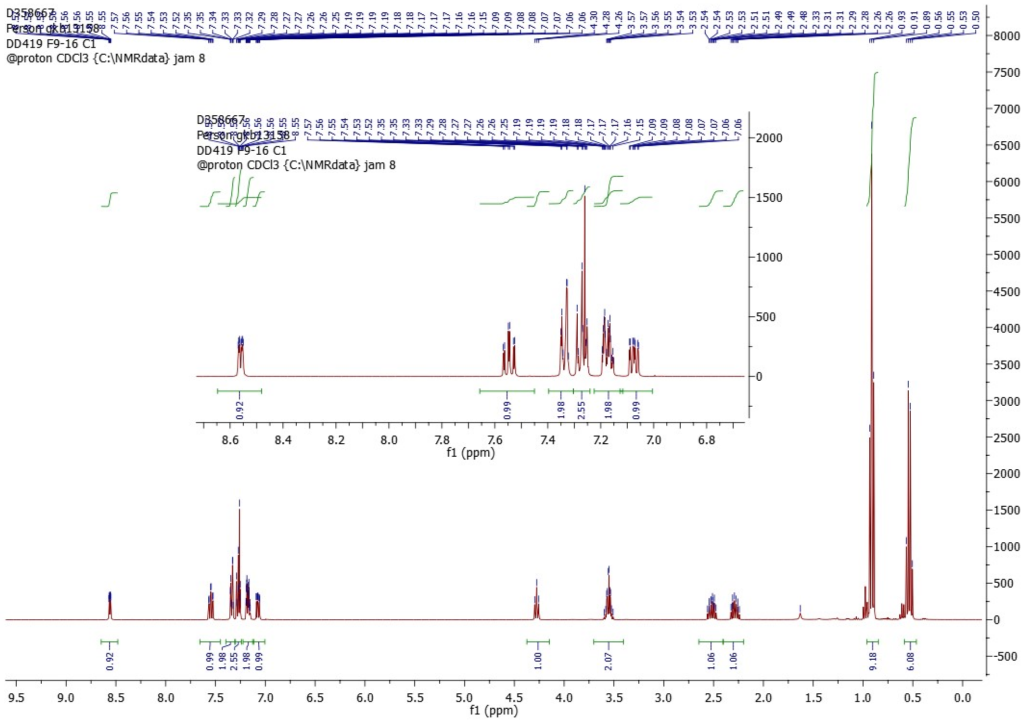

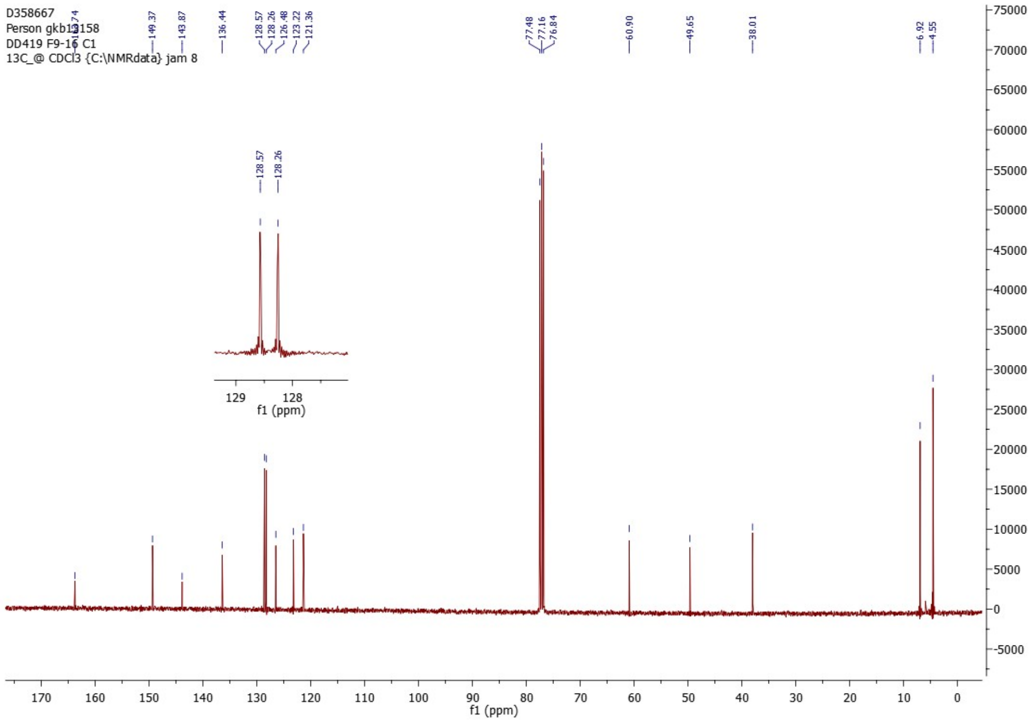

2-(9*H*-Xanthen-9-yl)acetic acid S17

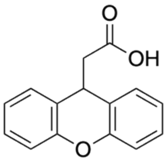

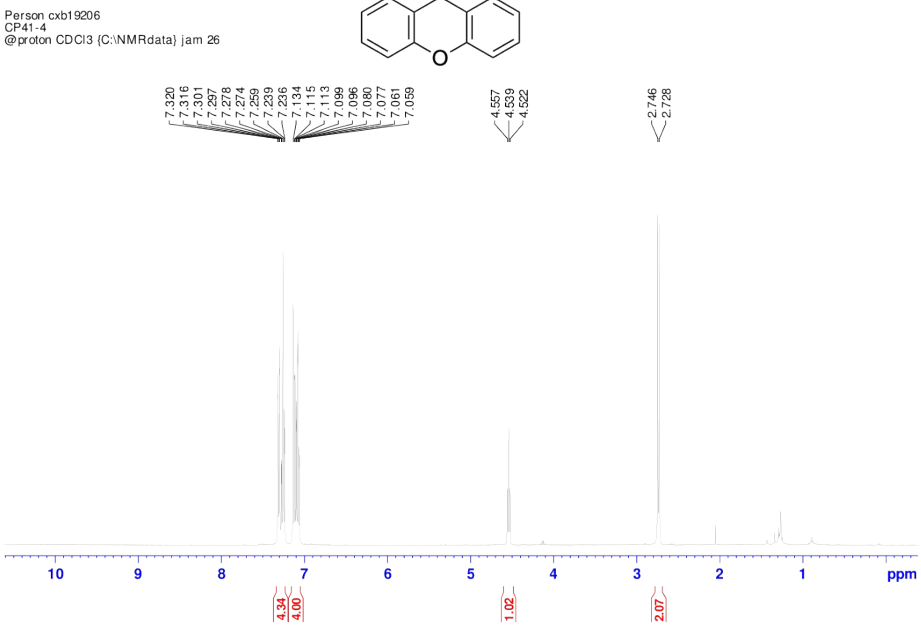

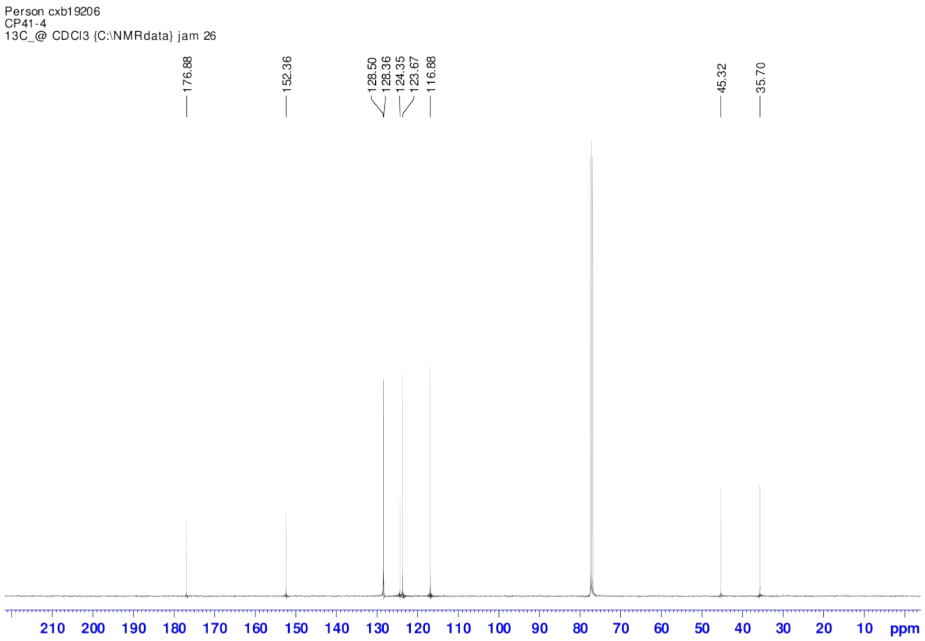

2-(9*H*-Xanthen-9-yl)ethan-1-ol S18

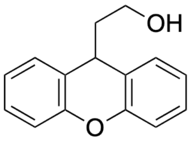

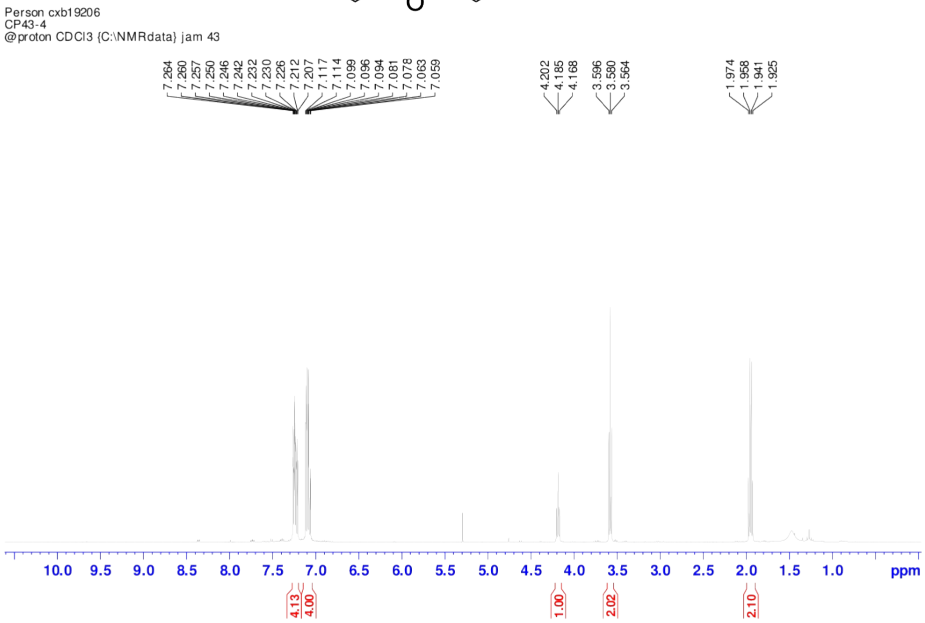

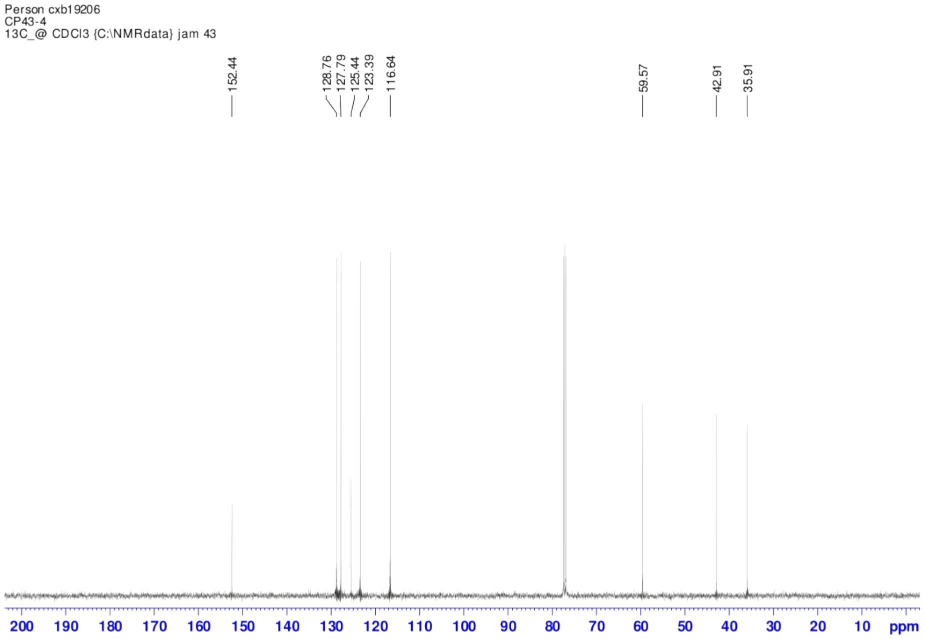

Methyl 3-(4-(*tert*-butyl)phenyl)propanoate S20

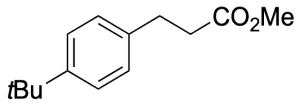

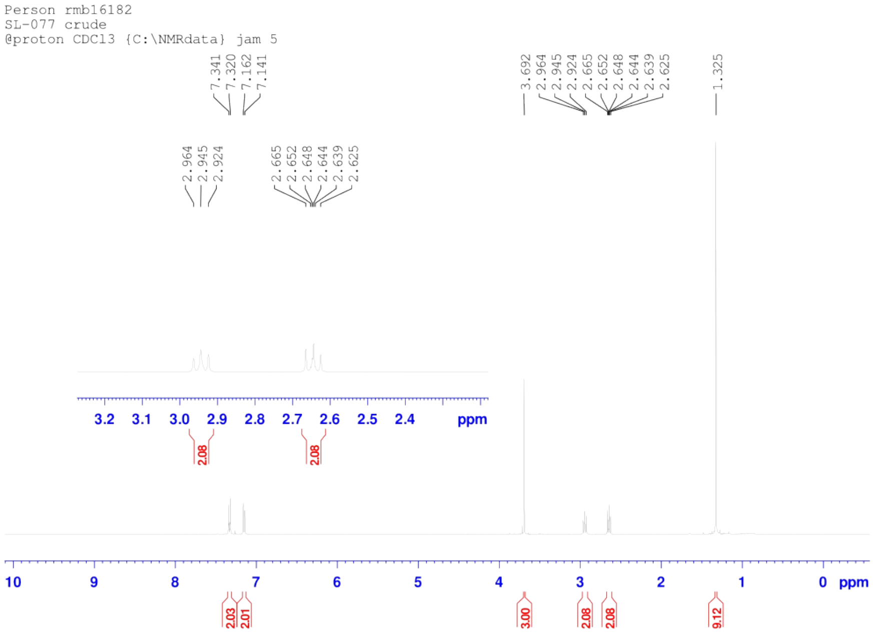

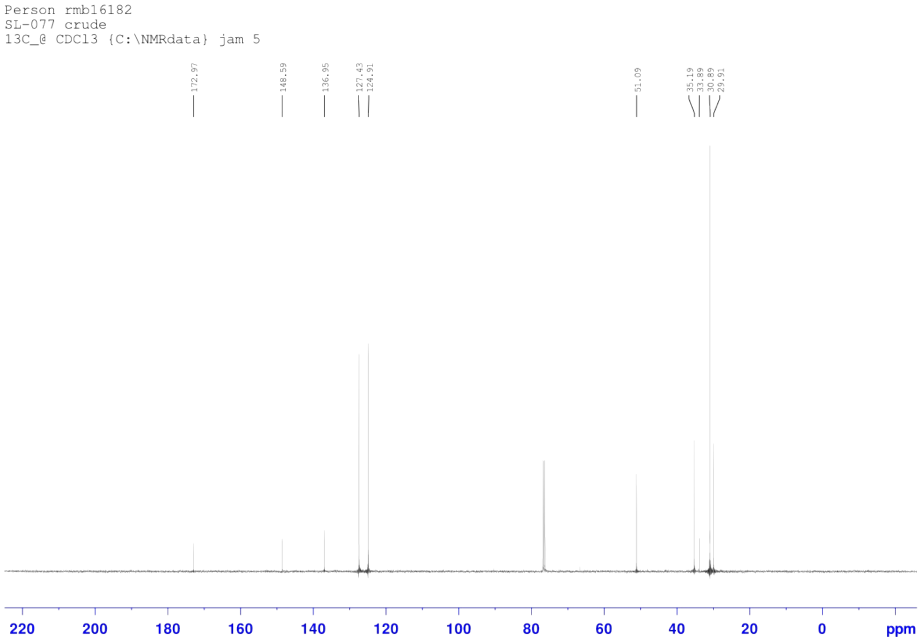

3-(4-(*Tert*-butyl)phenyl)propan-1-ol S21

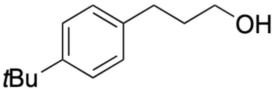

Person rmb16182
SL-089 crude
@proton16 CDCl3 {C:\NMRdata} JAM 1

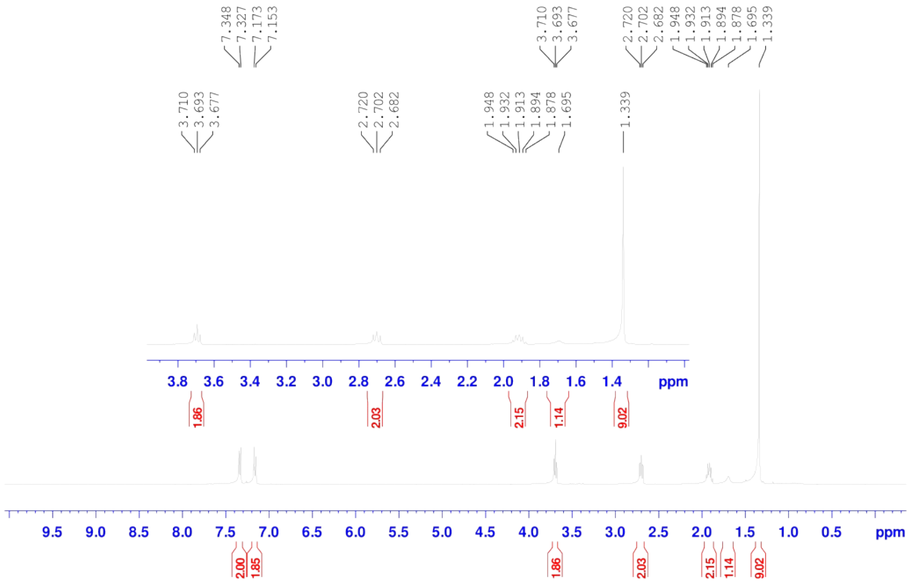

Person rmb16182
SL-089 carbon
13C_@ CDCl3 {C:\NMRdata} jam 8

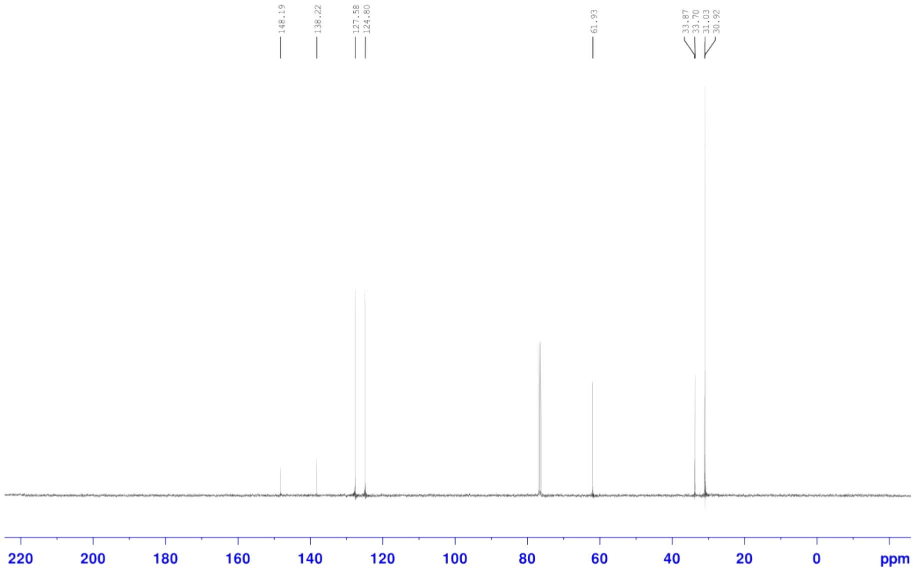

3-(Naphthalen-1-yl)propan-1-ol S23

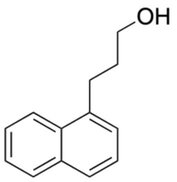

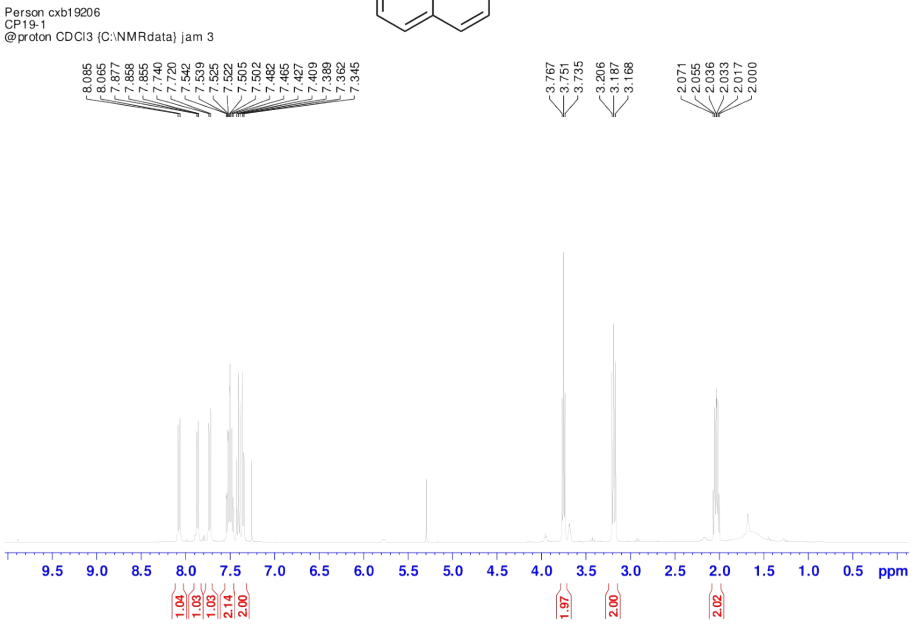

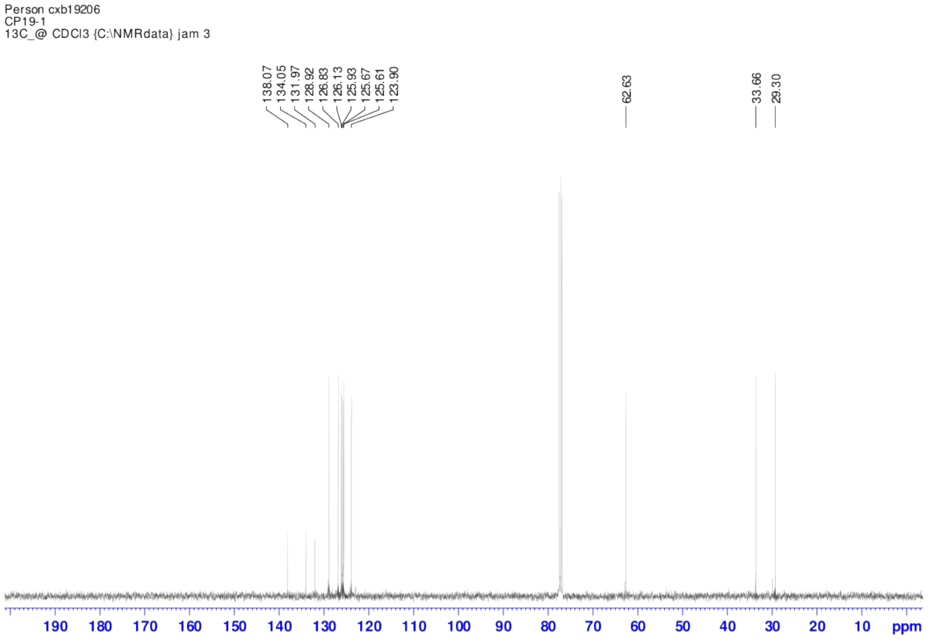

Methyl 3-(naphthalen-2-yl)propanoate S25

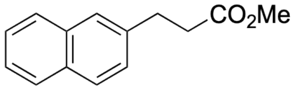

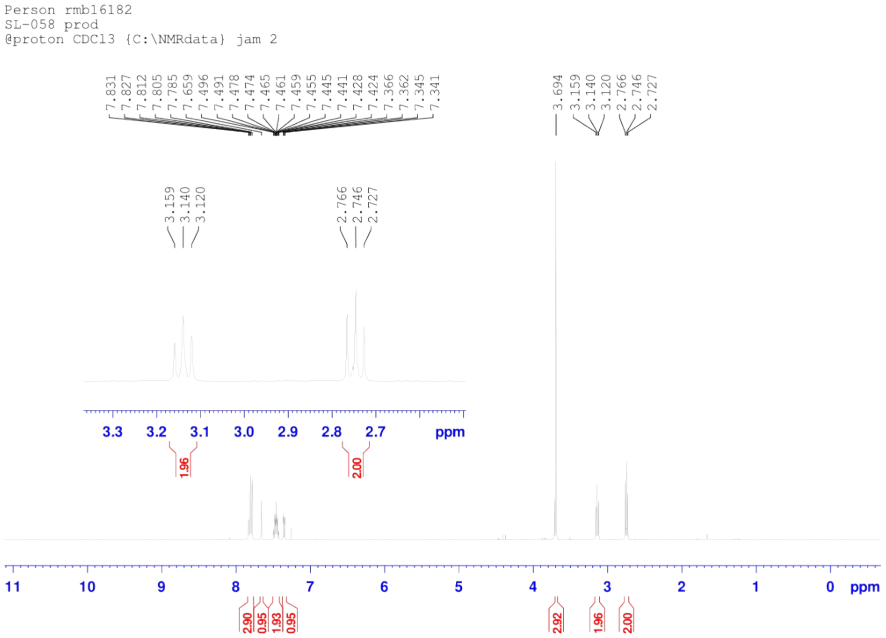

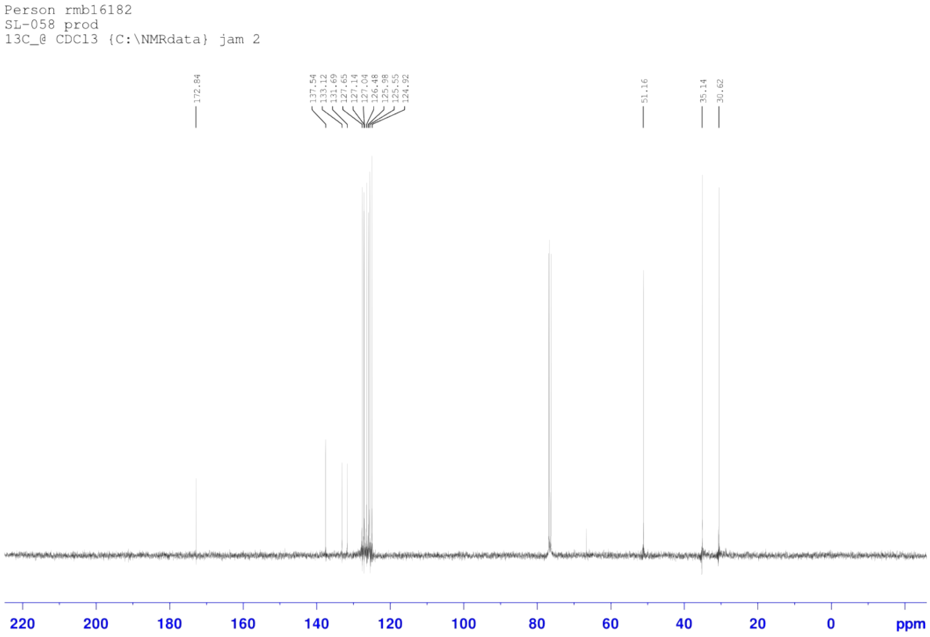

3-(Naphthalen-2-yl)propan-1-ol S26

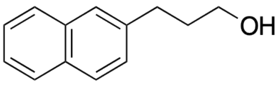

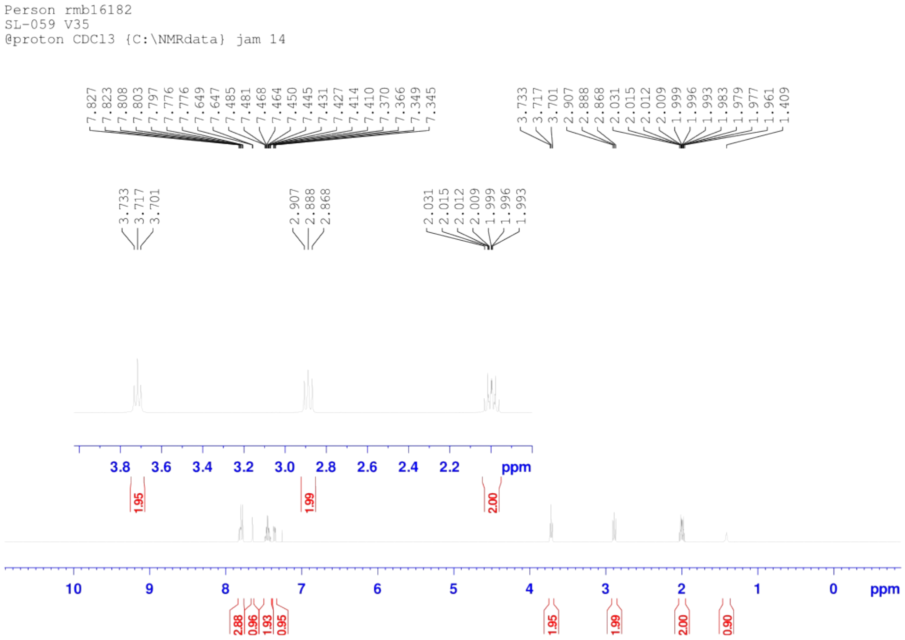

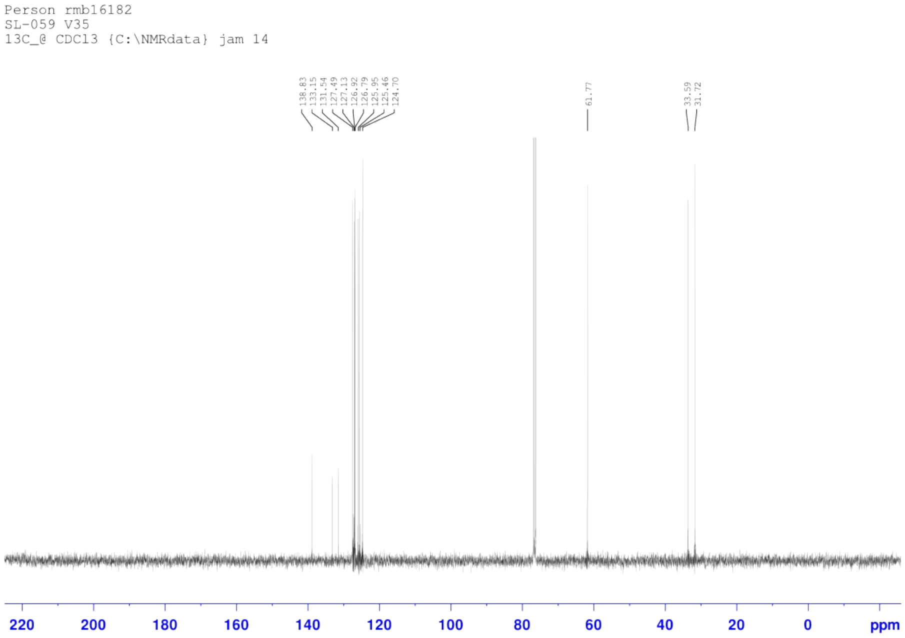

3-([1,1'-Biphenyl]-4-yl)prop-2-yn-1-ol S28

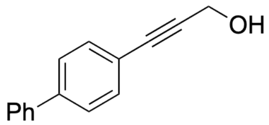

Person rmb16182
SL-051 prod
@proton CDCl3 {C:\NMRdata} jam 16

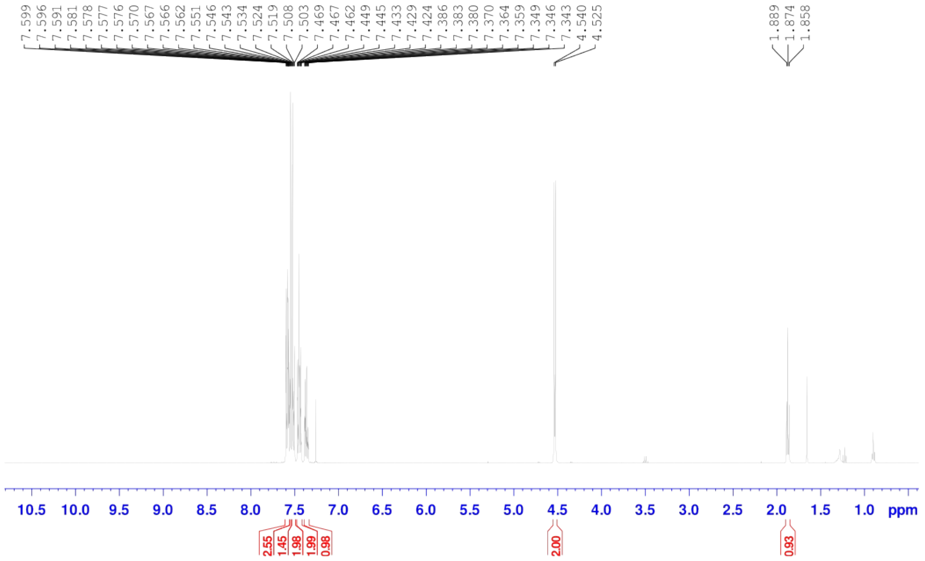

Person rmb16182
SL-051 prod
13C_@ CDCl3 {C:\NMRdata} jam 16

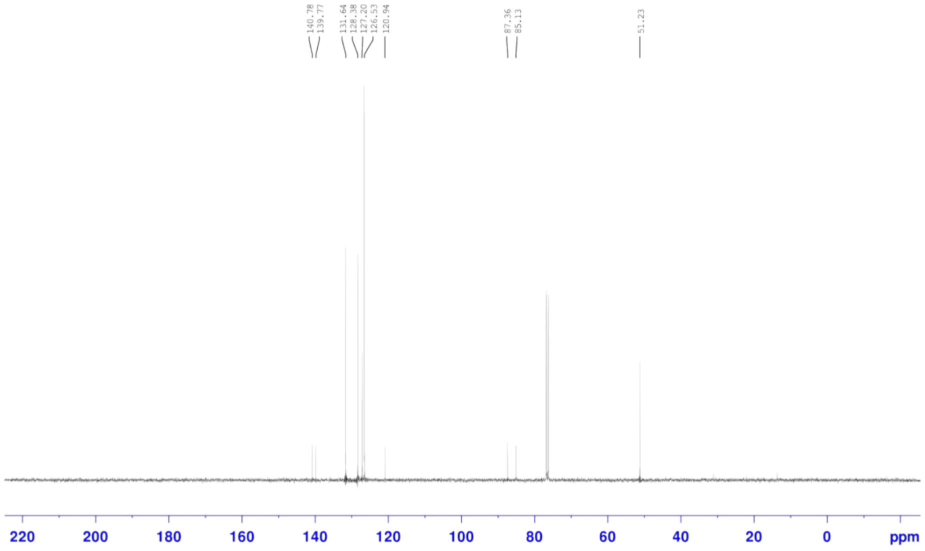

3-([1,1'-Biphenyl]-4-yl)propan-1-ol S29

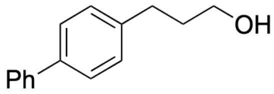

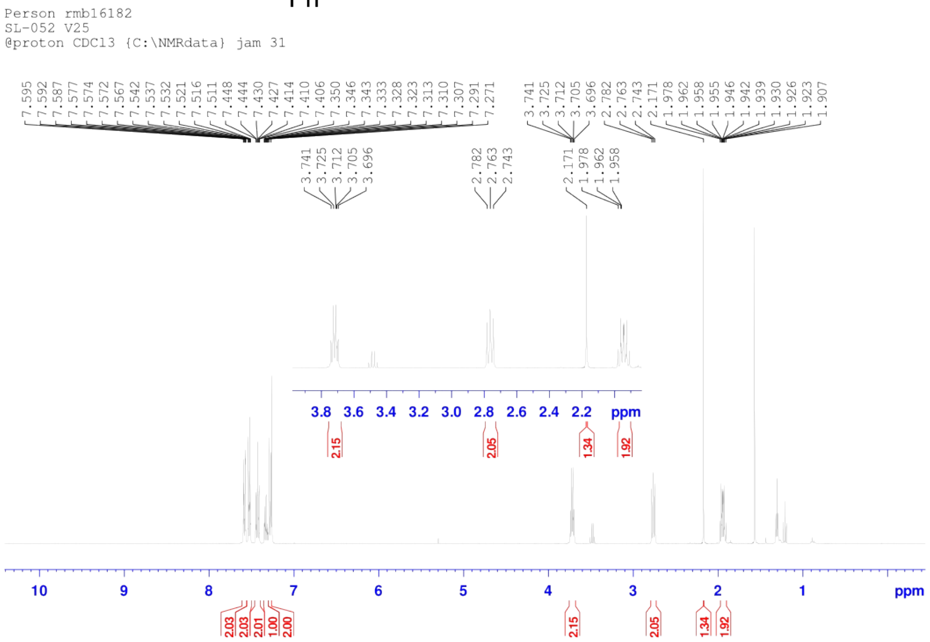

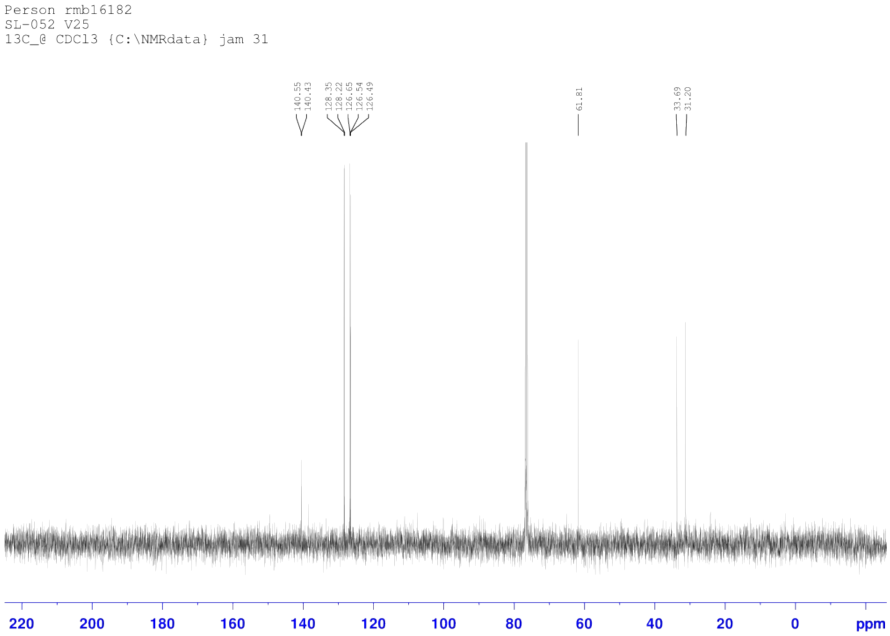

Methyl 3-([1,1'-biphenyl]-3-yl)propanoate S31

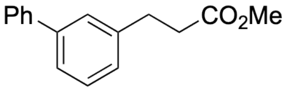

Person byb18186
KM-33-crude
@proton CDCl3 {C:\NMRdata} jam 21

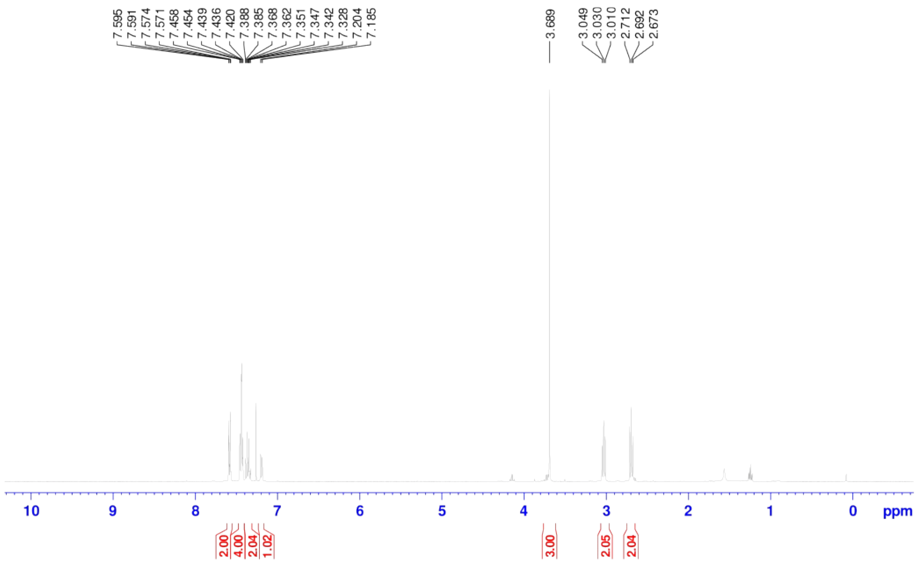

Person byb18186
KM-33-1
13C_@ CDCl3 {C:\NMRdata} jam 105

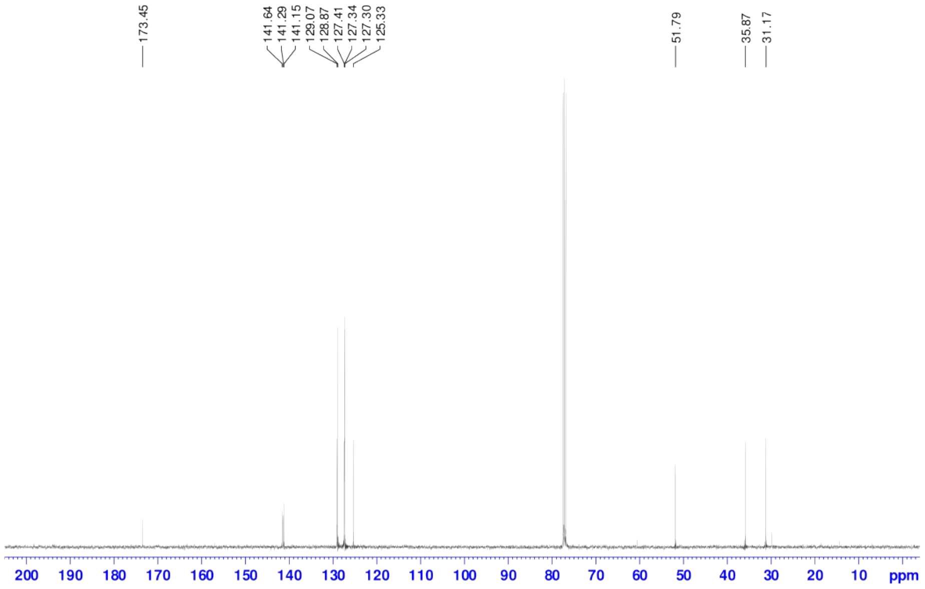

3-([1,1'-Biphenyl]-3-yl)propan-1-ol S32

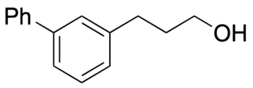

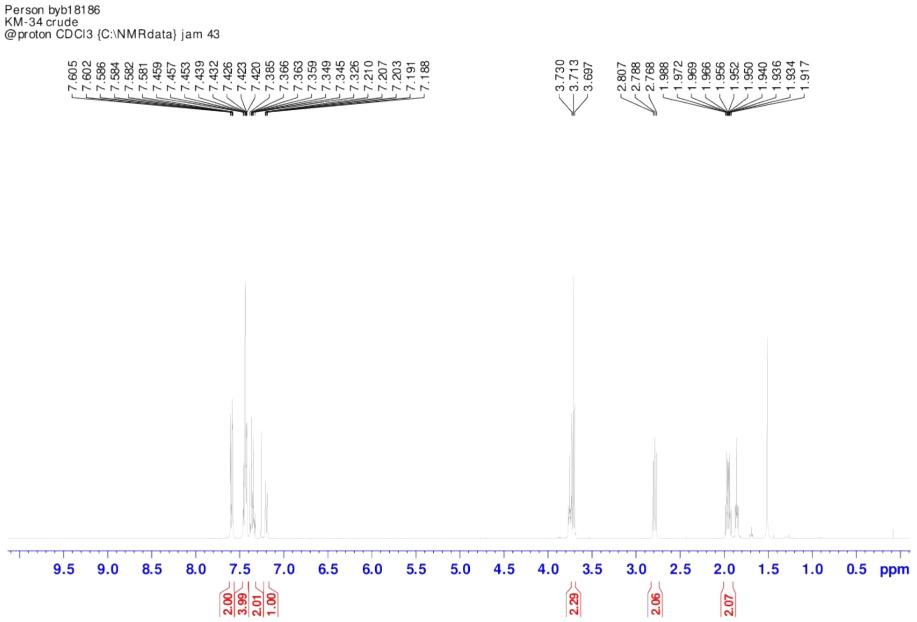

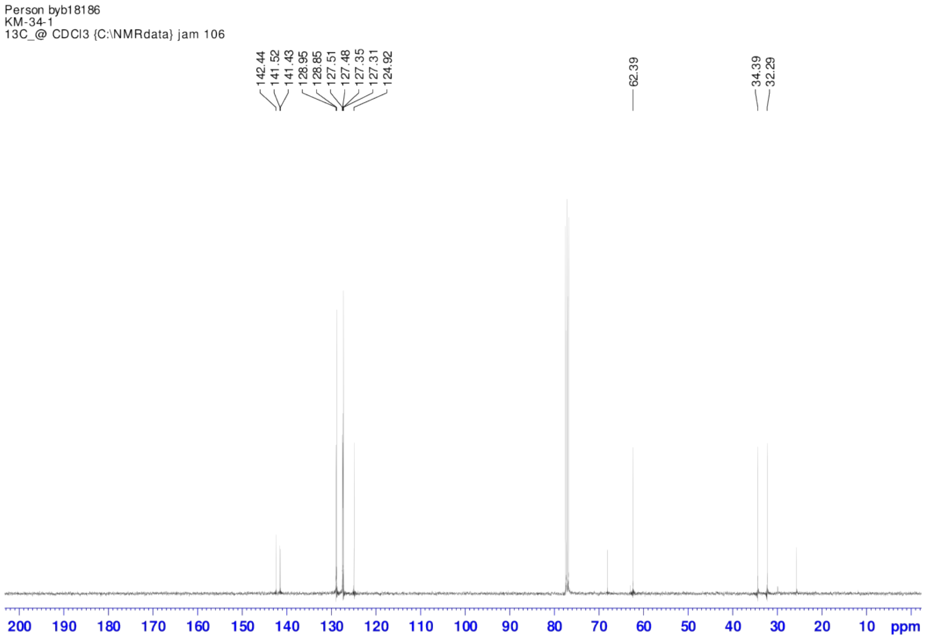

Methyl 3-([1,1'-biphenyl]-2-yl)propanoate S34

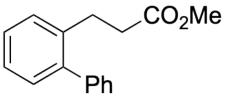

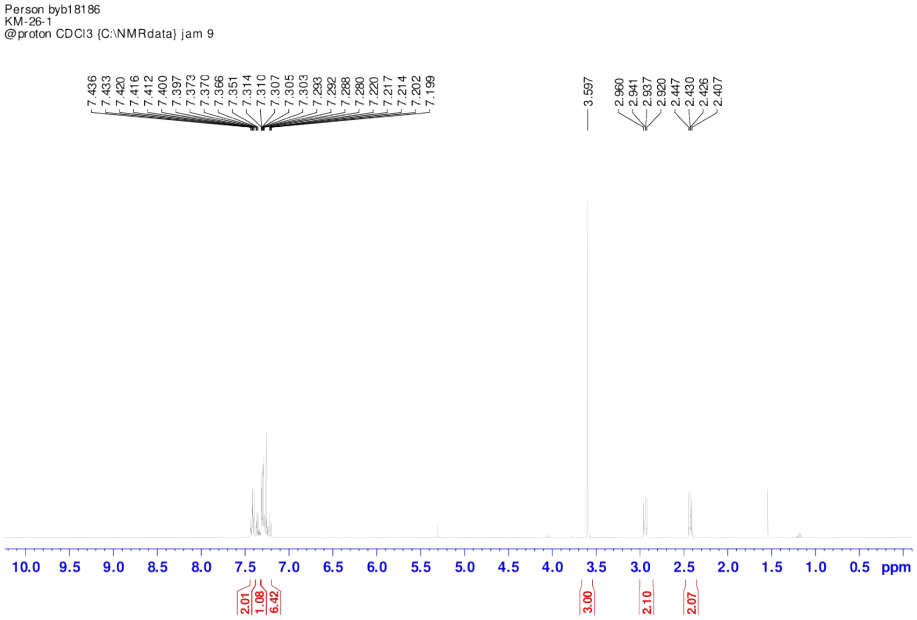

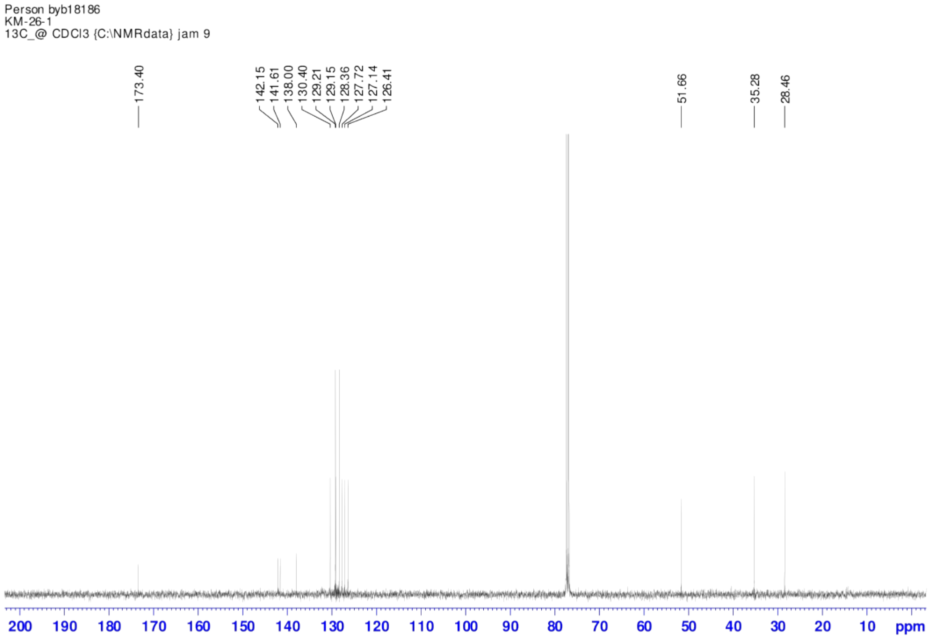

3-([1,1'-Biphenyl]-2-yl)propan-1-ol S35

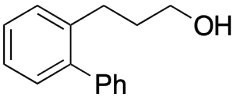

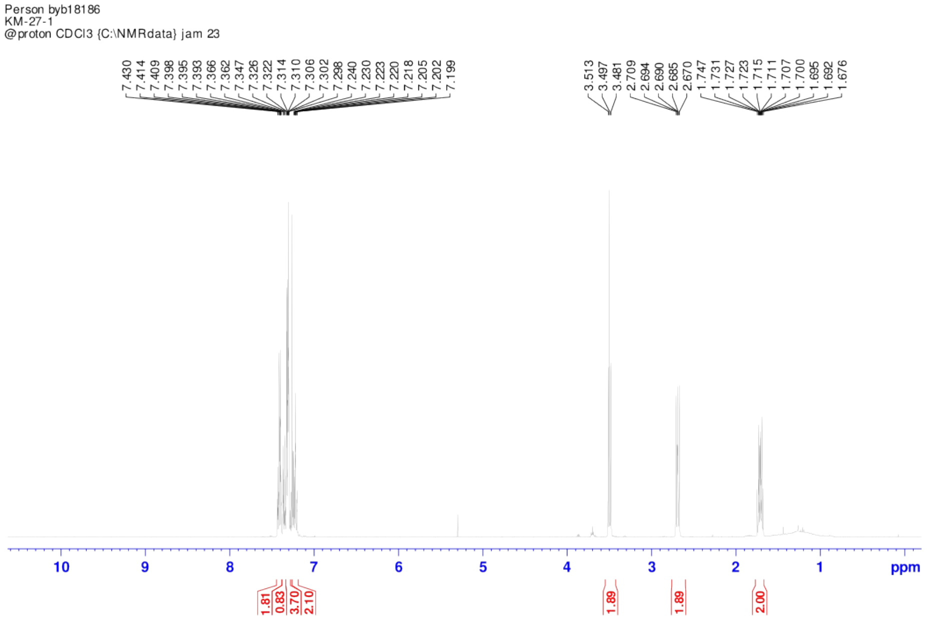

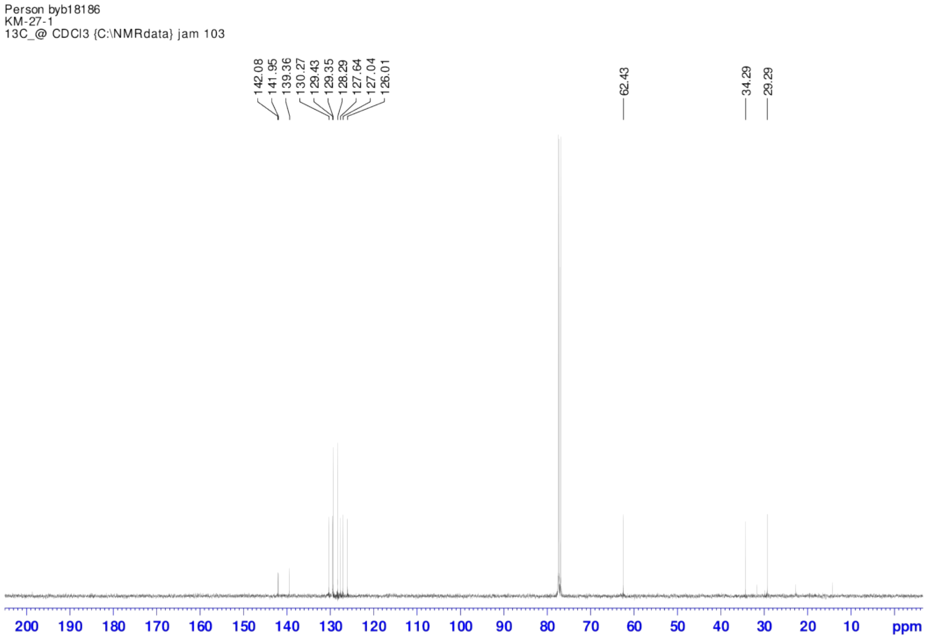

3-(Quinolin-2-yl)propan-1-ol S37

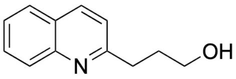

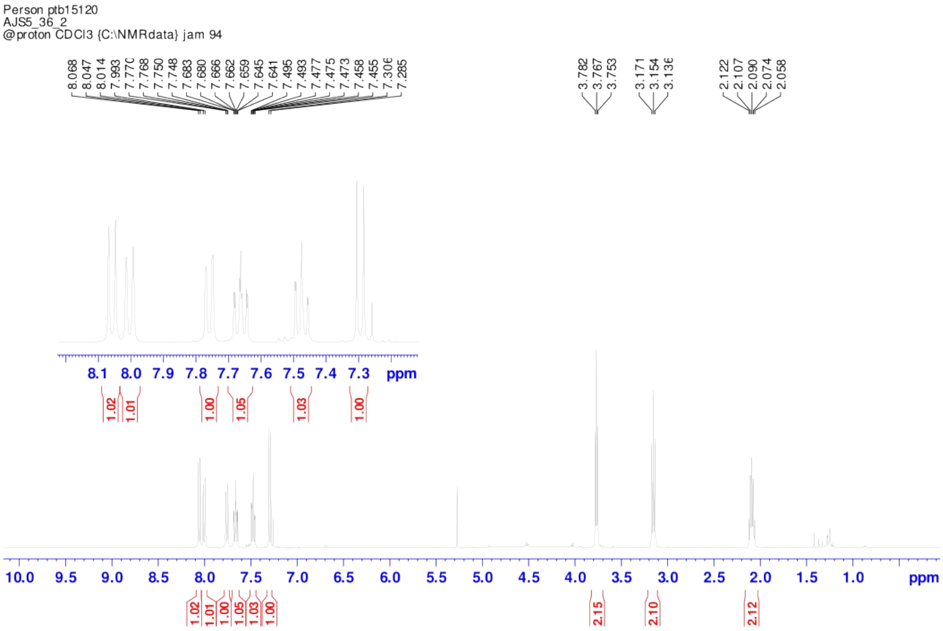

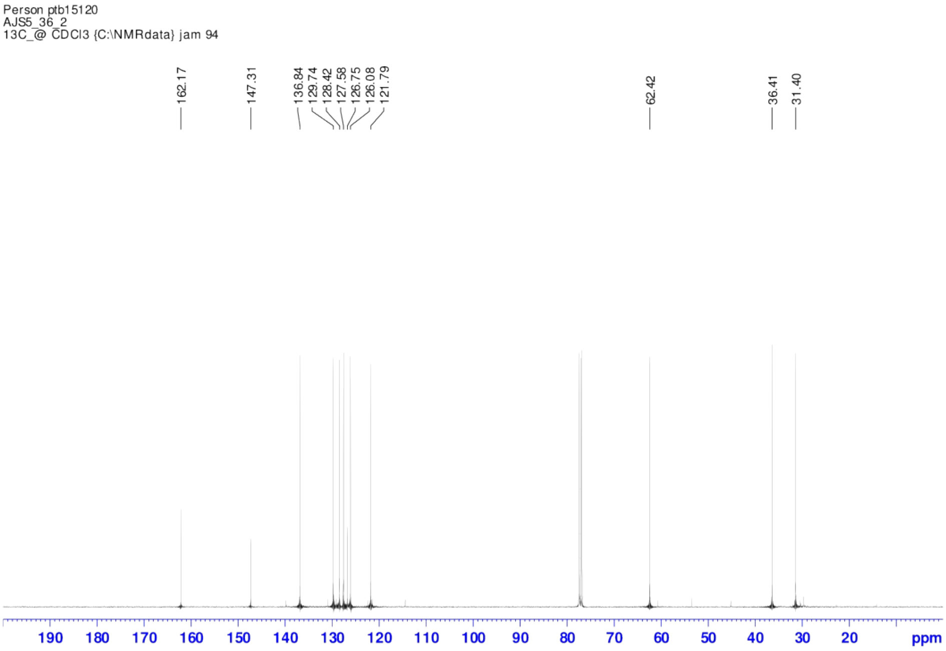

3-(Pyridin-4-yl)propan-1-ol S39

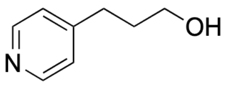

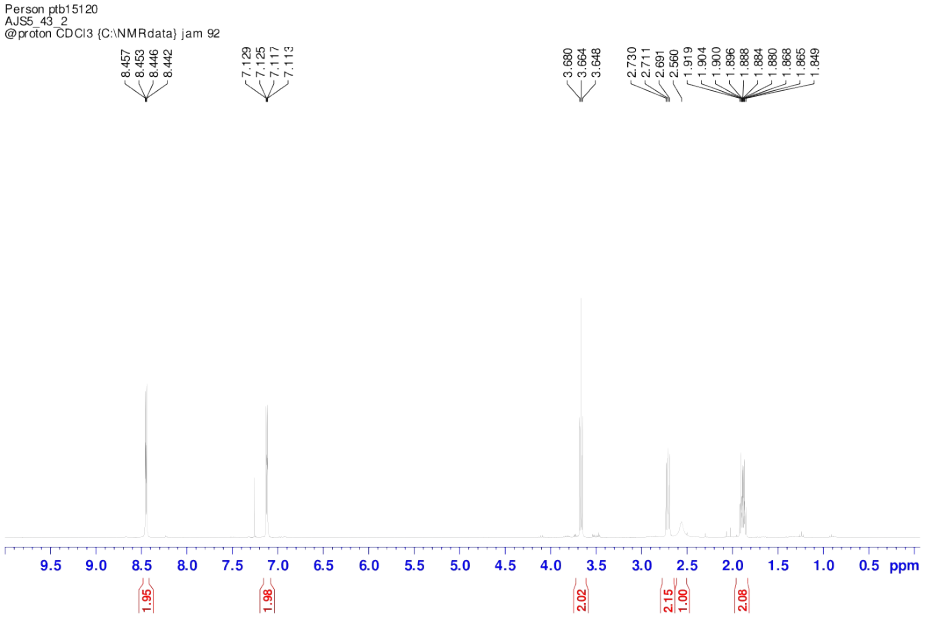

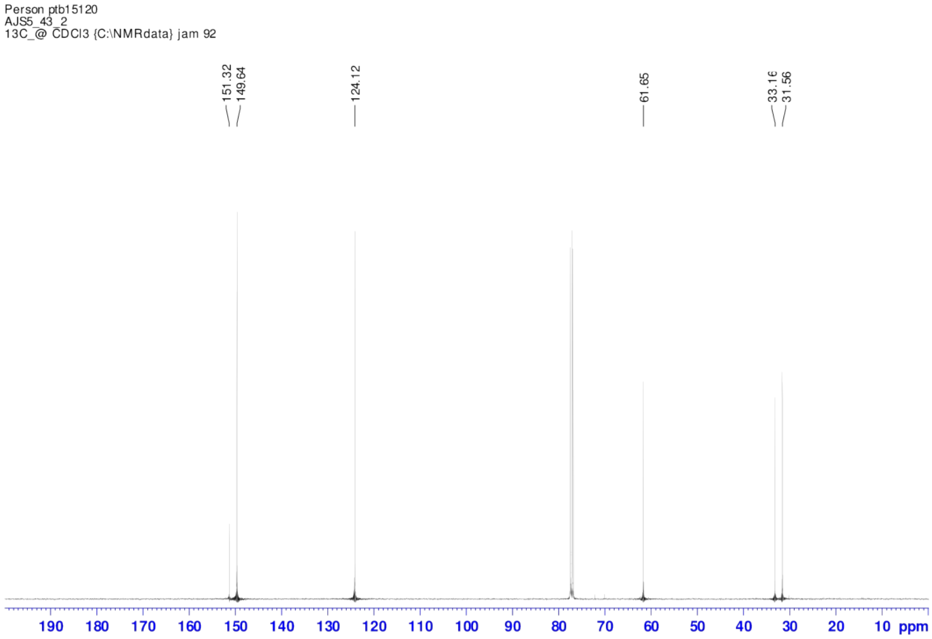

3-(4-Thiophen-2-yl)phenyl)propan-1-ol S41

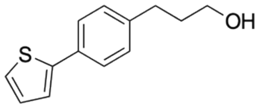

Person cxb19206
CP60-6
@proton CDCl3 {C:\NMRdata} jam 5

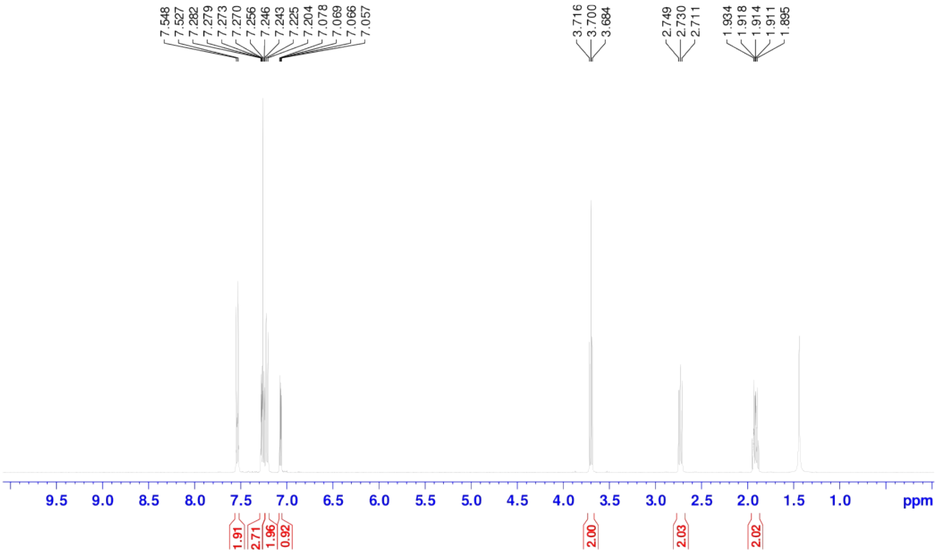

Person cxb19206
CP60-6
13C_@ CDCl3 {C:\NMRdata} jam 5

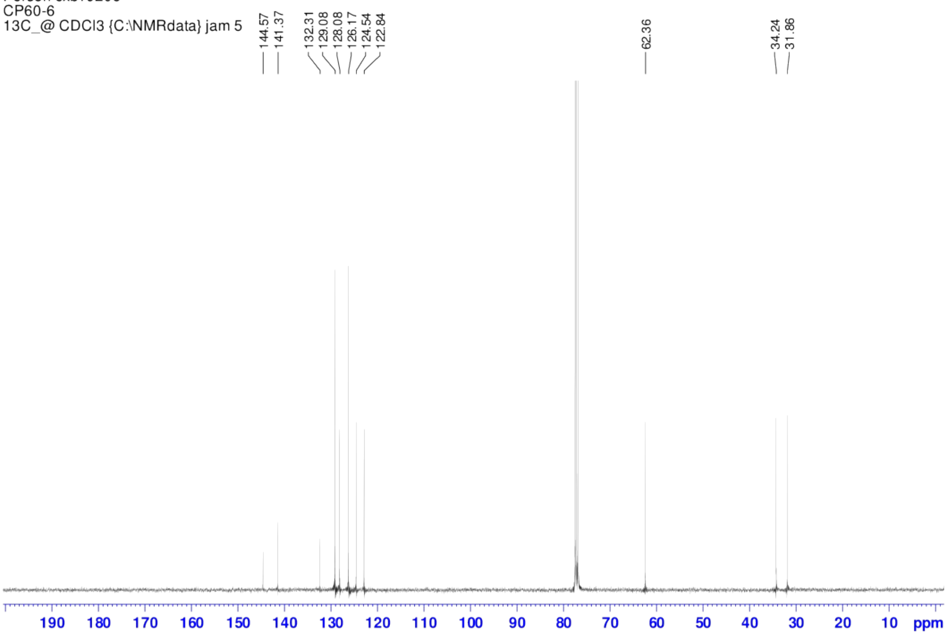

Methyl 3-(benzo[*b*]thiophen-3-yl)propanoate S43

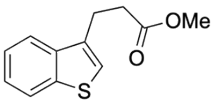

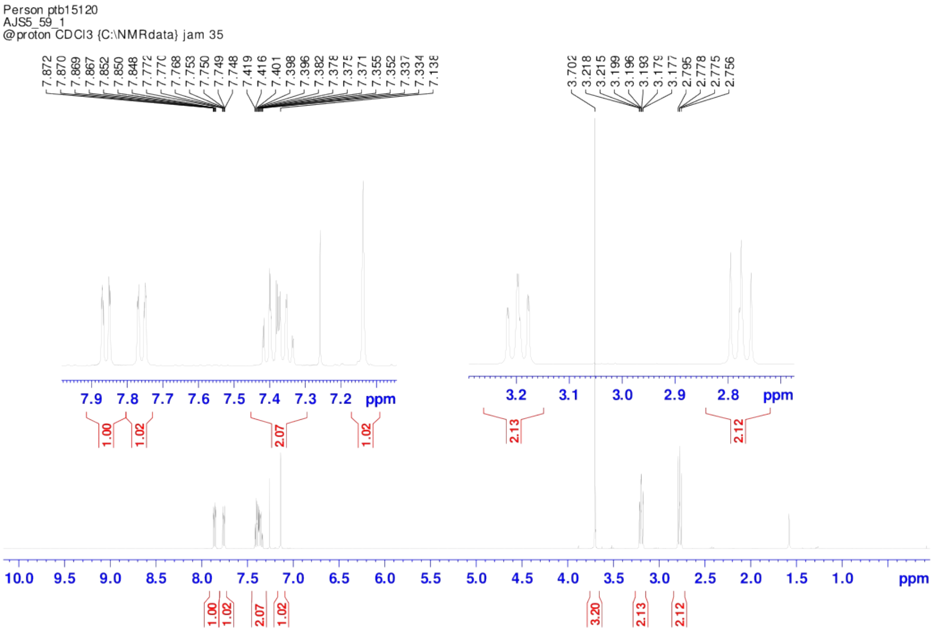

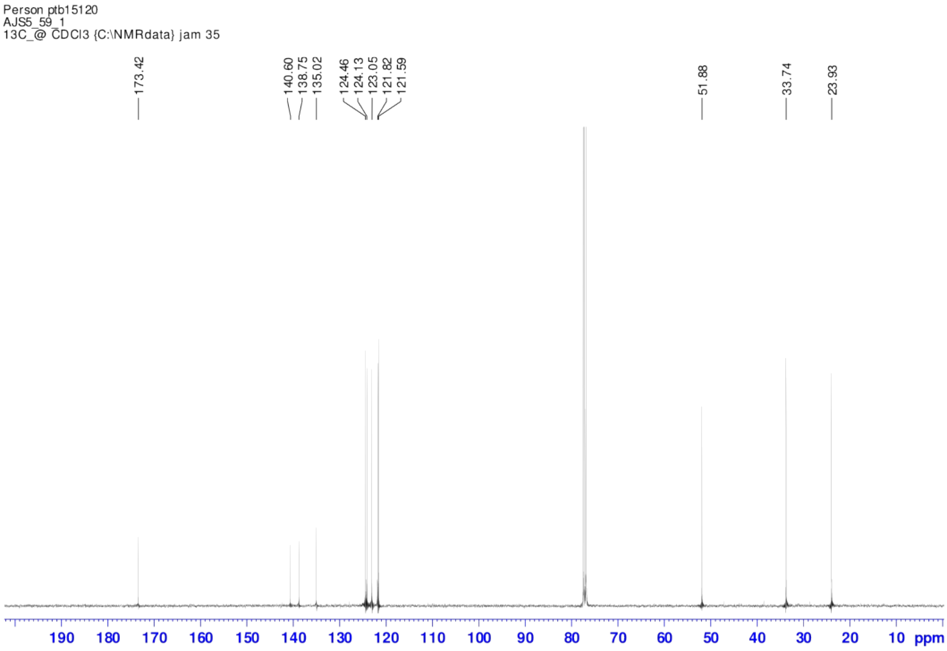

3-(Benzo[*b*]thiophen-3-yl)propan-1-ol S44

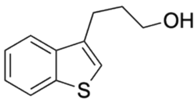

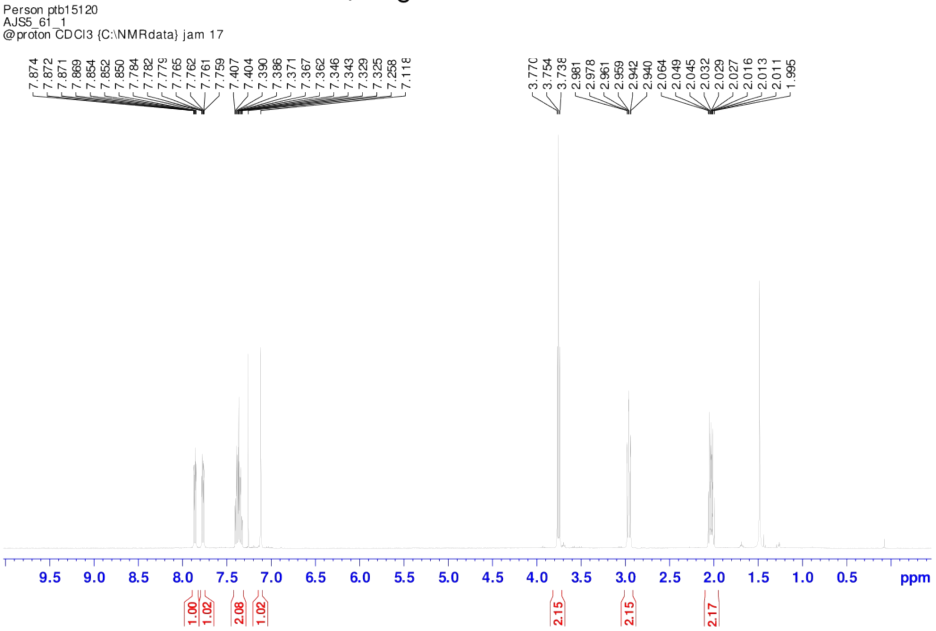

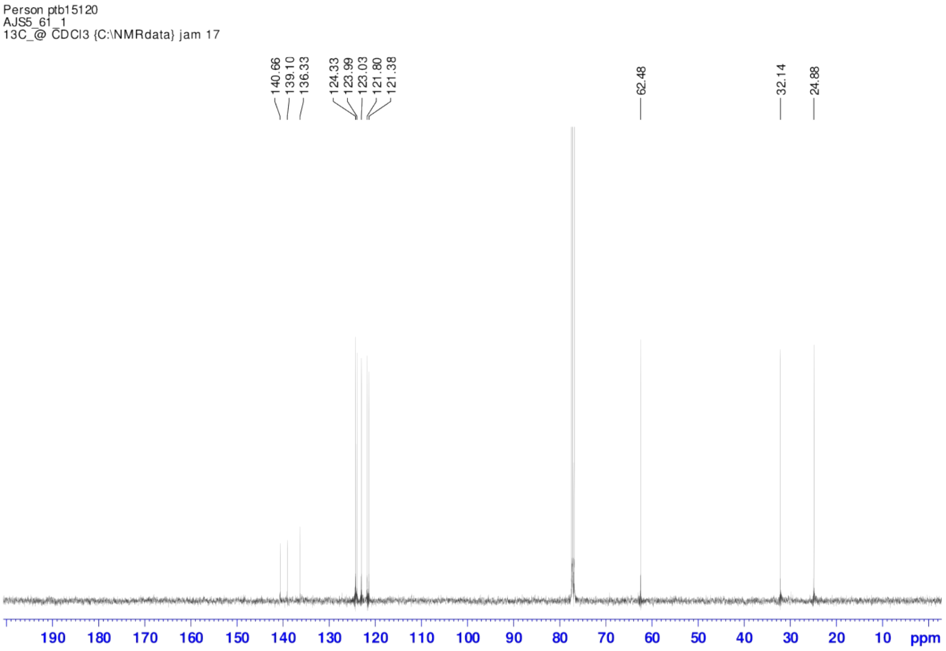

(3-Methoxypropane-1,1-diyl)dibenzene S47

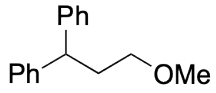

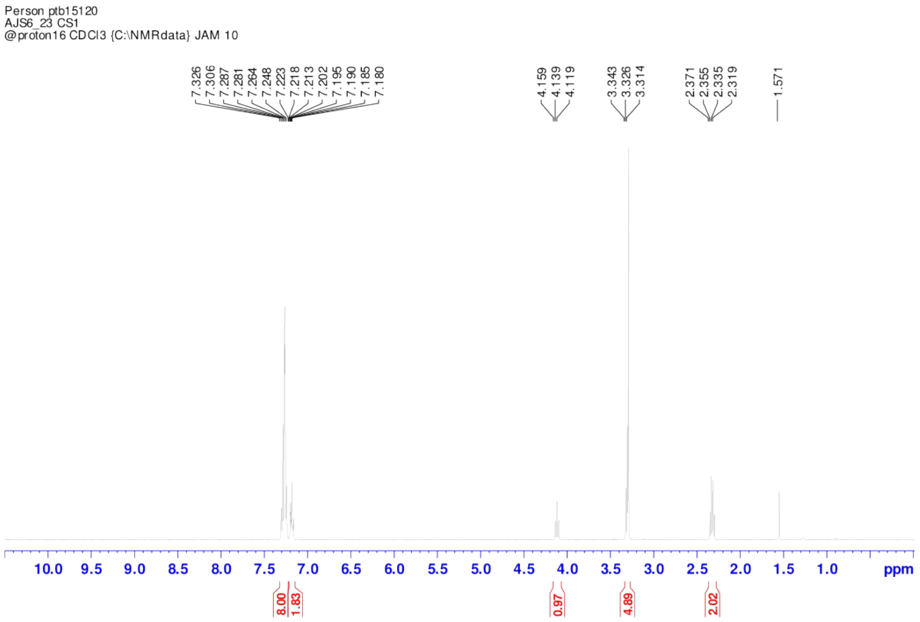

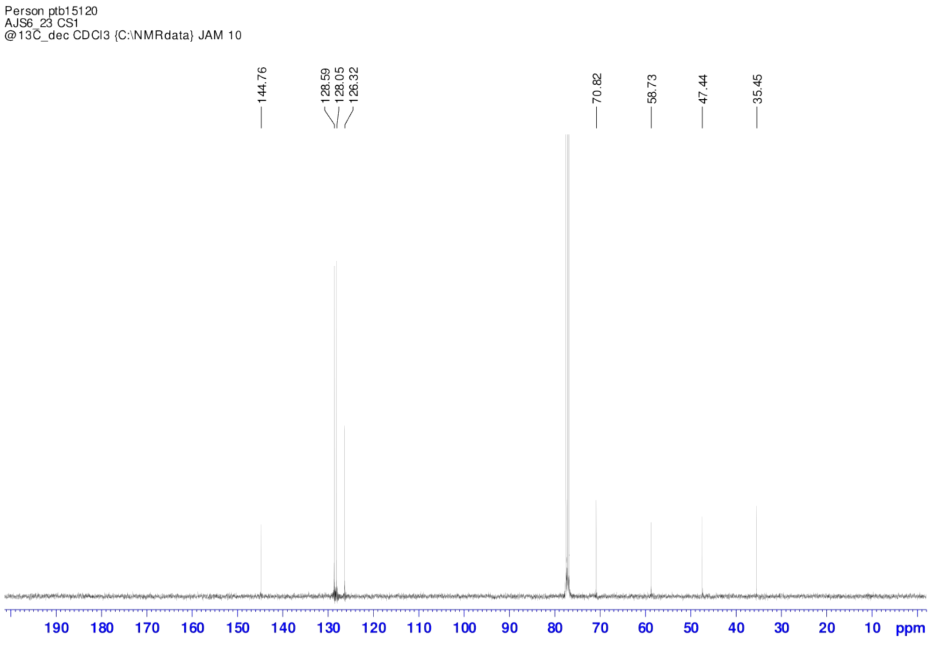

(3-Ethoxypropane-1,1-diyl)dibenzene S48

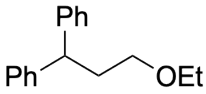

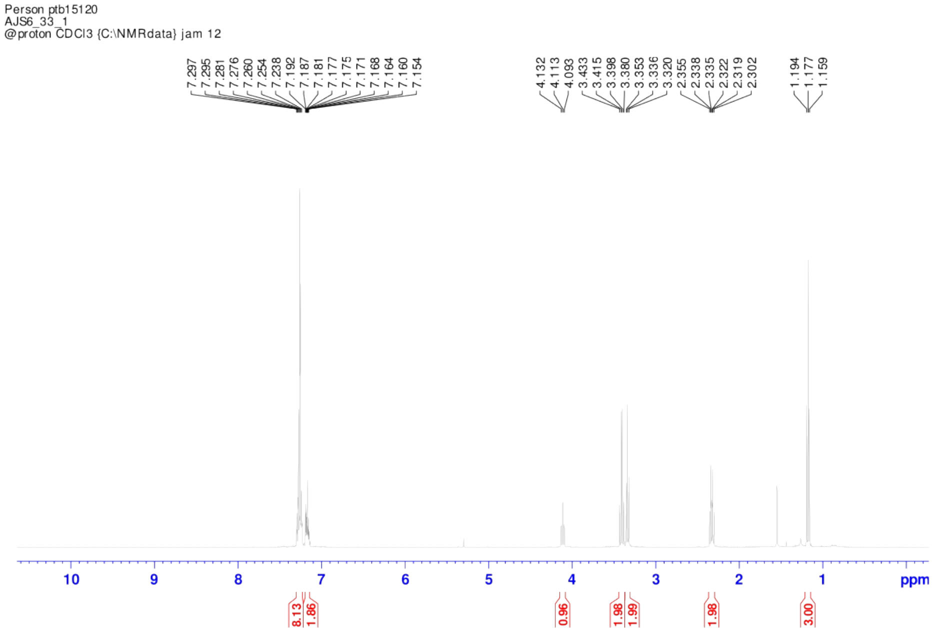

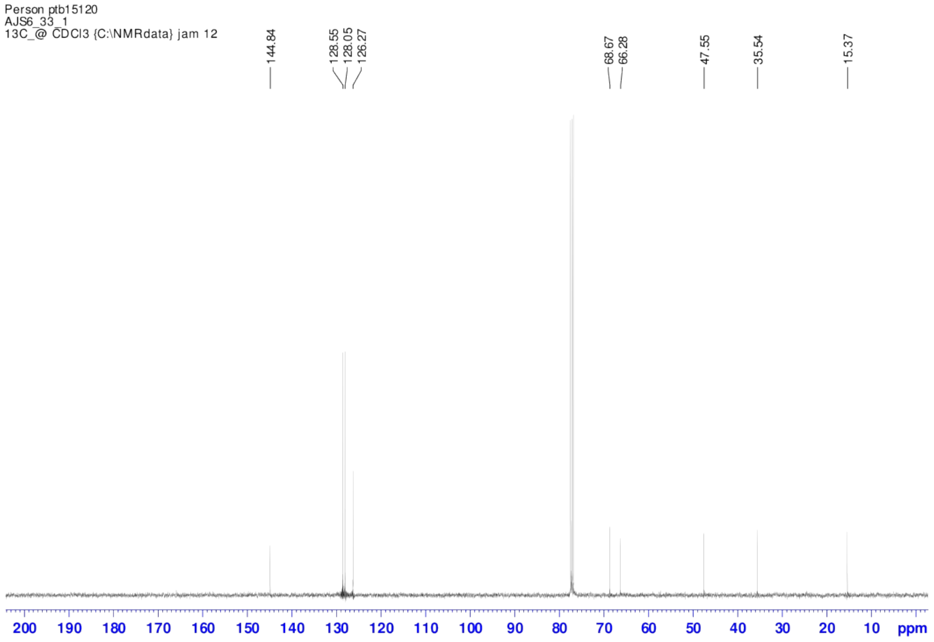

(3-Phenoxypropane-1,1-diyl)dibenzene S49

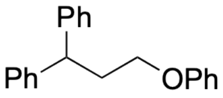

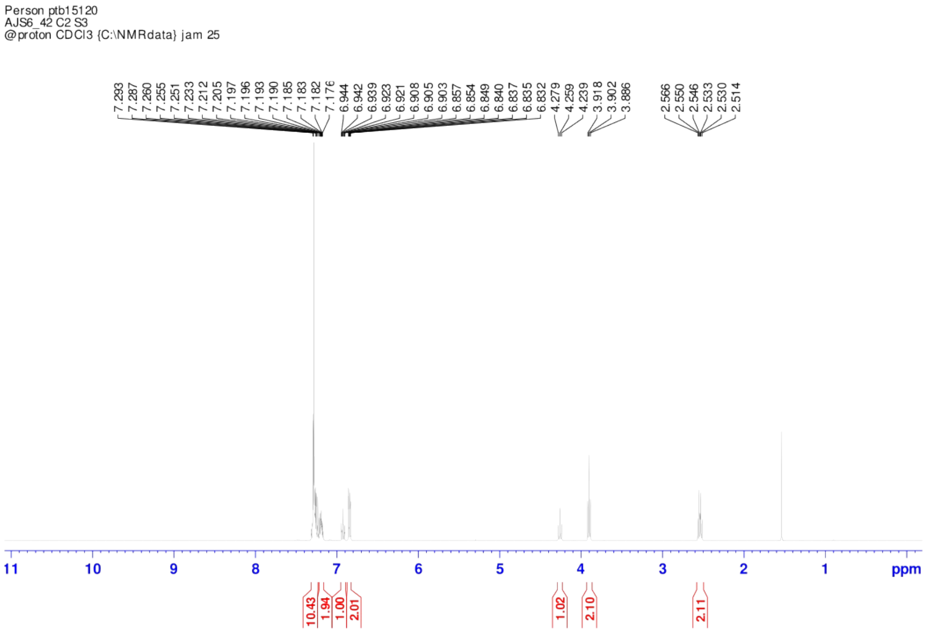

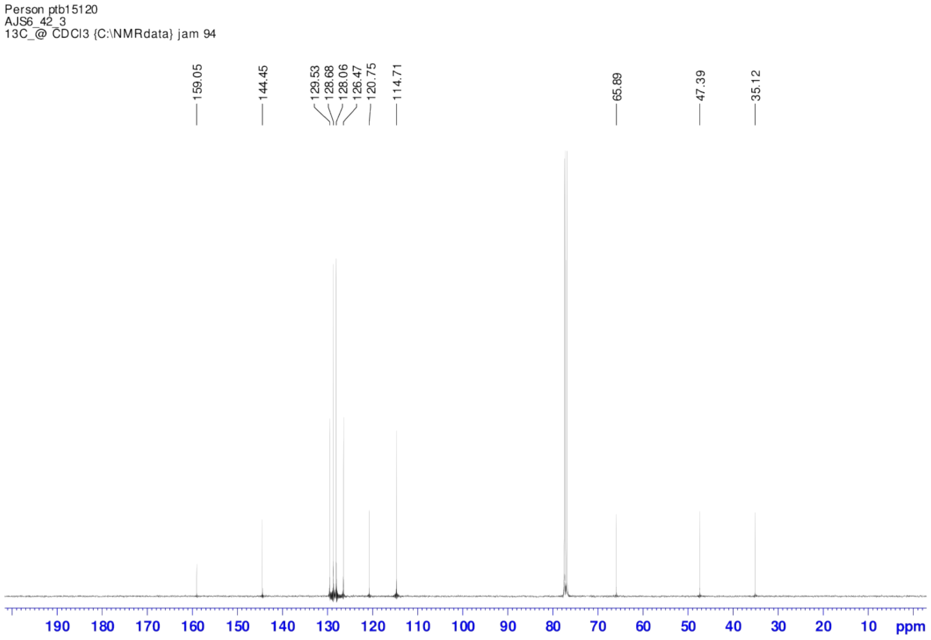

3,3-Diphenylpropyl methanesulfonate S50

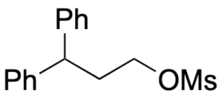

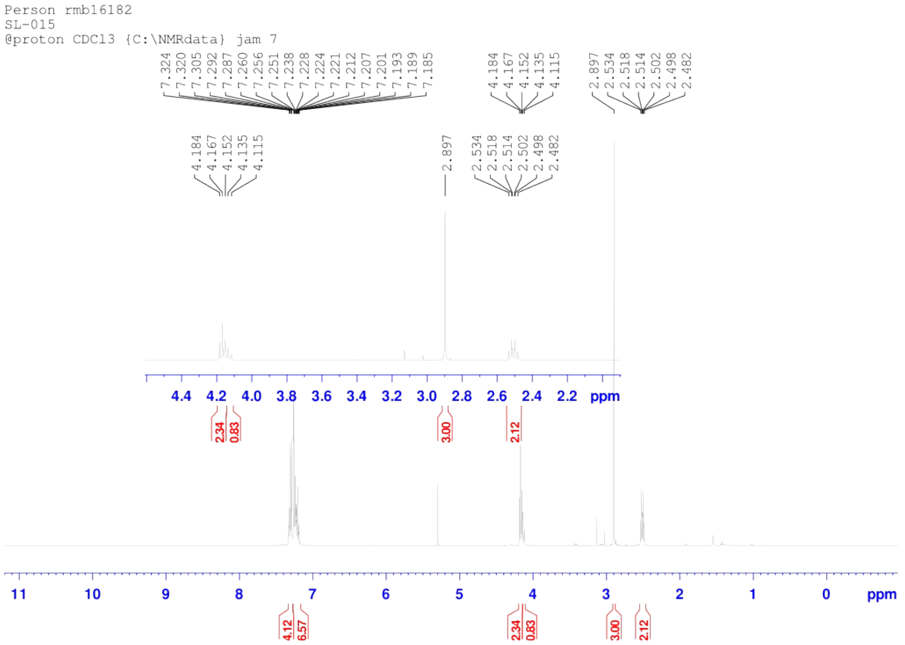

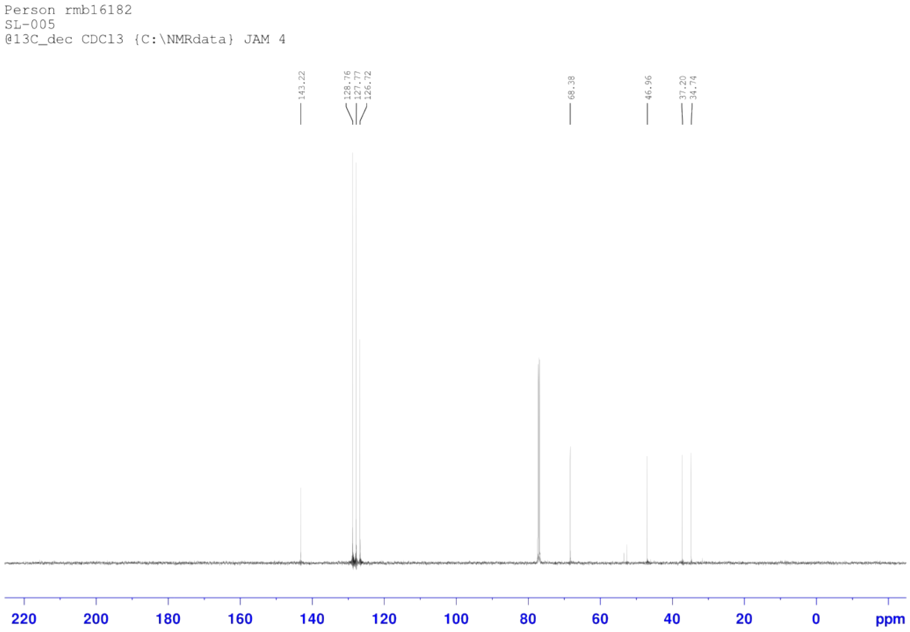

(3,3-Diphenylpropyl)(phenyl)sulfane 54

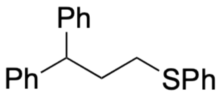

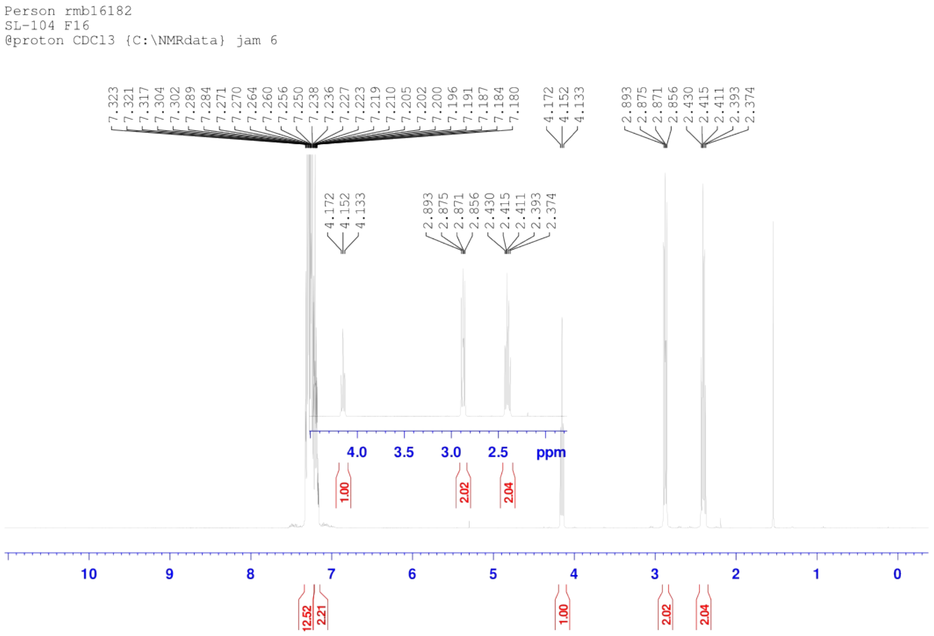

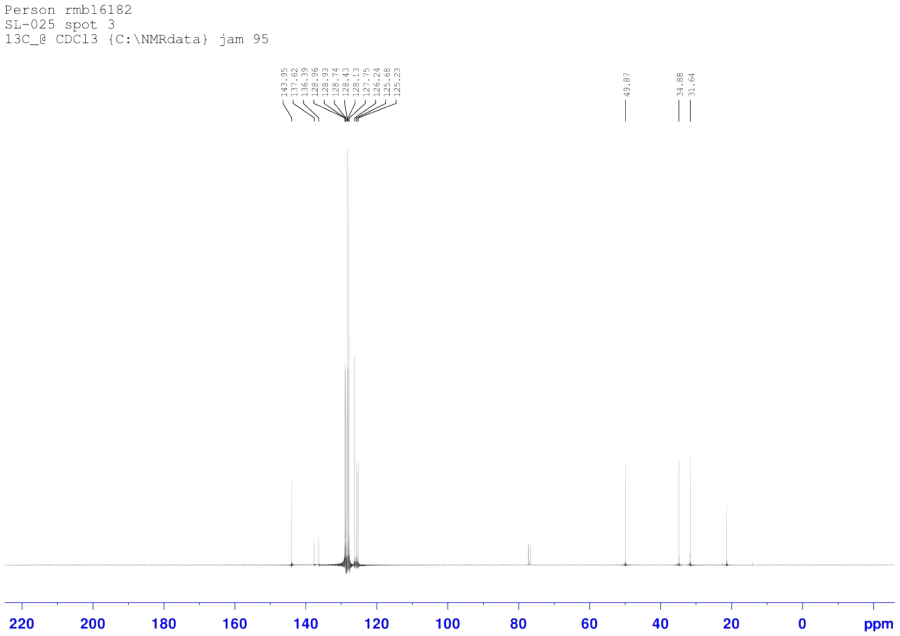

(3-(Phenylsulfinyl)propane-1,1-diyl)dibenzene 55

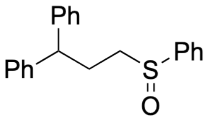

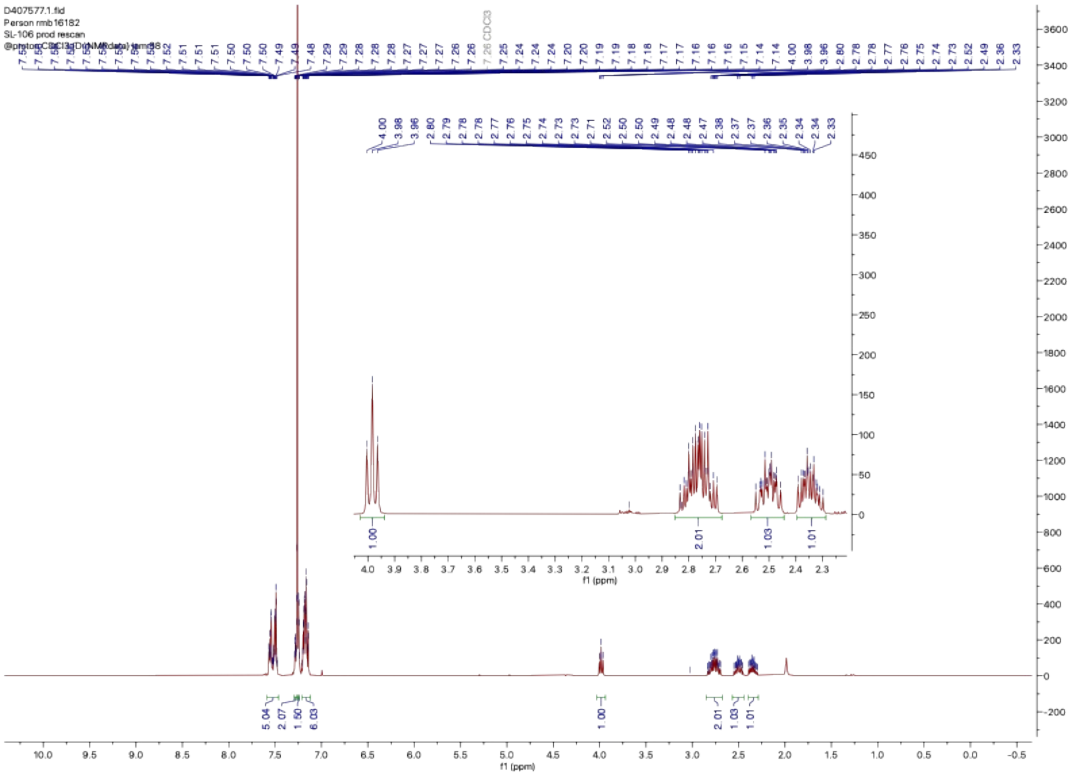

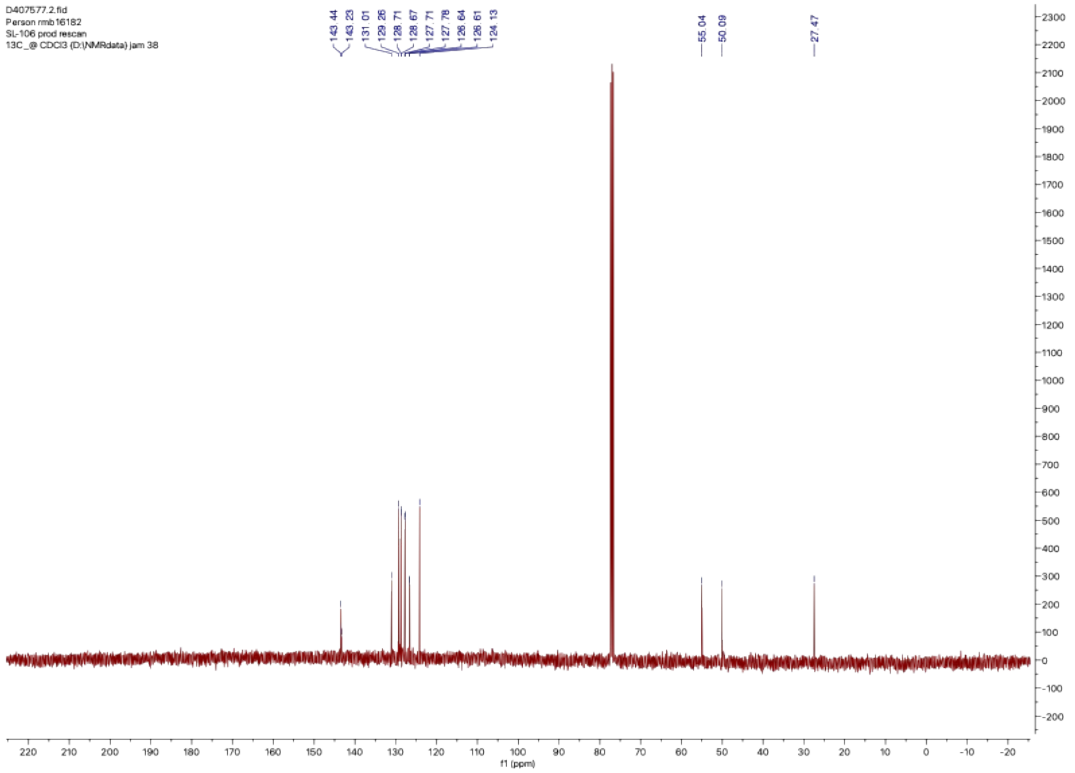

(3,3-Diphenylpropyl)(*p*-tolyl)sulfane 56

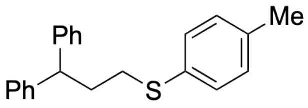

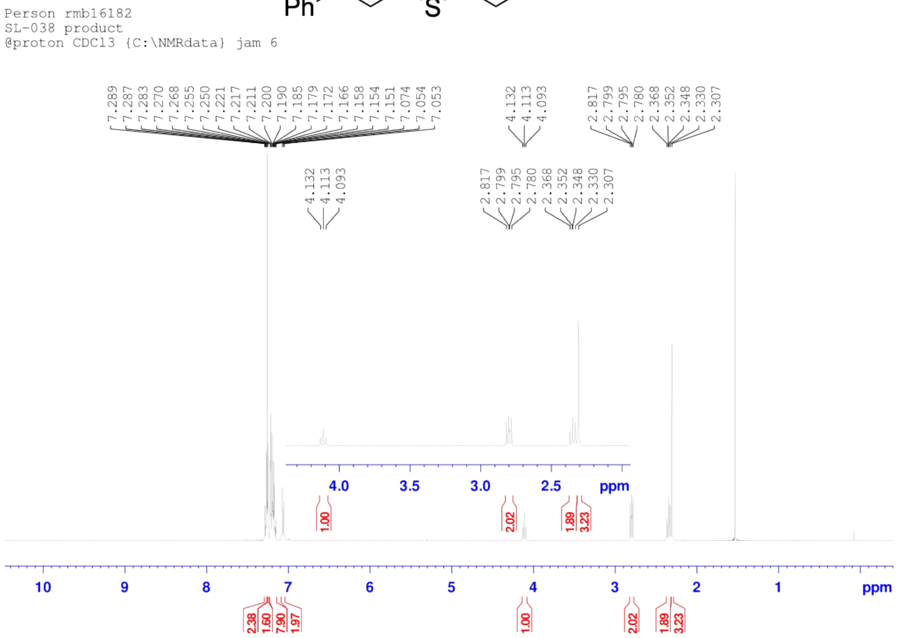

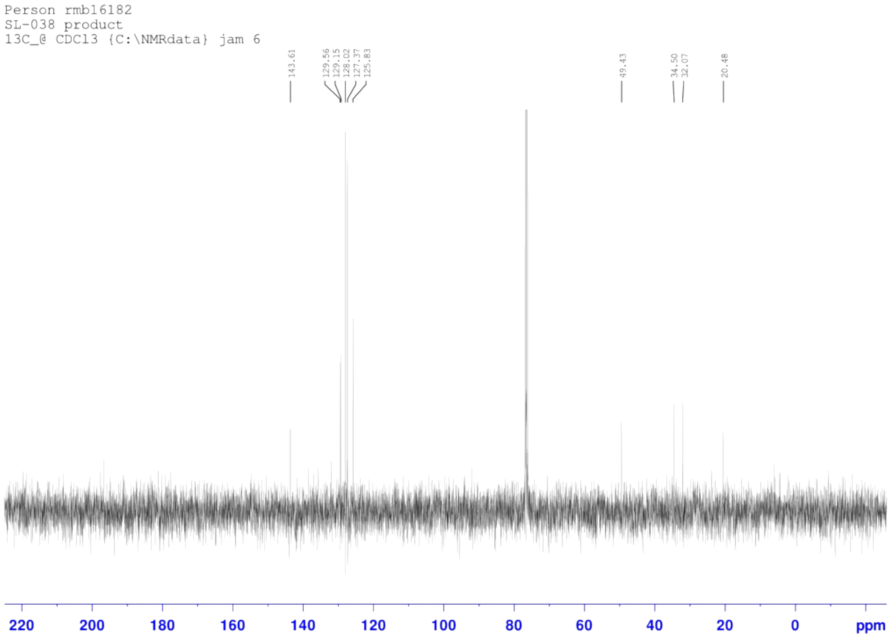

(3-(*p*-Tolylsulfinyl)propane-1,1-diyl)dibenzene 57

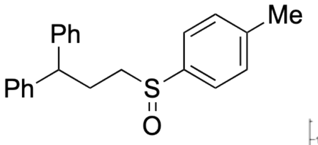

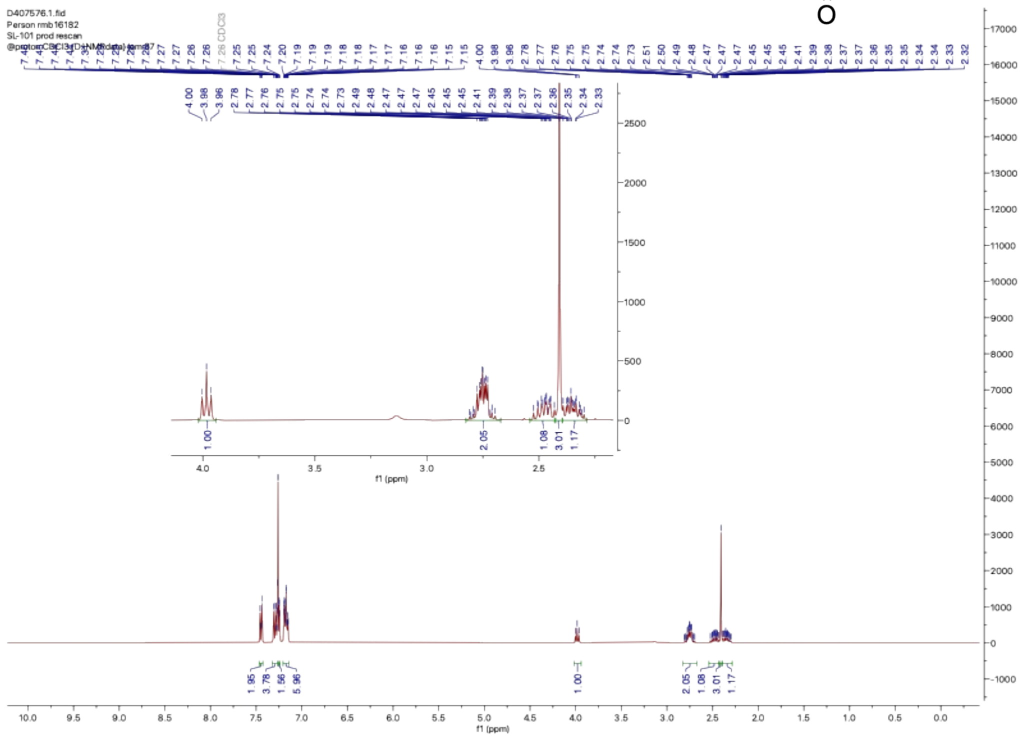

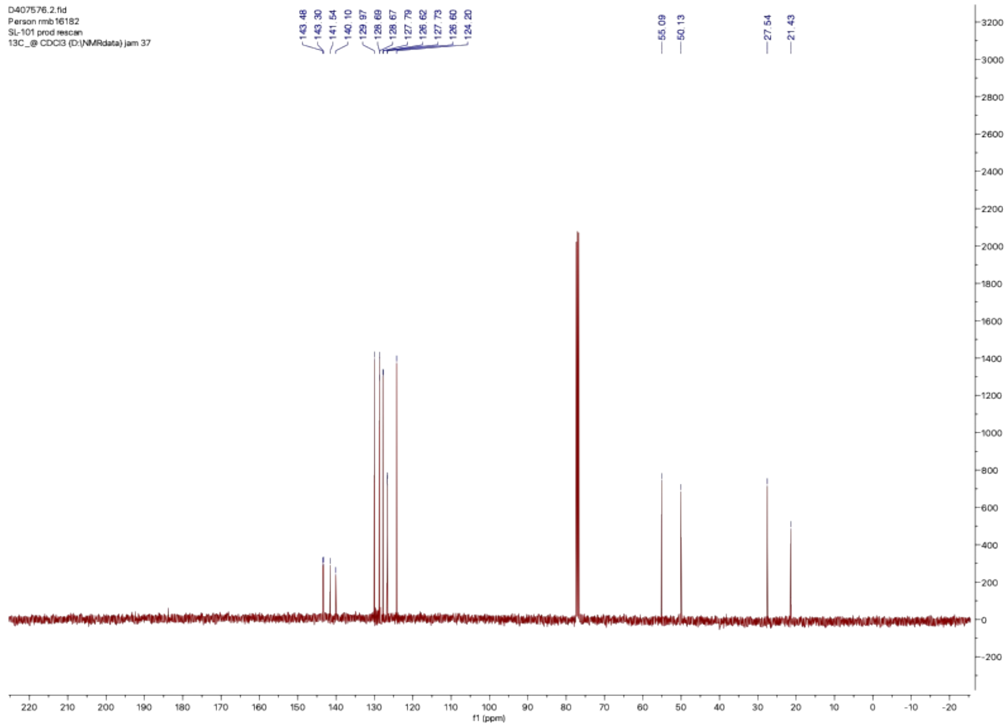

(3-Tosylpropane-1,1-diyl)dibenzene 58

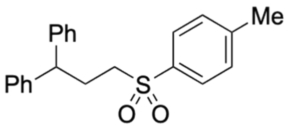

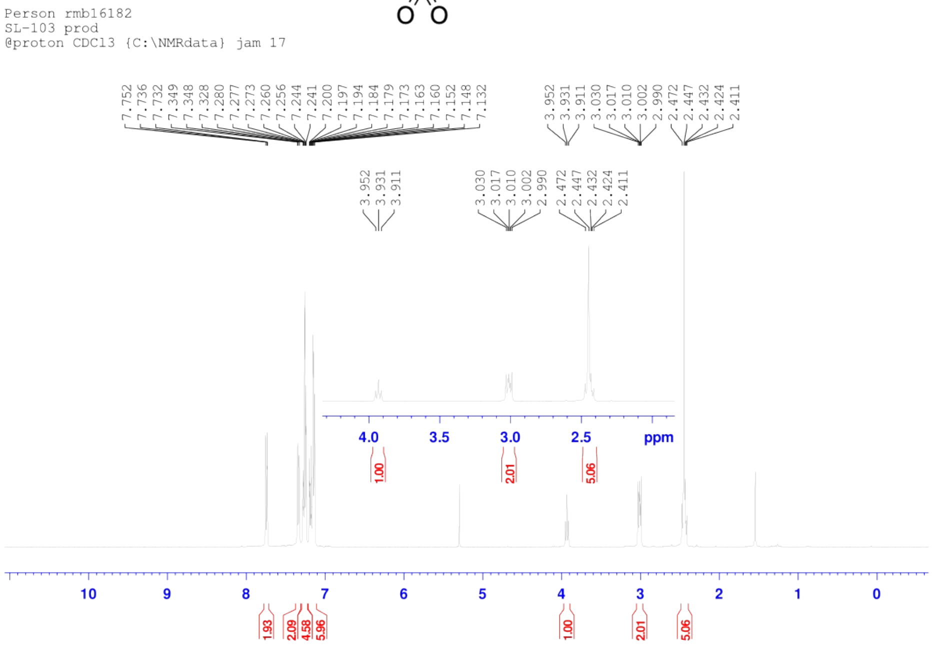

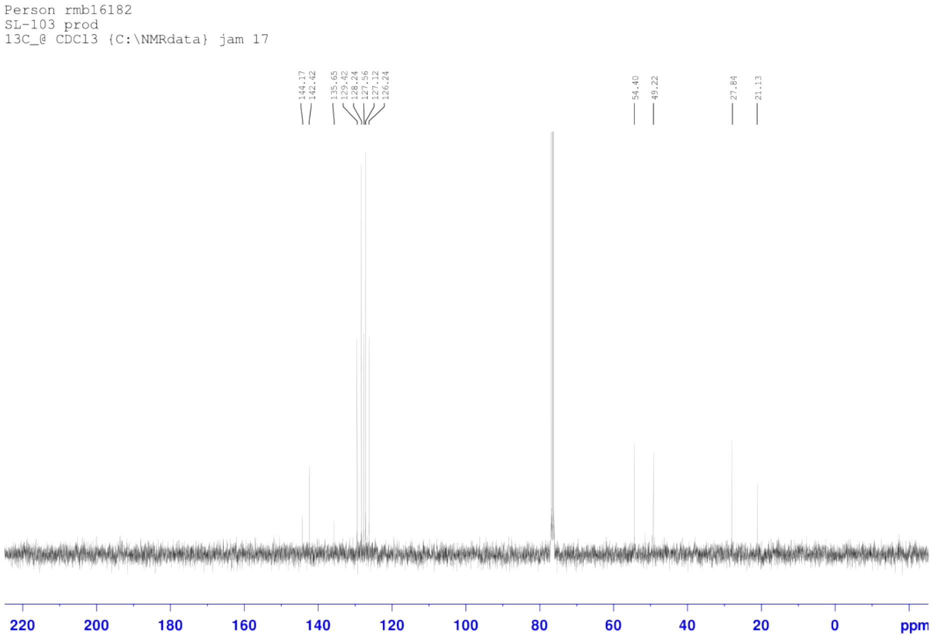

(3,3-diphenylpropyl)(methyl)sulfane 59

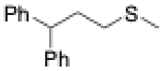

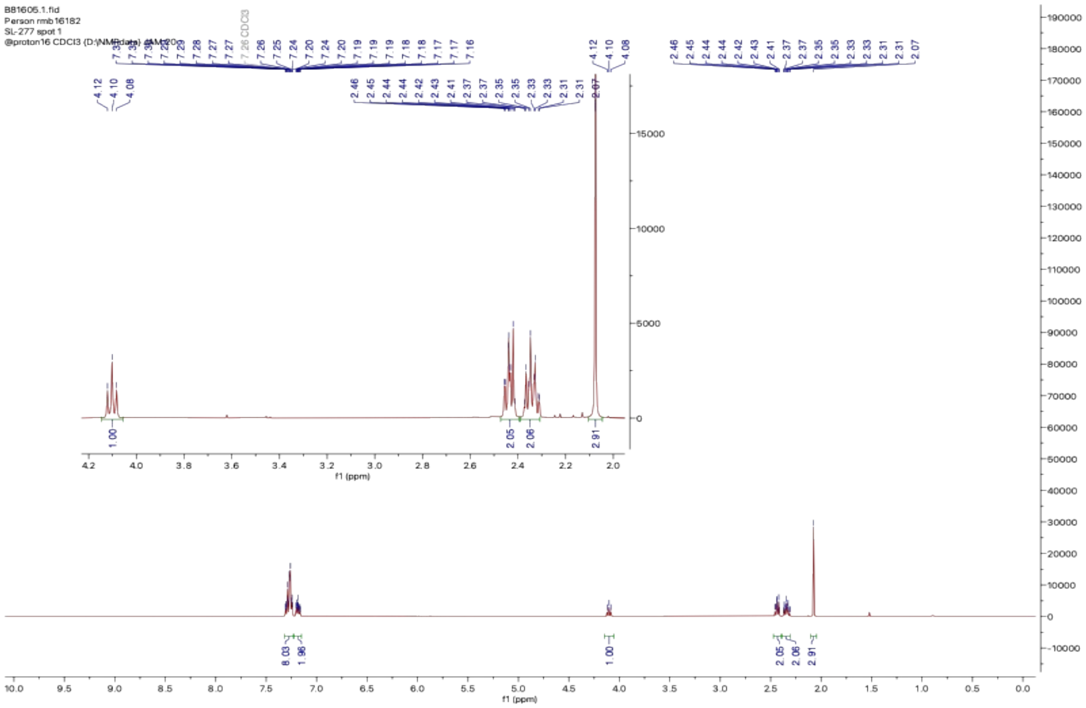

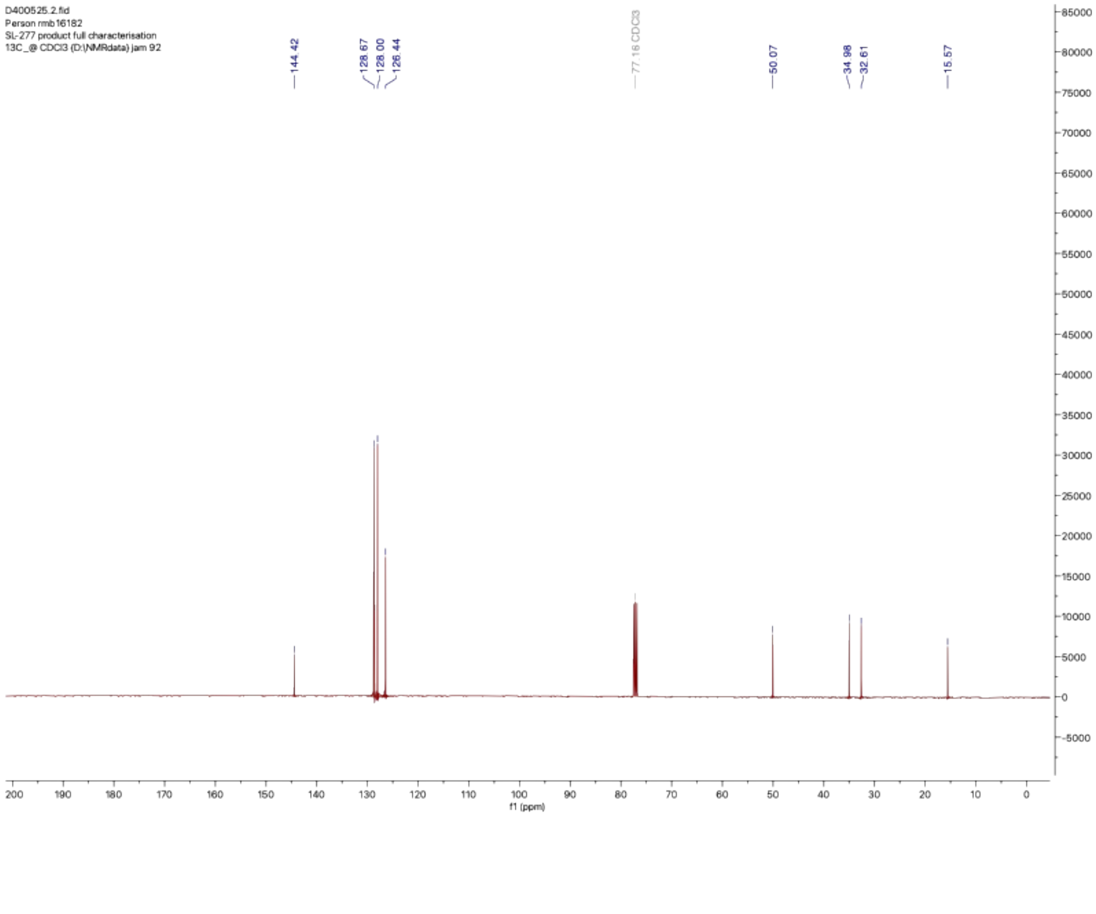

(3-(phenylsulfonyl)propane-1,1-diyl)dibenzene 60

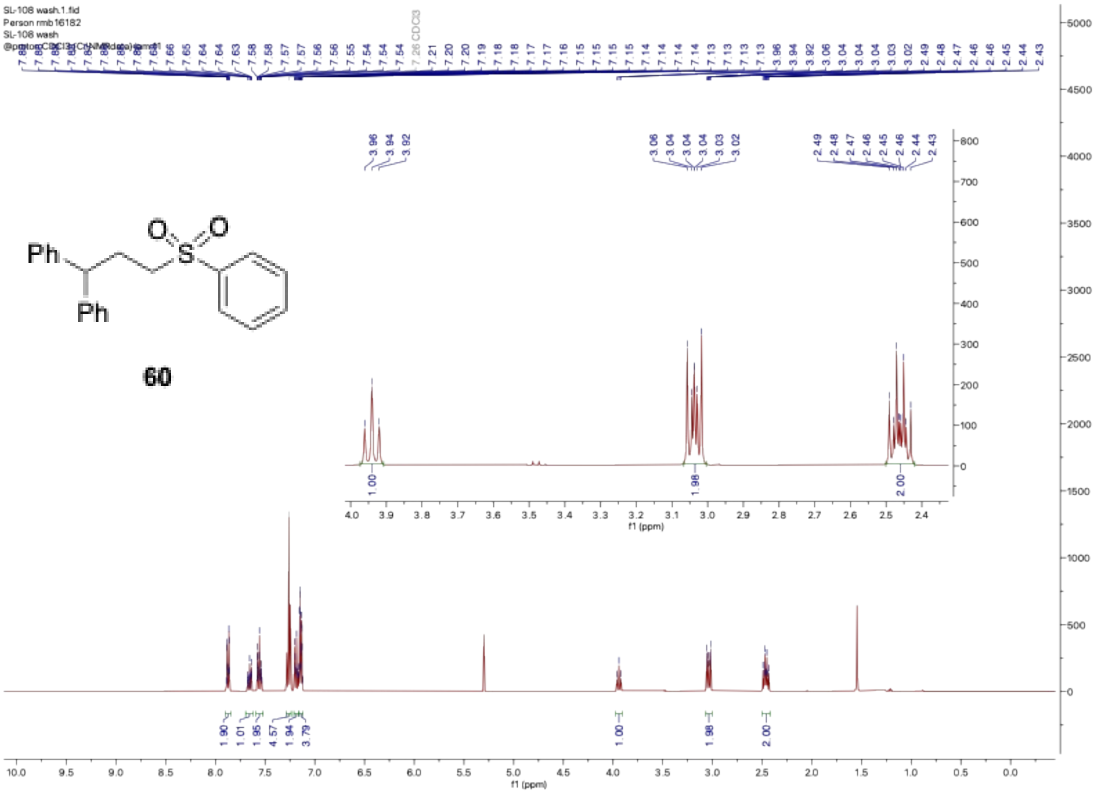

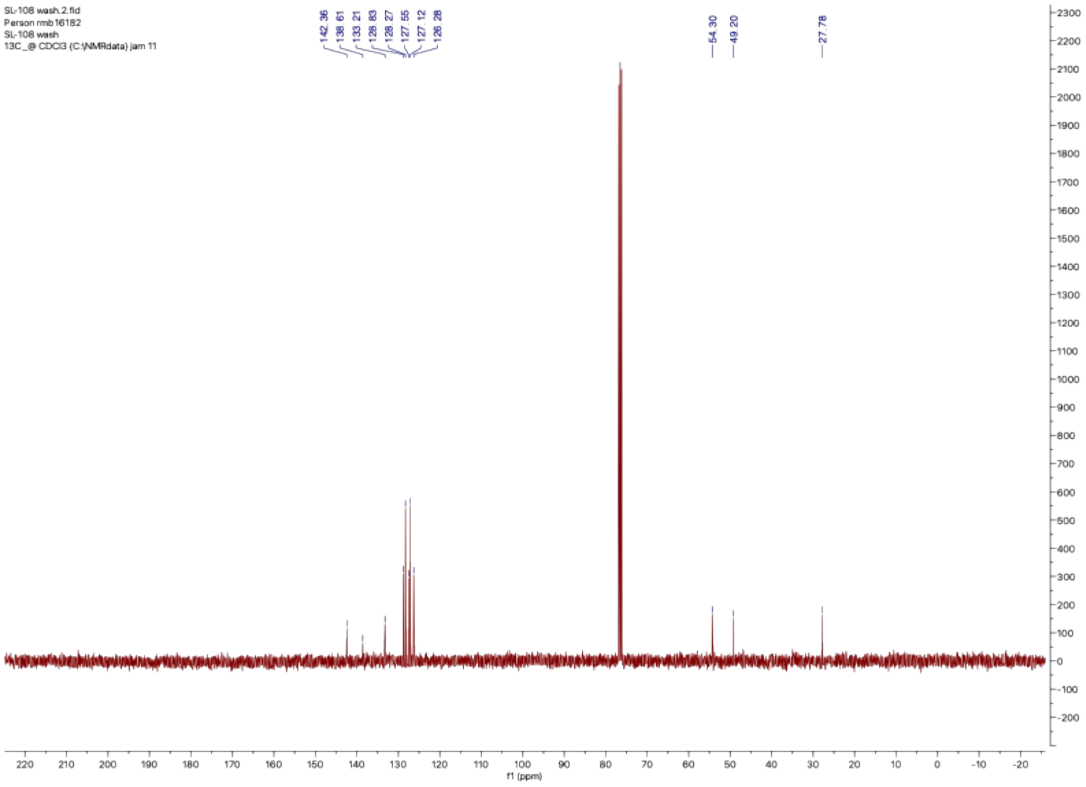

(3,3-diphenylpropyl)(4-(trifluoromethyl)phenyl)sulfane 61

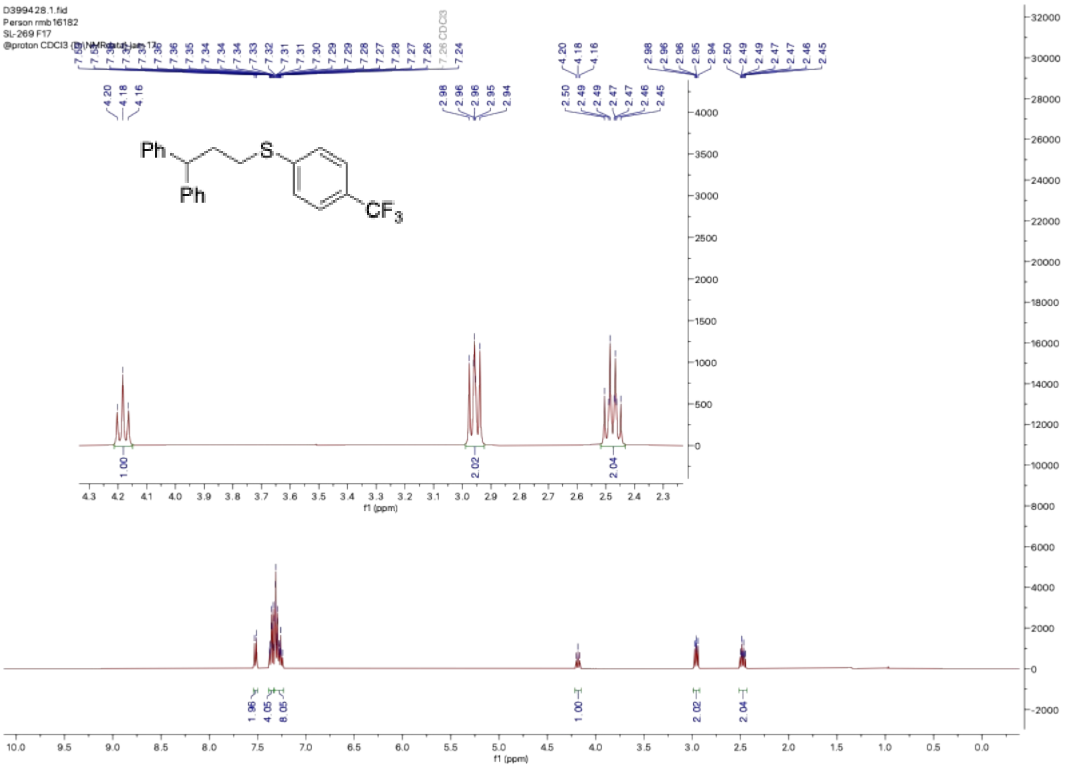

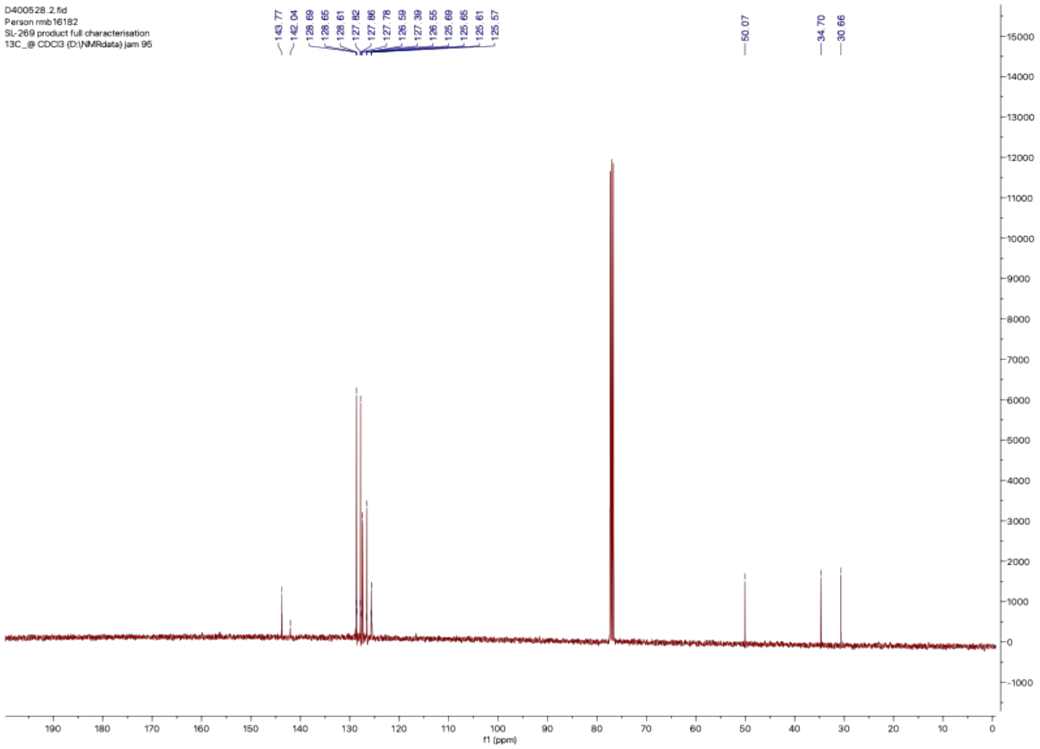

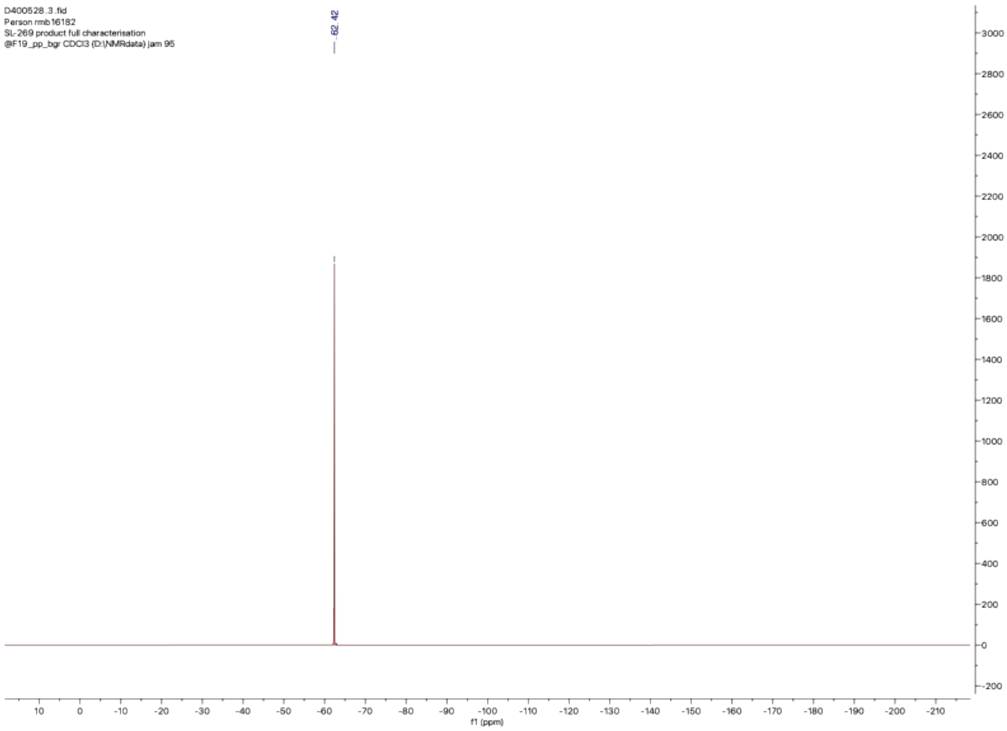
D400528.3.fid
Person rmb16182
SL-269 product full characterisation
@F19_pp_bgr CDCl3 (D:\NMRdata) jam 95
-62.42
3000
2800
2600
2400
2200
2000
1800
1600
1400
1200
1000
800
600
400
200
0
-200
10
0
-10
-20
-30
-40
-50
-60
-70
-80
-90
-100
-110
-120
-130
-140
-150
-160
-170
-180
-190
-200
-210
f1 (ppm)

(3,3-diphenylpropyl)(4-methoxyphenyl)sulfane 62

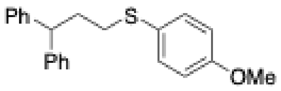

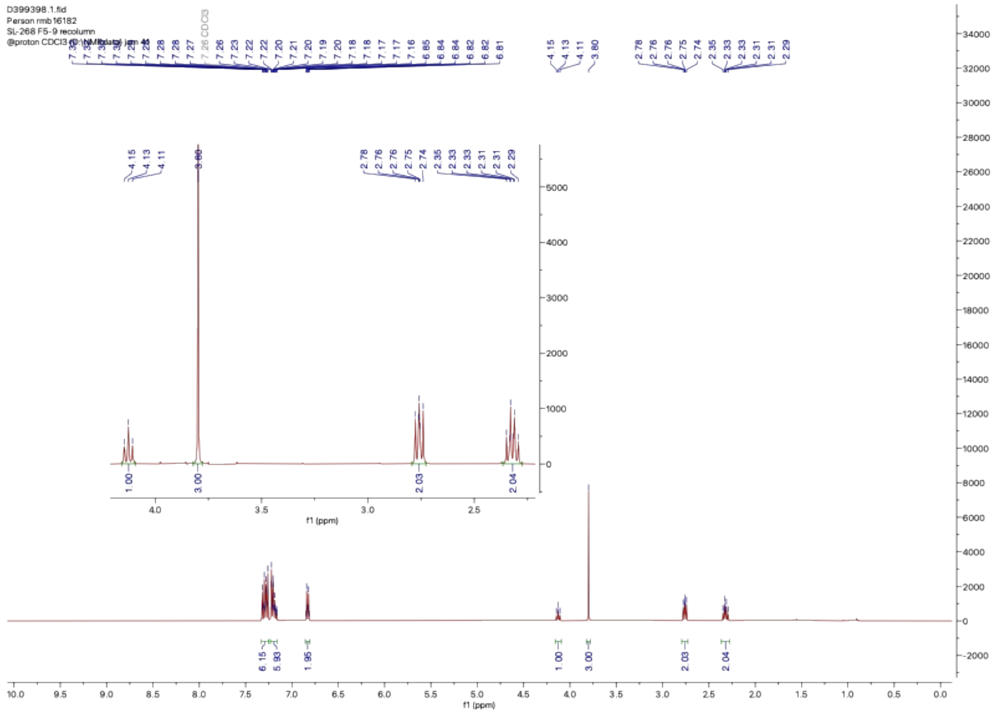

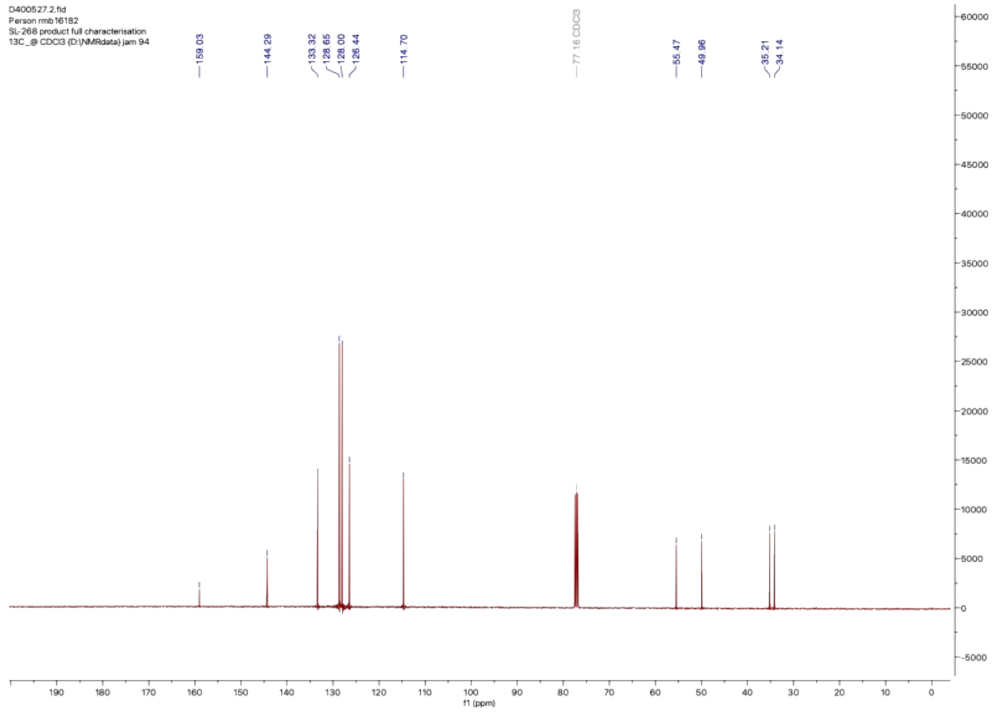

(3-((4-methoxyphenyl)sulfinyl)propane-1,1-diyl)dibenzene 63

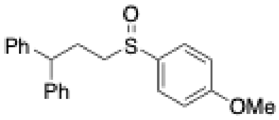

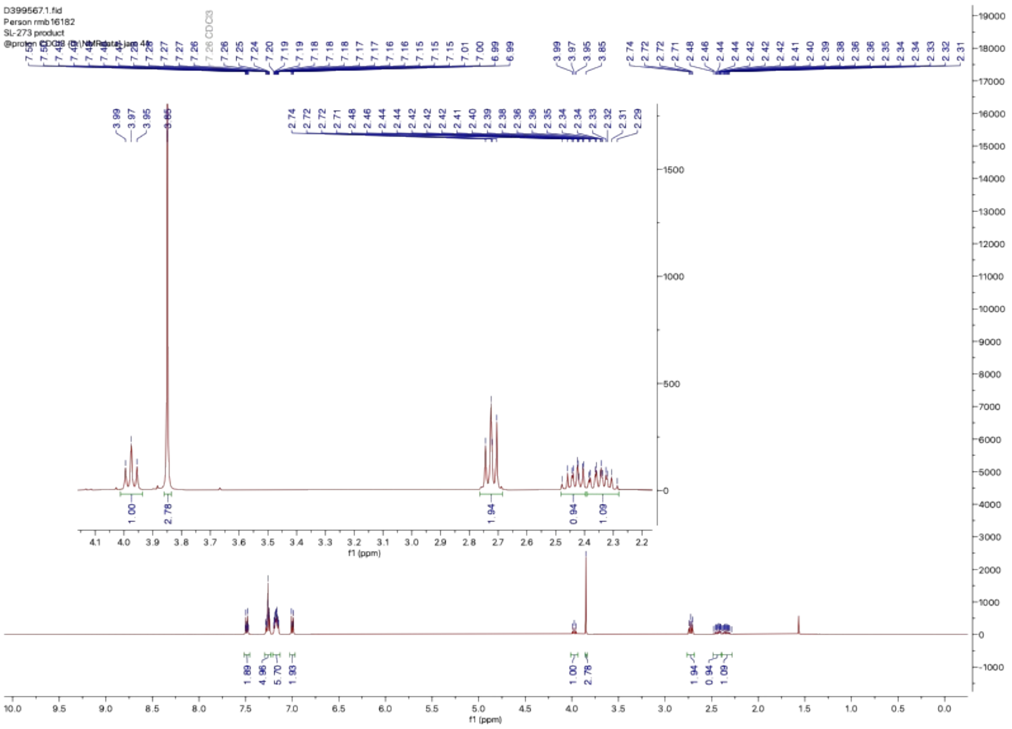

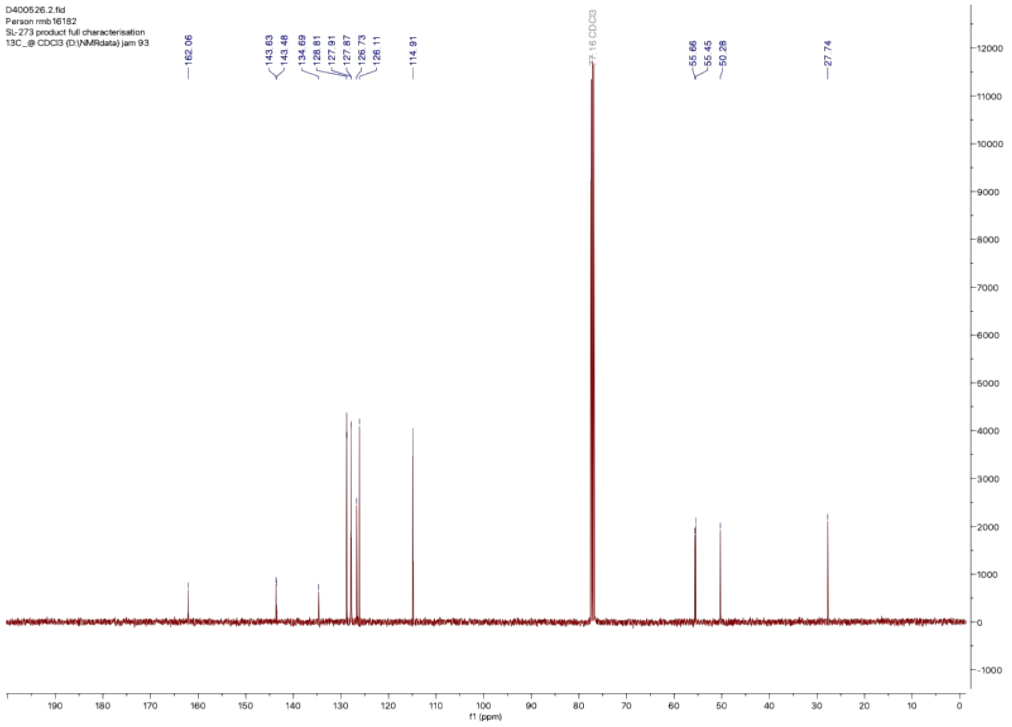

3-(4-(*Tert*-butyl)phenyl)propyl methanesulfonate S51

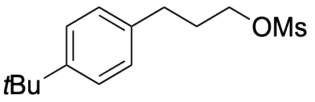

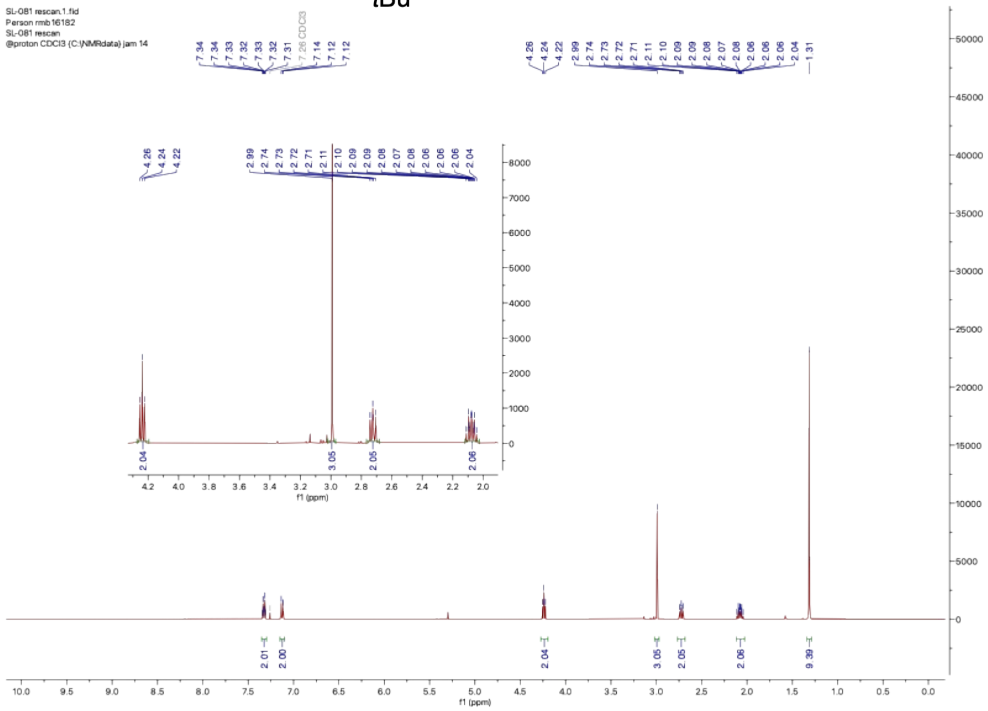

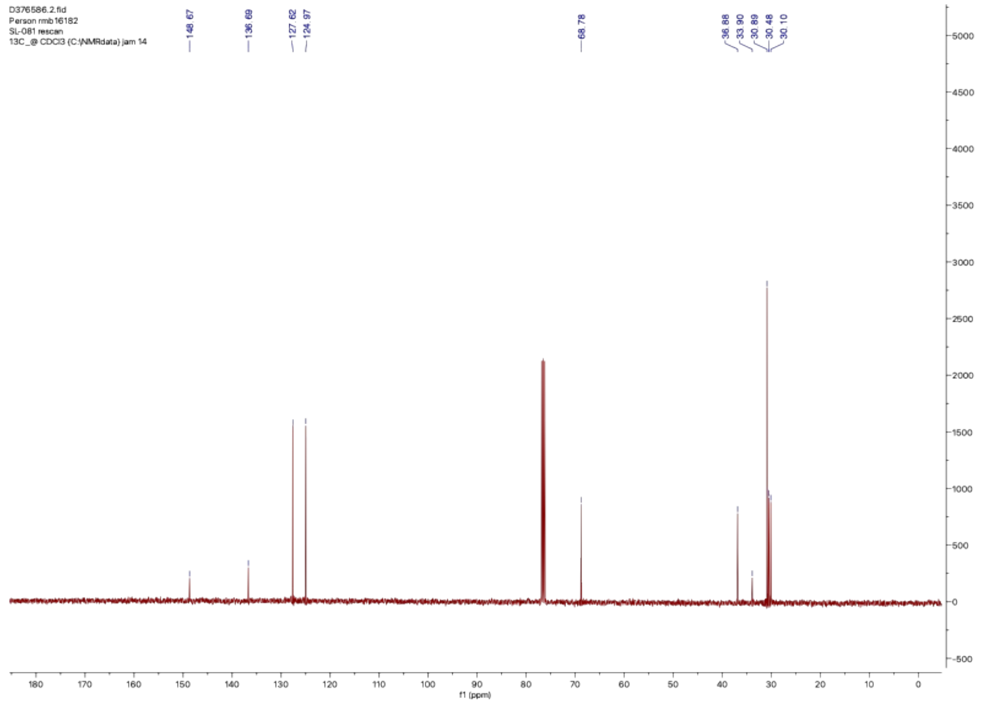

(3-(4-(*Tert*-butyl)phenyl)propyl)(*p*-tolyl)sulfane 64

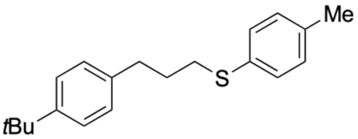

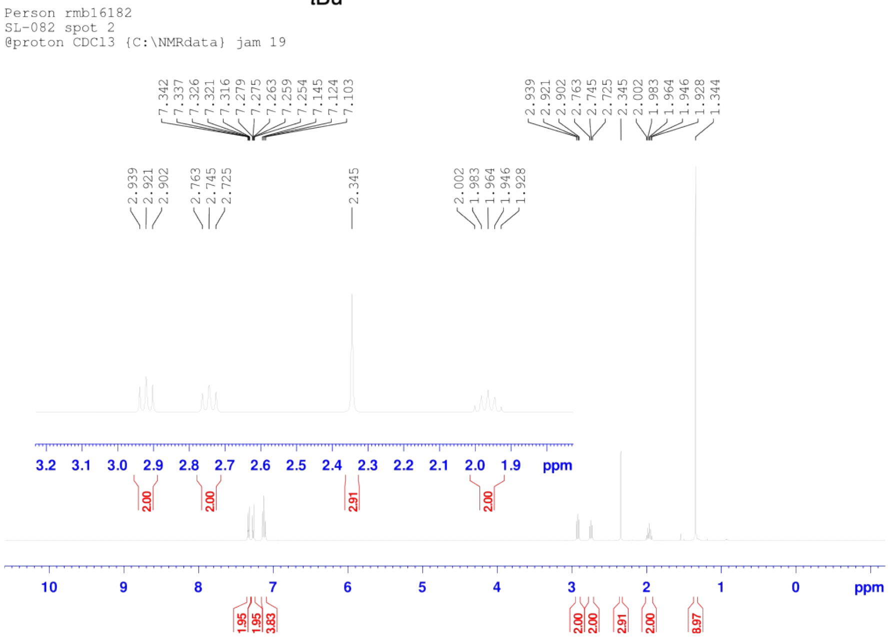

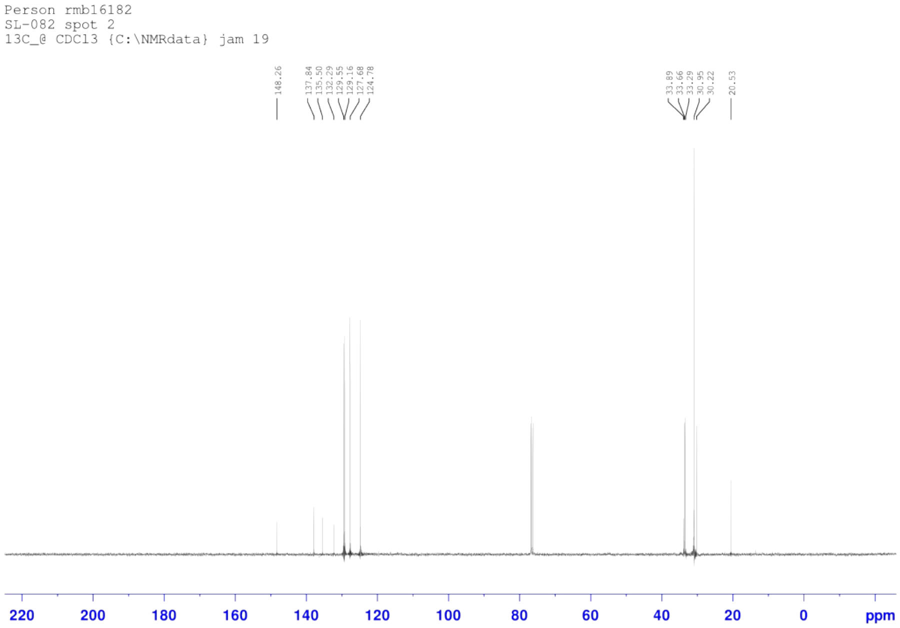

3-(Naphthalen-2-yl)propyl methanesulfonate S52

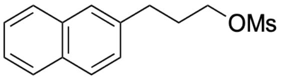

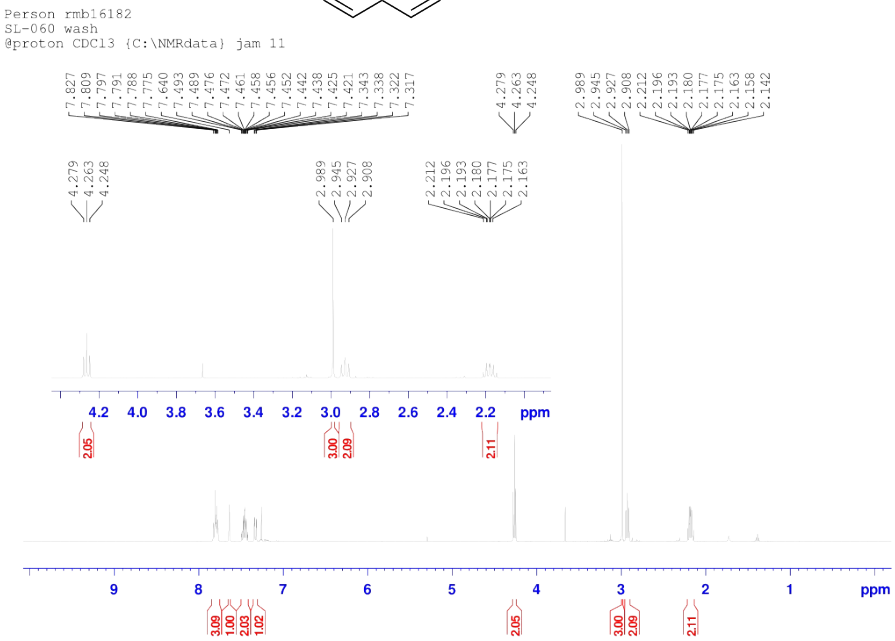

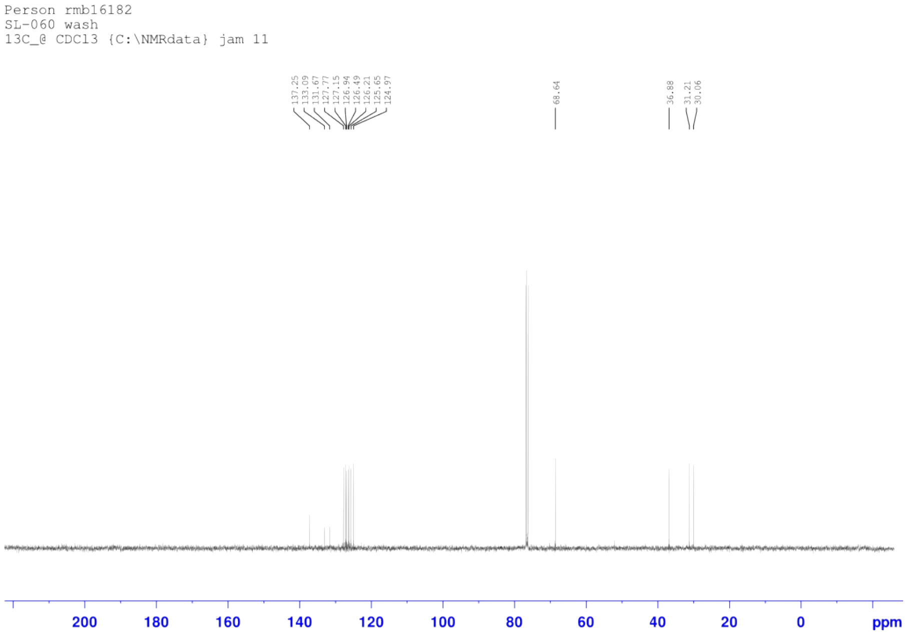

(3-(Naphthalen-2-yl)propyl)(*p*-tolyl)sulfane S53

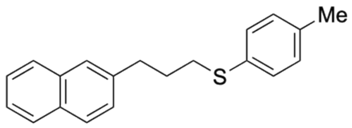

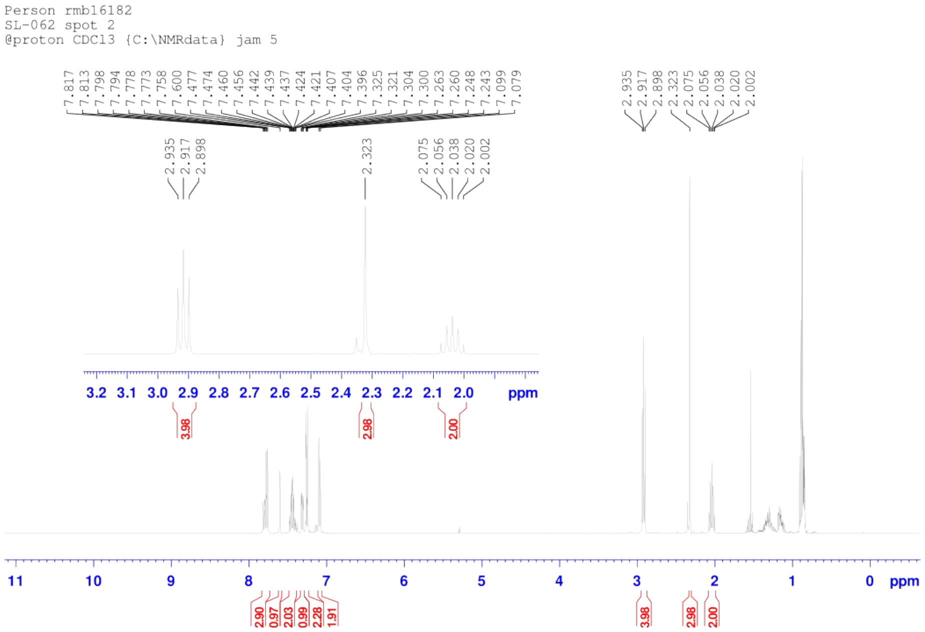

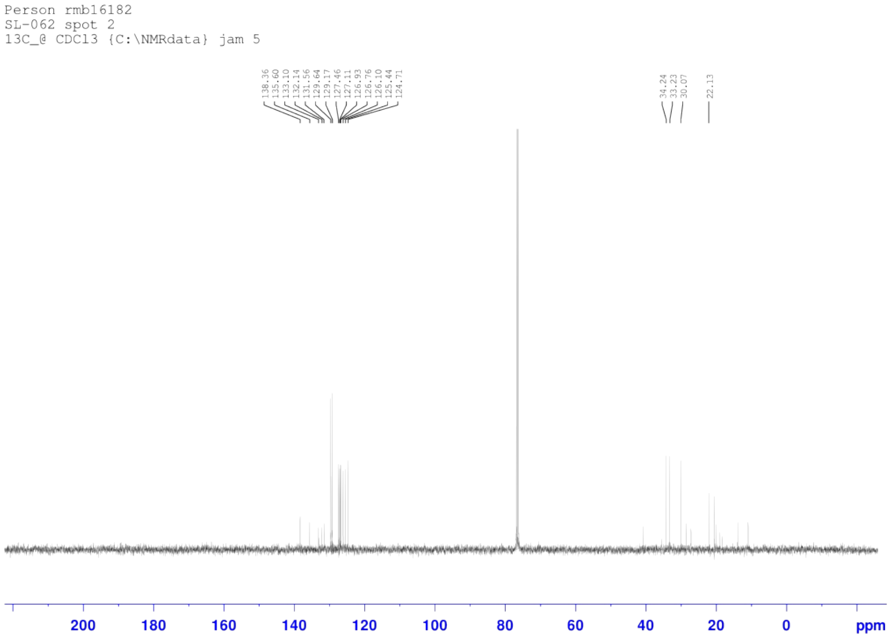

2-(3-(*p*-Tolylsulfinyl)propyl)naphthalene 65

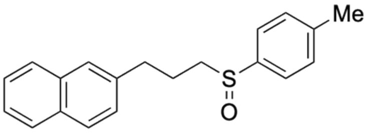

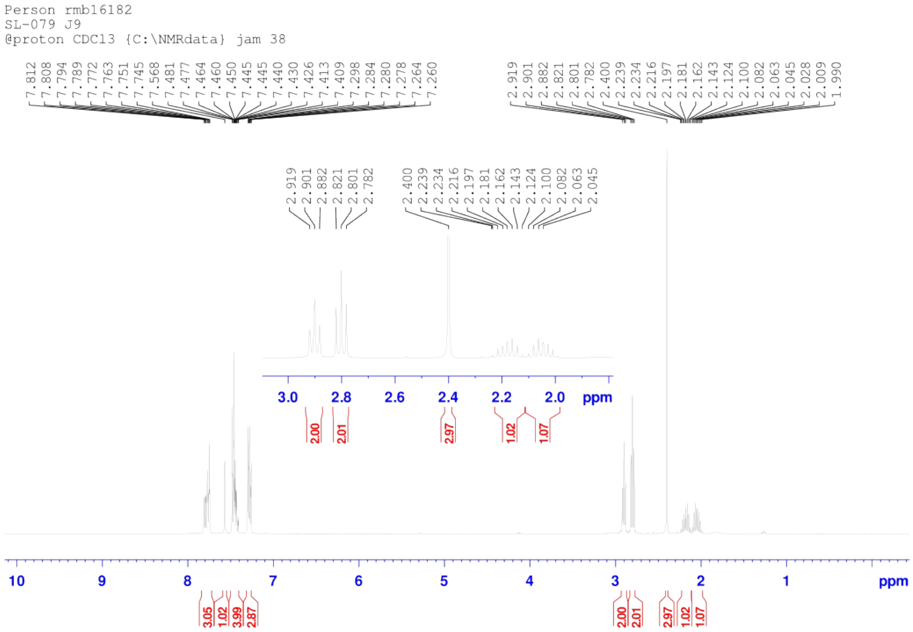

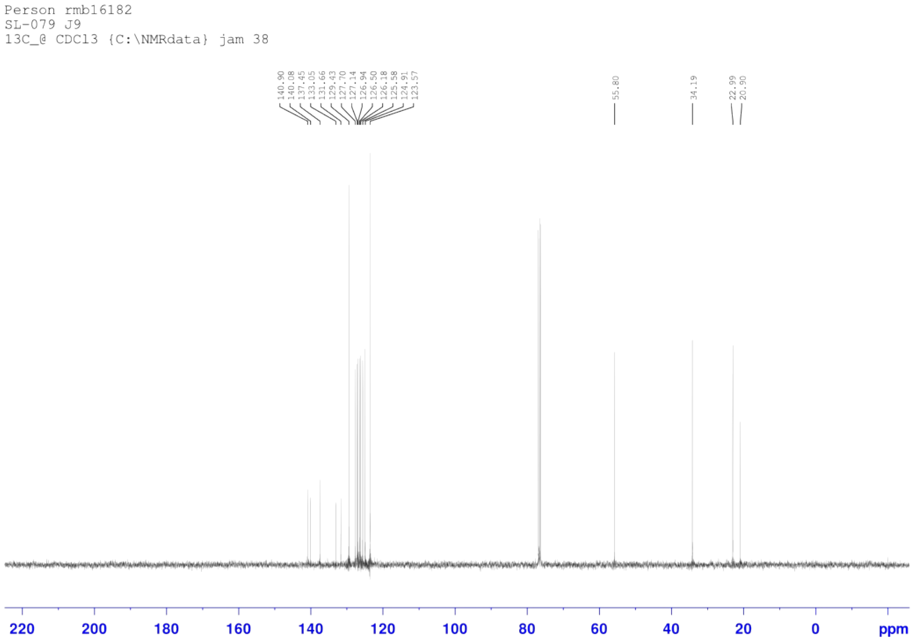

9-(2-(Phenylthio)ethyl)-9*H*-xanthene 66

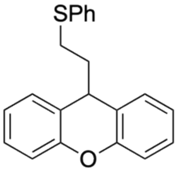

Person rmb16182
SL-042 V38
@proton CDCl3 {C:\NMRdata} jam 22

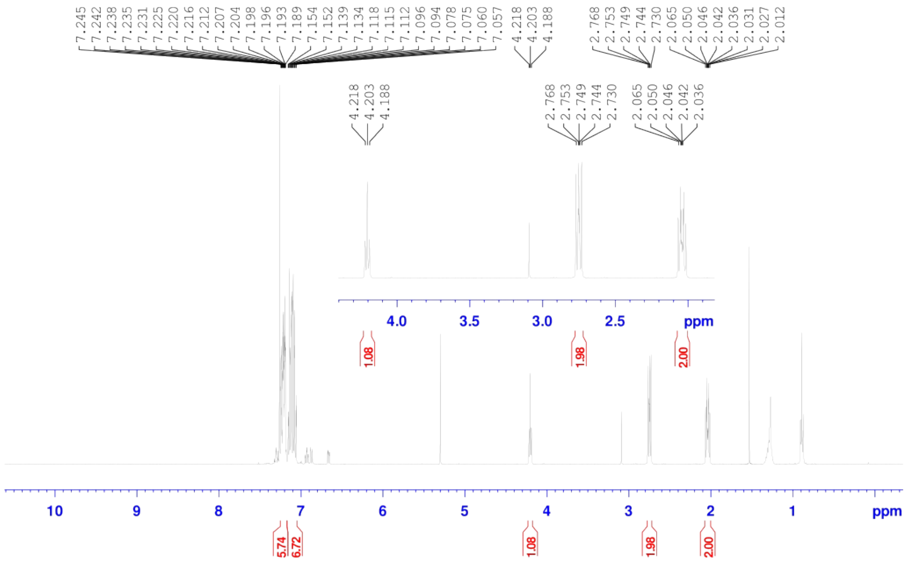

Person rmb16182
SL-042 V38
13C_@ CDCl3 {C:\NMRdata} jam 22

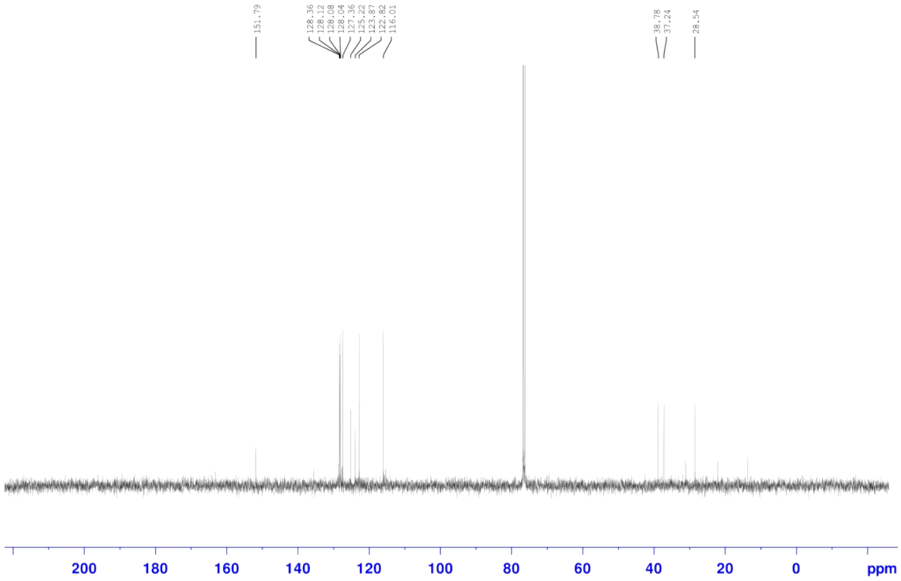

Product Spectra

2-Benzyl-*N*-methylaniline 12

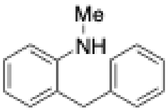

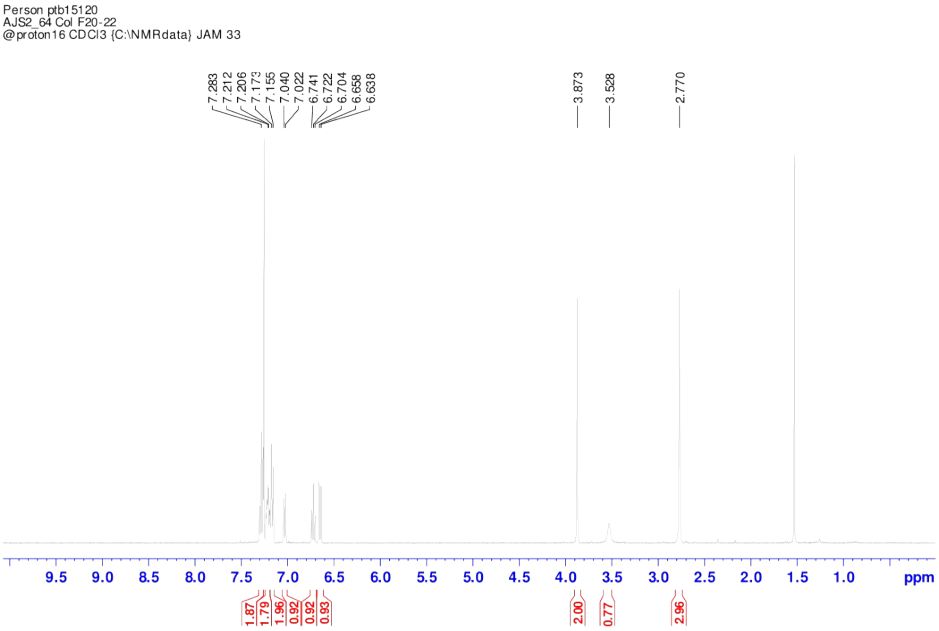

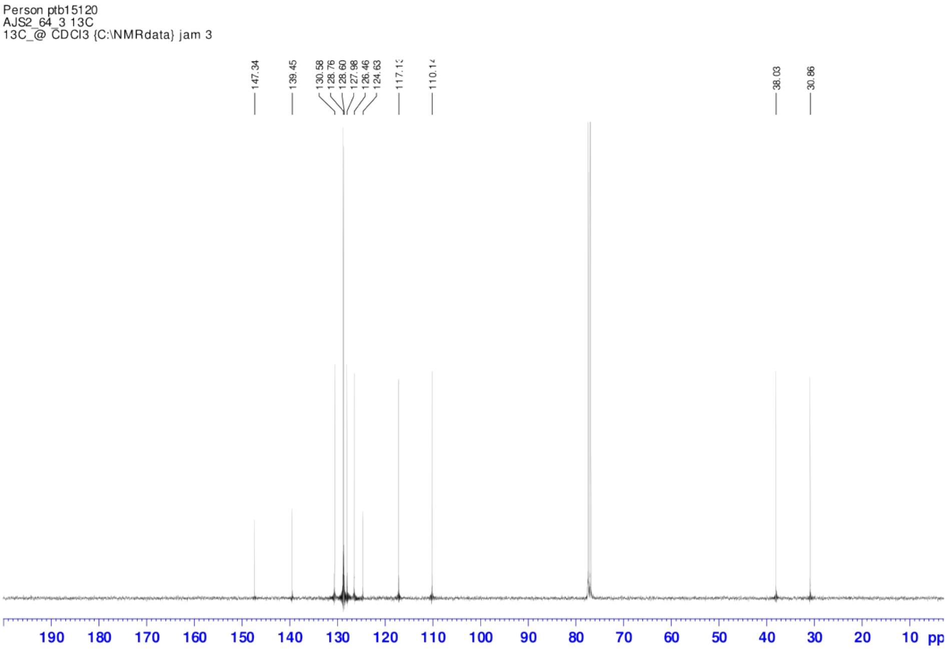

10-Methyl-10*H*-spiro[acridine-9,1'-cyclopentane] 14

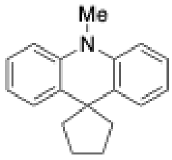

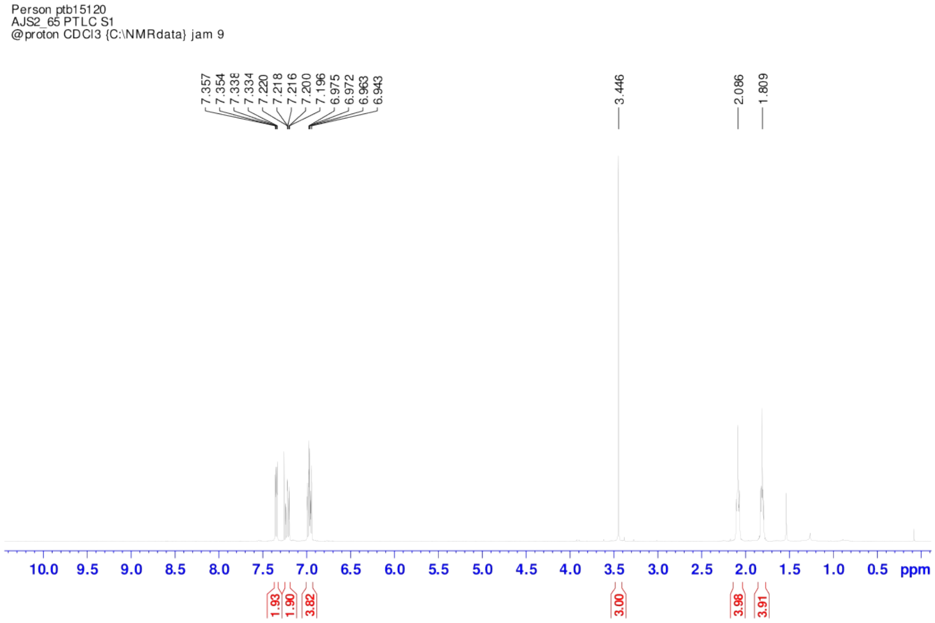

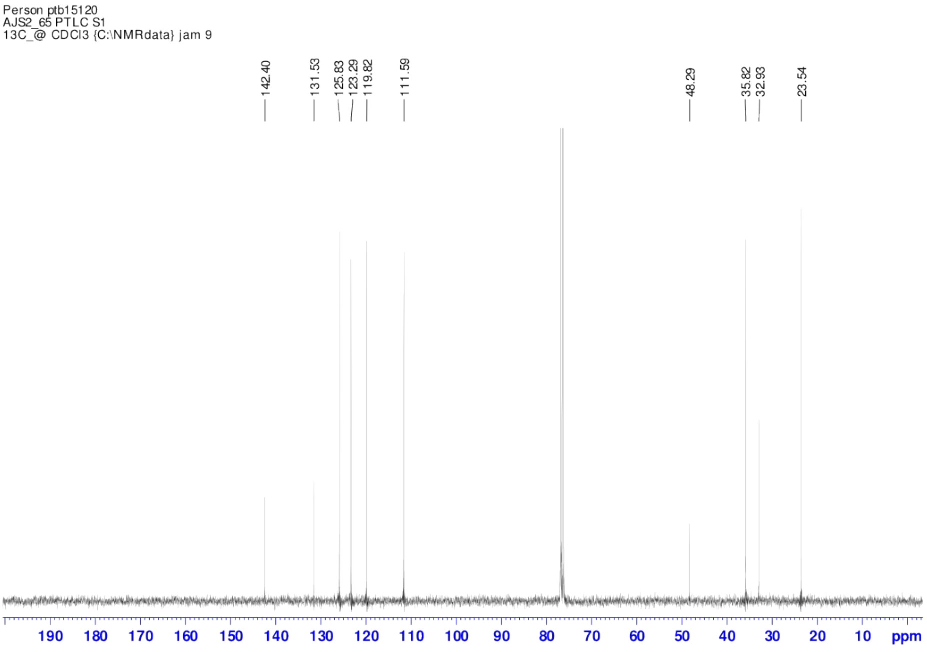

10-Methyl-10*H*-spiro[acridine-9,1'-cyclopentane]-2',2',3',3',4',4',5',5'-d_8 $d_8$14

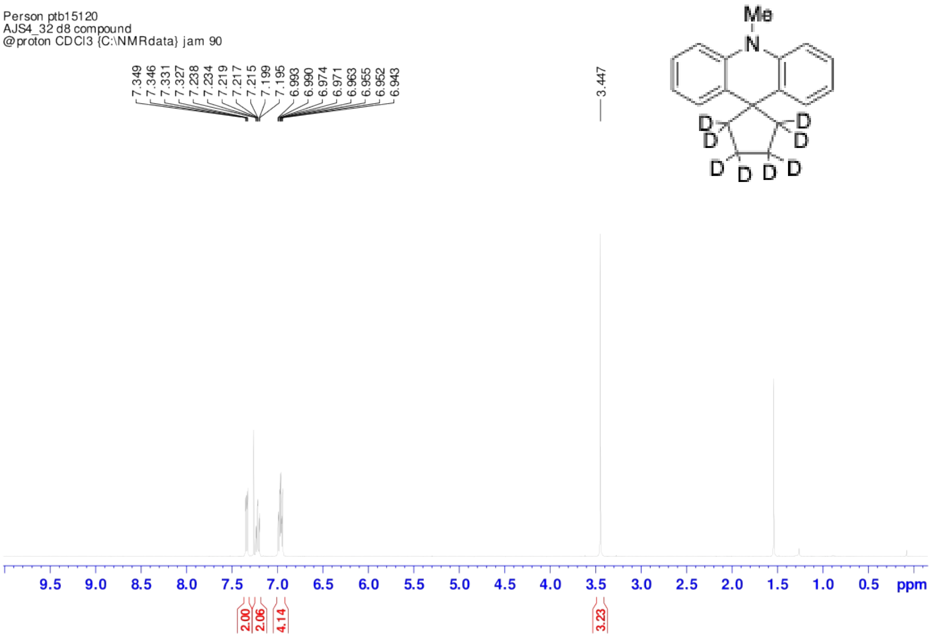

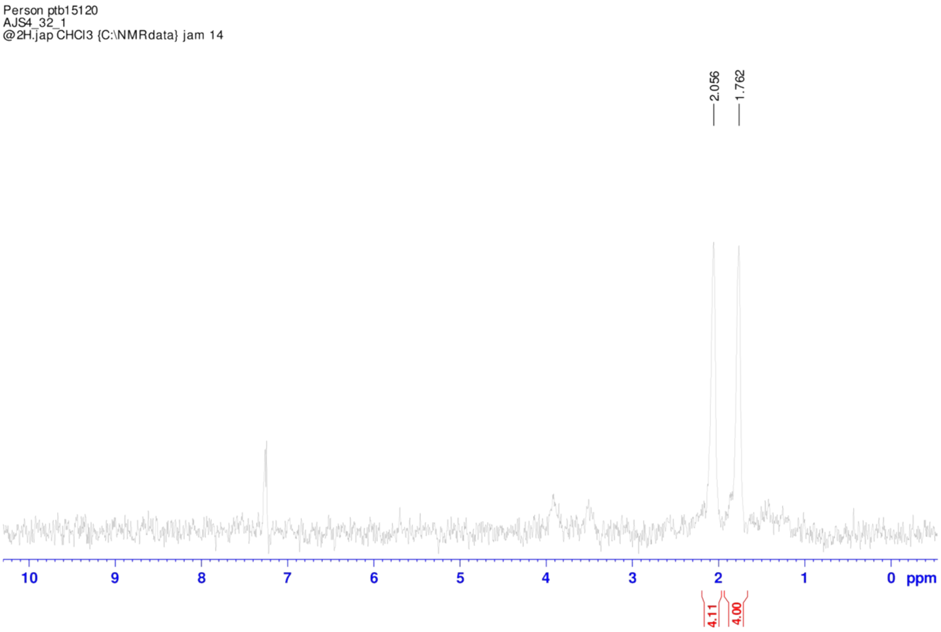

Person ptb15120
AJS4_32 d8 compound
13C_@ CDCl3 {C:\NMRdata} jam 90

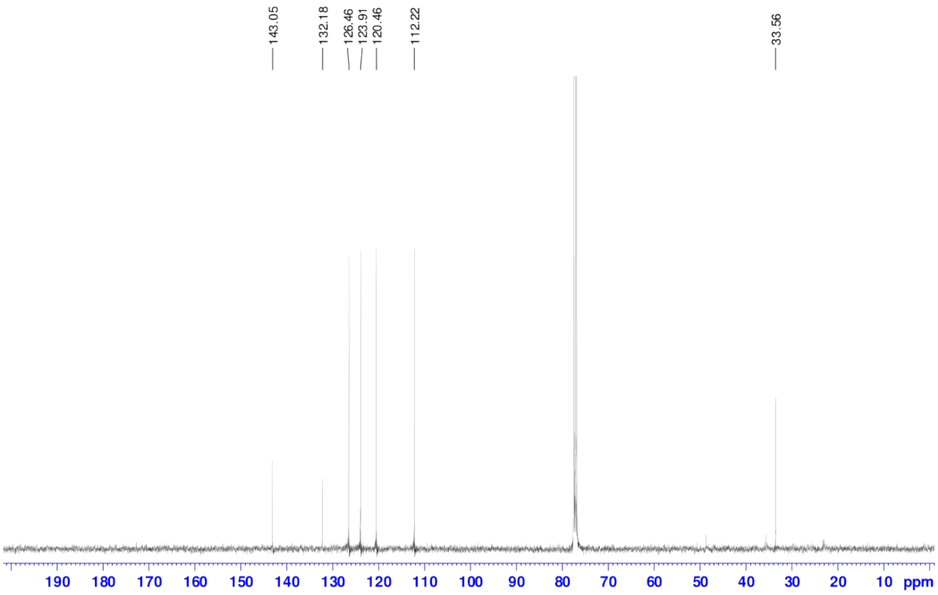

10,10'-Dimethyl-9,9',10,10'-tetrahydro-9,9'-biacridine 15

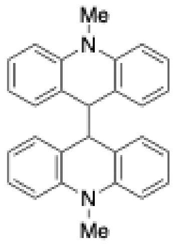

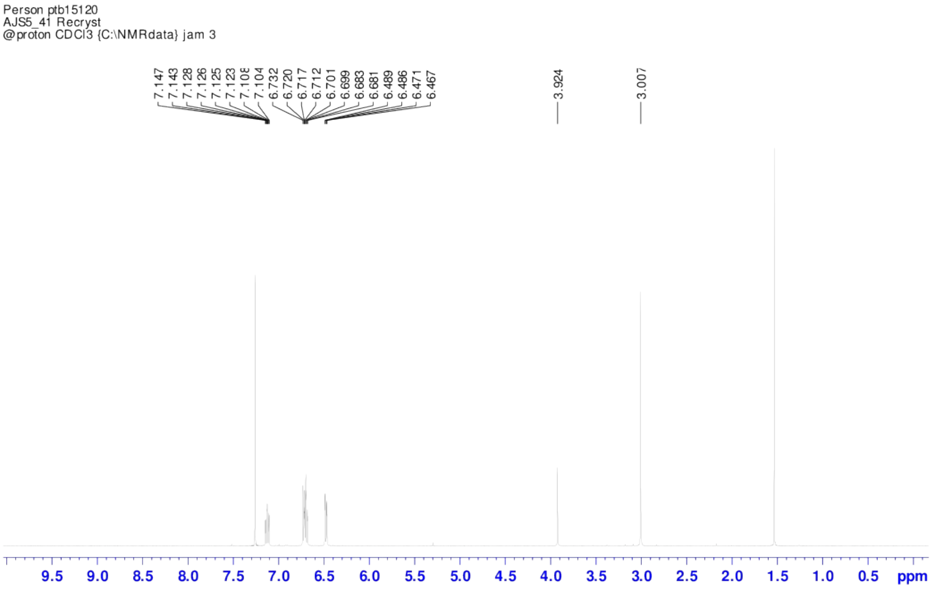

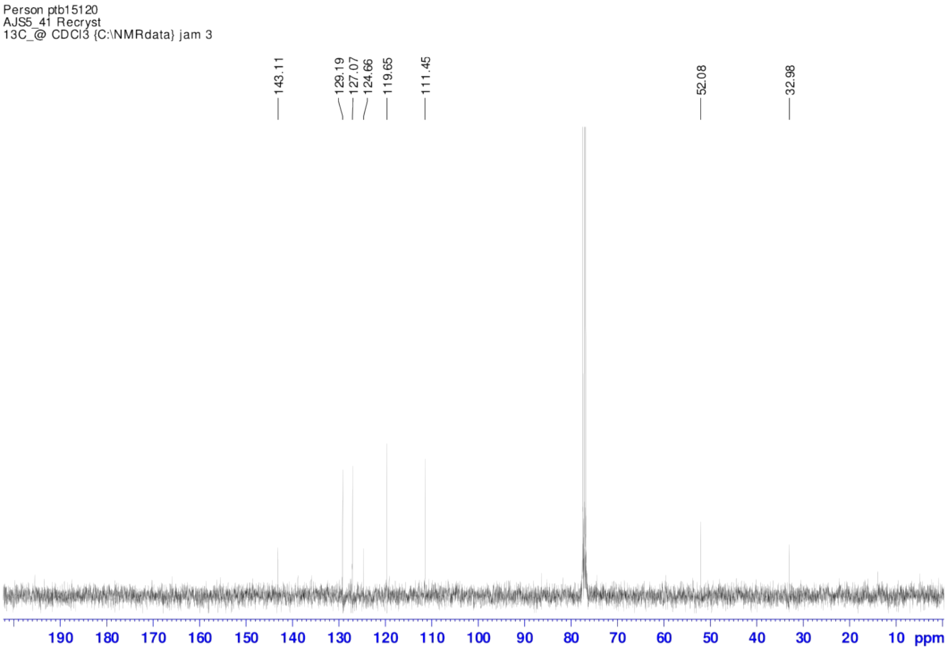

Cyclopentane-1,1-diyldibenzene 28

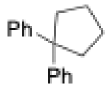

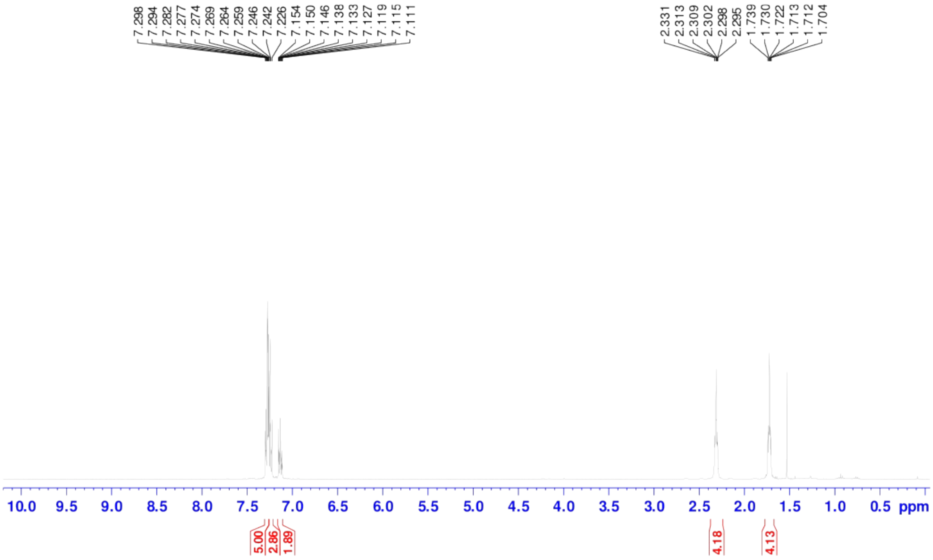

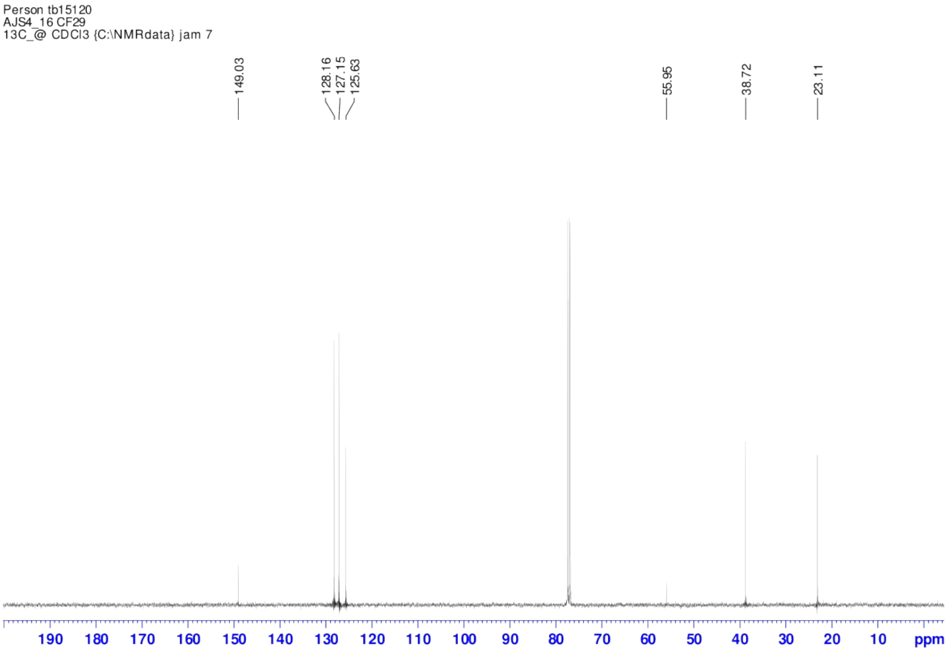

4-(10-Methyl-9,10-dihydroacridin-9-yl)butan-1-ol 29

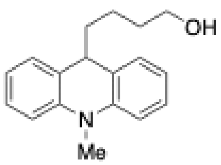

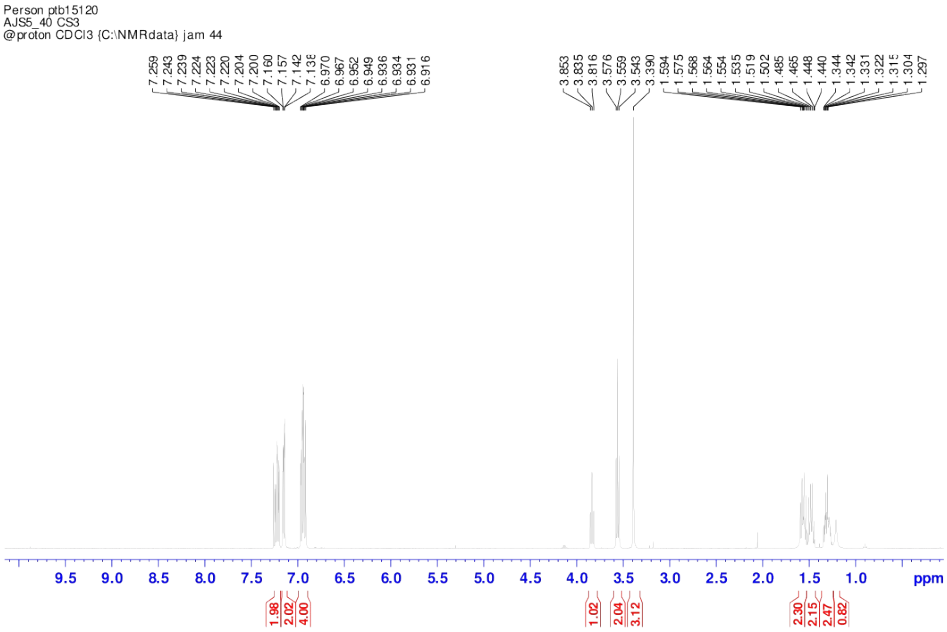

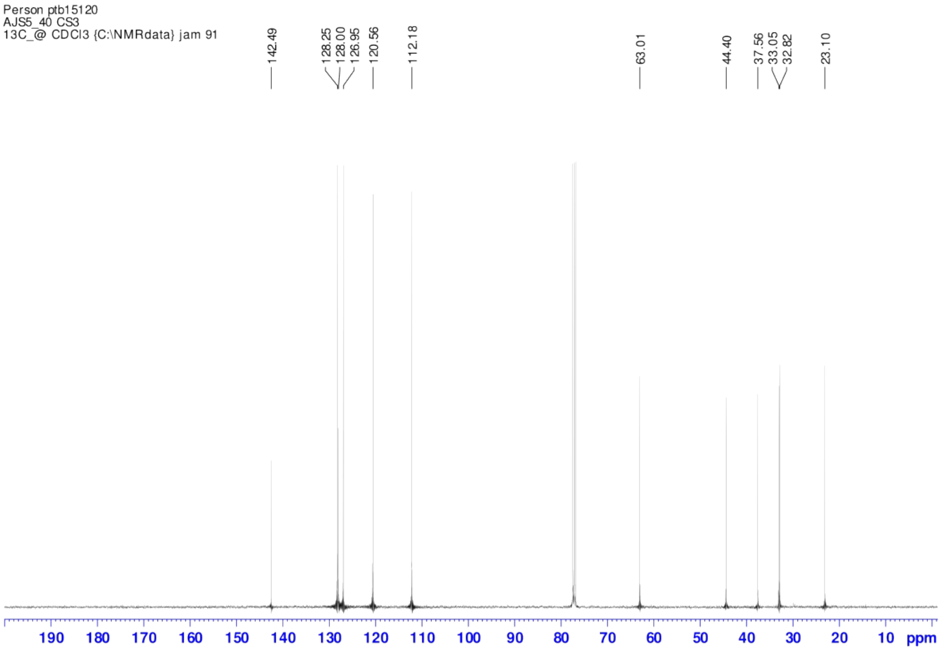

Spiro[cyclopentane-1,9'-xanthene] 31

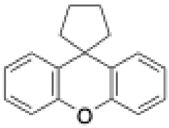

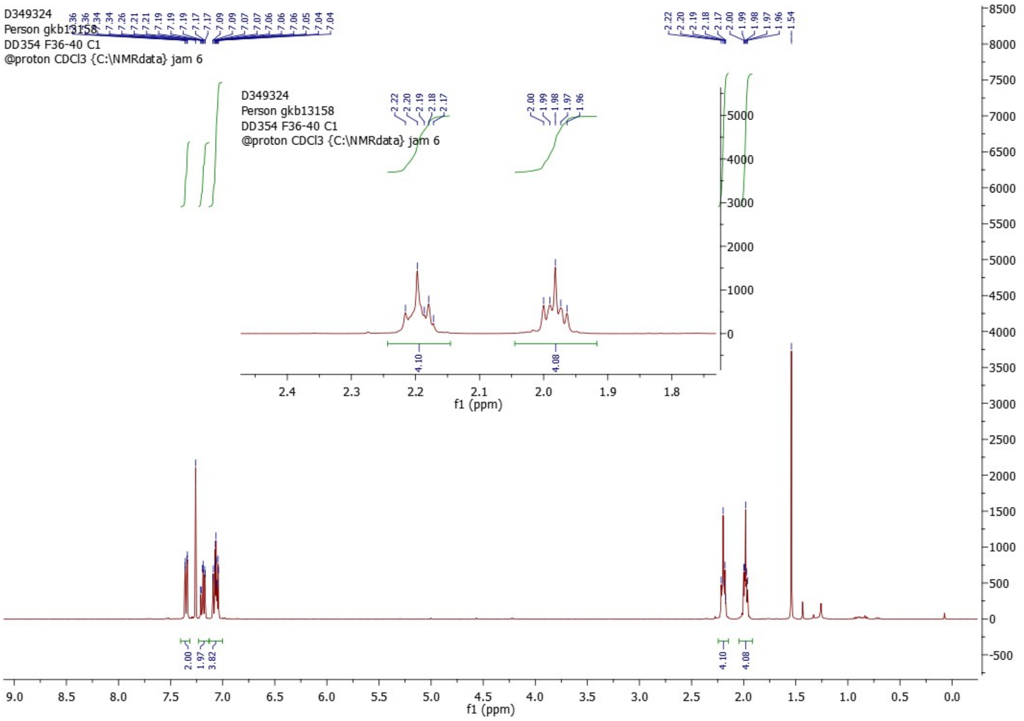

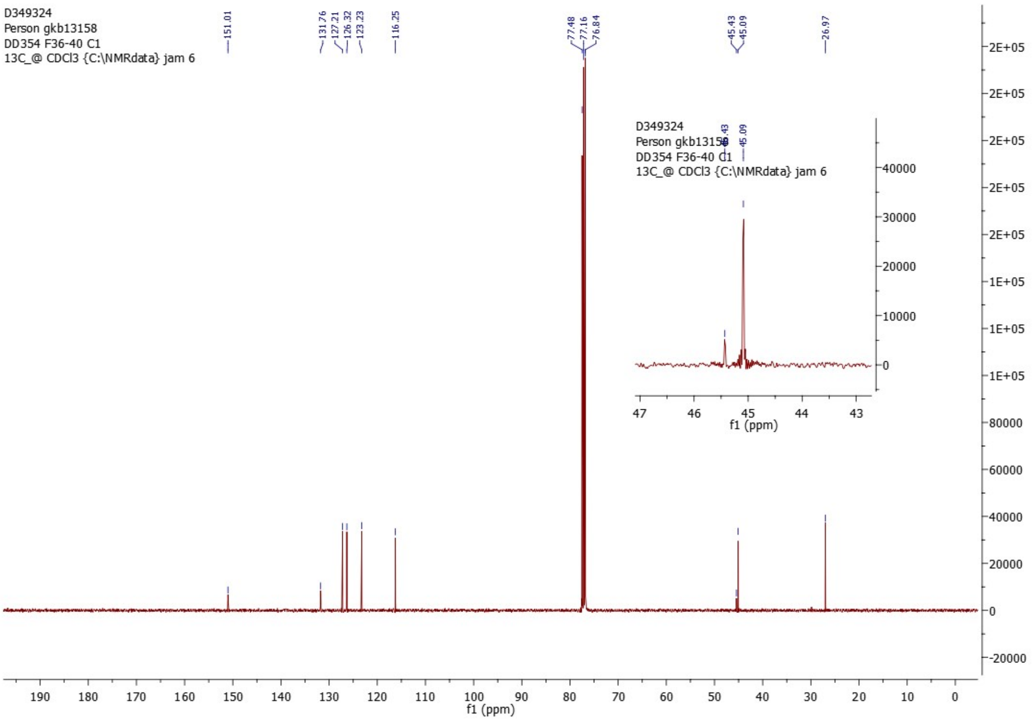

2-(1-Phenylcyclopropyl)pyridine 34

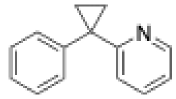

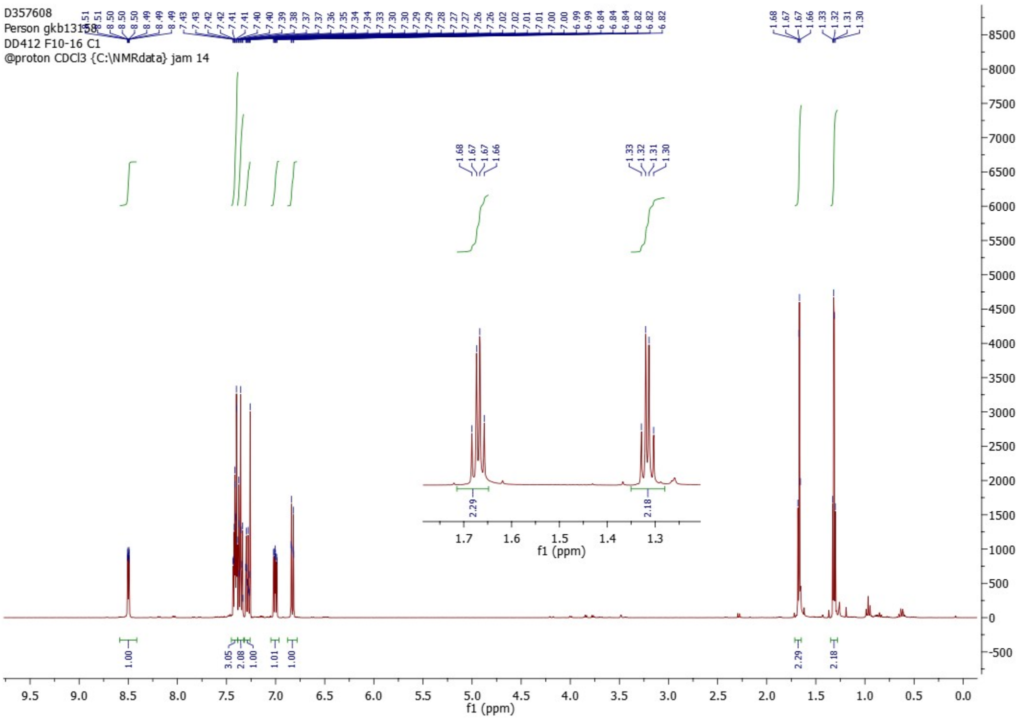

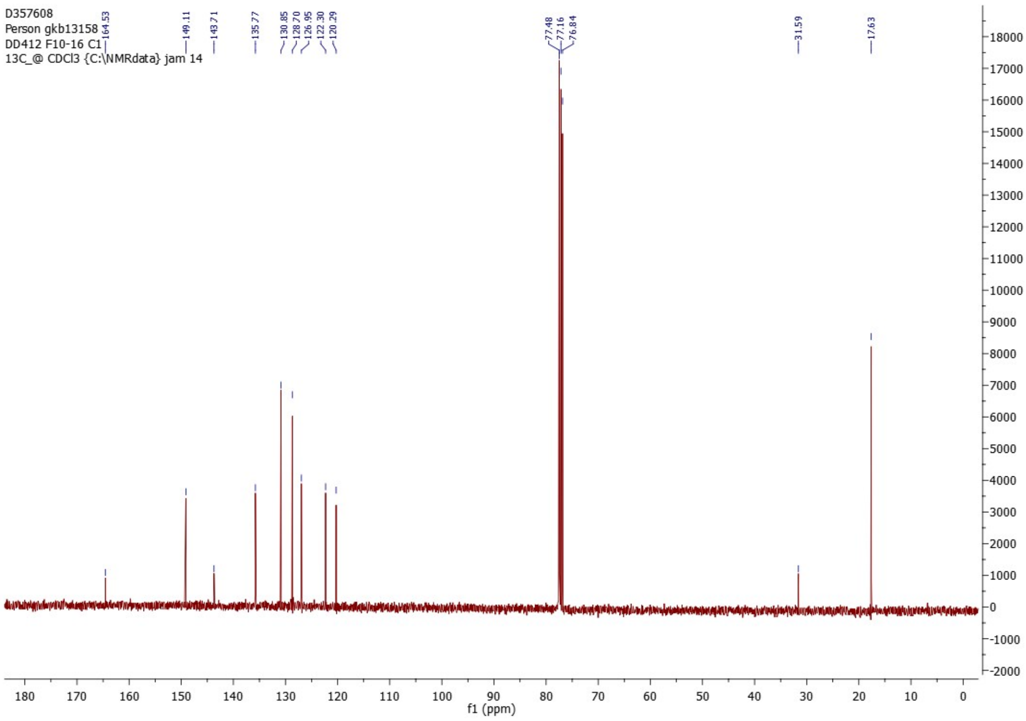

4-(1-Phenylcyclopropyl)pyridine 35

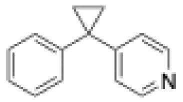

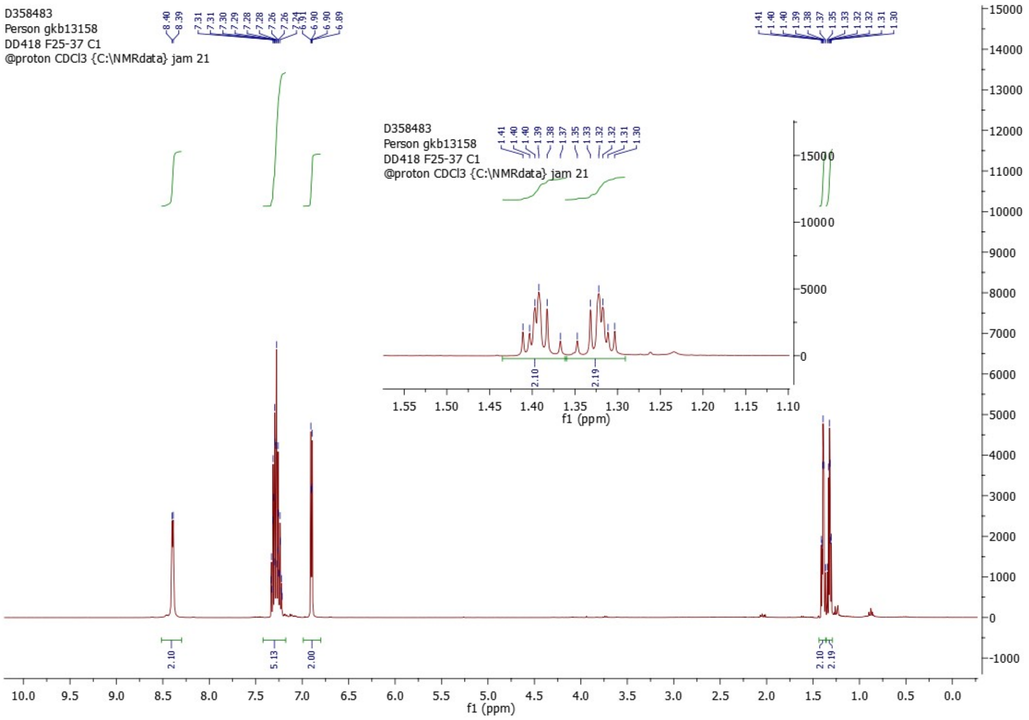

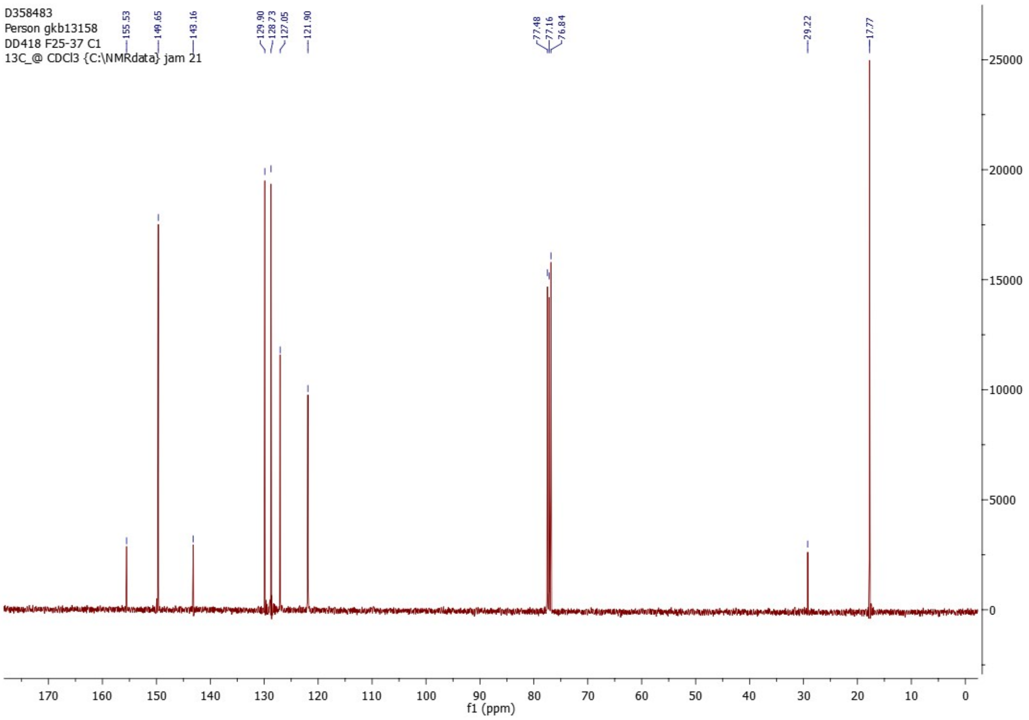

Cyclopropane-1,1-diyldibenzene 38

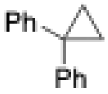

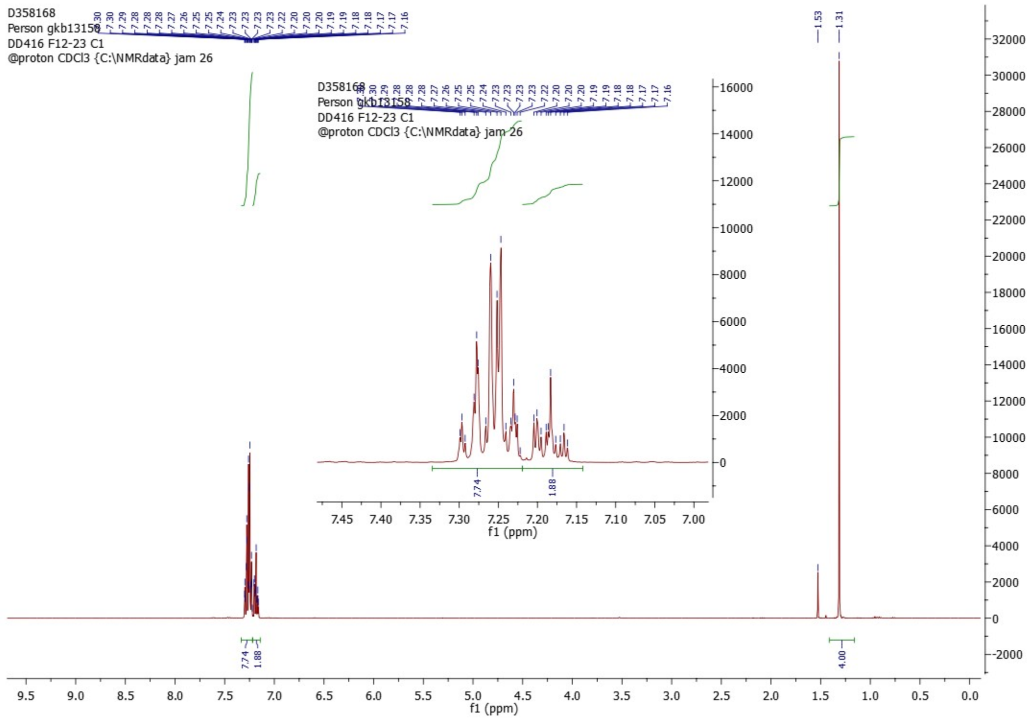

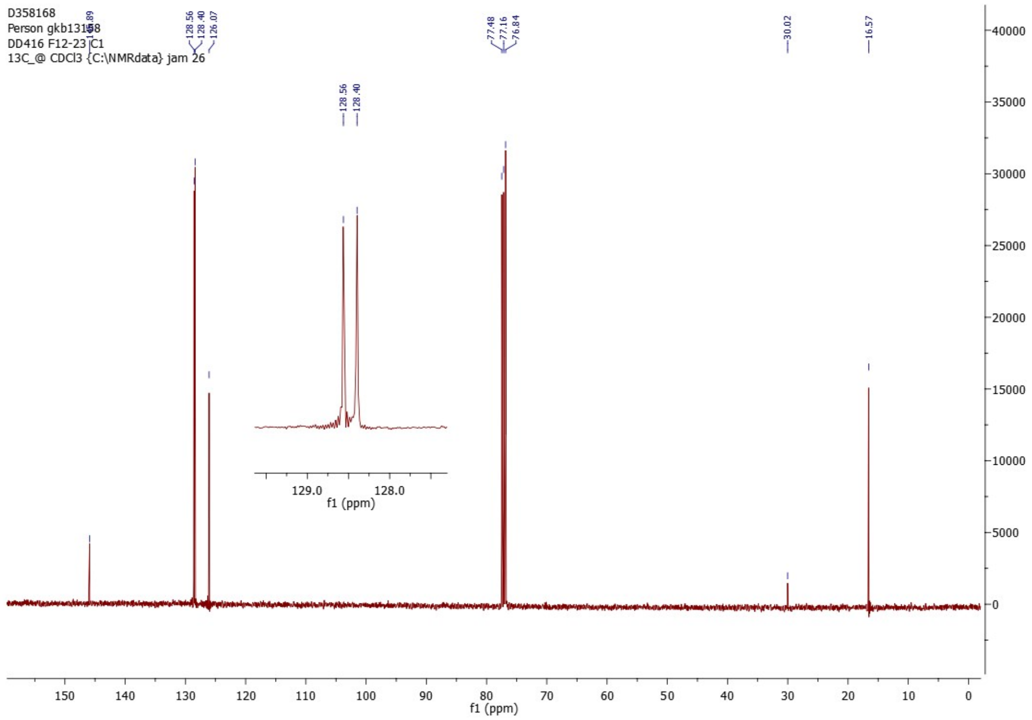

Spiro[cyclopropane-1,9'-xanthene] 39

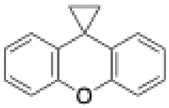

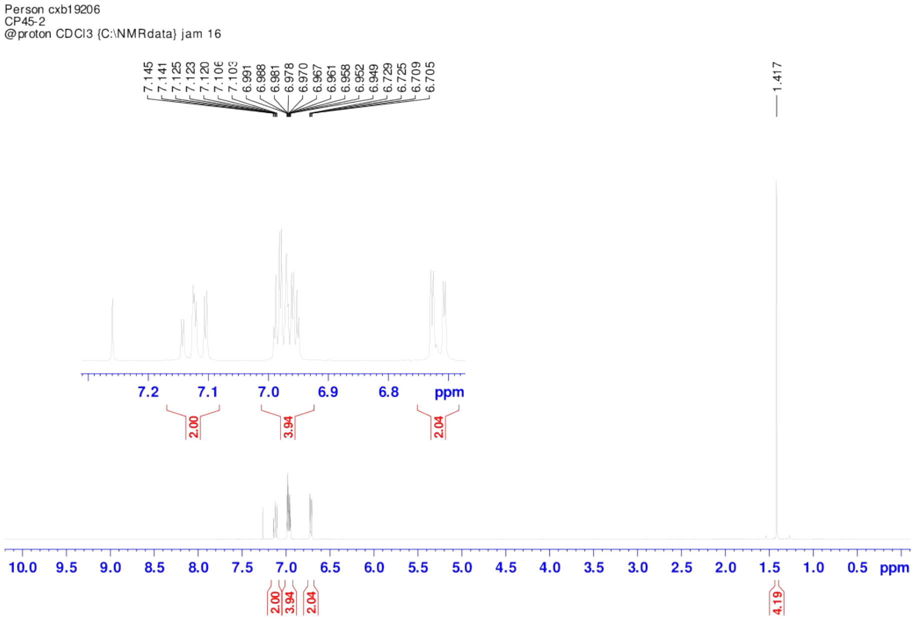

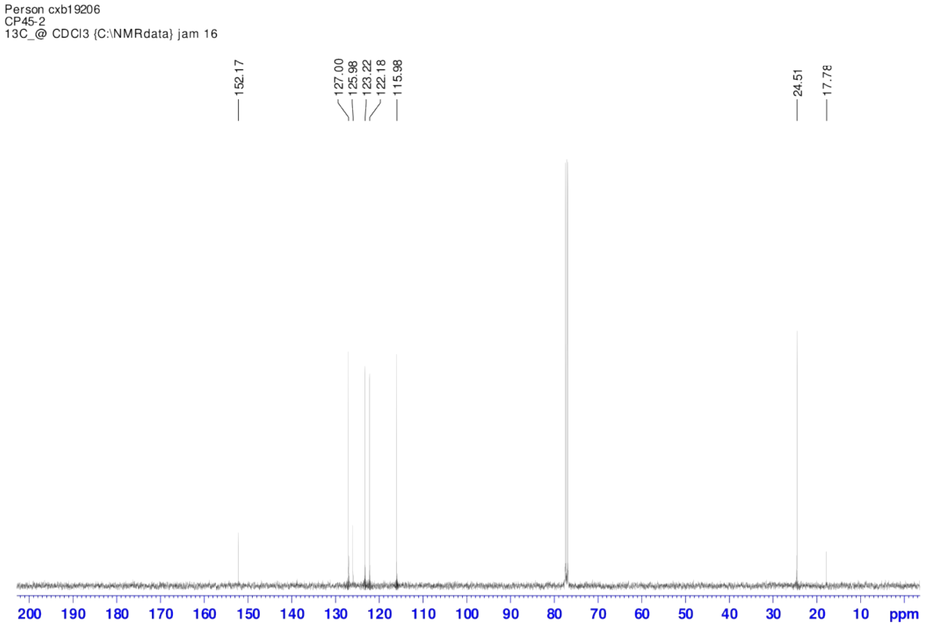

1-(*tert*-Butyl)-4-cyclopropylbenzene 41

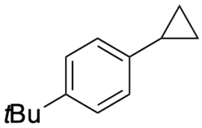

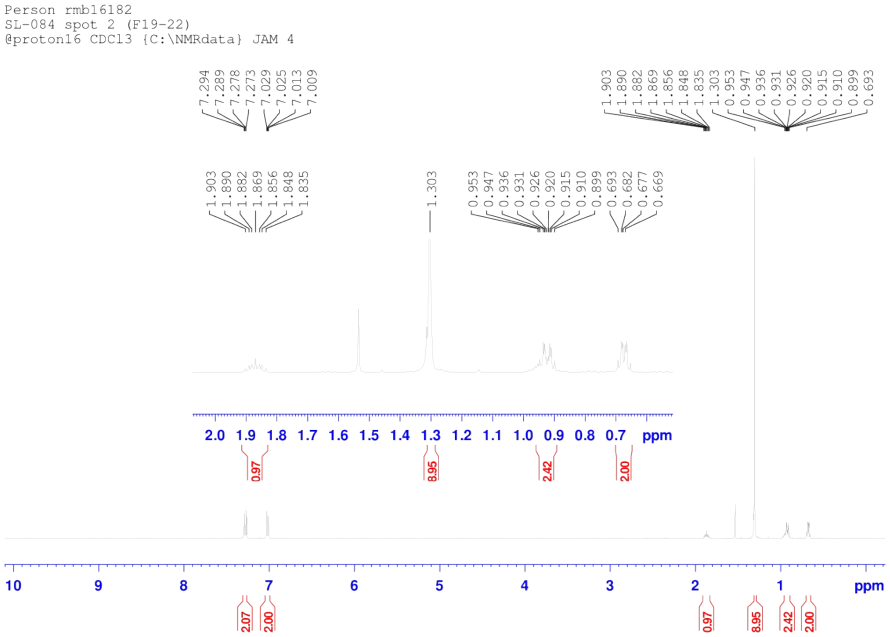

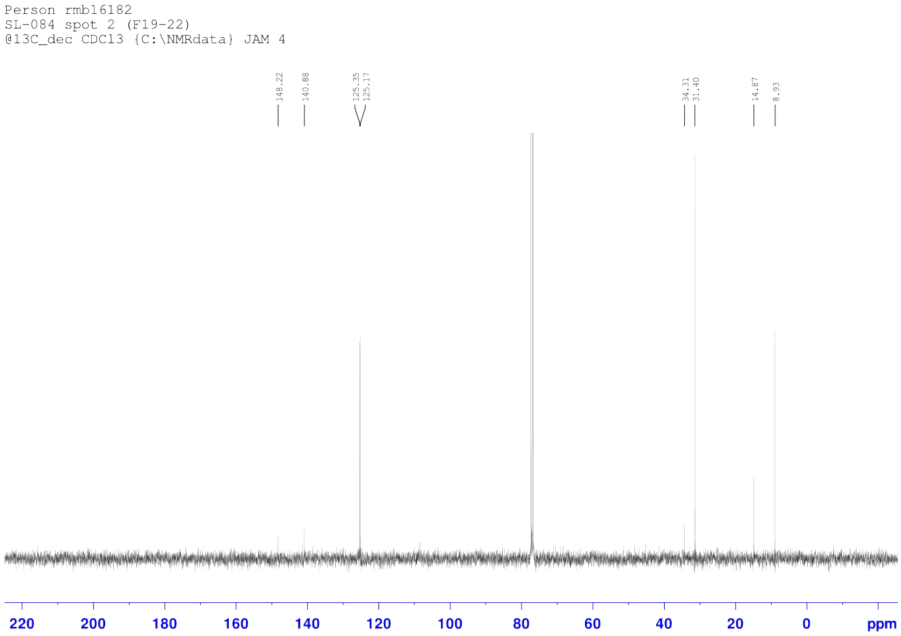

1-Cyclopropylnaphthalene 42

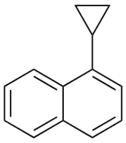

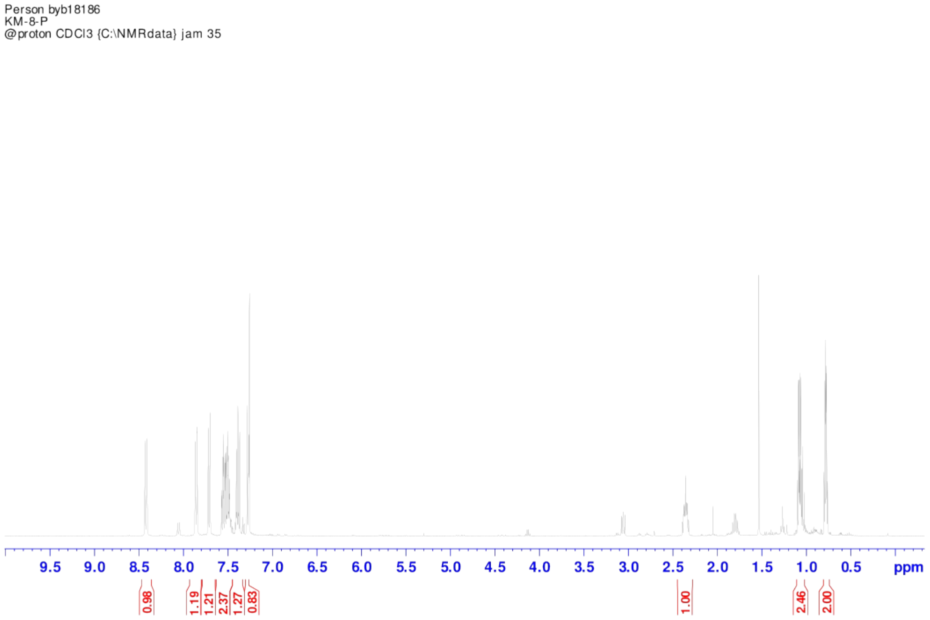

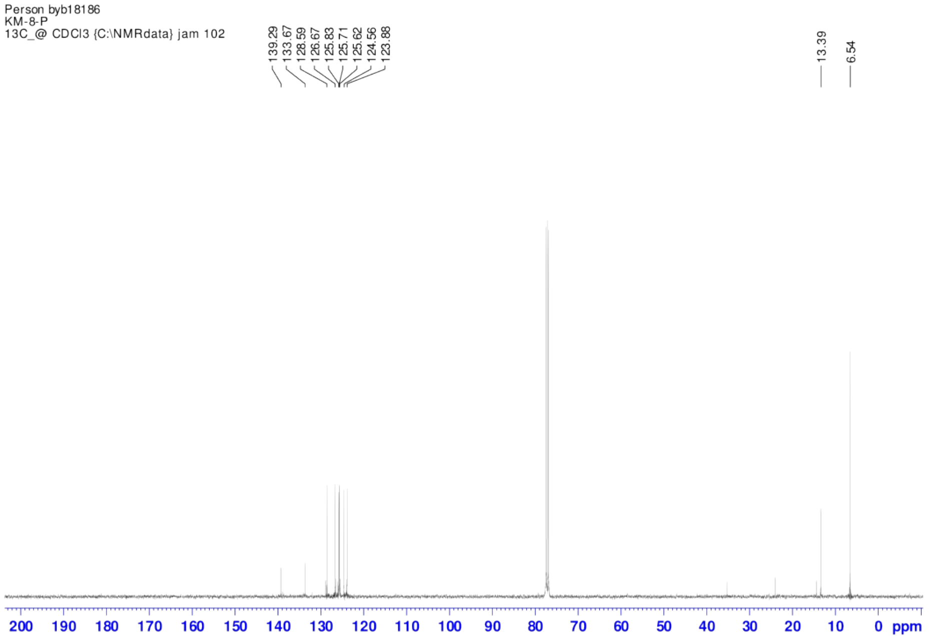

2-Cyclopropylnaphthalene 43

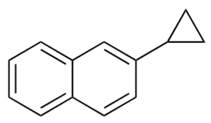

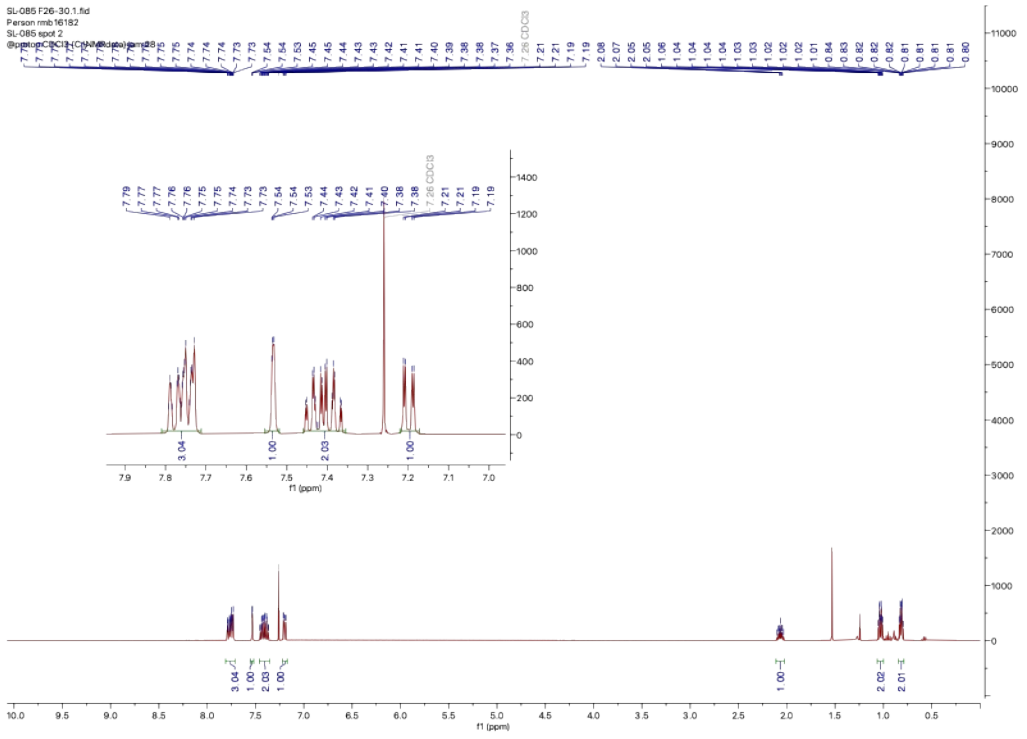

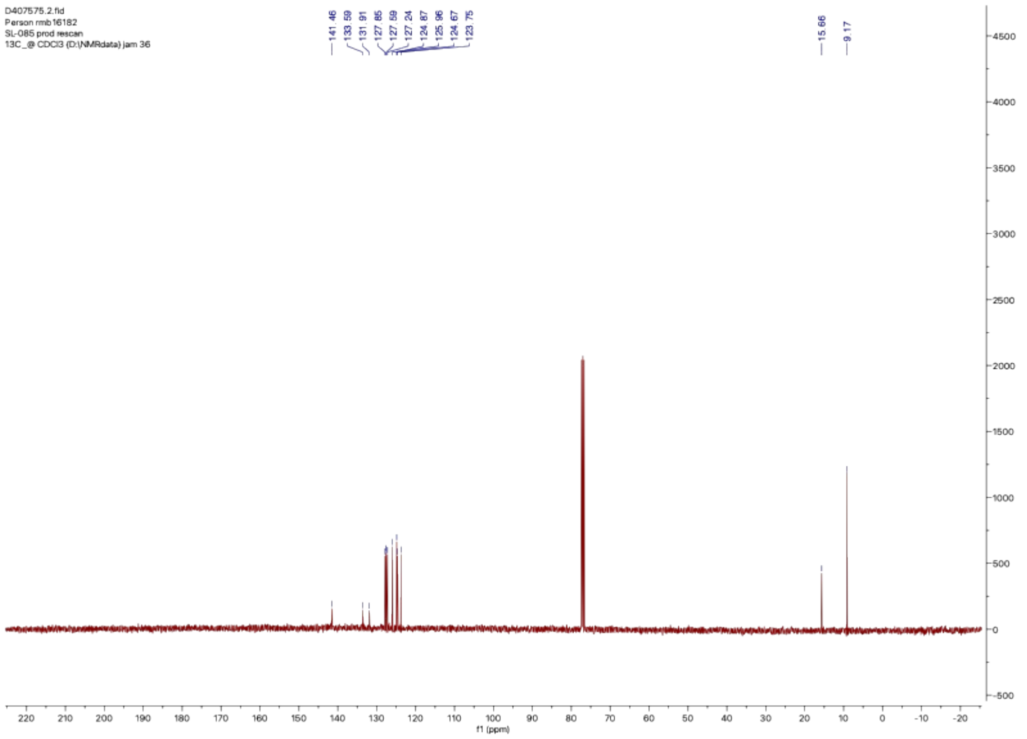

4-Cyclopropyl-1,1'-biphenyl 44

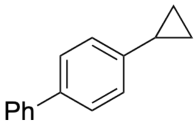

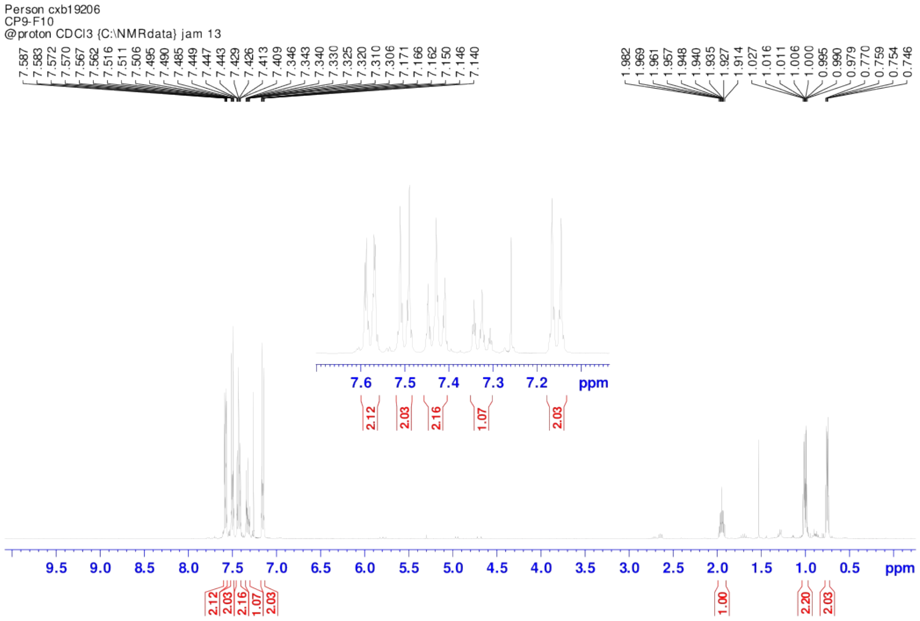

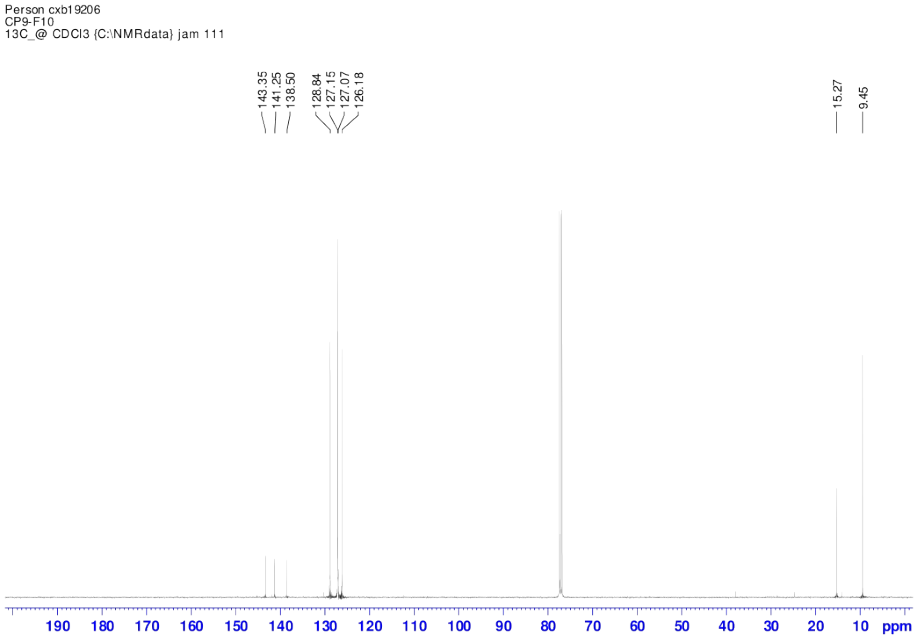

3-Cyclopropyl-1,1'-biphenyl 45

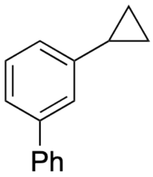

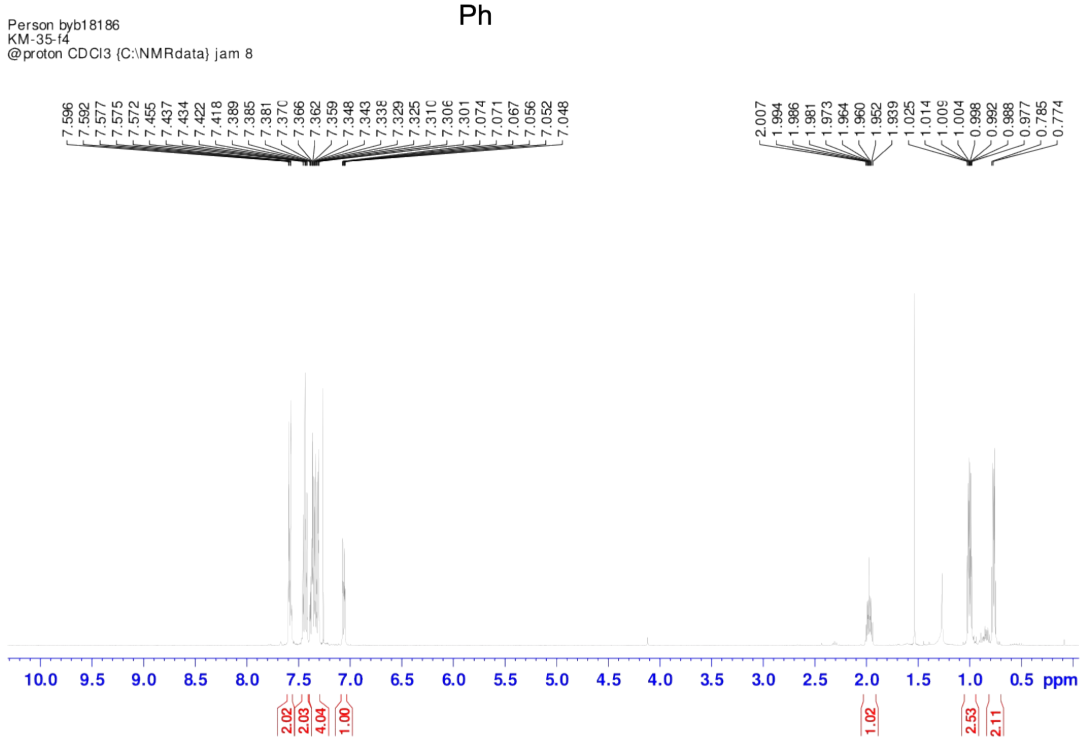

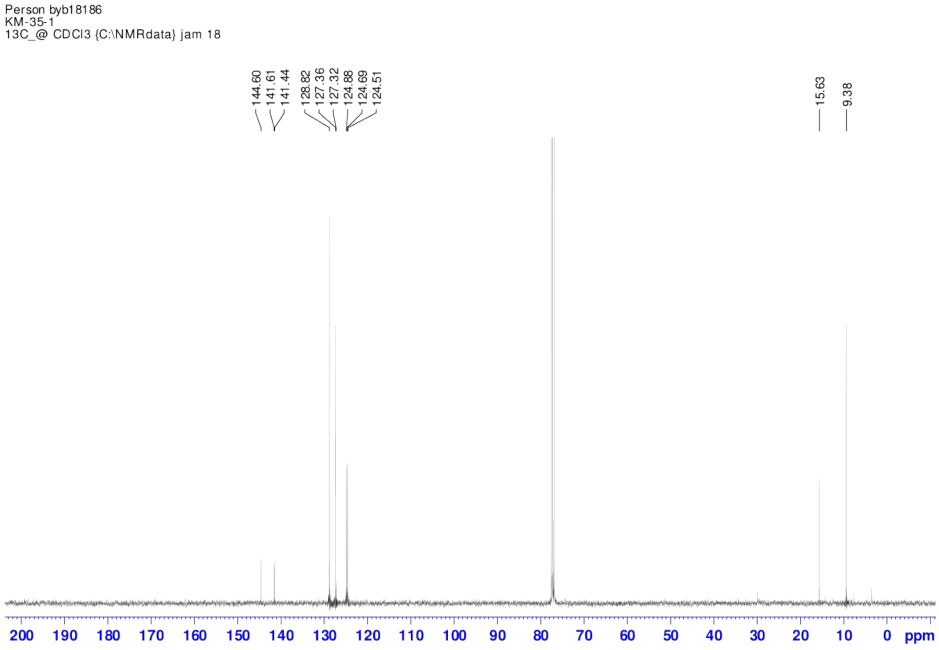

2-cyclopropyl-1,1'-biphenyl 46

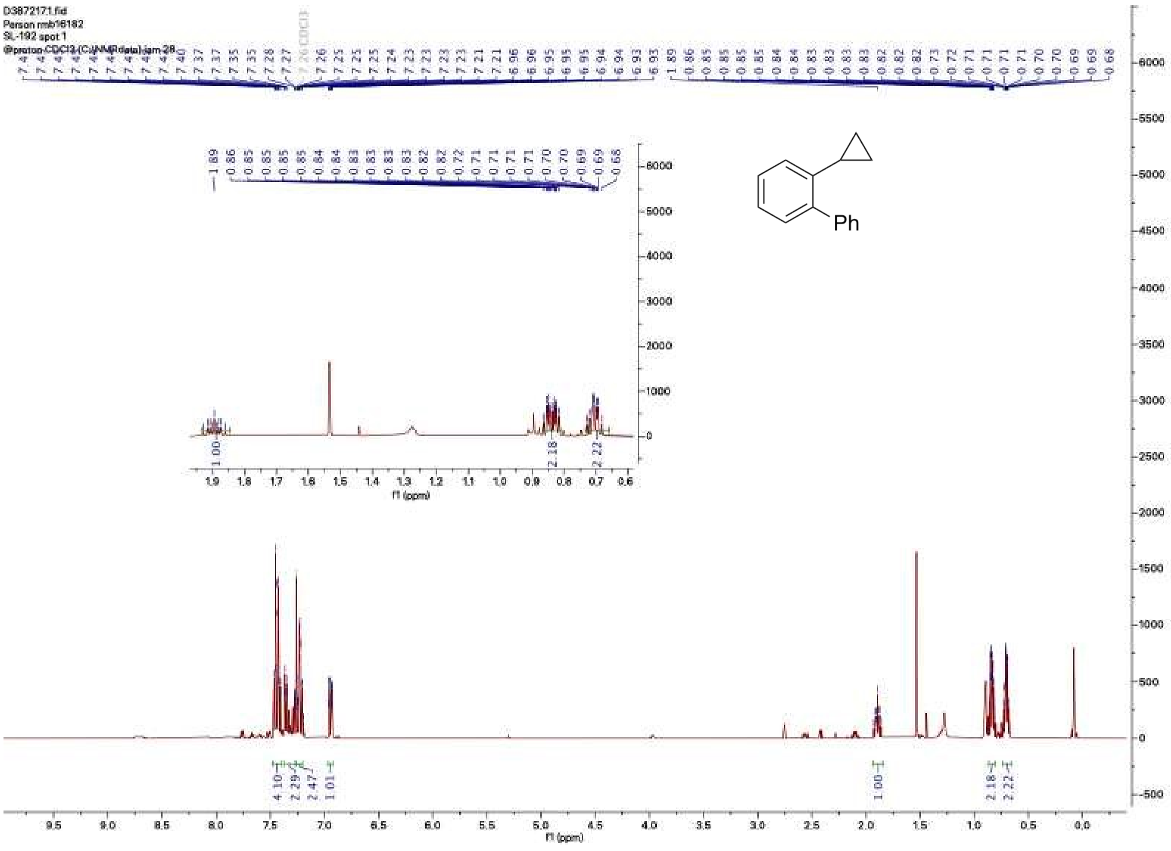

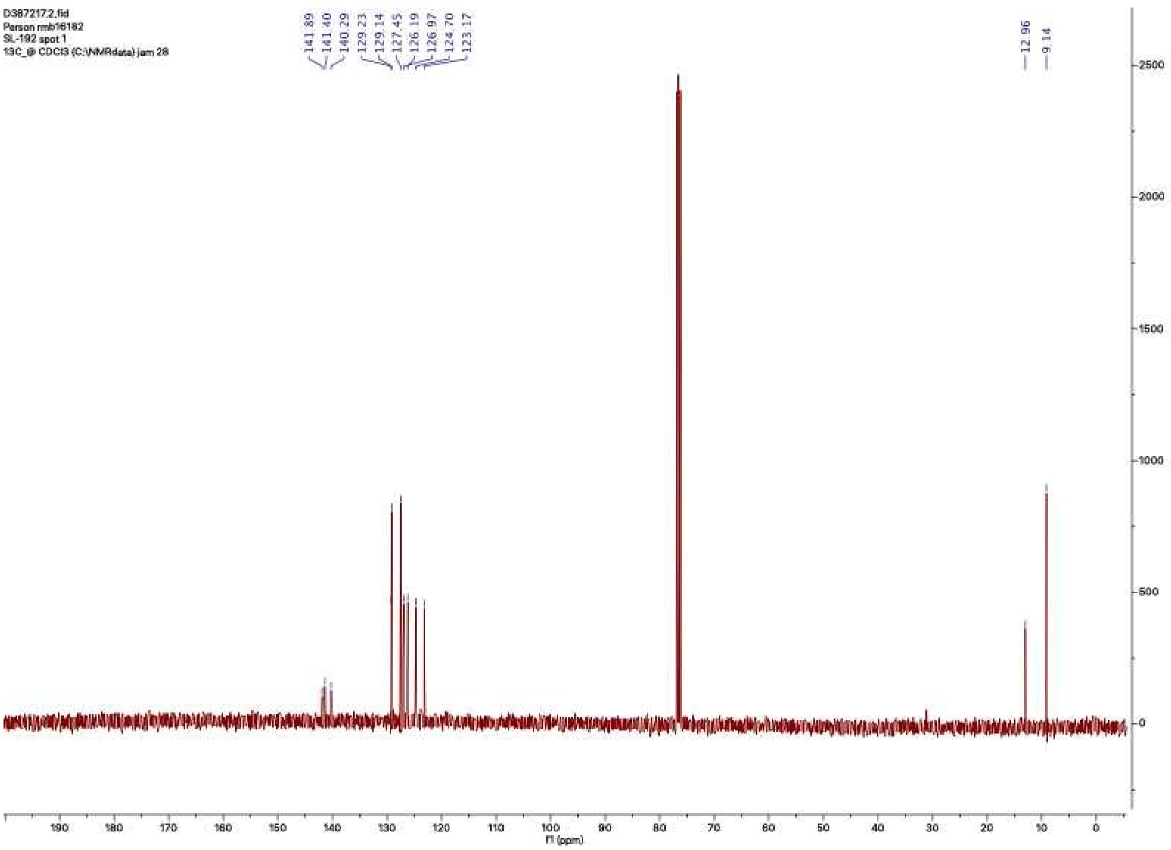

2-Cyclopropylquinoline 47

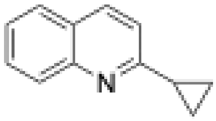

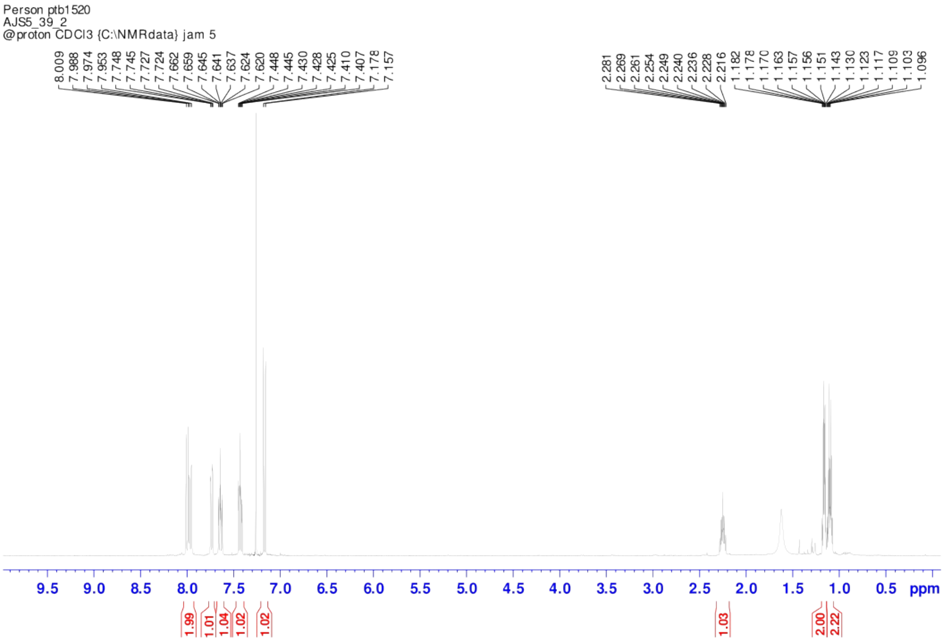

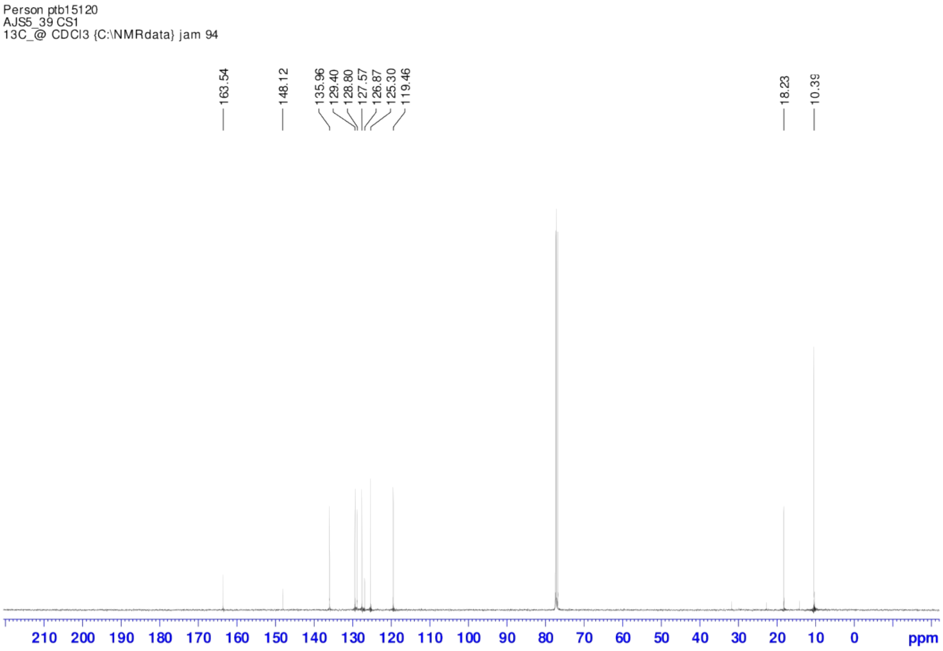

4-Cyclopropylpyridine 48

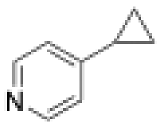

Person ptb15120
AJS5_46_2
@proton CDCl3 {C:\NMRdata} jam 1

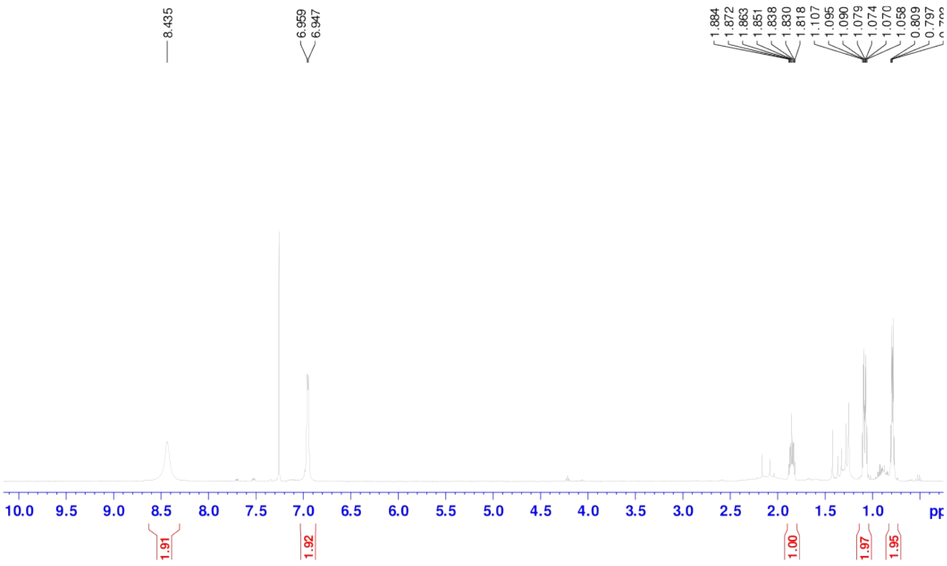

Person pttb15120
AJS5_83_2
13C_@ CDCl3 {C:\NMRdata} jam 90

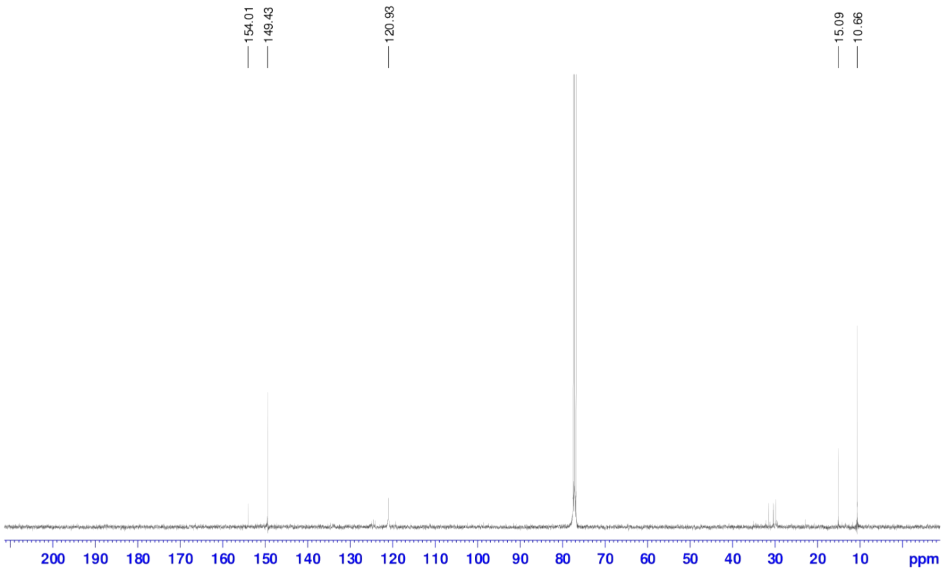

2-(4-Cyclopropylphenyl)thiophene 49

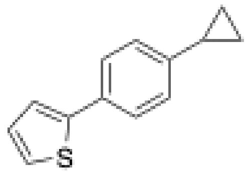

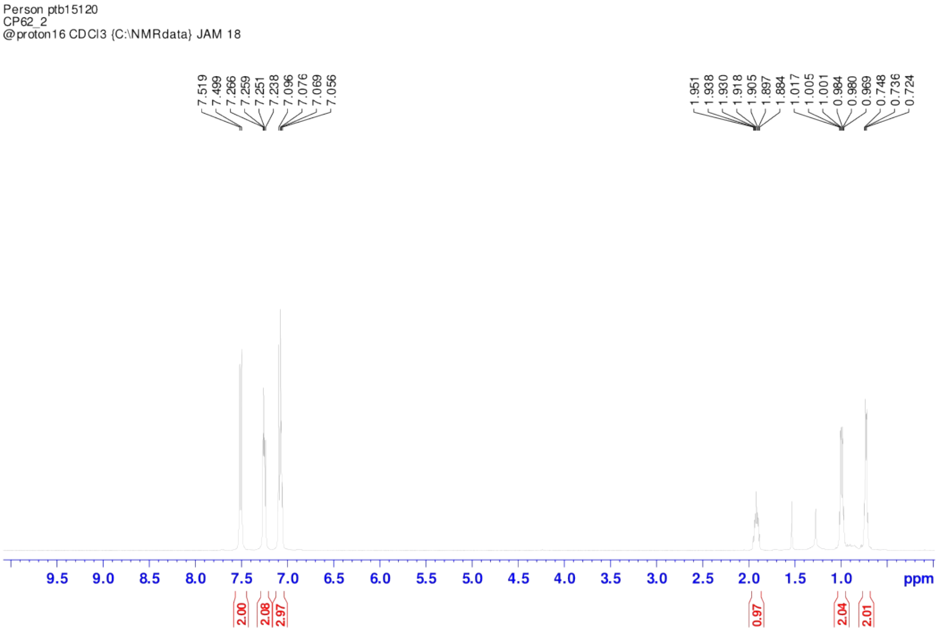

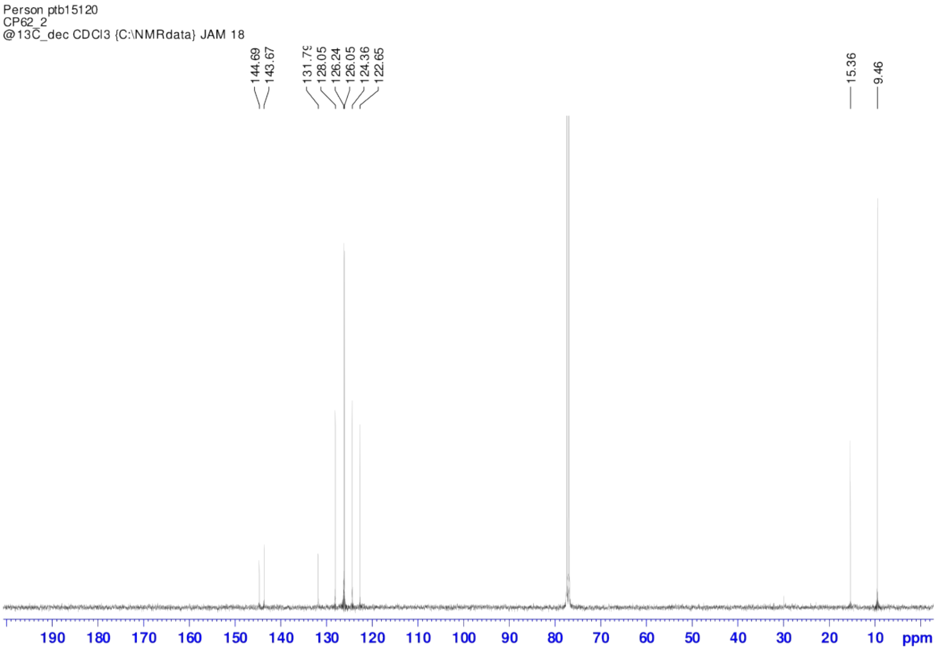

3-Cyclopropylbenzo[*b*]thiophene 50

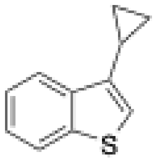

Person ptb15120
AJS5_63_2
@proton CDCl3 {C:\NMRdata} jam 90

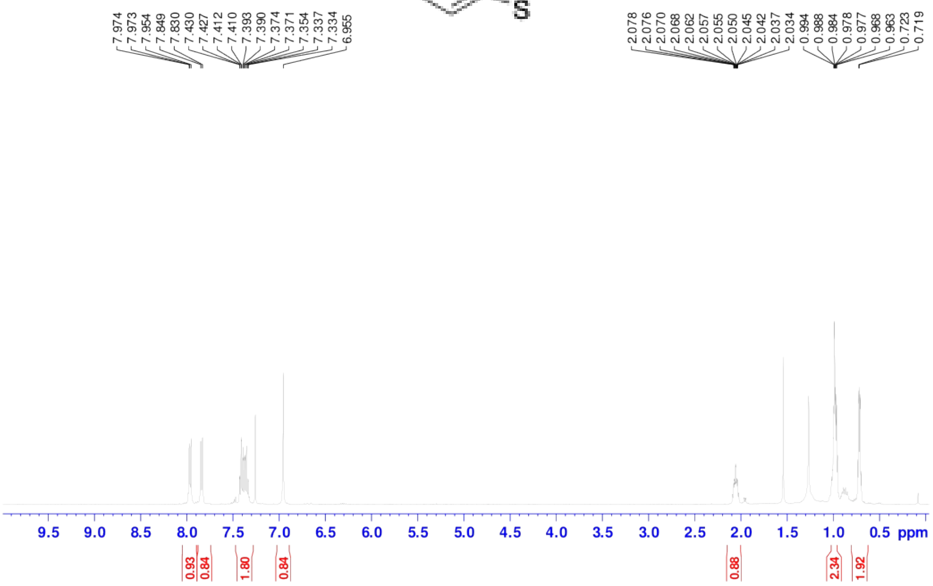

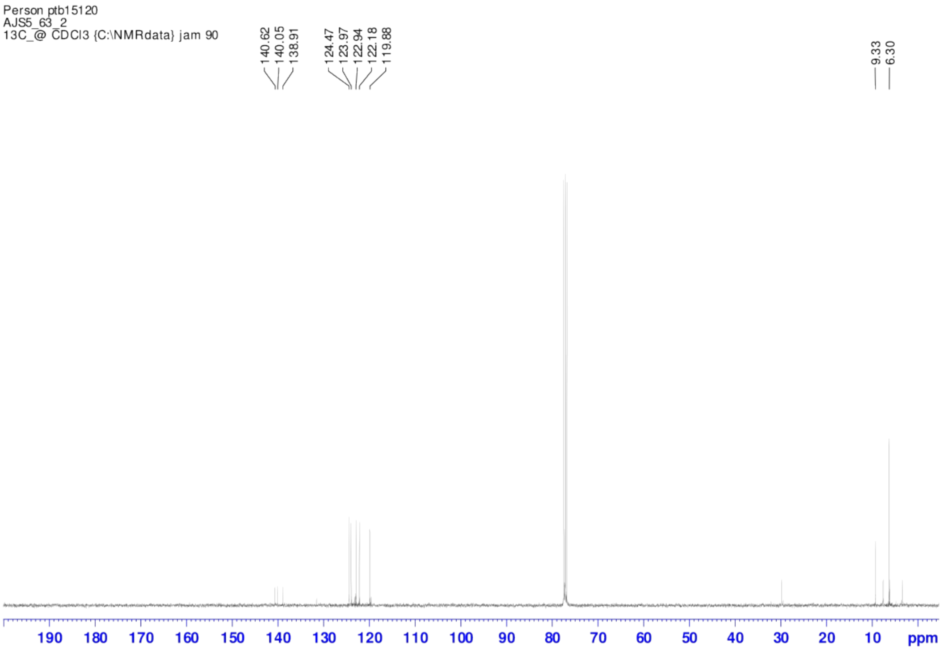

(3-(Benzo[*b*]thiophen-3-yl)propoxy)triethylsilane S55

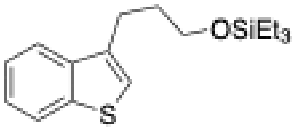

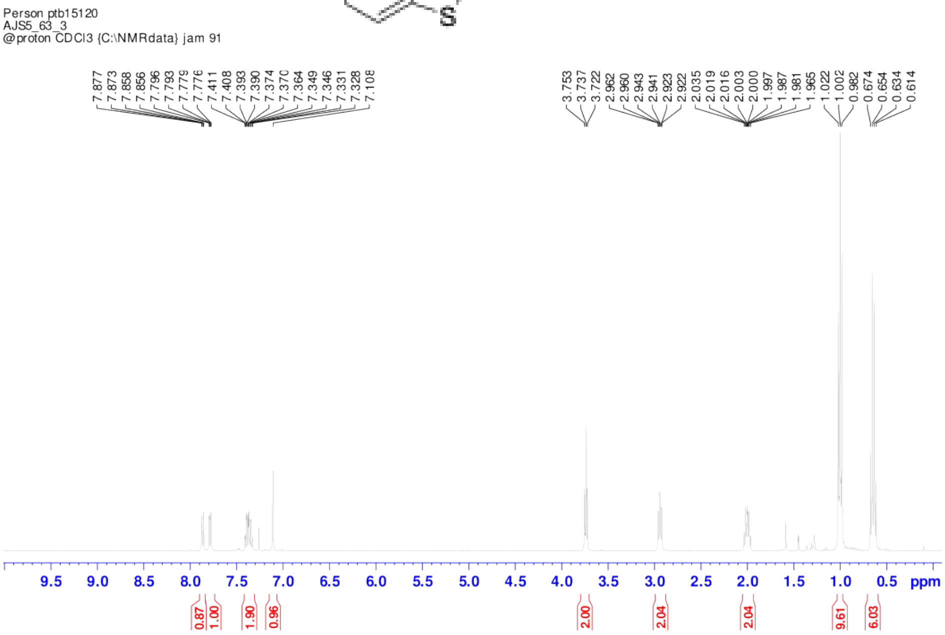

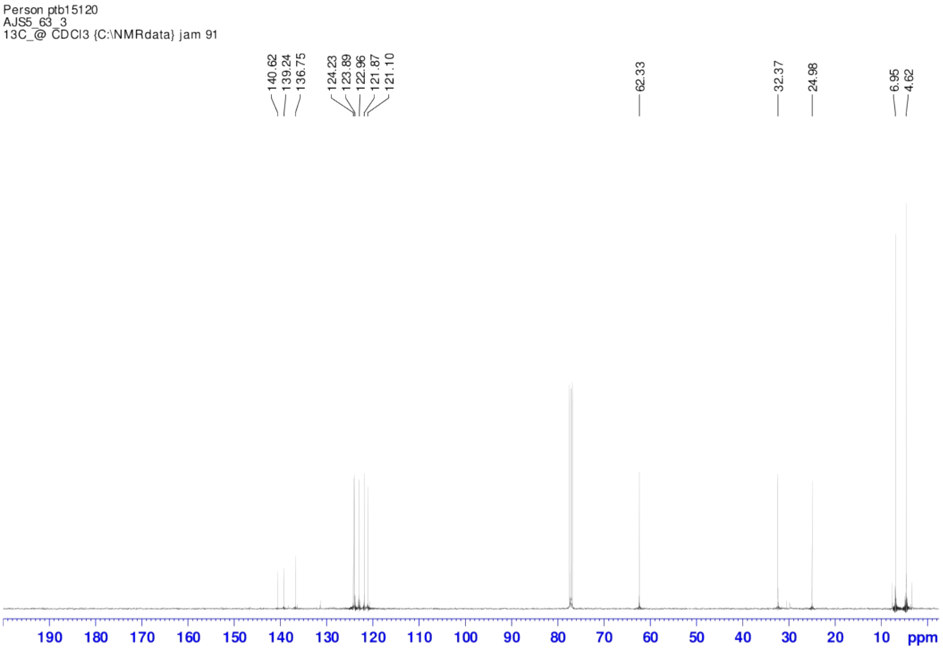

(3,3-Diphenylpropoxy)triisopropylsilane S57

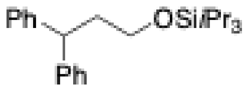

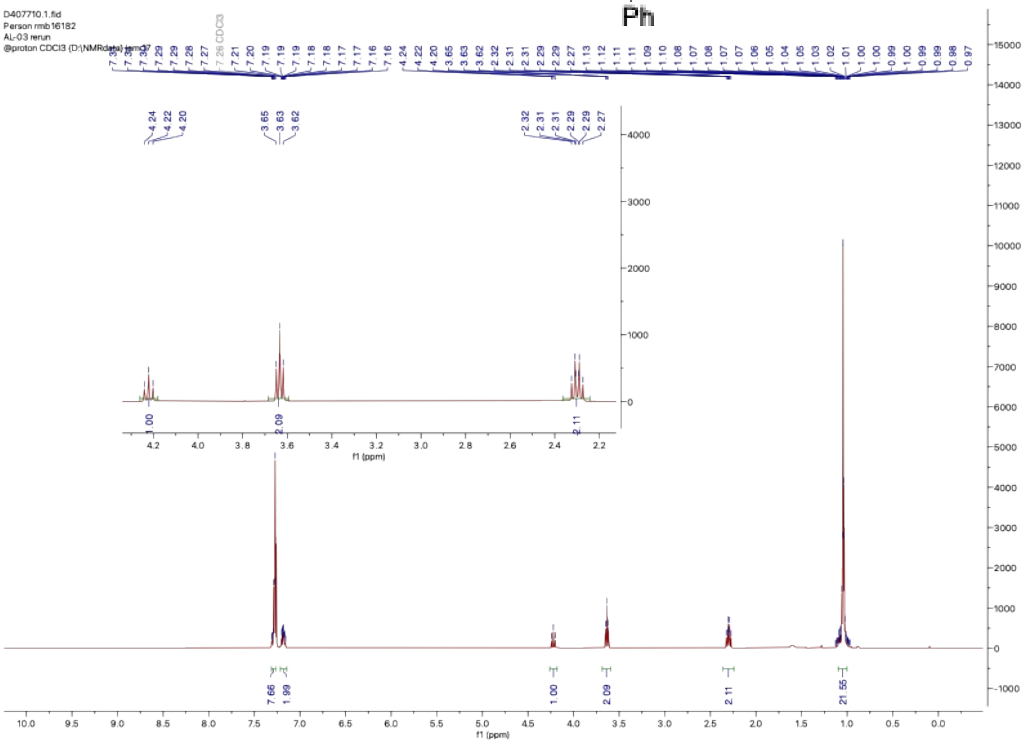

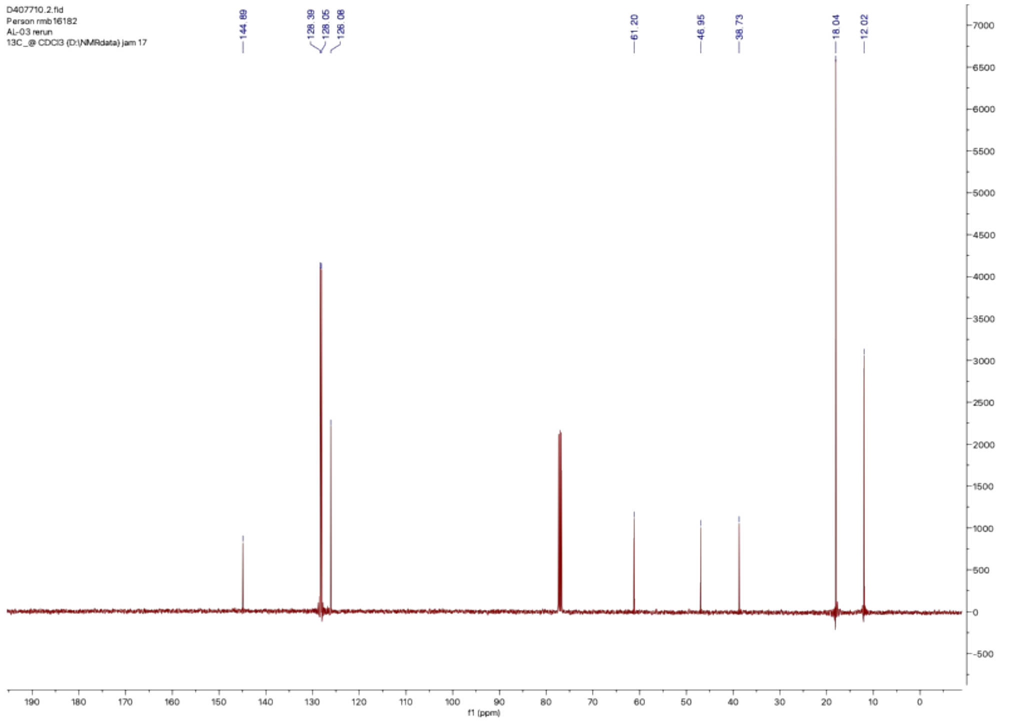

tert-Butyl(3,3-diphenylpropoxy)dimethylsilane S58

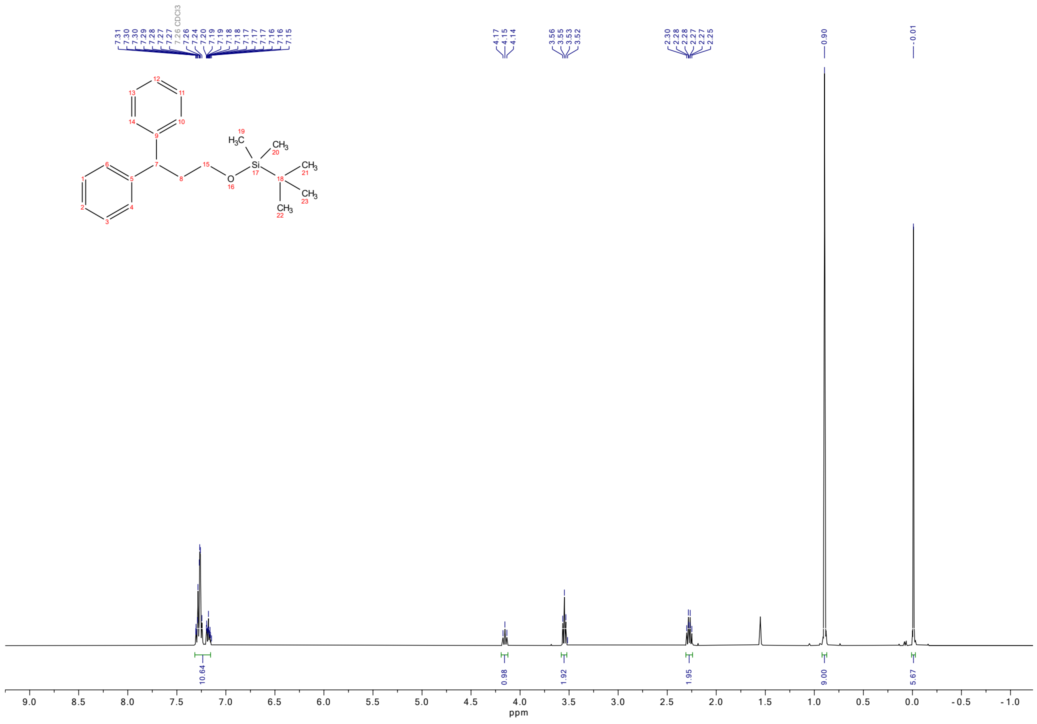

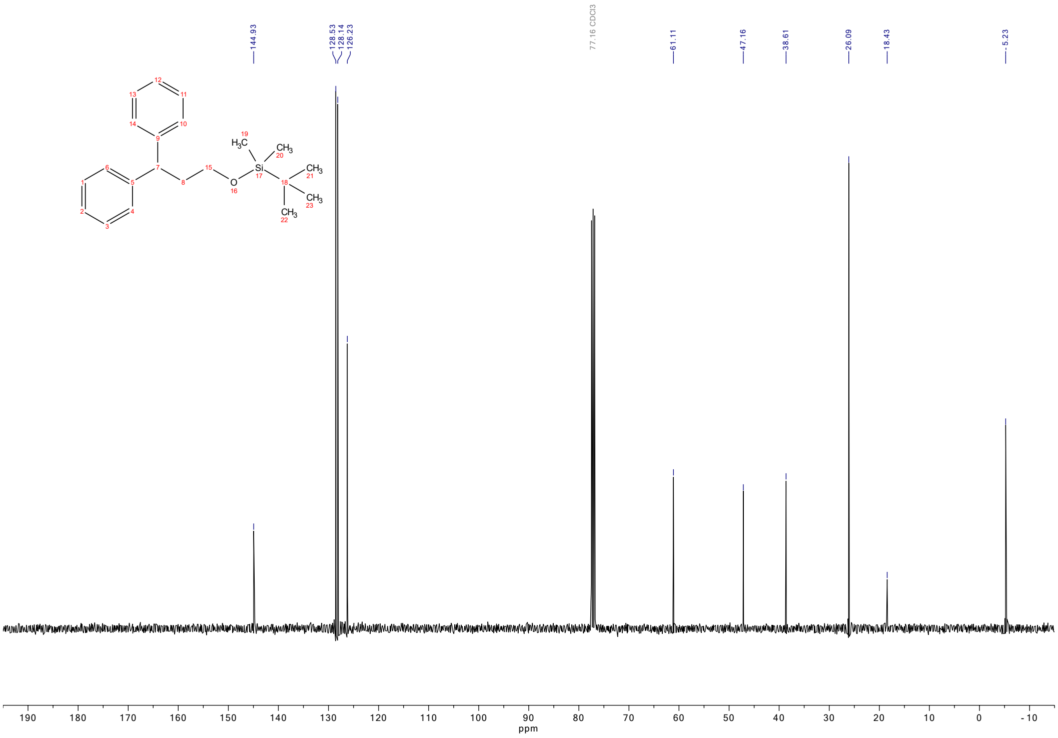

References

1 E. Tsui, A. J. Metrano, Y. Tsuchiya and R. R. Knowles, *Angew. Chem. Int. Ed.*, 2020, **59**, 11845–11849.
2 M. Altamura and E. Perrotta, *J. Org. Chem.*, 1993, **58**, 272–274.
3 C. M. M. Hendriks, P. Lamers, J. Engel and C. Bolm, *Adv. Synth. Catal.*, 2013, **355**, 3363–3368.
4 J. P. Wolfe, S. Wagaw and S. L. Buchwald, *J. Am. Chem. Soc.*, 1996, **118**, 7215–7216.
5 P. Weber, T. Scherpf, I. Rodstein, D. Lichte, L. T. Scharf, L. J. Gooßen and V. H. Gessner, *Angew. Chem. Int. Ed.*, 2019, **58**, 3203–3207.
6 R. Gericke, L. M. Doyle, E. R. Farquhar and A. R. McDonald, *Inorg. Chem.*, 2020, **59**, 13952–13961.
7 M. Gaydou, T. Moragas, F. Juliá-Hernández and R. Martin, *J. Am. Chem. Soc.*, 2017, **139**, 12161–12164.
8 Dimitrova, D. *Ph*D Thesis: *Investigating Novel Reagents in Organic Synthesis;* University of Strathclyde: Glasgow, 2022.
9 K. D. Collins, A. Rühling, F. Lied and F. Glorius, *Chem. Eur. J.*, 2014, **20**, 3800–3805.
10 M. Mikhael, W. Guo, D. J. Tantillo and S. E. Wengryniuk, *Adv. Synth. Catal.*, 2021, **363**, 4867–4875.
11 M. H. S. A. Hamid, C. L. Allen, G. W. Lamb, A. C. Maxwell, H. C. Maytum, A. J. A. Watson and J. M. J. Williams, *J. Am. Chem. Soc.*, 2009, **131**, 1766–1774.
12 C.-H. Ma, Y. Ji, J. Zhao, X. He, S.-T. Zhang, Y.-Q. Jiang and B. Yu, *Chin J. Catal.*, 2022, **43**, 571–583.
13 P. Sauerberg, I. Pettersson, L. Jeppesen, P. S. Bury, J. P. Mogensen, K. Wassermann, C. L. Brand, J. Sturis, H. F. Wöldike, J. Fleckner, A.-S. T. Andersen, S. B. Mortensen, L. A. Svensson, H. B. Rasmussen, S. V. Lehmann, Z. Polivka, K. Sindelar, V. Panajotova, L. Ynddal and E. M. Wulff, *J. Med. Chem.*, 2002, **45**, 789–804.
14 A. E. Bosnidou and K. Muñiz, *Angew. Chem. Int. Ed.*, 2019, **58**, 7485–7489.
15 M. Kobayashi, S. Itoh, K. Yoshimura, Y. Tsukamoto and Y. Obora, *J. Org. Chem.*, 2020, **85**, 11952–11958.
16 C. Huang, W. Ma, X. Zheng, M. Xu, X. Qi and Q. Lu, *J. Am. Chem. Soc.*, 2022, **144**, 1389–1395.
17 C. Sun, Q. Zhou, C.-Y. Li, Z.-W. Hou and L. Wang, *Org. Lett.*, 2024, **26**, 883–888.
18 S. E. Sloane, A. Reyes, Z. P. Vang, L. Li, K. T. Behlow and J. R. Clark, *Org. Lett.*, 2020, **22**, 9139–9144.
19 A. B. Sanford, T. A. Thane, T. M. McGinnis, P.-P. Chen, X. Hong and E. R. Jarvo, *J. Am. Chem. Soc.*, 2020, **142**, 5017–5023.
20 J. Zheng, Y. Huang and Z. Li, *Org. Lett.*, 2013, **15**, 5064–5067.
21 C. Liu, M. Wang, S. Liu, Y. Wang, Y. Peng, Y. Lan and Q. Liu, *Angew. Chemie Int. Ed.*, 2021, **60**, 5108–5113.
22 J. A. Gurak and K. M. Engle, *ACS Catal.*, 2018, **8**, 8987–8992.
23 A. Martins, D. Alberico and M. Lautens, *Org. Lett.*, 2006, **8**, 4827–4829.
24 L. J. Perez, W.-L. Ng, P. Marano, K. Brook, B. L. Bassler and M. F. Semmelhack, *J. Med. Chem.*, 2012, **55**, 9669–9681.
25 P. C. Too, G. H. Chan, Y. L. Tnay, H. Hirao and S. Chiba, *Angew. Chem. Int. Ed.*, 2016, **55**, 3719–3723.
26 G. A. Pinna, G. Cignarella, G. Loriga, G. Murineddu, J. M. Mussinu, S. Ruiu, P. Fadda and W. Fratta, *Bioorganic Med. Chem.*, 2002, **10**, 1929–1937.
27 Q. Zhu, J. Long, X. Song, K. Wang, J. Zeng and Y. Fan, *J. Org. Chem.*, 2024, **89**, 3726–3731.
28 D. W. Kim, D. J. Hong, K. S. Jang and D. Y. Chi, *Adv. Synth. Catal.*, 2006, **348**, 1719–1727.

29 E. C. Creencia, K. Taguchi and T. Horaguchi, *J. Heterocycl. Chem.*, 2008, **45**, 837–843.
30 A. K. Colter, C. C. Lai, A. G. Parsons, N. B. Ramsey and G. Saito, *Can. J. Chem.*, 1985, **63**, 445–451.
31 S. Fukuzumi, K. Ohkubo and J. Otera, *J. Org. Chem.*, 2001, **66**, 1450–1454.
32 J. W. Wilt, R. A. Dabek and K. C. Welzel, *J. Org. Chem.*, 1972, **37**, 425–430.
33 M. Puthanveedu, V. Polychronidou and A. P. Antonchick, *Org. Lett.*, 2019, **21**, 3407–3411.
34 Z. Zhang, Q. He, X. Zhang and C. Yang, *Org. Biomol. Chem.*, 2022, **20**, 1969–1973.
35 J. Choi, G. Laudadio, E. Godineau and P. S. Baran, *J. Am. Chem. Soc.*, 2021, **143**, 11927–11933.
36 R. L. Svec and P. J. Hergenrother, *Angew. Chem. Int. Ed.*, 2020, **59**, 1857–1862.
37 M. Koishi, K. Tomota, M. Nakamoto and H. Yoshida, *Adv. Synth. Catal.*, 2023, **365**, 682–686.
38 Y.-Z. Yu, J. Bai, J.-M. Peng, J.-S. Yao and C.-X. Zhuo, *J. Am. Chem. Soc.*, 2023, **145**, 8781–8787.
39 JP2005247691A, 2005.
40 S. Umio, I. Ueda and H. Nojima, *J. Med. Chem.*, 1972, **15**, 855–856.
41 K. D. Collins, A. Rühling, F. Lied and F. Glorius, *Chem. Eur. J.*, 2014, **20**, 3800–3805.
